# Metal-free sustainable synthesis of oximes and hydrazones from benzyl alcohol and benzylamine

**DOI:** 10.1039/d6ra02204a

**Published:** 2026-05-26

**Authors:** Rupali B. Devgunde, Bapurao D. Rupanawar, Prasad B. Rupnavar, Suresh B. Waghmode

**Affiliations:** a Department of Chemistry, Savitribai Phule Pune University (Formerly University of Pune) Ganeshkhind Pune 411007 India suresh.waghmode@gmail.com suresh.waghmode@unipune.ac.in; b Chemical Engineering & Process Development Division, CSIR-National Chemical Laboratory Dr Homi Bhabha Road Pune 411008 India

## Abstract

Herein, we report a one-pot synthesis of oximes and hydrazones *via* oxidative coupling of alcohols and amines with hydroxylamine hydrochloride and hydrazine, mediated by *N*-bromosuccinimide (NBS) and phenyliodine(iii) diacetate (PIDA), respectively. NBS mediates the benzyl alcohol oxidation, followed by coupling with hydroxylamine hydrochloride, as well as hydrazines, leading to the formation of oximes and hydrazones, respectively. Additionally, PIDA oxidizes benzylamine to the corresponding intermediate imine, which then reacts with hydroxylamine hydrochloride and hydrazine to form oximes and hydrazones, respectively. We have also demonstrated the effectiveness of this methodology through a gram-scale reaction and synthetic transformation.

## Abstract 2

A one-pot synthesis of oximes and hydrazones *via* oxidative coupling of alcohols and amines with hydroxylamine hydrochloride and hydrazine, mediated by *N*-bromosuccinimide (NBS) and, additionally, phenyliodine(iii) diacetate (PIDA).

## Graphical abstract

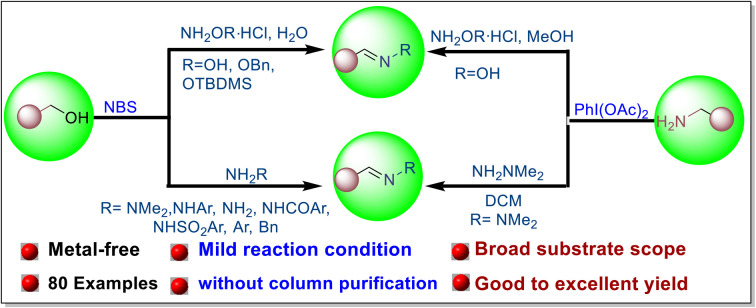

## Introduction

**Fig. 1:**
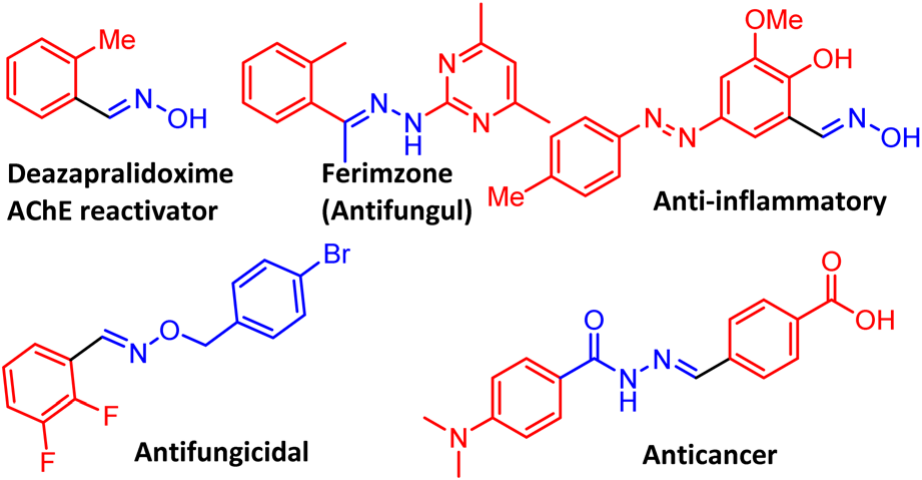
Representative natural products containing oximes and hydrazones motifs, drugs, and bioactive compounds.

**Scheme 1:**
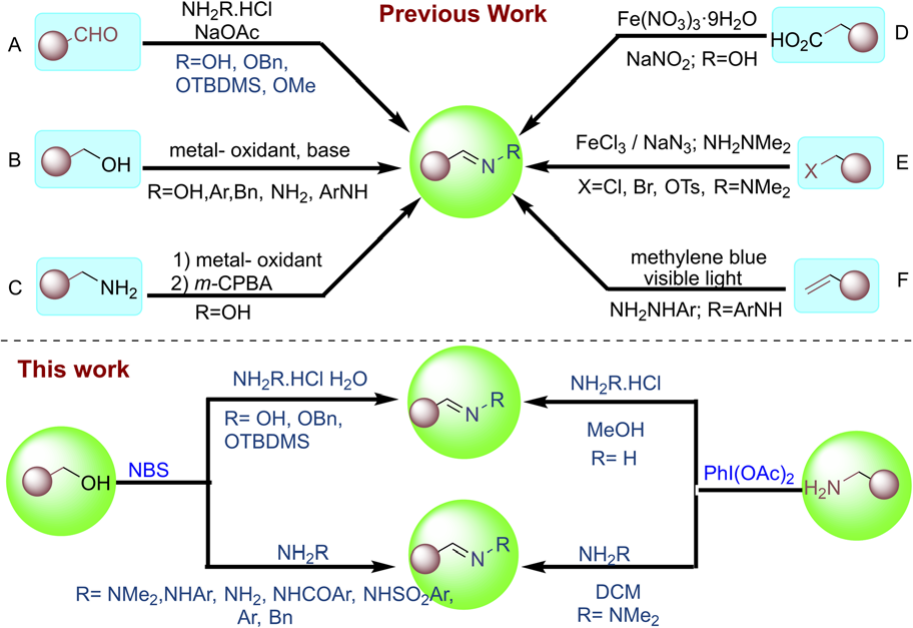
Strategies for the synthesis of oximes, hydrazones, and imines.

Oximes, hydrazones, and imines find wide application in the syntheses of key commodity chemicals, pharmaceuticals, polymers, heterocycles, and fine chemicals. Derivatives of these compounds exhibit biological properties with a wide range of applications, including antifungal, antibacterial, antimicrobial, antiviral, anticonvulsant, antimalarial, antiproliferative, anticancer, enzyme inhibitory, antioxidant, and antidepressant (Fig. 1).^1–4,11^ As ligands for mono- and polynuclear metal complexes, oximes and hydrazones play a crucial role in coordination chemistry, creating useful transition metal complexes for catalysis.^3,4^ Traditionally, oximes, hydrazones, and imines are synthesized by reacting carbonyl compounds with hydroxylamine hydrochloride, hydrazine, and amines (Scheme 1A).^5^ Furthermore, metal-catalysed oxidative couplings of benzyl alcohol with hydroxylamine hydrochloride, hydrazine, and amines have also been well explored. Au nanoparticles supported on hydroxyapatite (Au/HAP), Mn and Ru pincer complexes, Cu–Zn bimetallic catalysts, Ir and other metal complexes have been reported for tandem synthesis of oximes, hydrazones, and imines from alcohols (Scheme 1B).^6^ An alternative methodology for synthesizing oximes is *via* amine oxidation.

To achieve this, oxidants such as H_2_O_2_, TBHP, along with metallic systems such as nano-TiO_2_, Mo catalysts, titanium silicalite (TS-1), and chromium silicate are reported in prior art.^7^ The Shankarling group reports *m*-CPBA-mediated oxidation of aliphatic amines to oximes (Scheme 1C).^8^ Other miscellaneous synthetic routes to oximes and hydrazones include decarboxylation of alkyl carboxylic acids with NaNO_2_,^9*a*^ formation of *N*,*N*-dimethylhydrazones from alkyl halides and NaN_3_ using FeCl_3_,^9*b*^ and synthesis of hydrazones from styrene and hydrazine using visible light^9*c*^ (Scheme 1D–F).

However, these methods have several crucial drawbacks, such as harsh reaction conditions, use of toxic metals, low yields, and limited substrate diversity, that challenge the efficient synthesis of the desired oximes, hydrazones, and imines. Therefore, exploring more effective alternative synthetic routes to oximes, hydrazones, and imines would be beneficial. As part of our research on developing mild and environmentally friendly protocols for oxidation reactions, we previously described an oxidative olefination method using active methylene and Wittig reagents.^10^ Herein, we describe a one-pot oxidative coupling methodology using NBS/PIDA to efficiently transform alcohols and amines to oximes, hydrazones, and imines at benign reaction conditions, *via* reaction with hydroxylamine hydrochloride, hydrazine, and amines, respectively (Scheme 1).

## Results and discussion

The reaction optimization started after studying the model reaction between benzyl alcohol 1a (1 equiv.) and NBS (1.0 equiv.). The oxidant *N*-bromo succinamide (NBS) was added under stirring to benzyl alcohol 1a in methanol, and aldehyde formation was observed in 1 h (*T*_1_) reaction time, which was monitored by TLC. To this reaction mixture, hydroxylamine hydrochloride 4 (1.1 equiv.) and sodium acetate (1.1 equiv.) were added. The desired oxime 5a product was obtained in 56% yield in 3 h (*T*_2_) reaction time (Table 1; entry 1). The use of stoichiometric amounts of base is required to neutralize the acid in the hydroxylamine hydrochloride salt to release the free hydroxylamine for the condensation reaction.

**Table 1:** Optimization of reaction conditions for the synthesis of oxime

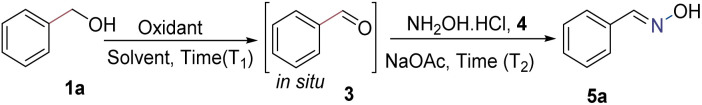				
Entry	Oxidant (equiv.)	Solvent	Time (*T*_1_) (h)	Yield^a^ (%)
1	NBS (1.1)	MeOH	1	56
2	NBS (1.1)	CH_3_CN	1	31
3	NBS (1.1)	DCM	1	37
4	NBS (1.1)	DMSO	1	29
5	NBS (1.1)	EtOH	1	52
6	NBS (1.1)	IPA	1	31
7	NBS (1.1)	*t*-BuOH	1	25
8	NBS (1.1)	*n*-BuOH	1	27
9	NBS (1.1)	H_2_O	1	61
10	NBS (1.1)	H_2_O	0.5	61
11^b^^,^^c^	**NBS** (1.2)	**H**_**2**_ **O**	0.5	**81**
12	NBS (1.2)	H_2_O	0.5	60
13	NBS (1.2)	H_2_O	0.5	56^d^/40^e^
14	—	H_2_O	1	NR
15^f^	NBS (1.2)	H_2_O	0.5	NR
16	NIS (1.2)	H_2_O	0.5	NR
17	NCS (1.2)	H_2_O	0.5	NR
18	PIDA (1.2)	H_2_O	0.5	NR
19	IBX (1.2)	H_2_O	0.5	Trace
20	DMP (1.2)	H_2_O	0.5	Trace

a Isolated yield.

b NH_2_OH·HCl (1.3 equiv.).

c NaOAc (1.3 equiv.).

d Solvent (1 M).

e Solvent (0.1 M).

f Without NaOAc, NR = no reaction.

The results indicate that the desired oxime product could be successfully synthesized using methanol as solvent and NBS as oxidant by the above methodology. Use of various solvents such as acetonitrile (CH_3_CN), DCM, dimethyl sulfoxide (DMSO), ethanol, isopropyl alcohol (IPA), *tert*-BuOH, *n*-BuOH, water, *etc.*, gave satisfactory product yields in the presence of NBS as oxidant (entries 2–9). Water turned out to be the best solvent, give 61% yield with NBS as oxidant (entry 9). Notably, in the presence of water as solvent and NBS as oxidant, even upon lowering of reaction time from 1 h to 0.5 h, no change in yield was observed (entry 10). Further, at optimized amounts of oxidant (1.2 equiv.), salt (1.3 equiv.), and sodium acetate (1.3 equiv.), notably, product yield increased to 81% (entry 11). Further, an increase in the concentration of the oxidant does not result in an improvement in the yield was observed. No reaction occurred in the absence of oxidant (entry 14) and base (entry 15), indicating that both oxidant and base are essential for transformation. However, in the absence of NBS oxidant and water as solvent, satisfactory oxime yields were not achieved, in spite of using other oxidants, *viz.* NIS, NCS, PIDA, IBX, and DMP (entries 16–20), the oxidation rate of alcohol is slower as compared to NBS, which may be due to chemoselectivity. In contrast, oxidizing benzyl alcohol using PIDA, IBX, and DMP involves harsher reaction conditions. As a result, the reaction does not occur within the desired time frame and parameters. Further, solvent concentration (0.1 and 1 M) slightly affects the yield of the product formation (entry 13).

**Scheme 2:**
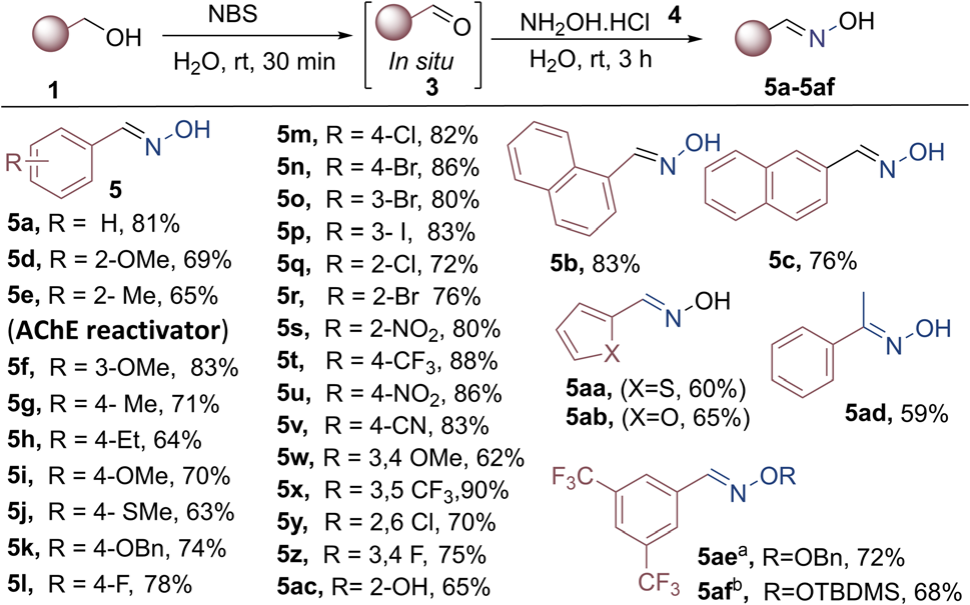
Substrate scope for the synthesis of oxime derivatives from benzyl alcohol. Reaction conditions: 1 (1 mmol, 1 equiv.), oxidant 2 (1.2 equiv.), 4a (1.3 equiv.), NaOAc, (1.3 equiv.), H_2_O solvent (0.5 M), time = rt 3 h, isolated yield, ^*a*^4b, *O*-benzylhydroxylamine hydrochloride, ^*b*^4c, *O*-(*tert*-butyldimethylsilyl) hydroxylamine.

With optimized reaction conditions in hand, we investigated the substrate scope for the oxidative coupling reaction. Various substituted benzyl alcohols (1a–1af) were well-tolerated under optimised reaction conditions (5a–5af) in good yields. The results have been summarised in Scheme 2. Benzyl alcohol bearing electron-donating groups such as Me (AChE reactivator 5e), Et, OMe, OBn, and SMe at *ortho*, *meta*, and *para* positions exhibited moderate to high yield (5d–5k; 63–83%). Halogenated benzyl alcohols (F, Cl, and Br) delivered the desired products (5l–r) in good yield (72–86%). The electron-withdrawing group bearing benzyl alcohols (NO_2_, CN, and CF_3_) gave corresponding oximes (5s–5v) in excellent yields (80–88%). Disubstituted benzyl alcohol 3,4-dimethoxy, 3,5-di(trifluoromethyl), 2,6-dichloro, and 3,5-difluoro also delivered the corresponding product (5w–z) in good yield (62–90%). 1- and 2-Naphthalenemethanol (1b–1c) successfully generated the corresponding oximes (5b–5c) with notable yields of 83% and 76%, respectively. It should be noted that heterocyclic thiophen-2-yl-methanol and furan-2-yl-methanol yielded satisfactory results, producing the desired products (5aa–ab) with 60% and 65% yields, respectively. 2-Hydroxybenzylalcohol and 1-phenylethanol also furnished the oxime products in 65% and 59% yield, respectively. Next, the scope of the reaction was studied with different hydroxylamines, *viz.*, *o*-benzyl hydroxylamine hydrochloride, and *o*-(*tert*-butyldimethylsilyl) hydroxylamine, which were compatible to afford products 5ae–aaf, in 68–72% yields.

**Scheme 3:**
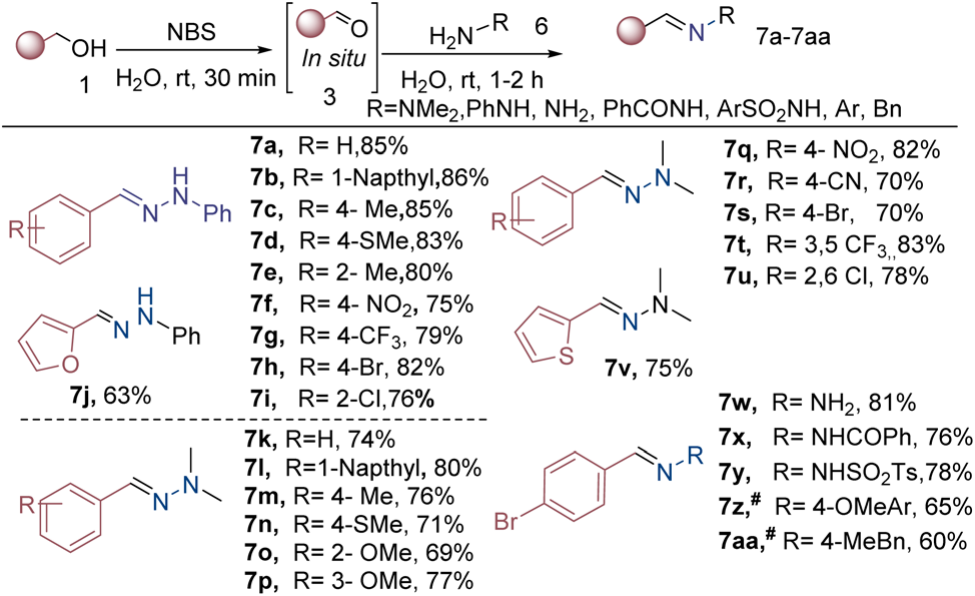
Substrate scope for the synthesis of hydrazones and imines from benzylalcohols. Reaction conditions: 1 (1 mmol, 1 equiv.), oxidant 2 (1.2 equiv.), 6 (1.3 equiv.), H_2_O solvent (0.5 M), rt 1–3 h, isolated yield. ^#^CH_2_Cl_2_, ^#^MgSO_4_ (2 equiv.).

The optimized reaction condition was further extended to the synthesis of hydrazones and imines without the base shown in Scheme 3. The results demonstrate that benzyl and naphthyl alcohol (1a–b) achieve yields of the corresponding products (7a–b) in 85% and 86%, respectively. Substituted benzyl alcohols featuring electron-donating SMe groups at *ortho* and *para* positions demonstrated a favourable outcome, with excellent yield (7c–7e) ranging from 80% to 85%.

Similarly, the presence of electron-withdrawing and halogen groups, such as NO_2_, CF_3_, Br, and Cl at *ortho*- and *para*-positions, has led to hydrazone products with yields in 75% and 82% (7f–7i), respectively (Scheme 3). Furthermore, heterocyclic furan-2-yl-methanol 1j showed promising performance under the reaction conditions, yielding hydrazone product 7j with a yield of 63%.

Further, we replaced phenylhydrazine with *N*,*N*-dimethyl-hydrazine, which reacts with benzyl and 1-naphthyl alcohols to obtain their corresponding hydrazone products, in 74–80% yield (7k–7l). Substituted benzyl alcohols (with various electron-donating and withdrawing substituents, including Me, SMe, OMe, NO_2_, CN, and Br) were found to be compatible in reactivity. They yielded the desired products (7m–7s) in good yields (69–82%). Additionally, disubstituted benzylalcohol and heterocyclic benzylalcohol also participated in hydrazone formation, with products (7t–7v) yields of 75–83%.

In subsequent experiments, we examined how different hydrazines react under the optimised reaction conditions. The hydrazine hydrochloride reacted efficiently with 4-bromobenzyl alcohol 1w, producing (*E*)-(4-bromobenzylidene) hydrazine 7w with an impressive 81% yield. Additionally, benzhydrazide and tosyl hydrazide were compatible, yielding products (7x–7y) with high yields (76–78%). Reaction of *p*-anisidine and 4-methylbenzylamine under optimized reaction conditions did not give satisfactory results, therefore, the reaction was performed in DCM solvent with 2 equivalents of MgSO_4_, which led to encouraging results (65% and 60% yields, respectively).

Based on our successful methodology for the synthesis of various oxime and hydrazine derivatives, we shifted our focus to benzyl amines as key partners with hydroxylamine hydrochloride (4a) and *N*,*N*-dimethyl-hydrazine (6b). Using our optimized reaction conditions with benzylamine 8a and hydroxylamine hydrochloride, satisfactory results were not obtained (Table 2, entry 1). Replacing the oxidant with NCS or PIDA in H_2_O also did not yield the desired product. Then we screened various solvents, *viz.* DCM, DCE, CH_3_CN, DMSO, DMF, *n*-BuOH, IPA, EtOH, and MeOH (entries 3–12). Oxidant PIDA gave the highest yield in methanol (58%, entry 12). The polar protic solvents gave superior yields than polar aprotic solvents. Extending the reaction time to 12 h did not improve the yield (entry 13). The yield was enhanced by up to 71% by increasing the concentration of PIDA from 1.1 to 1.3 equivalents (entry 14). In the absence of an oxidant, no reaction was observed, thereby confirming its essential role (entry 15).

**Table 2:** Optimisation of reaction conditions for the synthesis of oxime from benzylamine

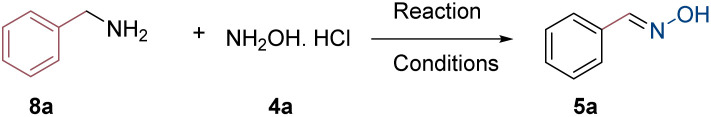				
Entry	Oxidant	Solvent	Time (h)	Yield^a^ (%)
1	NBS	H_2_O	5	NR^c^
2	NCS	H_2_O	5	NR
3	PhI(OAc)_2_	H_2_O	5	NR
4	PhI(OAc)_2_	DCM	5	31
5	PhI(OAc)_2_	DCE	5	26
6	PhI(OAc)_2_	CH_3_CN	5	38
7	PhI(OAc)_2_	DMSO	5	17
8	PhI(OAc)_2_	DMF	5	23
9	PhI(OAc)_2_	EtOH	5	52
10	PhI(OAc)_2_	*n*-BuOH	5	31
11	PhI(OAc)_2_	IPA	5	45
12	PhI(OAc)_2_	MeOH	5	58
13	PhI(OAc)_2_	MeOH	12	64
**14** b	**PhI(OAc)**_**2**_	**MeOH**	**5**	**71**
15	—	MeOH	5	NR

a Isolated yield.

b 4a (1.3 mmol, 1.3 equiv.).

c NR = no reaction.

**Scheme 4:**
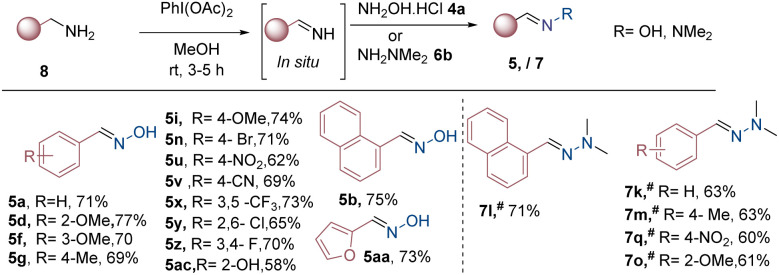
Substrate scope for the synthesis of oximes and hydrazones from benzylamines. Reaction conditions: 8 (1 mmol 1 equiv.), 4/6 (1.1 mmol, 1.1 equiv.), PIDA (1.3 mmol) in MeOH/^#^DCM (5 mL), at room temperature for 5 h, isolated yield.

At optimized reaction conditions (Table 2, entry 14), we explored substrate scope for oxidative coupling reactions. Various substituted benzylamines 8 were well tolerated under these optimized reaction conditions, leading to good yields of 5 (Scheme 4). 2-Naphthalene methanamine 8a generated the corresponding oxime products 5b at a yield of 75%. Benzylamines with electron-donating groups such as (Me, OMe) at *ortho*, *meta*, and *para* positions exhibited good yields of the corresponding oximes (5d, 5f, 5g, 5i; 69–77%). However, halo, NO_2,_ and CN, at *para* positions, gave moderate amounts of oximes (5n, 5u, 5v) in 62–69% yields. Disubstituted benzylamine 3,5-di(trifluoromethyl), 2,6-dichloro, and 3,5-difluoro also delivered the expected products (5x–z, 65–73%). Notably, heterocyclic compounds, furan-2-yl-methanamine, and *o*-hydroxy benzylamine satisfactorily yielded the desired products 5aa and 5ac at 58% and 73% yields, respectively (Scheme 4).

Further, we changed the coupling partner from hydroxylamine hydrochloride to *N*,*N*-dimethylhydrazine with our optimized reaction conditions, but we didn't get a satisfactory result. Therefore, we changed the solvent from MeOH to DCM, and we observed good results, which are summarized in (Scheme 4). Further, we screen various benzylamines, including benzylamine, naphthalen-1-ylmethanamine, and electron-donating and electron-withdrawing groups, such as Me, OMe, and NO_2_, which yield the corresponding hydrazone products with yields between 58 and 71% (7K, 7l, 7m, 7q, and 7o).

**Scheme 5:**
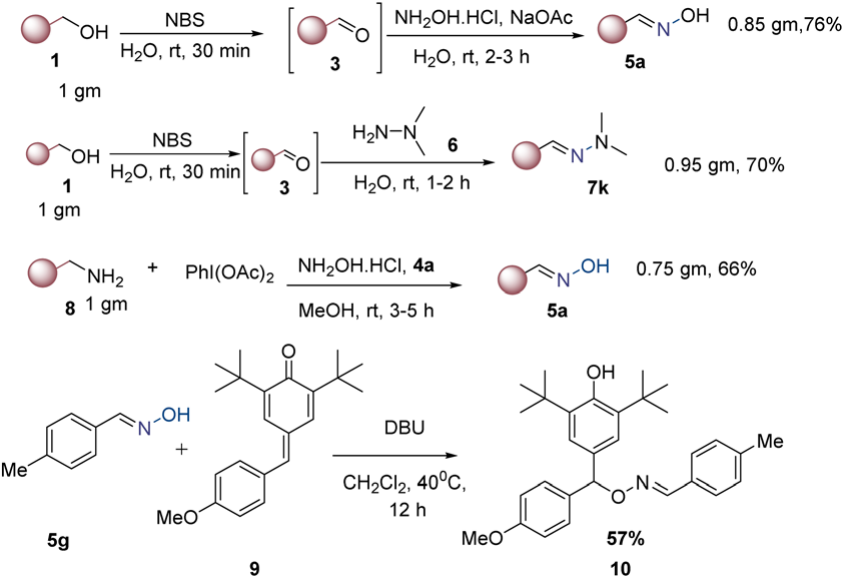
Gram scale and synthetic transformations.

To demonstrate the utility of the current method, a gram-scale reaction was performed under optimal reaction conditions. As a result, products 5a, 7k, and 5a were obtained in 0.85 g, 0.95 g, and 0.75 g with yields of 76%, 70%, and 66%, respectively. To showcase the synthetic potential of our methodology, we conducted a synthetic transformation on 5g using *para*-quinone methides 9. This reaction, carried out in the presence of DBU in DCM solvent at 40 °C for 12 hours, gave the desired product 10 in 57% yield (see Scheme 5).

**Scheme 6:**
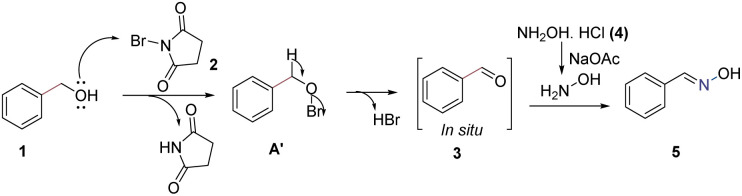
Plausible reaction mechanism for benzyl alcohol.

Based on literature reports,^11^ a plausible reaction pathway for benzyl alcohol has been proposed (Scheme 6). Initially, NBS reacts with benzyl alcohol 1 to form a hypobromite intermediate A′; followed by the removal of HBr, which leads to the formation of *in situ* benzaldehyde 3. Additionally, molecular bromine is generated from the reaction between NBS and HBr. Subsequently, sodium acetate abstracts protons from hydroxylamine hydrochloride and forms salt-free hydroxylamine. Finally, benzaldehyde reacts with this hydroxylamine to produce oxime derivatives 5 (see Scheme 6). The reaction of 1g, was carried out in deuterated water, the hydrogen of hydroxylamine of 5g was found to be exchanged with deuterium about 25%, which supports a plausible reaction mechanism.

**Scheme 7:**
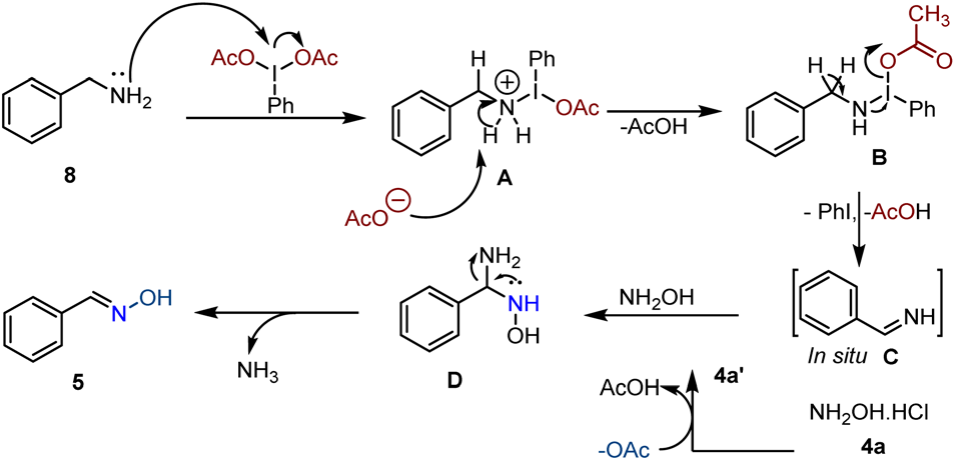
Plausible reaction mechanism for benzylamines.

Plausible reaction mechanism for synthesizing oximes from benzylamine through oxidative coupling is proposed in Scheme 7.^10^ Initially, benzylamine 8 reacts with PIDA to form an intermediate A, which goes through deprotonation, converts into B, releasing acetic acid as a side product. Then, intermediate B undergoes intramolecular deprotonation, producing a reactive imine intermediate C along with iodobenzene and acetic acid. The acetate anion generated *in situ* acts as a base, allowing for the formation of salt-free hydroxylamine hydrochloride 4a′. 4a′ reacts with imine C to produce intermediate D, leading to the final removal of ammonia and the formation of oxime 5, as illustrated in Scheme 7.

## Conclusions

In summary, we have successfully developed a one-pot protocol for the oxidative coupling of benzyl alcohol and benzylamines driven by NBS and hypervalent iodine reagents, resulting in the efficient synthesis of oximes, hydrazones, and imines. NBS-mediated oxidation of benzyl alcohol transforms it into an aldehyde, which couples with hydroxylamine hydrochloride, hydrazines, and amines to generate benzaldoximes, hydrazones, and imines in excellent yields and high *E* selectivity. PIDA mediates the oxidation of benzylamines to imines, which readily couple with hydroxylamine hydrochloride and hydrazine, to produce corresponding oximes and hydrazones. Furthermore, we have also demonstrated the efficiency of this methodology on a gram scale, highlighting its substantial synthetic utility.

## Conflicts of interest

There are no conflicts to declare.

## Supplementary Material

RA-016-D6RA02204A-s001

## Data Availability

The data supporting this article have been included as part of the supplementary information (SI). Supplementary information: experimental details, characterization and analytical data. See DOI: https://doi.org/10.1039/d6ra02204a.

## References

[cit1] (a) Robertoson G. M. , in Comprehensive Functional Group Transformation, ed. A. R. Katritzky, O. Meth-Cohn and C. W. Rees, Elsevier, Oxford, 1995, vol. 3, p. 425

[cit2] Bolotin D. S., Bokach N. A., Demakova M. Y., Kukushkin V. Y. (2017). Chem. Rev..

[cit3] Zhou X. T., Yuan Q.-L., Ji H.-B. (2010). Tetrahedron Lett..

[cit4] Humphrey G. R., Kuethe J. T. (2006). Chem. Rev..

[cit5] Pohjakallio A., Pihko P. M. (2009). Chem.–Eur. J..

[cit6] Sun H., Su Z. F., Ni J., Cao Y., He Y. H., Fan N. K. (2009). Angew. Chem., Int. Ed..

[cit7] Kidwai M., Bhardwaj S. (2011). Synth. Commun..

[cit8] Patil V. V., Gayakwad E. M., Shankarling G. S. (2016). J. Org. Chem..

[cit9] Yang S., Wang Y., Xu W., Tian X., Bao M., Yu X. (2023). Org. Lett..

[cit10] Rupanawar B. D., Veetil S. M., Suryavanshi G. (2019). Eur. J. Org Chem..

[cit11] Muneeswara M., Sundaravelu N., Sekar G. (2019). Tetrahedron.

