# Supplementary material for: Metal-free sustainable synthesis of oximes and hydrazones from benzyl alcohol and benzylamine
Source: RSC Adv. 2026 May 26;16(31):28482–7. doi: 10.1039/d6ra02204a (PMC13213572; doi:10.1039/d6ra02204a)
Supplement: RA-016-D6RA02204A-s001 [file RA-016-D6RA02204A-s001.pdf]

Supplementary Information (SI) for RSC Advances.
This journal is © The Royal Society of Chemistry 2026

Electronic Supplementary Information (ESI)

Metal-Free Sustainable Synthesis of Oximes and Hydrazones from Benzyl Alcohol and Benzylamine

Rupali Devgunde,[a] Bapurao Rupanawar,[b] Prasad Rupnavar,[a] and Suresh B. Waghmode*[a]

[a]Department of Chemistry, Savitribai Phule Pune University (Formerly University of Pune), Pune-411007, India

[b]Chemical Engineering & Process Development Division, CSIR-National Chemical Laboratory, Pune-411008, India

E-mail: suresh.waghmode@unipune.ac.in and suresh.waghmode@gmail.com

**Table of Contents**		
**Sr. No.**	**Description**	**Page No.**
1	General Information	S3
2	General Experimental Procedure for the Synthesis of Compound (**5a-5af**) from Benzyl alcohol	S4
3	General Experimental Procedure for Gram Scale Synthesis of Compound (**5a**) from Benzyl alcohol	S4
4	General Experimental Procedure for the Synthesis of Compounds (**7a-7y**) from Benzyl alcohol	S5
5	General Experimental Procedure for Gram-Scale Synthesis of Compound (**7k**) from Benzyl alcohol	S5
6	General Experimental Procedure for the Synthesis of Compounds (**7z** and **7aa**) from Benzyl alcohol	S5
7	General Experimental Procedure for the Synthesis of Compound (**5a-5ac**) from Benzylamines	S6
8	General Experimental Procedure for Gram-Scale Synthesis of Compound (**5a**) from Benzylamine	S7
9	General Experimental Procedure for the Synthesis of Compound (**7**)	S7
10	General Experimental Procedure for the Synthesis of Compound (**10**)	S7
11	General Experimental Procedure for the Synthesis of Deuterated Compound (**5g'**) from Benzyl alcohol	S8
12	Spectral data	S9
13	References	S26
14	^{1}H, ^{13}C and ^{19}F NMR spectra	S28

General Information

Unless otherwise noted, all commercially available components, reagents, and solvents were obtained from suppliers and used without further purification. Analytical thin-layer chromatography (TLC) was performed using a Merck silica gel 60 F254 precoated plate (0.2 mm thickness). TLC plates were visualized under UV light (254 nm). Further visualization was possible by staining with a basic solution of potassium permanganate or an acidic aqueous solution of ceric molybdate. Column chromatography was carried out on silica gel (100-200 mesh) or neutral silica using EtOAc/*n*-hexane as an eluent. ^{1}H NMR (500 MHz) and ^{13}C NMR (126MHz) spectra were measured on Bruker-500 spectrometers with $CDCl_3$, DMSO-D_6, or MeOD-D_4 as solvents and tetramethylsilane (TMS) as internal standard. Chemical shifts were reported in units (ppm) by assigning the TMS resonance in the ^{1}H spectrum as 0.00 ppm. The peaks at 7.27 ppm, 2.50 ppm, and 3.31 ppm in ^{1}H NMR and 77.00 ppm, 39.50 ppm, and 49.00 ppm in ^{13}C NMR correspond to $CDCl_3$, DMSO-D_6, and MeOD-D_4, respectively. All coupling constants (J values) were reported in Hertz (Hz). Multiplicities were given as: s (singlet); d (doublet); t (triplet); q (quartet); dd (doublets of doublet); ddd (doublets of doublets of doublet); dt (doublets of triplet); or m (multiplets), coupling constants (Hz), and integration. Chemical shifts of common trace ^{1}H NMR impurities (ppm): H_2O in $CDCl_3$: 1.56 ppm, H_2O in DMSO-D_6: 3.33 ppm, H_2O in MeOD-D_4: 4.87 ppm. HRMS (ESI) spectra were recorded using a Bruker Impact HD quadrupole plus ion trap at the CIF, S. P. Pune University. Melting points were determined with a Büchi B-540 capillary melting point apparatus in open capillaries and were uncorrected.

General Experimental Procedure for the Synthesis of Compound (5a-5af) from Benzyl alcohol

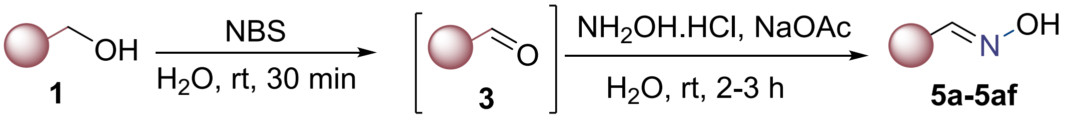

An oven-dried 50 mL reaction tube containing benzyl alcohol **1** (1 mmol, 1 equiv.) in H_2O was charged with NBS (1.2 mmol, 1.2 equiv.) and stirred under air at room temperature for 30-60 min, during which the evolution of a brown gas (Br_2) was observed, and the reaction mixture gradually turned reddish-brown. Subsequently, hydroxylamine hydrochloride (1.3 mmol, 1.3 equiv.) and NaOAc (1.3 mmol, 1.3 equiv.) were added portion-wise, and the reaction mixture was stirred for an additional 2–3 h. After completion of the reaction, as monitored by TLC, CH_2Cl_2 (10 mL) and brine (10 mL) were added, and the mixture was extracted with CH_2Cl_2 (2 × 10 mL). The combined organic layers were dried over anhydrous Na_2SO_4, filtered, and concentrated under reduced pressure to afford the desired product in pure form without further purification.

General Experimental Procedure for Gram Scale Synthesis of Compound (5a) from Benzyl alcohol

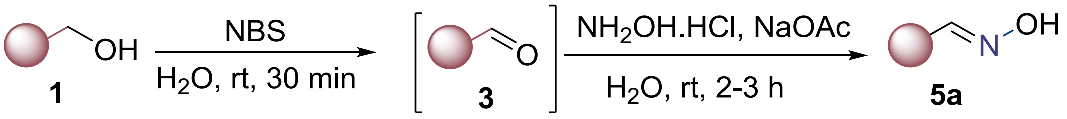

An oven-dried 100 mL reaction tube containing benzyl alcohol **1** (1gm, 9.25 mmol) in 19 mL H_2O was charged with NBS (1.98gm, 11.10 mmol) and stirred under air at room temperature for 30-60 min, during which the evolution of a brown gas (Br_2) was observed, and the reaction mixture gradually turned reddish-brown. Subsequently, hydroxylamine hydrochloride (0.835gm, 12.02 mmol) and NaOAc (0.986 gm 12.02 mmol) were added portion-wise, and the reaction mixture was stirred for an additional 2-3 h. After completion of the reaction, as monitored by TLC, CH_2Cl_2 (50 mL) and brine (50 mL) were added, and the mixture was extracted with CH_2Cl_2 (2 × 50 mL). The combined organic layers were dried over anhydrous Na_2SO_4, filtered, and concentrated under reduced pressure to afford the 850 mg, 76% yield of the desired product in pure form without further purification.

General Experimental Procedure for the Synthesis of Compounds (7a-7y) from Benzyl alcohol

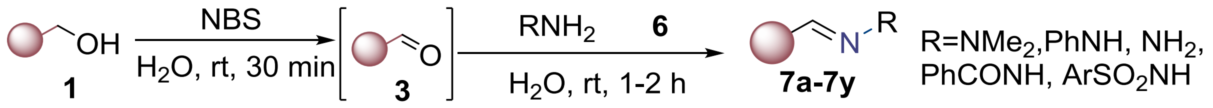

An oven-dried 50 mL reaction tube containing benzyl alcohol **1** (1 mmol, 1 equiv.) in H_2O was charged with NBS (1.2 mmol, 1.2 equiv.) under an open atmosphere and stirred for 30-60 min at room temperature, the generation of brown colour gas (bromine), which resulted in a reddish-brown colour mixture, then dropwise added hydrazine (**6**) (1.3 mmol, 1.3 equiv.) and stirring for 1-2 h. TLC monitored the progress of the reaction. Upon the completion of the reaction, the mixture was filtered through filter paper and washed with water and brine (5x10 mL). The solid residue dried to afford the desired product, which does not need further purification.

General Experimental Procedure for Gram-Scale Synthesis of Compound (7k) from Benzyl alcohol

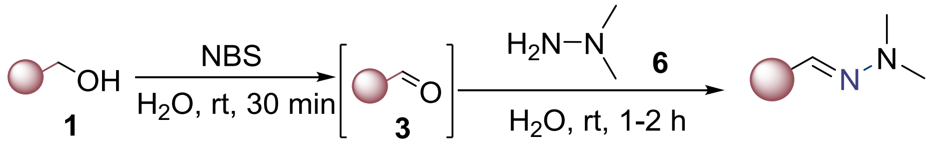

7k

An oven-dried 100 mL reaction tube containing benzyl alcohol **1** (1 gm, 9.25 mmol) in 19 mL H_2O was charged with NBS (1.98 gm, 11.1 mmol) under an open atmosphere and stirred for 30-60 min at room temperature, the generation of brown colour gas (bromine), which resulted in a reddish-brown colour mixture, then dropwise added 1,1-Dimethylhydrazine, (12.02 mmol,) and stirring for 1-2 h. TLC monitored the progress of the reaction. Upon the completion of the reaction, the mixture was filtered through filter paper and washed with water and brine (5x 50 mL). The solid residue dried to afford 0.955 gm, 70% yield of the desired product, which does not need further purification.

General Experimental Procedure for the Synthesis of Compound (7z and 7aa) from Benzyl alcohol

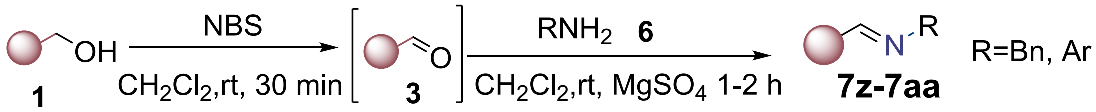

An oven-dried 50 mL reaction tube was charged with benzyl alcohol 1 (1.0 mmol, 1.0 equiv) dissolved in CH_2Cl_2, followed by the addition of *N*-bromosuccinimide (NBS; 1.2 mmol, 1.2 equiv). The reaction mixture was stirred under air at room temperature for 30–60 min, during which the evolution of a brown gas was observed, and the mixture gradually turned reddish-brown. Subsequently, the amine nucleophile (1.0 mmol, 1.0 equiv) was added, then $MgSO_4$ (2.0 equiv.) was added, and the reaction was stirred for an additional 6 h. TLC monitored the progress of the reaction. Upon complete consumption of the reaction, the solvent was removed under reduced pressure, and there was no need to do an aqueous workup of the reaction. The resulting residue was purified by column chromatography on silica gel (100–200 mesh) using petroleum ether/ethyl acetate (5–20%) along with a small amount of triethylamine as the eluent to afford the desired product.

General Experimental Procedure for the Synthesis of Compound (5a-5ac) from Benzylamines

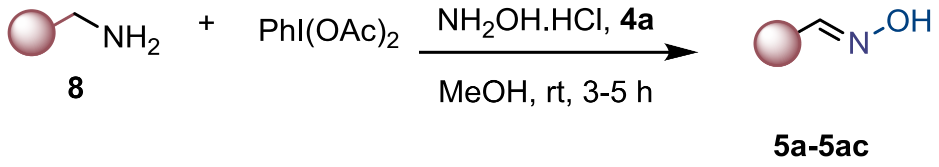

In a oven-dried 25 mL round-bottom flask, the benzyl amine (1.0 mmol) was dissolved in 5 mL of dehydrated MeOH under an argon atmosphere, $PhI(OAc)_2$ (1.3 mmol) was added in a single lot, followed by hydroxyl amine hydrochloride salt (1.3 mmol) at room temperature, the resulting mixture was stirred at room temperature for 3-5 h. After the completion of the reaction (monitored by TLC), the reaction mixture was concentrated under reduced pressure to remove methanol. The product was quenched with a saturated solution of $NaHCO_3$ (10 mL), and extracted with CH_2Cl_2 (3 × 10 mL). The combined organic layers were washed with brine and dried over Na_2SO_4 and concentrated under reduced pressure. The residue was purified by column chromatography on silica gel (100-200 mesh) and 5-20% ethyl acetate in petroleum ether as an eluting solvent to afford the desired product.

General Experimental Procedure for Gram-Scale Synthesis of Compound (5a) from Benzyl amine

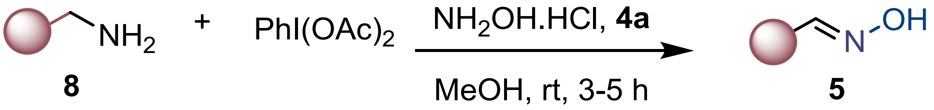

In a oven-dried 100 mL round-bottom flask, the benzyl amine (1 g, 9.33 mmol) was dissolved in 50 mL of dehydrated MeOH under an argon atmosphere, $PhI(OAc)_2$ (3.91 g, 12.13 mmol) was added in a single lot, followed by hydroxyl amine hydrochloride salt (0.843 g, 12.13 mmol) at room temperature, the resulting mixture was stirred at room temperature for 3-5 h. After the completion of the reaction (monitored by TLC), the reaction mixture was concentrated under reduced pressure to remove methanol. The product was quenched with a saturated solution of $NaHCO_3$ (50 mL), and extracted with CH_2Cl_2 (3 × 50 mL). The combined organic layers were washed with brine and dried over Na_2SO_4 and concentrated under reduced pressure. The residue was purified by column chromatography on silica gel (100-200 mesh) and 5-20% ethyl acetate in petroleum ether as an eluting solvent to afford the desired product in 0.751g with 66% yield.

General Experimental Procedure for the Synthesis of Compound (7) from Benzyl amine

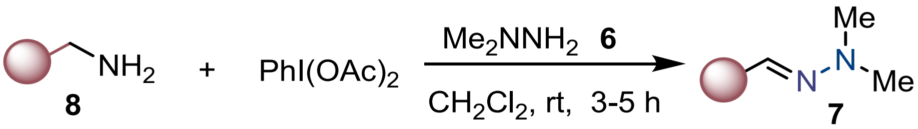

In a round-bottom 25 mL flask, the benzyl amines (1.0 mmol) were dissolved in 5 mL of CH_2Cl_2 to stirred the reaction mixture $PhI(OAc)_2$ (1.3 mmol) and 1,1-Dimethylhydrazine (1.3 mmol) was slowly added in one at the same instant. The reaction mixture was stirred at room temperature for the indicated time, until full conversion of the benzyl amines was observed *via* TLC. After completion, add saturated solution of $NaHCO_3$ (10 mL) and extract with CH_2Cl_2 (10 mL). The organic layers were combined, washed with brine, and dried over Na_2SO_4 and concentrated under reduced pressure. The residue was purified by (Neutral silica gel) column chromatography with 5-20% ethyl acetate in petroleum ether along with a small amount of triethylamine as an eluting solvent, to afford the desired product.

General Experimental Procedure for the Synthesis of Compound (10)

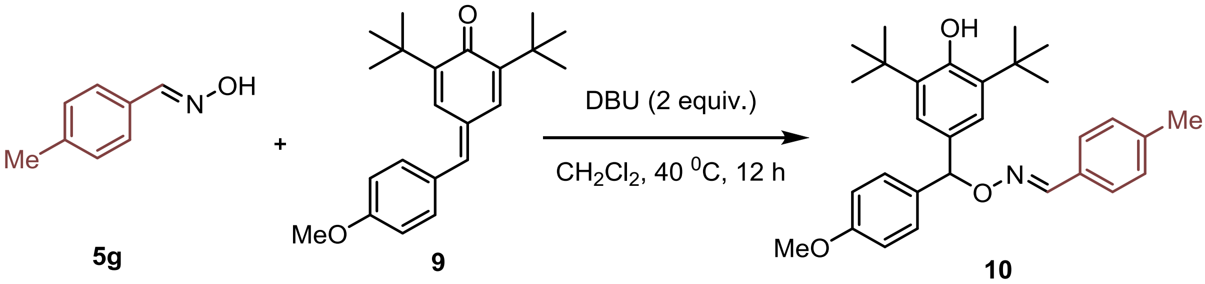

To a 25 mL round-bottom 25 mL flask with stir bar were added *p*-QMs (8) (32.4 mg, 0.1 mmol) and oxime 4g (20.2 mg, 0.15 mmol) in dry CH_2 Cl_2(1 mL), and DBU (30.6 mg, 0.2 mmol,) was added in the reaction mixture. The resultant reaction mixture was stirred at 40 ^{0}C for 12 h. After completion (monitored by TLC), the reaction mixture was quenched with water (10 mL) and extracted with CH_2Cl_2 (3 x 10 mL). The combined organic layer was washed with brine (10 mL), dried over Na_2SO_4, and evaporated in vacuo. The crude was subjected to purification on flash silica gel chromatography (petroleum ether/ethylacetate = 95:05) to afford desired product (**10**).

General Experimental Procedure for the Synthesis of Deuterated Compound (5g') from Benzyl alcohol

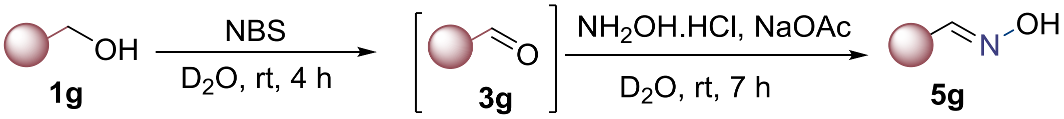

An oven-dried 50 mL reaction tube containing benzyl alcohol **1g** (1 mmol, 1 equiv.) in D_2O was charged with NBS (1.2 mmol, 1.2 equiv.) and stirred under air at room temperature for 4 h, during which the evolution of a brown gas (Br_2) was observed, and the reaction mixture gradually turned orange. Subsequently, hydroxylamine hydrochloride (1.3 mmol, 1.3 equiv.) and NaOAc (1.3 mmol, 1.3 equiv.) were added portion-wise, and the reaction mixture was stirred for an additional 7 h. After completion of the reaction, as monitored by TLC, the product precipitated out was filtered, desired product was obtained in pure.

Spectral Data

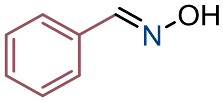

(*E*)-benzaldehyde oxime (5a)[1,2.3,4,5,6,7,8]

Yield: :81% (103 mg), [$BnNH_2$,71% 86 mg]; colorless liquid; **^{1}H NMR** (500 MHz, $CDCl_3$) δ: 8.18 (d, *J* = 1.7 Hz, 1H), 7.59 (ddd, *J* = 5.3, 2.5, 1.5 Hz, 2H), 7.43-7.35 (m, 3H).; **^{13}C {^{1}H} NMR** (126 MHz, $CDCl_3$) δ: 150.5, 132.0, 130.2, 130.2, 128.9, 127.1.; **HRMS** (ESI) m/z calcd. $[M+H]^+$ for C_7H_8O 122.0606; found 122.0617.

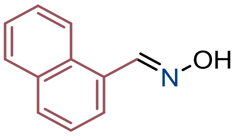

(*E*)-1-naphthaldehyde oxime (5b)[1,2,3,5,7,8]

Yield: 83% (103 mg), [$BnNH_2$,75% 128 mg]; White solid; **MP**: 96 ºC; **^{1}H NMR** (400 MHz, $CDCl_3$) δ: 8.81 (s, 1H), 8.54 – 8.43 (m, 1H), 7.90 (td, *J* = 5.3, 2.7 Hz, 3H), 7.78 (dd, *J* = 7.1, 1.2 Hz, 1H), 7.62 – 7.45 (m, 3H).; **^{13}C {^{1}H} NMR** (126 MHz, $CDCl_3$) δ: 150.3, 133.9, 130.8, 130.7, 128.90, 128.1, 127.3, 127.2, 126.3, 125.4, 124.4.; **HRMS** (ESI) m/z calcd. $[M+H]^+$ for $C_{11}H_{10}NO$ 172.0762; found 172.0765.

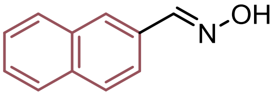

(*E*)-2-naphthaldehyde oxime ((5c)[3,7]

Yield: 76% (130mg); White solid; **MP**: 152 ºC; **^{1}H NMR** (500 MHz, $CDCl_3$) δ: 8.31 (s, 1H), 7.91-7.88 (m, 1H), 7.89-7.81 (m, 5H), 7.56-7.48 (m, 2H).; **^{13}C {^{1}H} NMR** (126 MHz, $CDCl_3$) δ: 150.7, 134.2, 133.2, 129.7, 128.8, 128.7, 128.4, 128.0, 127.1, 126.7, 122.8.; **HRMS** (ESI) m/z calcd. $[M+H]^+$ for $C_{11}H_{10}NO$ 172.0762; found 172.0761.

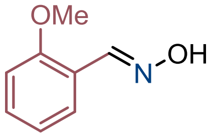

***(E)-2-methoxybenzaldehyde oxime* (5d)**[2,3,8]

Yield: 69% (104mg);[$BnNH_2$,77% 116 mg] White solid; **MP**: 88 ºC; **^{1}H NMR** (500 MHz, $CDCl_3$) δ: ^{1}H NMR (500 MHz, Chloroform-*d*) δ 8.76 (s, 1H), 8.48 (s, 1H), 7.66 (dd, *J* = 7.7,

1.8 Hz, 1H), 7.36 (ddd, J = 8.3, 7.4, 1.8 Hz, 1H), 7.09-6.82 (m, 2H), 3.87 (s, 3H).; **^{13}C {^{1}H} NMR** (126 MHz, $CDCl_3$) δ: 157.7, 146.9, 131.3, 127.4, 120.0, 120.7, 111.2, 55.6.; **HRMS** (ESI) m/z calcd. $[M+H]^+$ for $C_8H_{10}NO_2$ 152.0712; found 152.0723.

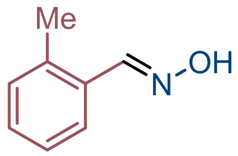

(*E*)-2-methyl benzaldehyde oxime (5e)[9]

Yield: 65% (90mg); White solid; **MP**: 54 ºC; **^{1}H NMR** (500 MHz, $CDCl_3$) δ: 9.21 (s, 1H), 8.50 (s, 1H), 7.73 (d, J = 7.7 Hz, 1H), 7.43-7.12 (m, 3H), 2.49 (s, 3H).; **^{13}C {^{1}H} NMR** (126 MHz, $CDCl_3$) δ:149.3, 136.9, 130.9, 130.2, 130.0, 126.7, 126.3, 19.8.; **HRMS** (ESI) m/z calcd. $[M+H]^+$ for $C_8H_{10}NO$ 136.0762; found 136.0767.

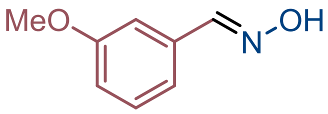

(*E*)-3-methoxybenzaldehyde oxime (5f)[2,3,8,9]

Yield: 83% (112mg););[BnNH$_2$,70% 106 mg], White solid; **MP**: 40 ºC; **^{1}H NMR** (500 MHz, $CDCl_3$) δ: 8.11 (s, 1H), 7.50 (s, 1H), 7.35-7.27 (m, 1H), 7.19-7.08 (m, 2H), 6.94 (ddd, J = 8.3, 2.7, 1.0 Hz, 1H), 3.83 (s, 3H).; **^{13}C {^{1}H} NMR** (126 MHz, $CDCl_3$) δ: 159.9, 150.5, 133.4, 129.9, 120.2, 116.5, 111.2, 55.4.; **HRMS** (ESI) m/z calcd. $[M+H]^+$ for $C_8H_{10}NO_2$ 152.0712; found 152.0720.

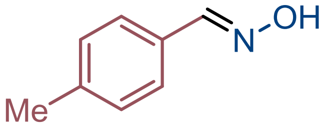

(*E*)-4-methylbenzaldehyde oxime (5g)[6,7]

Yield: 71% (96 mg);[BnNH$_2$,69% 93 mg] White solid; **MP**: 76 ºC; **^{1}H NMR** (500 MHz, $CDCl_3$) δ: 8.40 (d, J = 104.0 Hz, 1H), 8.14 (s, 1H), 7.48 (d, J = 8.2 Hz, 2H), 7.20 (d, J = 8.0 Hz, 2H), 2.38 (s, 3H).; **^{13}C {^{1}H} NMR** (126 MHz, $CDCl_3$) δ: 150.4, 140.4, 129.6, 129.2, 127.1, 21.6, 15.4.; **HRMS** (ESI) m/z calcd. $[M+H]^+$ for $C_8H_{10}NO$ 136.0762; found 136.0764.

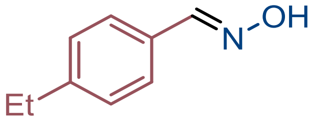

(*E*)-4-ethylbenzaldehyde oxime (5h)

Yield: 64% (95 mg); colourless liquid; **^{1}H NMR** (500 MHz, $CDCl_3$) δ: 8.72 (s, 1H), 8.15 (s,

1H), 7.64-7.39 (m, 2H), 7.25-7.15 (m, 2H), 2.67 (q, J = 7.7 Hz, 2H), 1.25 (t, J = 7.6 Hz, 3H).; **^{13}C {^{1}H} NMR** (126 MHz, $CDCl_3$) δ: 150.4, 146.7, 129.4, 128.4, 127.2, 28.9, 15.4.; **HRMS** (ESI) m/z calcd. $[M+H]^+$ for $C_9H_{12}NO$ 150.0919; found 150.0927.

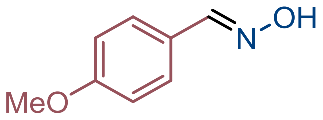

(*E*)-4-methoxybenzaldehyde oxime (5i)[1,2,3,5,6,7,8]

Yield: 70% (106mg); [$BnNH_2$,74% 112 mg] White solid; **MP**: 56 ºC; **^{1}H NMR** (500 MHz, $CDCl_3$) δ: 8.49 (s, 1H), 8.11 (s, 1H), 7.62-7.35 (m, 2H), 6.91 (d, J = 8.8 Hz, 2H), 3.83 (s, 3H).; **^{13}C {^{1}H} NMR** (126 MHz, $CDCl_3$) δ: 161.1, 150.0, 128.6, 124.7, 114.3, 55.4.; **HRMS** (ESI) m/z calcd. $[M+H]^+$ for $C_8H_{10}NO_2$ 152.0712; found 152.0721;

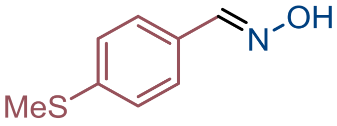

(*E*)-4-(methylthio)benzaldehyde oxime (5j)[7]

Yield: 63% (105 mg); colorless liquid; **^{1}H NMR** (500 MHz, $CDCl_3$) δ: 8.10 (s, 1H), 7.59-7.38 (m, 2H), 7.26-7.20 (m, 2H), 2.50 (d, J = 0.8 Hz, 3H).; **^{13}C {^{1}H} NMR** (126 MHz, $CDCl_3$) δ: 150.0, 141.4, 128.6, 127.4, 126.2, 125.5, 15.4.; **HRMS** (ESI) m/z calcd. $[M+H]^+$ for $C_8H_{10}NOS$ 168.0483; found 168.0485.

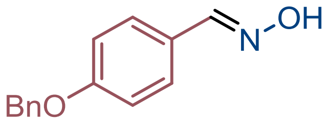

(*E*)-4-(benzyloxy)benzaldehyde oxime (5k)[7]

Yield: 74% (168 mg); colorless liquid ;; **^{1}H NMR** (500 MHz, $CDCl_3$) δ: 8.35 (d, J = 1.9 Hz, 1H), 8.11 (s, 1H), 7.52 (dd, J = 9.2, 2.6 Hz, 2H), 7.48-7.37 (m, 4H), 7.37-7.29 (m, 1H), 6.99 (d, J = 8.6 Hz, 2H), 5.10 (s, 2H).; **^{13}C {^{1}H} NMR** (126 MHz, $CDCl_3$) δ: 160.3, 150.0, 136.6, 128.7, 128.6, 128.2, 127.6, 124.9, 115.2, 70.1.; **HRMS** (ESI) m/z calcd. $[M+H]^+$ for $C_{14}H_{14}NO_2$ 228.1025; found 228.1026.

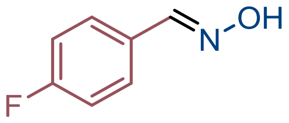

(*E*)-4-fluorobenzaldehyde oxime (5l)[1,2,3,4,5,6,7,8]

Yield: 78% (108 mg); White solid; **MP**: 86 ºC; **^{1}H NMR** (500 MHz, $CDCl_3$) δ: 8.49 (s, 1H), 8.14 (s, 1H), 7.57 (ddd, J = 9.2, 5.8, 2.8 Hz, 2H), 7.15-6.92 (m, 2H) ; **^{13}C {^{1}H} NMR** (126 MHz, $CDCl_3$) δ: 164.9, 162.9, 149.4, 129.0, 128.9, 128.2, 116.2, 116.0.; **^{19}F NMR** (471 MHz, $CDCl_3$) δ: -109.94 -110.05 (m).; **HRMS** (ESI) m/z calcd. $[M+H]^+$ for C_7H_7NOF 140.0512; found 140.0518.

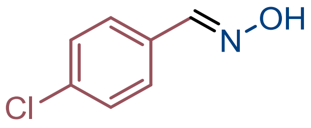

(*E*)-4-chlorobenzaldehyde oxime (5m)[1,2,3,4,5,6,7,8,9]

Yield: 82% (127 mg); White solid; **MP**: 110 ºC; **^{1}H NMR** (500 MHz, $CDCl_3$) δ: 8.11 (s, 1H), 7.86 (s, 1H), 7.57-7.44 (m, 2H), 7.40-7.31 (m, 2H).; **^{13}C {^{1}H} NMR** (126 MHz, $CDCl_3$) δ: 149.4, 136.0, 130.6, 129.2, 128.3.; **HRMS** (ESI) m/z calcd. $[M+H]^+$ for C_7H_7ONCl 156.0216; found 156.0226.

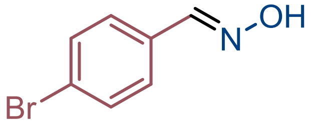

(*E*)-4-bromobenzaldehyde oxime (5n)[1,3,4,5,6,7]

Yield: 86% (172 mg); [$BnNH_2$,71% 142 mg], White solid; **MP**: 108 ºC; **^{1}H NMR** (500 MHz, $CDCl_3$) δ: 8.27 (s, 1H), 8.10 (s, 1H), 7.58-7.48 (m, 2H), 7.48-7.37 (m, 2H).; **^{13}C {^{1}H} NMR** (126 MHz, $CDCl_3$) δ: 149.5, 132.1, 130.9, 128.5, 124.4.; **HRMS** (ESI) m/z calcd. $[M+H]^+$ for C_7H_7NOBr 199.9711; found 199. 9715.

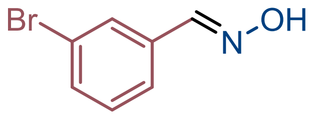

(*E*)-3-bromobenzaldehyde oxime (5o)[10]

Yield: 80% (160 mg); White solid; **MP**: 74 ºC; **^{1}H NMR** (500 MHz, $CDCl_3$) δ: 8.10 (s, 1H), 7.74 (t, J = 1.8 Hz, 1H), 7.54-7.45 (m, 2H), 7.29-7.22 (m, 1H).; **^{13}C {^{1}H} NMR** (126 MHz,

$CDCl_3$) δ: 149.1, 134.0, 133.1, 130.4, 129.9, 125.8, 123.0.; **HRMS** (ESI) m/z calcd. $[M+H]^+$ for C_7H_7NOBr 199.9711; found 199. 9717.

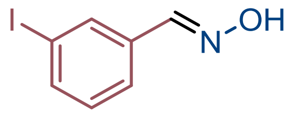

(*E*)-3-iodobenzaldehyde oxime (5p)[11]

Yield: 83% (204 mg); White solid; **MP**: 66 ºC, **^{1}H NMR** (500 MHz, $CDCl_3$) δ: 8.05 (s, 1H), 8.00 (d, *J* = 1.4 Hz, 1H), 7.94 (t, *J* = 1.7 Hz, 1H), 7.71 (ddd, *J* = 7.9, 1.8, 1.0 Hz, 1H), 7.53 (dt, *J* = 7.8, 1.4 Hz, 1H), 7.12 (t, *J* = 7.8 Hz, 1H).; **^{13}C {^{1}H} NMR** (126 MHz, $CDCl_3$) δ: 149.0, 138.9, 135.8, 134.1, 130.5, 126.3, 94.6.; **HRMS** (ESI) m/z calcd. $[M+H]^+$ for C_7H_7NOI 247.9572; found 247.9577

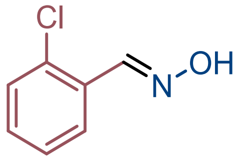

(*E*)-2-chlorobenzaldehyde oxime (5q)[5,6]

Yield: 72% (111 mg); Brown solid; **MP**: 74 ºC. **^{1}H NMR** (500 MHz, $CDCl_3$) δ: 8.58 (s, 1H), 8.11 (s, 1H), 7.83 (dd, *J* = 7.7, 1.8 Hz, 1H), 7.39 (dd, *J* = 7.9, 1.4 Hz, 1H), 7.34-7.30 (m, 1H), 7.30-7.26 (m, 1H).; **^{13}C {^{1}H} NMR** (126 MHz, $CDCl_3$) δ: 147.7, 134.1, 131.1, 130.0, 129.9, 127.2, 127.1.; **HRMS** (ESI) m/z calcd. $[M+H]^+$ for C_7H_7ONCl 156.0216; found 156.0224

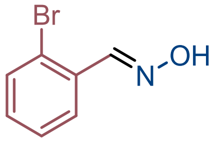

(*E*)-2-bromobenzaldehyde oxime (5r)[12]

Yield: 76% (152 mg); White solid; **MP**: 102 ºC, **^{1}H NMR** (400 MHz, $CDCl_3$) δ: 8.54 (s, 1H), 8.29 – 8.07 (m, 1H), 7.81 (dd, *J* = 7.8, 1.8 Hz, 1H), 7.58 (dd, *J* = 7.9, 1.3 Hz, 1H), 7.32 (td, *J* = 7.6, 1.3 Hz, 1H), 7.26 – 7.21 (m, 1H).; **^{13}C {^{1}H} NMR** (101 MHz, $CDCl_3$) δ ^{13}C NMR (126 MHz, Chloroform-*d*) δ 150.0, 133.3, 131.5, 131.3, 127.7, 127.6, 124.0.; **HRMS** (ESI) m/z calcd. $[M+H]^+$ for C_7H_7NOBr 199.9711; found 199. 9713.

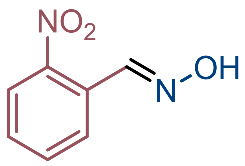

(*E*)-2-nitrobenzaldehyde oxime (5s)[2,8]

Yield: 80% (133 mg); White solid; **MP**: 100 ºC; **^{1}H NMR** (400 MHz, $CDCl_3$) δ: 8.69 (s, 1H), 8.07 (dd, *J* = 8.2, 1.3 Hz, 1H), 8.03 (s, 1H), 7.92 (dd, *J* = 7.8, 1.5 Hz, 1H), 7.65 (td, *J* = 7.6, 1.3 Hz, 1H), 7.56 (td, *J* = 7.8, 1.5 Hz, 1H).; **^{13}C {^{1}H} NMR** (126 MHz, $CDCl_3$) δ: 147.2, 133.6, 130.5, 128.9, 127.3, 125.0.; **HRMS** (ESI) m/z calcd. $[M+H]^+$ for $C_7H_7N_2O_3$ 167.0445; found 167.0449.

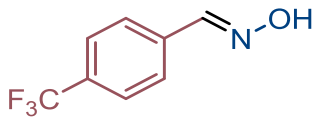

(*E*)-4-(trifluoromethyl)benzaldehyde oxime (5t)[3,4,6,7]

Yield: 88% (166 mg); Yellow solid; **MP**: 90 ºC; **^{1}H NMR** (500 MHz, $CDCl_3$) δ: 8.18 (s, 1H), 7.82 (s, 1H), 7.70 (d, *J* = 8.1 Hz, 2H), 7.64 (d, *J* = 8.4 Hz, 2H).; **^{13}C {^{1}H} NMR** (126 MHz, $CDCl_3$) δ: 149.2, 135.5, 131.9, 131.6, 127.3, 125.9, 125.8, 125.8, 125.8, 125.0, 122.9,120.7.; **^{19}F NMR** (471 MHz, $CDCl_3$) δ: -62.86.; **HRMS** (ESI) m/z calcd. $[M+H]^+$ for $C_8H_7NOF_3$ 190.0480; found 190.0488

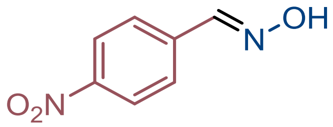

***(E)-4-*nitrobenzaldehyde oxime (5u)**[1,2,5,8]

Yield: 86% (143 mg);[$BnNH_2$,62% 103 mg], light yellow solid; **MP**: 126 ºC; **^{1}H NMR** (500 MHz, MeOD-D_4) δ: 8.21 (d, *J* = 8.9 Hz, 2H), 8.17 (s, 1H), 7.80 (d, *J* = 8.9 Hz, 2H).; **^{13}C {^{1}H} NMR** (126 MHz, MeOD-D_4) δ: 149.3, 147.9, 140.9, 128.3, 124.8,124.8.; **HRMS** (ESI) m/z calcd. $[M+H]^+$ for $C_7H_7N_2O_3$ 167.0445; found 167.0449.

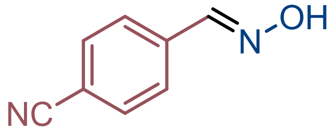

(*E*)-4-((hydroxyimino)methyl)benzonitrile (5v)[3,4]

Yield: 83% (121 mg);[$BnNH_2$,69% 100 mg], White solid; **MP**: 176 ºC; **^{1}H NMR** (500 MHz, DMSO-d_6) δ: 11.73 (s, 1H), 8.24 (s, 1H), 7.86 (d, J = 8.2 Hz, 2H), 7.82-7.71 (m, 2H).; **^{13}C {^{1}H} NMR** (126 MHz, DMSO-d_6) δ: 147.1, 137.6, 132.7, 127.0, 118.7, 111.3.; **HRMS** (ESI) m/z calcd. $[M+H]^+$ for $C_8H_7N_2O$ 147.0558; found 147.0565.

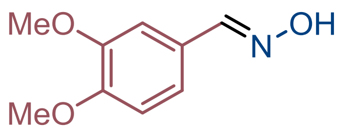

(*E*)-3,4-dimethoxybenzaldehyde oxime (5w)[2,3,7,8]

Yield: 62% (112 mg); White solid; **MP**: 94 ºC; **^{1}H NMR** (500 MHz, $CDCl_3$) δ: 9.37 (s, 1H), 8.33 (d, J = 2.2 Hz, 1H), 7.51-7.40 (m, 1H), 7.27 (dt, J = 8.2, 2.0 Hz, 1H), 7.09 (dd, J = 8.0, 2.3 Hz, 1H), 4.14 (s, 6H).; **^{13}C {^{1}H} NMR** (126 MHz, $CDCl_3$) δ: 150.8, 150.3, 149.3, 124.8, 121.7, 110.8, 108.1, 55.9, 55.9.; **HRMS** (ESI) m/z calcd. $[M+H]^+$ for C_9H_{12} NO_3 182.0817; found 182.0820.

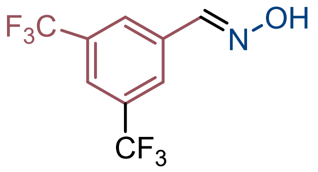

(*E*)-3,5-*bis*(trifluoromethyl)benzaldehyde oxime (5x)[13]

Yield: 90% (230 mg);[$BnNH_2$,73% 187 mg], White Solid; **MP**: 91 ºC; **^{1}H NMR** (400 MHz, $CDCl_3$) δ: 8.21 (s, 1H), 8.03 (d, J = 1.7 Hz, 2H), 7.88 (s, 1H), 7.76 (s, 1H).; **^{13}C {^{1}H} NMR** (126 MHz, $CDCl_3$) δ: 147.7, 134.4, 132.8, 132.5, 132.2, 132.0, 126.9, 126.9, 126.4, 124.2, 123.4, 123.3, 123.3, 123.3,123.3, 122.0, 119.9.; **^{19}F NMR** (471 MHz, $CDCl_3$) δ: -63.10, --63.11.; **HRMS** (ESI) m/z calcd. $[M+H]^+$ for $C_9H_6F_6NO$ 258.0354; found 258.0359.

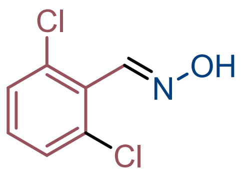

(*E*)-2,6-dichlorobenzaldehyde oxime (5y)[14]

Yield: 70% (133mg);[$BnNH_2$,65% 123 mg], colorless liquid; **^{1}H NMR** (400 MHz, $CDCl_3$) δ: 9.00 (s, 1H), 8.38 (s, 1H), 7.34 (d, J = 8.1 Hz, 2H), 7.22 (dd, J = 9.5, 2.0 Hz, 1H).; **^{13}C {^{1}H} NMR** (126 MHz, $CDCl_3$) δ: 146.3, 135.1, 130.5, 128.9, 128.6.; **HRMS** (ESI) m/z calcd. $[M+H]^+$ for $C_7H_6Cl_2NO$ 189.9826; found 189.9829.

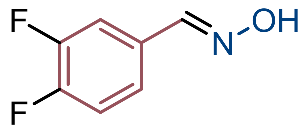

(*E*)-3,4-difluorobenzaldehyde oxime (5z)[15]

Yield: 75% (118 mg);[$BnNH_2$,70% 110 mg], White solid; **^{1}H NMR** (500 MHz, $CDCl_3$) δ: 8.06 (d, *J* = 0.9 Hz, 1H), 7.46 (ddd, *J* = 11.0, 7.7, 2.1 Hz, 1H), 7.41 (s, 1H), 7.29-7.26 (m, 1H), 7.18 (dt, *J* = 9.9, 8.2 Hz, 1H).; **^{13}C {^{1}H} NMR** (126 MHz, $CDCl_3$) δ: 152.6, 152.5, 151.8, 150.6, 149.8, 148.5, 148.5, 148.5, 129.4, 129.3, 129., 129.3, 123.9, 123.9, 123.9, 123.8, 117.8, 117.7, 115.5, 115.3.; **^{19}F NMR** (471 MHz, $CDCl_3$) δ: -134.75 – -134.91 (m), -136.60 – -136.76 (m).; **HRMS** (ESI) m/z calcd. $[M+H]^+$ for $C_7H_6F_2NO$ 158.0417; found 158.0424.

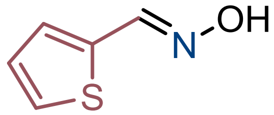

(*E*)-thiophene-2-carbaldehyde oxime (5aa)[1,3,5]

Yield: 60% (76 mg); White solid; **MP**: 130 ºC; **^{1}H NMR** (500 MHz, $CDCl_3$) δ: 8.30 (t, *J* = 0.7 Hz, 1H), 7.94 (s, 1H), 7.34 (dt, *J* = 5.1, 1.0 Hz, 1H), 7.21 (dd, *J* = 3.6, 1.2 Hz, 1H), 7.05 (dd, *J* = 5.1, 3.6 Hz, 1H).; **^{13}C {^{1}H} NMR** (126 MHz, $CDCl_3$) δ: 145.1, 135.5, 129.8, 127.7, 127.4.; **HRMS** (ESI) m/z calcd. $[M+H]^+$ for C_5H_6NOS 128.0170; found 128.0180.

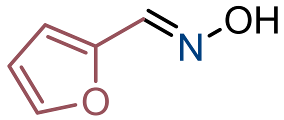

(*E*)-furan-2-carbaldehyde oxime (5ab)[1,5]

Yield: 65% ; (72 mg); [$BnNH_2$,73% 81 mg], White solid; **MP**: 96 ºC; **^{1}H NMR** (500 MHz, $CDCl_3$) δ: 9.20 (s, 1H), 7.61-7.38 (m, 2H), 7.33 (d, *J* = 3.4 Hz, 1H), 6.69-6.23 (m, 1H).; **^{13}C {^{1}H} NMR** (126 MHz, $CDCl_3$) δ: 145.1, 143.5, 137.3, 118.3, 112.4.; **HRMS** (ESI) m/z calcd. $[M+H]^+$ for $C_5H_6NO_2$ 112.0399; found 112.0417.

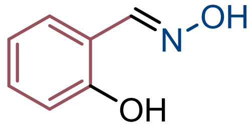

(*E*)-2-hydroxybenzaldehyde oxime (5ac)[1,5]

Yield: 65% (75 mg); [$BnNH_2$,58% 79 mg], White solid; ^{1}**H NMR** (400 MHz, $CDCl_3$) δ: 10.00 (s, 1H), 8.20 (s, 1H), 7.92 (s, 1H), 7.31-7.19 (m, 1H), 7.15 (dd, *J* = 7.6, 1.7 Hz, 1H), 6.97 (dd, *J* = 8.3, 1.0 Hz, 1H), 6.90 (td, *J* = 7.5, 1.2 Hz, 1H).; ^{13}**C {^{1}H} NMR** (126 MHz, $CDCl_3$) δ: 157.4, 153.3, 131.5, 130.9, 119.8, 116.9, 116.3.; **HRMS** (ESI) m/z calcd. $[M+H]^+$ for $C_7H_8NO_2$ 138.0555; found 138.0564.

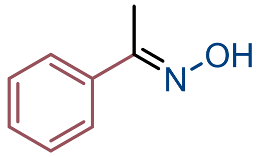

(*E*)-1-phenylethan-1-one oxime (5ad)[2,8]

Yield: 59% (80 mg); White solid; **MP**: 60ºC; ^{1}**H NMR** (400 MHz, $CDCl_3$) δ: 9.82 (s, 1H), 7.75 -7.57 (m, 2H), 7.41 (dt, *J* = 4.8, 3.1 Hz, 3H), 2.34 (s, 3H).; ^{13}**C {^{1}H} NMR** (126 MHz, $CDCl_3$) δ: 156.3, 136.6, 129.3, 128.6, 126.1, 12.2. **HRMS** (ESI) m/z calcd. $[M+H]^+$ for $C_8H_{10}NO$ 136.0762; found 136.0766

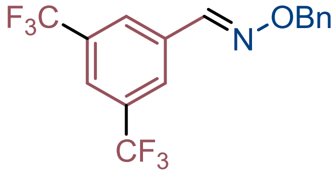

(*E*)-3,5-*bis*(trifluoromethyl)benzaldehyde *O*-benzyl oxime (5ae)

Yield: 72% (248 mg); colourless liquid ; ^{1}**H NMR** (500 MHz, $CDCl_3$) δ: 8.19 (s, 1H), 8.11-7.95 (m, 2H), 7.91-7.80 (m, 1H), 7.53-7.29 (m, 5H), 5.28 (s, 2H).; ^{13}**C {^{1}H} NMR** (126 MHz, $CDCl_3$) δ: 146.0, 136.8, 134.4, 132.3, 132.0, 128.5, 128.4, 128.2, 126.8, 126.7, 124.1, 123.0, 123.0, 123.0, 121.9, 77.1.; ^{19}**F NMR** (471 MHz, $CDCl_3$) δ -63.07.; **HRMS** (ESI) m/z calcd. $[M+H]^+$ for $C_{16}H_{12}F_6NO$ 348.0823; found 348.0831.

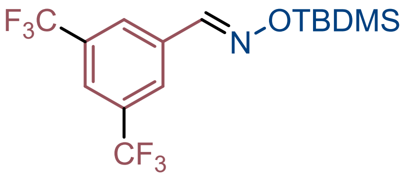

(*E*)-3,5-*bis*(trifluoromethyl)benzaldehyde *O*-(*tert*-butyldimethylsilyl) oxime (5af)

Yield: 68% (261mg); colorless liquid; ^{1}**H NMR** (500 MHz, $CDCl_3$) δ: 8.27 (s, 1H), 8.04-7.96 (m, 2H), 7.86 (dt, *J* = 1.8, 0.9 Hz, 1H), 0.98 (s, 9H), 0.26 (s, 6H).;^{13}**C {^{1}H} NMR** (126 MHz,

$CDCl_3$) δ: 150.7, 150.7, 135.1, 132.4, 132.1, 126.9, 126.8, 126.7, 126.7, 124.3, 123.2, 123.0, 122.1, 26.1, 26.1, 26.0, 18.3, -4.9, -5.0, -5.1.; **^{19}F NMR** (471 MHz, $CDCl_3$) δ: δ -63.12. **HRMS** (ESI) m/z calcd. $[M+H]^+$ for $C_{15}H_{20}F_6NOSi$ 372.1218; found 372.1226.

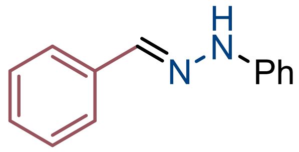

(*E*)-1-benzylidene-2-phenylhydrazine (7a)

Yield: 85% (158 mg); white solid; **^{1}H NMR** (500 MHz, $CDCl_3$) δ: 7.70-7.65 (m, 3H), 7.40 - 7.35 (m, 2H), 7.32 -7.27 (m, 3H), 7.13 (dt, J = 7.8, 1.1 Hz, 2H), 6.88 (d, J = 1.2 Hz, 1H).; **^{13}C {^{1}H} NMR** (126 MHz, $CDCl_3$) δ: 144.7, 137.4, 135.4, 129.4, 128.7, 128.5, 126.3, 120.2, 112.8.; **HRMS** (ESI) m/z calcd. $[M+H]^+$ for $C_{13}H_{13}N_2$ 197.1079 found 197.1085.

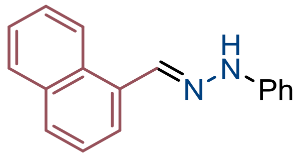

(*E*)-1-(naphthalen-1-ylmethylene)-2-phenylhydrazine(7b)

Yield: 86% (211 mg); Brown soliod; **^{1}H NMR** (500 MHz, $CDCl_3$) δ: 8.73 (d, J = 8.5 Hz, 1H), 8.18 (d, J = 4.0 Hz, 1H), 7.88 – 7.71 (m, 3H), 7.67 (s, 1H), 7.54 (ddd, J = 6.8, 5.0, 3.4 Hz, 1H), 7.51 – 7.38 (m, 2H), 7.27 (ddt, J = 9.3, 5.7, 1.8 Hz, 2H), 7.16 – 7.07 (m, 2H), 6.87 (td, J = 7.4, 1.5 Hz, 1H)..; **^{13}C {^{1}H} NMR** (126 MHz, $CDCl_3$) δ: 144.7, 137.0, 134.0, 130.7, 130.4, 129., 129.3, 129.1, 128.8, 126.8, 126.4, 126.0, 125.5, 124.5, 120.2, 112.9, 112.2. **HRMS** (ESI) m/z calcd. $[M+H]^+$ for $C_{17}H_{15}N_2$ 247.1235; found 247.1239.

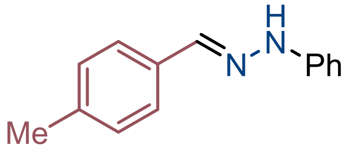

(*E*)-1-(4-methylbenzylidene)-2-phenylhydrazine(7c)

Yield: 85% (179 mg); Pale White solid; **^{1}H NMR** (500 MHz, $CDCl_3$) δ: 7.67 (s, 1H), 7.61-7.47 (m, 3H), 7.27 (dd, J = 8.3, 1.5 Hz, 2H), 7.23-7.14 (m, 2H), 7.14 – 7.05 (m, 2H), 6.91-6.80 (m, 1H), 2.37 (s, 3H). **^{13}C {^{1}H} NMR** (126 MHz, $CDCl_3$) δ: 144.9, 138.6, 137.6, 132.6, 129.4, 129.4, 126.2, 120.0, 112.8, 21.5.; **HRMS** (ESI) m/z calcd. $[M+H]^+$ for $C_{14}H_{15}N_2$ 211.1235; found 211.1243.

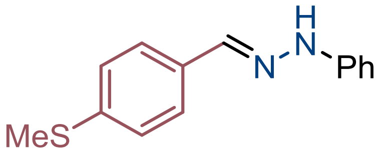

(*E*)-1-(4-(methylthio) benzylidene)-2-phenylhydrazine(7d)

Yield: 83% (201 mg); Pale white solid; **^{1}H NMR** (500 MHz, $CDCl_3$) δ: 7.62 (d, J = 0.9 Hz, 1H), 7.61-7.54 (m, 3H), 7.29-7.22 (m, 4H), 7.13 – 7.07 (m, 2H), 6.91-6.82 (m, 1H), 2.50 (s, 3H).; **^{13}C {^{1}H} NMR** (126 MHz, $CDCl_3$) δ: 144.6, 138.9, 136.8, 132.2, 129.3, 126.5, 126.4, 120.1, 112.7, 15.6. **HRMS** (ESI) m/z calcd. $[M+H]^+$ for $C_{14}H_{15}N_2S$ 243.0956; found 243.0960.

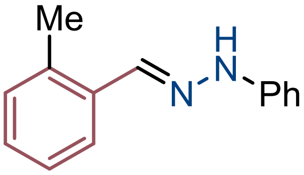

(*E*)-1-(2-methylbenzylidene)-2-phenylhydrazine(7e)

Yield: 80% (170 mg); Brown solid; **^{1}H NMR** (500 MHz, $CDCl_3$) δ: 7.93 (s, 1H), 7.84 (dd, J = 7.3, 1.9 Hz, 1H), 7.66 (s, 1H), 7.33-7.27 (m, 2H), 7.26 – 7.17 (m, 3H), 7.13 (dt, J = 7.7, 1.1 Hz, 2H), 6.95 – 6.85 (m, 1H).; **^{13}C {^{1}H} NMR** (126 MHz, $CDCl_3$) δ: 144.8, 136.4, 135.5, 133.2, 130.9, 129.4, 129.3, 128.2, 126.5, 126.2, 120.1, 112.8, 112.2, 20.3.; **HRMS** (ESI) m/z calcd. $[M+H]^+$ for $C_{14}H_{15}N_2$ 211.1235; found 211.1244.

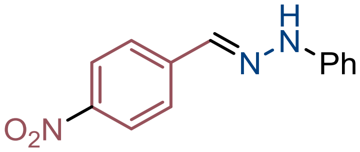

(*E*)-1-(4-nitrobenzylidene)-2-phenylhydrazine(7f)

Yield: 75% (180 mg); Red solid; **^{1}H NMR** (500 MHz, $CDCl_3$) δ: 8.22 (d, J = 8.9 Hz, 2H), 7.99 (s, 1H), 7.77 (d, J = 8.8 Hz, 2H), 7.69 (d, J = 1.2 Hz, 1H), 7.37 – 7.29 (m, 2H), 7.18-7.11 (m, 2H), 6.95 (tt, J = 7.3, 1.1 Hz, 1H).; **^{13}C {^{1}H} NMR** (126 MHz, $CDCl_3$) δ: 147.1, 143.7, 141.8, 133.9, 129.5, 126.3, 124.2, 121.4, 113.2.; **HRMS** (ESI) m/z calcd. $[M+H]^+$ for $C_{13}H_{12}N_3O_2$ 242.0930; found 242.0938.

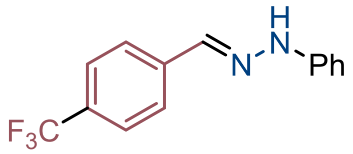

(*E*)-1-phenyl-2-(4-(trifluoromethyl)benzylidene)hydrazine(7g)

Yield: 79% (208 mg); Pale yellow Solid; **^{1}H NMR** (500 MHz, $CDCl_3$) δ: 7.82-7.70 (m, 3H), 7.69 (s, 1H), 7.61 (d, J = 8.0 Hz, 2H), 7.34-7.26 (m, 2H), 7.14 (d, J = 8.0 Hz, 2H), 6.92 (td, J = 7.3, 1.3 Hz, 1H)..; **^{13}C {^{1}H} NMR** (126 MHz, $CDCl_3$) δ: 144.2, 138.8, 135.2, 129.5, 126.2, 125.7, 125.6, 125.6, 120.8, 113.0.; **HRMS** (ESI) m/z calcd. $[M+H]^+$ for $C_{14}H_{12}F_3N_2$ 265.0953; found 265.0959.

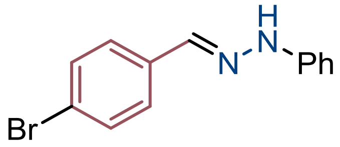

(*E*)-1-(4-bromobenzylidene)-2-phenylhydrazine(7h)

Yield: 82% (225 mg); White solid; **^{1}H NMR** (500 MHz, $CDCl_3$) δ: 7.64 (s, 1H), 7.60 (d, J = 1.0 Hz, 1H), 7.55-7.45 (m, 4H), 7.33-7.25 (m, 2H), 7.16-7.07 (m, 2H), 6.93 – 6.85 (m, 1H). **^{13}C {^{1}H} NMR** (126 MHz, $CDCl_3$) δ: 144.4, 135.9, 134.4, 131.8, 129.4, 127.6, 122.3, 120.5, 112.9.; **HRMS** (ESI) m/z calcd. $[M+H]^+$ for $C_{13}H_{12}BrN_2$ 275.0184; found 275.0189.

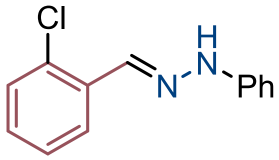

(*E*)-1-(2-chlorobenzylidene)-2-phenylhydrazine(7i)

Yield: 76% (175 mg); Brown solid; **^{1}H NMR** (500 MHz, $CDCl_3$) δ: 8.06 (dd, J = 8.0, 1.4 Hz, 2H), 7.87-7.72 (m, 1H), 7.32 (dd, J = 7.9, 1.4 Hz, 1H), 7.30-7.21 (m, 3H), 7.21-7.15 (m, 1H), 7.13-7.07 (m, 2H), 6.89 (tt, J = 7.3, 1.2 Hz, 1H)..; **^{13}C {^{1}H} NMR** (126 MHz, $CDCl_3$) δ:144.4, 133.5, 132.7, 132.6, 129.8, 129.4, 129.2, 127.0, 126.6, 120.5, 112.9.; **HRMS** (ESI) m/z calcd. $[M+H]^+$ for $C_{13}H_{12}ClN_2$ 231.0689; found 231.0696.

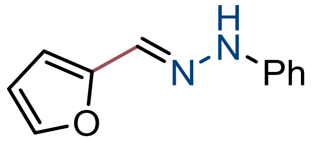

(*E*)-1-(furan-2-ylmethylene)-2-phenylhydrazine(7j)

Yield: 63% (117 mg); red solid; **^{1}H NMR** (500 MHz, $CDCl_3$) δ: 7.54 (d, *J* = 4.8 Hz, 2H), 7.44 (d, *J* = 1.8 Hz, 1H), 7.26 – 7.18 (m, 2H), 7.10 – 6.99 (m, 2H), 6.85 (t, *J* = 7.3 Hz, 1H), 6.53 (d, *J* = 3.4 Hz, 1H), 6.43 (dd, *J* = 3.4, 1.9 Hz, 1H)..; **^{13}C {^{1}H} NMR** (126 MHz, $CDCl_3$) δ: 150.6, 144.4, 143.1, 129.4, 127.8, 120.4, 112.9, 111.7, 109.3.; **HRMS** (ESI) m/z calcd. $[M+H]^+$ for $C_{11}H_{11}N_2O$ 187.0871; found 187.0877.

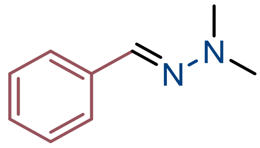

(*E*)-2-benzylidene-1,1-dimethylhydrazine(7k)[16, 17,18,19]

Yield: 74% (94 mg););[$BnNH_2$,63% 93 mg], colorless liquid; **^{1}H NMR** (500 MHz, $CDCl_3$) δ 7.59-7.39 (m, 2H), 7.26 (dd, *J* = 8.4, 6.9 Hz, 2H), 7.22-7.09 (m, 2H), 2.91 (s, 6H).; **^{13}C {^{1}H} NMR** (126 MHz, $CDCl_3$) δ: 136.9, 128.5, 127.4, 125.7, 42.9.; **HRMS** (ESI) m/z calcd. $[M+H]^+$ for $C_9H_{13}N$ 149.1079; found 149.1085.

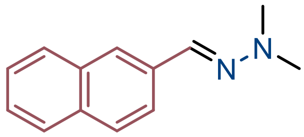

(*E*)-1,1-dimethyl-2-(naphthalen-2-ylmethylene)hydrazine (7l)[19]

Yield: 80% (138 mg););[$BnNH_2$,71% 82 mg], White solid; **MP**: 70ºC; **^{1}H NMR** (500 MHz, $CDCl_3$) δ: 7.92 (dt, *J* = 8.7, 1.7 Hz, 1H), 7.80 (ddd, *J* = 11.5, 6.8, 3.1 Hz, 4H), 7.50-7.33 (m, 3H), 3.03 (s, 6H).; **^{13}C {^{1}H} NMR** (126 MHz, $CDCl_3$) δ: 133.7, 133.2, 128.2, 128.0, 127.8, 126.2, 125.7, 123.1, 43.0.; **HRMS** (ESI) m/z calcd. $[M+H]^+$ for $C_{13}H_{15}N_2$ 199.1235; found 199.1243.

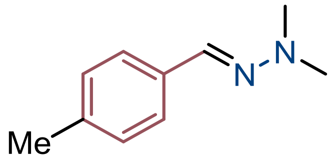

(*E*)-1,1-dimethyl-2-(4-methylbenzylidene)hydrazine(7m)[16]

Yield: 76% (106 mg););[$BnNH_2$,63% 102 mg], colourless liquid; **^{1}H NMR (**500 MHz, $CDCl_3$) δ: 7.55-7.40 (m, 2H), 7.30 (s, 1H), 7.14 (d, *J* = 7.9 Hz, 2H), 2.95 (s, 6H), 2.34 (s, 3H).; **^{13}C {^{1}H} NMR** (126 MHz, $CDCl_3$) δ: 137.4, 134.1, 129.3, 125.7, 43.0, 21.3.; **HRMS** (ESI) m/z calcd.

[M+H]$^+$ for $C_{10}H_{15}N_2$ 163.1235; found 163.123539.

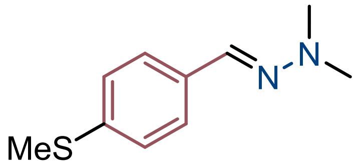

(*E*)-1,1-dimethyl-2-(4-(methylthio)benzylidene)hydrazine(7n)[21]

Yield: 71% (118 mg); colorless liquid; **^{1}H NMR** (500 MHz, CDCl$_3$**)** δ: 7.54-7.41 (m, 2H), 7.26 (s, 1H), 7.24-7.19 (m, 2H), 2.96 (s, 6H), 2.48 (s, 3H).; **^{13}C NMR (**126 MHz, CDCl$_3$**)** δ: 137.3, 134.0, 126.7, 126.7, 126.7, 126.0, 126.0, 126.0, 42.9, 42.9, 42.9, 16.0, 16.0, 15.9.; **HRMS** (ESI) m/z calcd. [M+H]$^+$ for $C_{10}H_{15}N_2S$ 195.0956; found 195.0958.

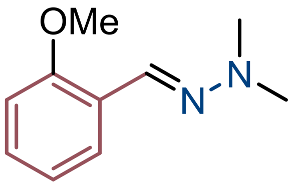

(*E*)-2-(2-methoxybenzylidene)-1,1-dimethylhydrazine(7o)[19]

Yield: 69% (104 mg););[BnNH$_2$,61% 109 mg], colorless liquid; **^{1}H NMR (**500 MHz, CDCl$_3$) δ: 7.86 (dd, *J* = 7.8, 1.8 Hz, 1H), 7.63 (s, 1H), 7.21 (ddd, *J* = 8.5, 7.4, 1.8 Hz, 1H), 6.95 (t, *J* = 7.5 Hz, 1H), 6.87 (dd, *J* = 8.3, 1.1 Hz, 1H), 3.86 (s, 3H), 2.98 (s, 6H).; **^{13}C {^{1}H} NMR** (126 MHz, CDCl$_3$) δ: 156.6, 128.4, 125.4, 125.06, 120.9, 110.8, 55.5, 43.1.; **HRMS** (ESI) m/z calcd. [M+H]$^+$ for $C_{10}H_{15}N_2O$ 179.1184; found 179.1189.

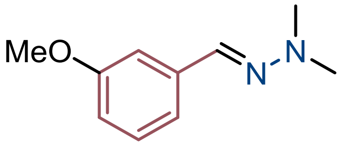

(*E*)-2-(3-methoxybenzylidene)-1,1-dimethylhydrazine(7p)[21]

Yield: 77% (119 mg); colorless liquid; **^{1}H NMR** (500 MHz, CDCl$_3$**)** δ: 7.44 -7.30 (m, 3H), 7.25 (d, *J* = 7.9 Hz, 1H), 6.93 (ddd, *J* = 8.2, 2.7, 1.0 Hz, 1H), 3.97 (s, 3H), 3.11 (s, 6H).; **^{13}C {^{1}H} NMR** (126 MHz, CDCl$_3$) δ: 159.9, 138., 129.5, 118.8, 113.8, 109.7, 55.3, 42.9.; **HRMS** (ESI) m/z calcd. [M+H]$^+$ for $C_{10}H_{15}N_2O$ 179.1184; found 179.1189.

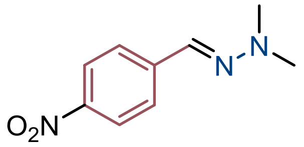

(*E*)-1,1-dimethyl-2-(4-nitrobenzylidene)hydrazine(7q)

Yield: 82% (139 mg););[BnNH$_2$,60% 115 mg], Yellow solid; **MP**: 111ºC; **^{1}H NMR** (500 MHz, CDCl$_3$**)** δ: 8.14 (dd, *J* = 9.0, 2.1 Hz, 2H), 7.62 (dd, *J* = 9.0, 2.1 Hz, 2H), 7.10 (s, 1H), 3.09 (s, 6H).; **^{13}C {^{1}H} NMR** (126 MHz, CDCl$_3$) δ: 146.0, 143.7, 143.7, 125.2, 124.1, 42.6.; **HRMS** (ESI) m/z calcd. [M+H]$^+$ for $C_9H_{12}N_3O_2$ 194.1093; found 194.1098.

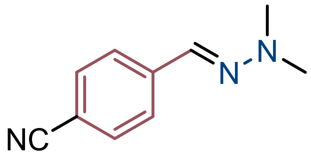

(*E*)-4-((2,2-dimethylhydrazineylidene) methyl) benzonitrile(7r)[21]

Yield: 70% (129 mg); White solid; **MP**: 140ºC; **^{1}H NMR (500 MHz** CDCl$_3$**))** δ: 7.68-7.46 (m, 4H), 7.10 (s, 1H), 3.05 (s, 6H).; **^{13}C {^{1}H} NMR** (126 MHz, CDCl$_3$) δ: 136.9, 128.5, 127.4, 125.7, 42.9.; **HRMS** (ESI) m/z calcd. [M+H]$^+$ for $C_{10}H_{12}N_3$ 174.1026; found 174.1039.

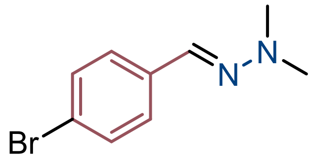

(*E*)-2-(4-bromobenzylidene)-1,1-dimethylhydrazine (7s)[21]

Yield: 70% (136 mg); White solid; **MP**: 60ºC; **^{1}H NMR** (500 MHz, CDCl$_3$) δ: 7.43 (s, 4H), 7.15 (s, 1H), 2.98 (s, 6H).; **^{13}C {^{1}H} NMR** (126 MHz, CDCl$_3$) δ: 136.0, 131.6, 127.1, 120.9, 42.8.; **HRMS** (ESI) m/z calcd. [M+H]$^+$ for $C_9H_{12}BrN_2$ 227.0184; found 227.0187.

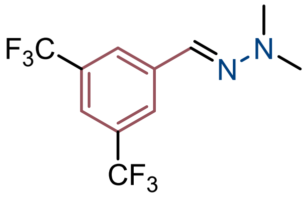

(*E*)-2-(3,5-*bis*(trifluoromethyl)benzylidene)-1,1-dimethylhydrazine(7t)

Yield: 83% (207 mg); colorless liquid; **^{1}H NMR (500 MHz,** CDCl$_3$) δ: 8.03-7.87 (m, 2H), 7.64 (dt, *J* = 1.9, 1.0 Hz, 1H), 7.13 (s, 1H), 3.06 (d, *J* = 0.8 Hz, 6H).; **^{13}C {^{1}H} NMR** (126 MHz,

CDCl$_3$) δ: 139.4, 131.9, 131.6, 131.3, 127.0, 127.0, 124.9, 124.9, 124.7, 122.5, 119.8, 119.8, 119.8, 119.8, 42.6, 42.6.; **^{19}F NMR** (471 MHz, CDCl$_3$) δ: -63.03 (d, *J* = 2.7 Hz).; **HRMS** (ESI) m/z calcd. $[M+H]^+$ for $C_{11}H_{11}F_6N_2$ 285.0826; found 285.0835.

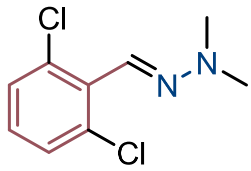

(*E*)-2-(2,6-dichlorobenzylidene)-1,1-dimethylhydrazine(7u)

Yield: 78% (148 mg); colorless liquid; **^{1}H NMR (500 MHz,** CDCl$_3$) δ 7.30 (d, *J* = 8.1 Hz, 2H), 7.26 (d, *J* = 4.1 Hz, 1H), 7.06 (t, *J* = 8.1 Hz, 1H), 3.04 (s, 6H).; **^{13}C {^{1}H} NMR** (126 MHz, CDCl$_3$) δ: 134.3, 132.8, 128.8, 128.0, 42.6.; **HRMS** (ESI) m/z calcd. $[M+H]^+$ for $C_9H_{11}Cl_2N_2$ 217.0299; found 217.0310.

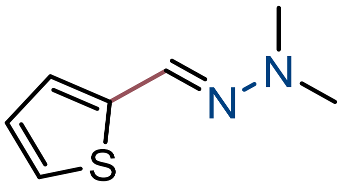

(*E*)-1,1-dimethyl-2-(thiophen-2-ylmethylene)hydrazine(7v)

Yield: 75% (100 mg); colorless liquid; **^{1}H NMR** (500 MHz, CDCl$_3$) δ: 7.43 (s, 1H), 7.14 (d, *J* = 5.1 Hz, 1H), 7.11-6.87 (m, 2H), 2.94 (s, 6H).; **^{13}C {^{1}H} NMR** (126 MHz, CDCl$_3$) δ: 142.6, 127.0, 124.6, 124.1, 42.9.; **HRMS** (ESI) m/z calcd. $[M+H]^+$ for C7$H_{11}N_2S$ 155.0643; found 155.0647.

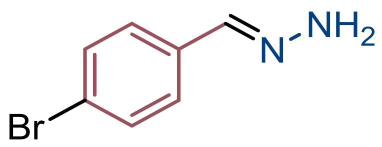

(*E*)-(4-bromobenzylidene)hydrazine (7w)

Yield: 81% (161 mg); White solid; **^{1}H NMR** (500 MHz, CDCl$_3$) δ: 7.63 (s, 1H), 7.47-7.40 (m, 2H), 7.40-7.33 (m, 2H), 5.61 (s, 2H)..; **^{13}C {^{1}H} NMR** (126 MHz, CDCl$_3$) δ:141.4, 134.2, 131.7, 127.5, 122.4.; **HRMS** (ESI) m/z calcd. $[M+H]^+$ for $C_7H_8BrN_2$ 198.9871; found 198.9880.

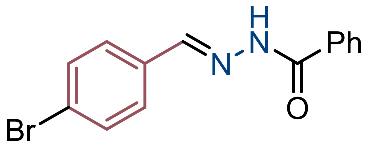

(*E*)-*N'*-(4-bromobenzylidene)benzohydrazide (7x)

Yield: 76% (230 mg); White solid; **^{1}H NMR** (500 MHz, DMSO-d6) δ: 11.94 (s, 1H), 8.44 (s, 1H), 8.04-7.82 (m, 2H), 7.67 (q, J = 8.4 Hz, 4H), 7.59 (d, J = 7.6 Hz, 1H), 7.53 (t, J = 7.6 Hz, 2H). ; **^{13}C {^{1}H} NMR** (126 MHz, DMSO-d6) δ:163.2, 146.5, 133.6, 133.3, 131.8, 128.9, 128.5, 127.6, 123.3.; **HRMS** (ESI) m/z calcd. $[M+H]^+$ for $C_{14}H_{12}F_3N_2$ 265.0953; found 265.0962.

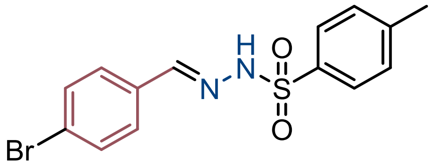

(*E*)-*N'*-(4-bromobenzylidene)-4-methylbenzenesulfonohydrazide(7y)

Yield: 78% (274 mg); White solid; **^{1}H NMR** (500 MHz, $CDCl_3$) δ: 10.87 (s, 1H), 7.82-7.45 (m, 3H), 7.30-7.09 (m, 4H), 7.07 (d, J = 7.8 Hz, 2H), 2.16 (s, 3H).; **^{13}C {^{1}H} NMR** (126 MHz, $CDCl_3$) δ: 145.0, 143.1, 135.8, 132.5, 131.2, 129.1, 128.0, 127.0, 123.4, 21.1.; **HRMS** (ESI) m/z calcd. $[M+H]^+$ for $C_{14}H_{14}BrN_2O_2S$ 352.9959; found 352.9966.

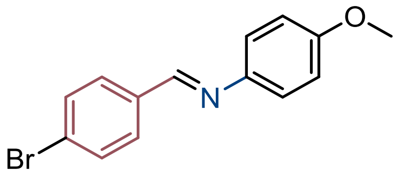

(*E*)-1-(4-bromophenyl)-*N*-(4-methoxyphenyl)methanimine(7z)

Yield: 65% (188 mg); Brown solid; **^{1}H NMR** (500 MHz, $CDCl_3$) δ: 8.42 (s, 1H), 7.75 (d, J = 8.5 Hz, 2H), 7.59 (d, J = 8.5 Hz, 2H), 7.24 (d, J = 8.9 Hz, 2H), 6.93 (d, J = 8.9 Hz, 2H), 3.83 (s, 3H).; **^{13}C {^{1}H} NMR** (126 MHz, $CDCl_3$) δ: 158.6, 156.8, 144.5, 135.4, 132.0, 130.0, 125.5, 122.3, 114.5, 55.6. **HRMS** (ESI) m/z calcd. $[M+H]^+$ for $C_{14}H_{12}BrNO$ 290.0175; found 290.0187

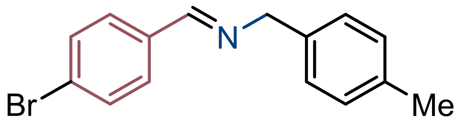

(*E*)-1-(4-bromophenyl)-*N*-(4-methylbenzyl)methanimine (7aa)

Yield: 60% (173 mg); white solid; **^{1}H NMR** (500 MHz, $CDCl_3$) δ: 8.32 (d, *J* = 1.5 Hz, 1H), 7.70- 7.57 (m, 2H), 7.60-7.48 (m, 2H), 7.22 (d, *J* = 8.1 Hz, 2H), 7.16 (d, *J* = 7.8 Hz, 2H), 4.77 (d, *J* = 1.4 Hz, 2H), 2.34 (s, 3H).; **^{13}C {^{1}H} NMR** (126 MHz, $CDCl_3$) δ: 160.5, 136.8, 136.0, 135.2, 131.9, 129.8, 129.3, 128.1, 125.2, 64.9, 21.2. **HRMS** (ESI) m/z calcd. $[M+H]^+$ for $C_{14}H_{14}BrN$ 288.0382; found 288.0389.

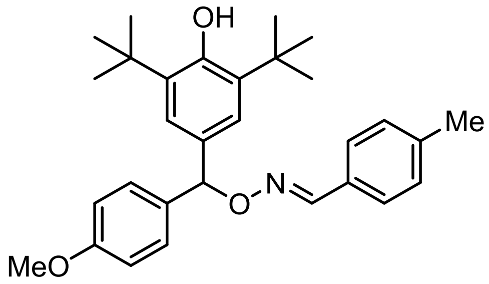

(*E*)-4-methylbenzaldehyde *O*-((3,5-*di*-*tert*-butyl-4-hydroxyphenyl)(4-methoxyphenyl) methyl) oxime (10)

Yield: 57% (25 mg); colorless gummy; **^{1}H NMR (500 MHz,** $CDCl_3$**)** δ: 8.21 (s, 1H), 7.45 (d, J = 8.2 Hz, 2H), 7.32 (d, J = 8.6 Hz, 2H), 7.17 (s, 2H), 7.14 (d, J = 7.9 Hz, 2H), 6.89 (d, J = 8.7 Hz, 2H), 6.24 (s, 1H), 5.18 (s, 1H), 3.81 (s, 3H), 2.35 (s, 3H), 1.41 (s, 18H).; **^{13}C {^{1}H} NMR** (126 MHz, $CDCl_3$) δ:158.9, 153.4, 149.2, 139.9, 135.6, 134.2, 131.7, 129.7, 129.4, 128.8, 127.2, 124.8, 113.7, 87.1, 55.3, 34.4, 30.4, 21.5.; **HRMS** (ESI) m/z calcd. $[M+H]^+$ for $C_{30}H_{38}NO_3$ 460.2852; found 460.2858.

References:

[1] J. Yu, X.Cao, and M. Lu, *Tetrahedron Lett.,* 2014, **55**, 5751-5755.

[2] V. V. Patil, E. M. Gayakwad, and G. S. Shankarling, *New J. Chem.* 2015, **39**, 6677-6682.

[3] S. Yang, Y. Wang, W. Xu, X. Tian, M. Bao, and X. Yu. *Org. Lett. 2023, **25**, 8834−8838*

[4] S. Wang, R. Xiang, P. Liao, J. Kang, S. Li, M. Mao, L. Liu, and G. Li, *Angew. Chem. Int. Ed.,* 2024, **63**, e202405553.

[5] J. Yu, Y. Jin, and M. Lua, *Adv. Synth. Catal.,* 2015, **357**, 1175-1180.

[6] L. Zhang, H. Chen, Z. Zha and Z. Wang, *Chem. Commun.,* 2012, **48**, 6574-6576.

[7] D. Manikpuri, R. V. Sankar, and C. Gunanathan; *Chem Asian J. 2023, **18**,* e202300678

[8] V. V. Patil, E. M. Gayakwad, and G. S. Shankarling; *J. Org. Chem*., 2016, 81, 781-786

[9] L. D. Nunno, P. Vitale, A. Scilimati., *Tetrahedron.,* 2008, **64**, 11198–11204

[10] C. B. Aakero¨y, D. J. Salmon, M, M. Smith, and J Desper; *Crystal Growth & Design*., 2006, **6**, 1035

[11] C. B. Aakeröy, A. S. Sinha, K. N. Epa, C. L. Spartz and J. Desper, *Chem. Commun.,* 2012, **48**, 11289-11291

[12] G. Zhang, X. Wen, Y. Wang, X. Han, Y. Luan, L. Zheng, C. Ding and X. Cao; *RSC Adv.,* 2013, **3**, 22918-22921.

[13] R. S. Torrientes, J, Pascual, I. G. Benito, S. Collavini, I. Kosta, R. Tena-Zaera, N. Martín, and J. L. Delgado. *ChemSusChem.,* 2017*,* **10***,* 2023- 2029

[14] P. R. Maloney, D. J. Parks, C. D. Haffner, A. M. Fivush, G. Chandra, K. D. Plunket, K. L. Creech, L. B. Moore, J. G. Wilson, M. C. Lewis, S. A. Jones, T. M. Willson. *J. Med. Chem,* 2000, **43**, 2971-2974.

[15] M. R. Barbachyn, G. J. Cleek, L. A. Dolak, S. A. Garmon, J. Morris, E. P. Seest, R. C. Thomas, D. S. Toops, W. Watt, D. G. Wishka, C. W. Ford, G. E. Zurenko, J. C. Hamel, R. D. Schaadt, D. Stapert, B. H. Yagi, W. J. Adams, J. M. Friis, J. G. Slatter, J. P. Sams, N. L. Oien, M. J. Zaya, L. C. Wienkers, and M. A. Wynald, *J. Med. Chem*., 2003, **Vol. 46, No. 2,** 287

[16] V. Sridharan, P. Ribelles, V. EstØvez, M. Villacampa, M. Teresa Ramos, P. T. Perumal, and J. C. Menondez, *Chem. Eur. J.* 2012, **18***,* 5056-5063

[17] F. Noël, L. El Kaïm, G. Masson, and Aurélie Claraz, *Org. Biomol. Chem*., 2024, **22**, 4269,

[18] Eve M. Carter, Fabiana Subrizi, John M. Ward, Tom D. Sheppard, and Helen C. Hailes, *ChemCatChem,* 2021*,* **13***,* 4520 - 4523,

[19] A. V. Dubrovskiy and R. C. Larock, *ACS Catal*., 2016, **6**, 7197-7201

[20] A. Ros, R. López-Rodríguez, B. Estepa, E. Á. lvarez, R. Fernández, and J. M. Lassaletta, *J. Am. Chem. Soc.,* 2012, **134**, 4573-4576

[21] B. Janhsen and A. Studer, *J. Org. Chem*., 2017, **82**, 11703-11710.

Spectra’s

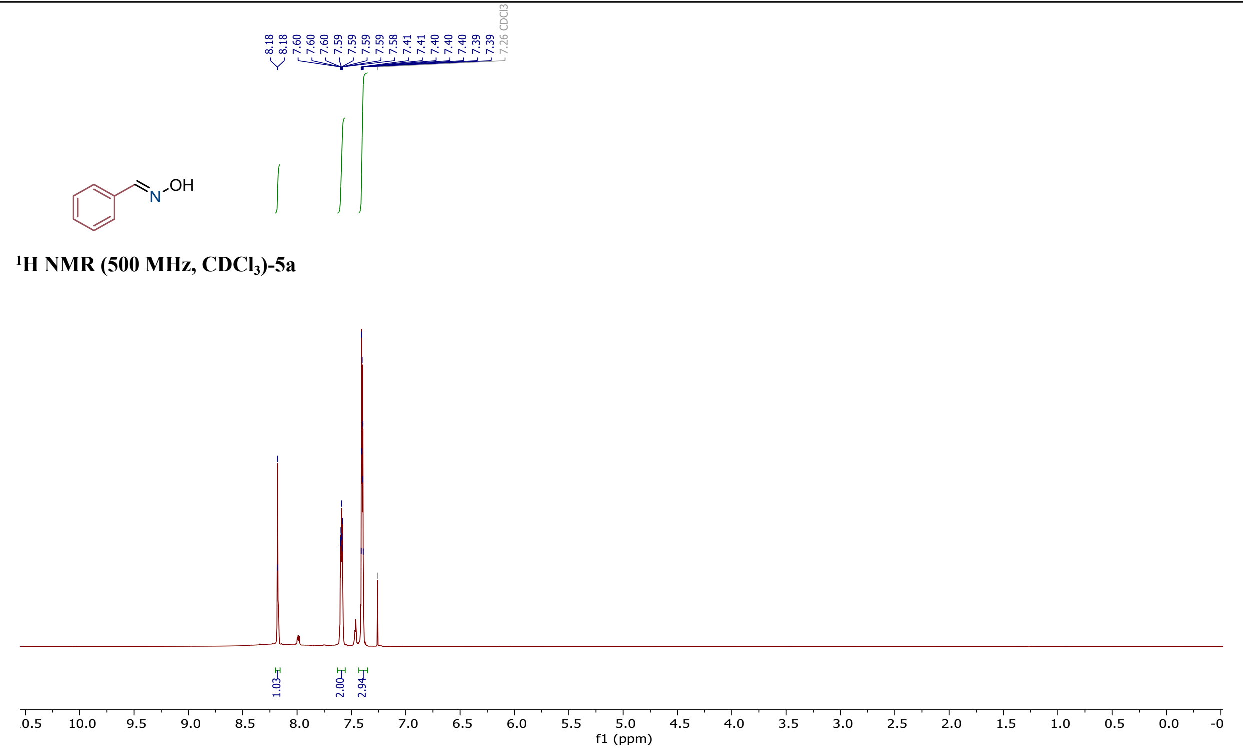

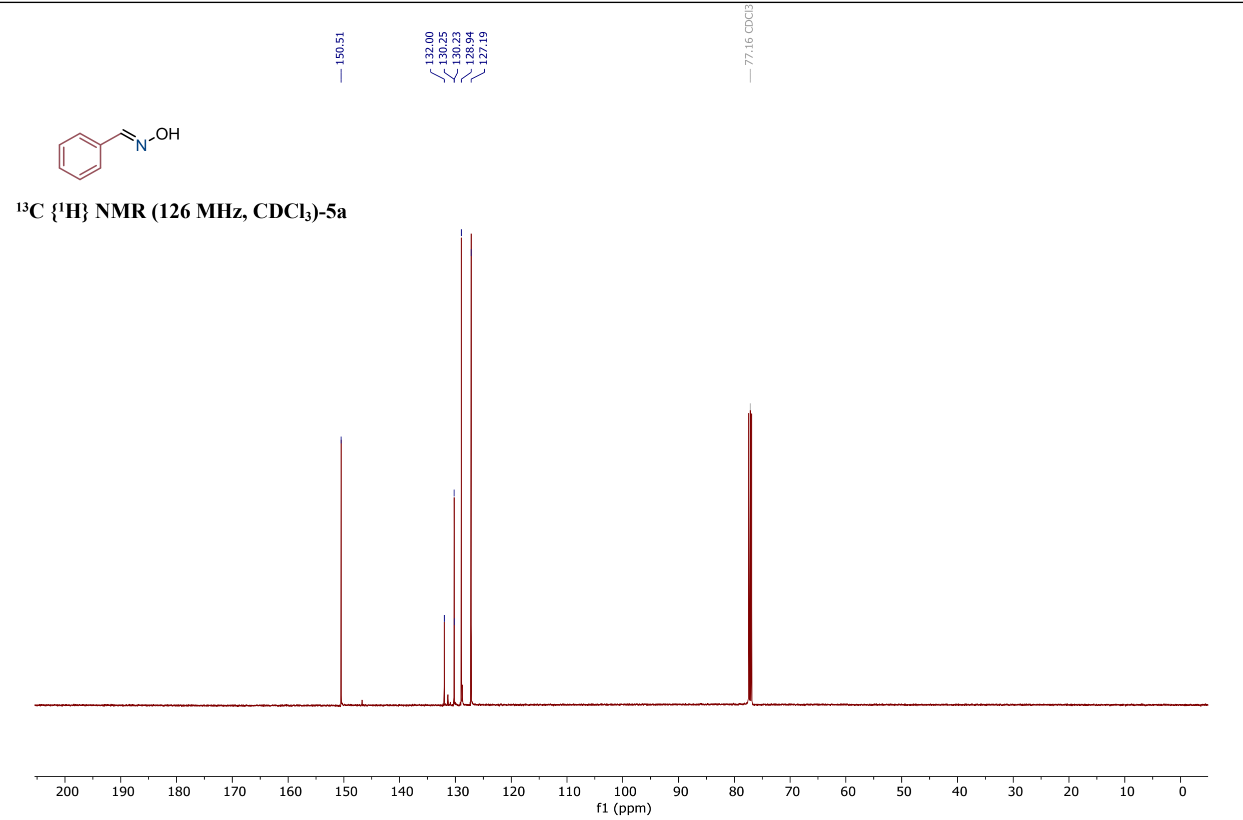

(*E*)-benzaldehyde oxime (5a)

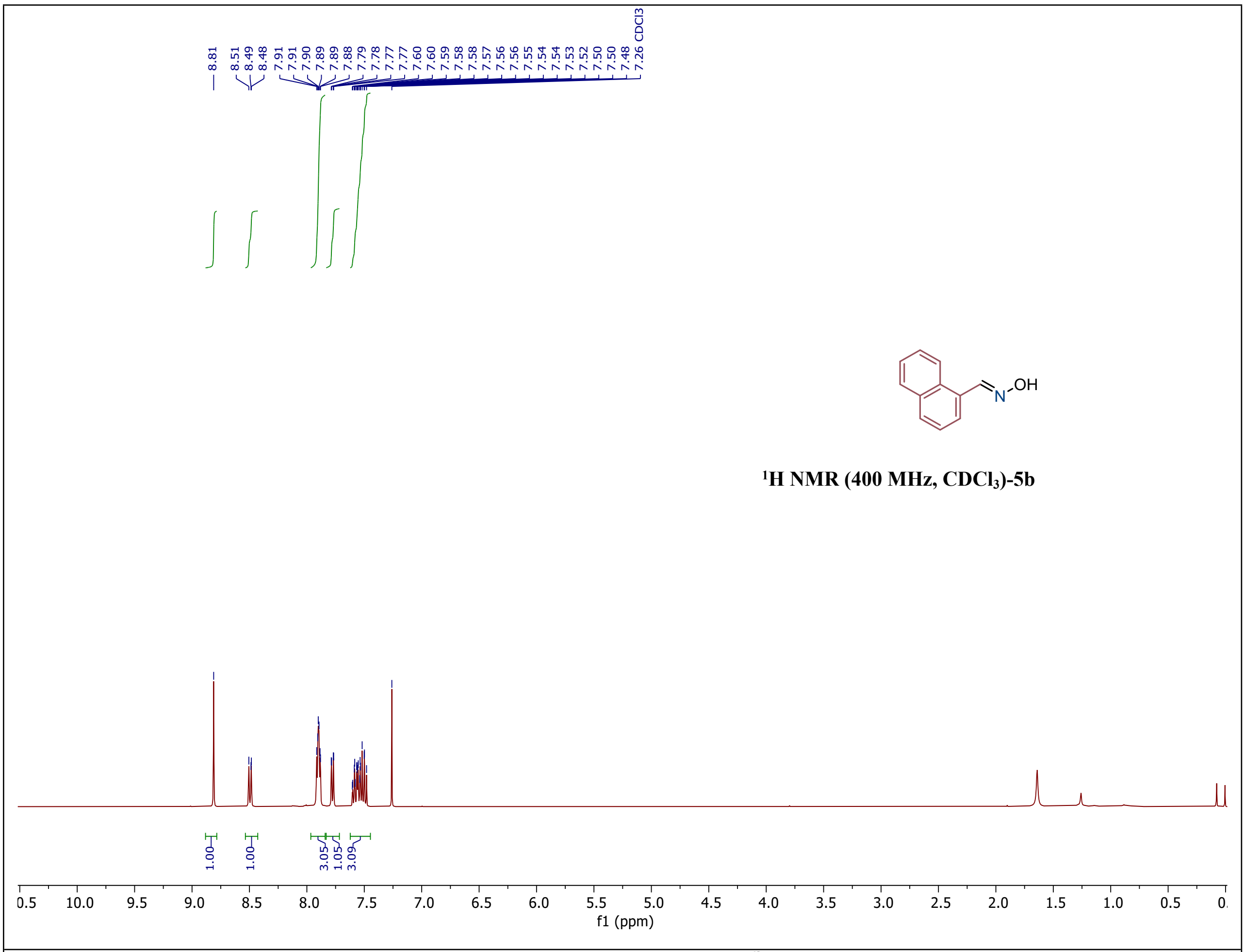

^{1}H NMR (400 MHz, $CDCl_3$)-5b

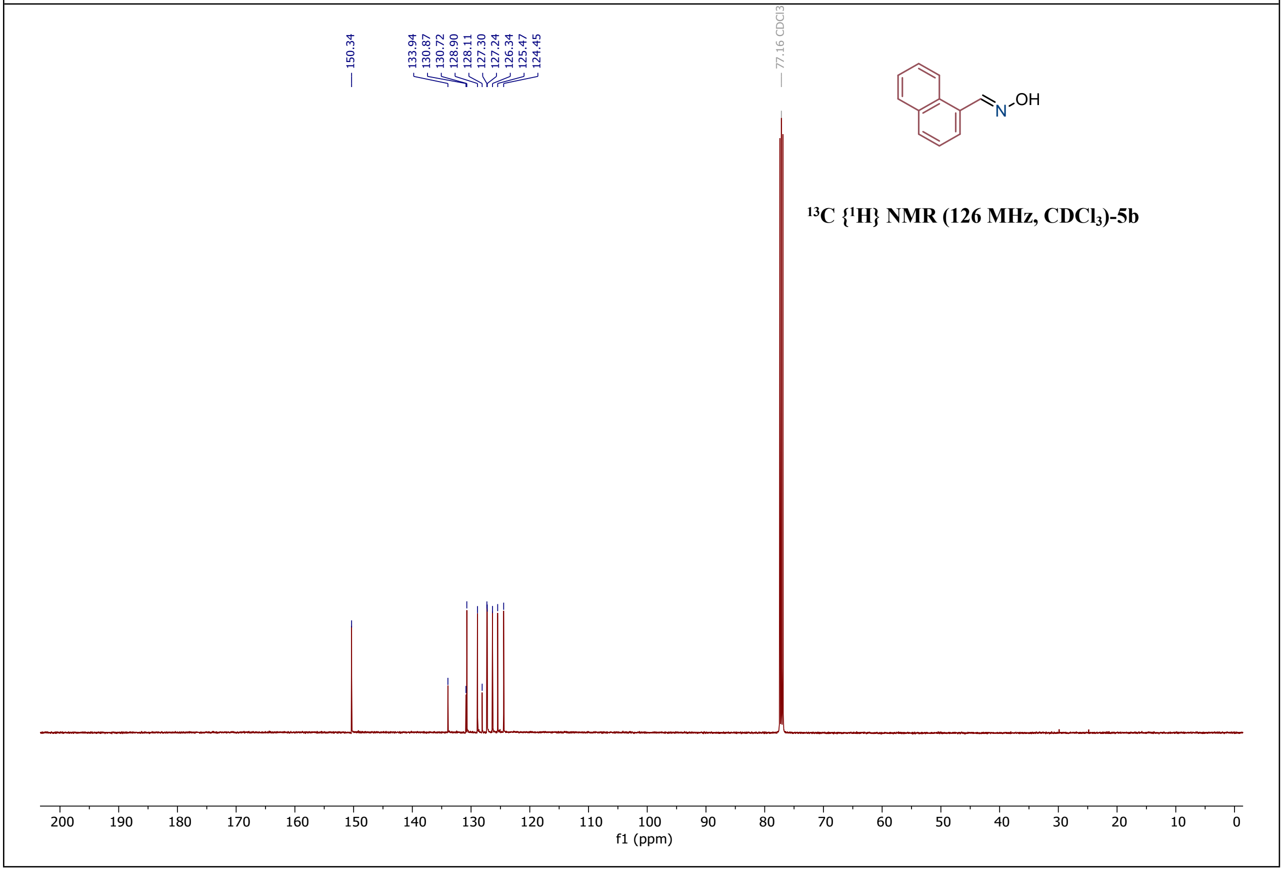

^{13}C {^{1}H} NMR (126 MHz, $CDCl_3$)-5b

(*E*)-1-naphthaldehyde oxime (5b)

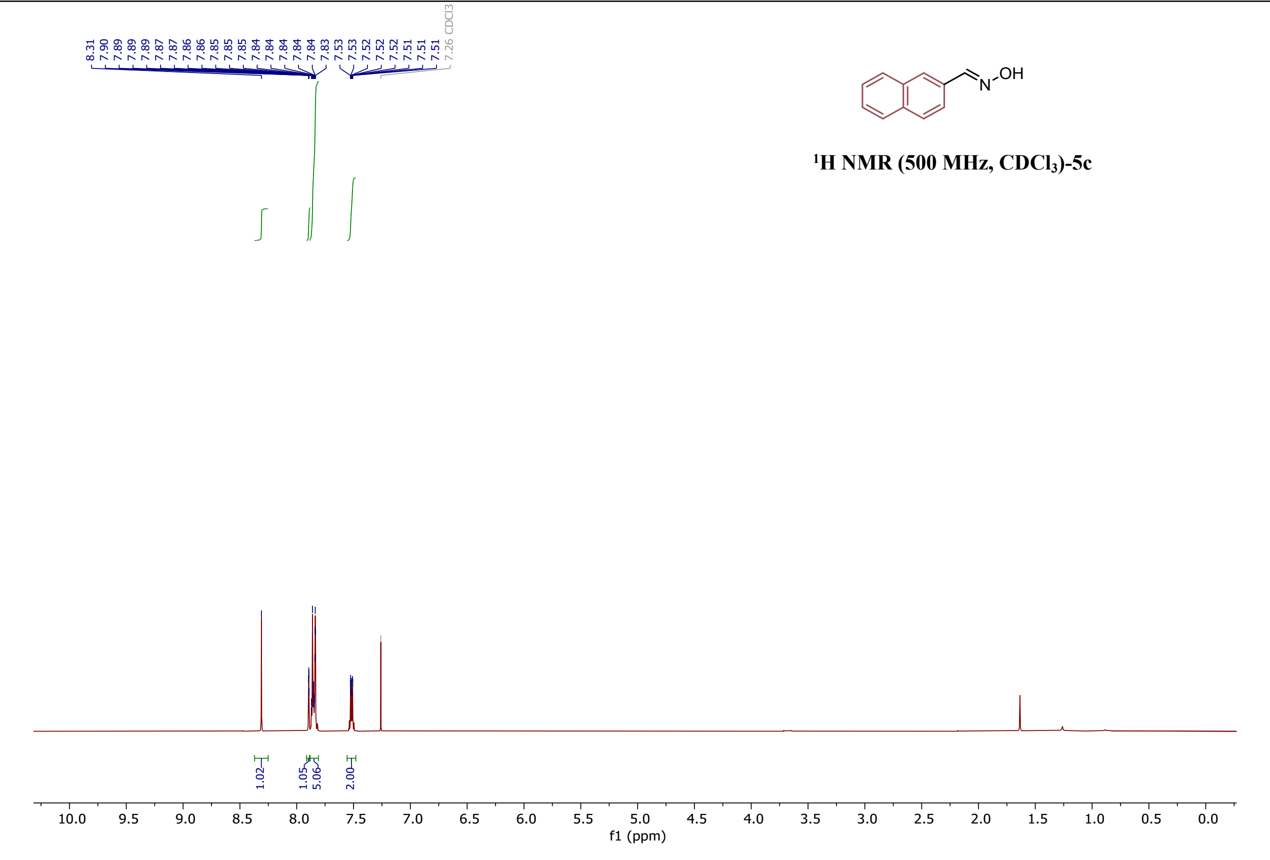

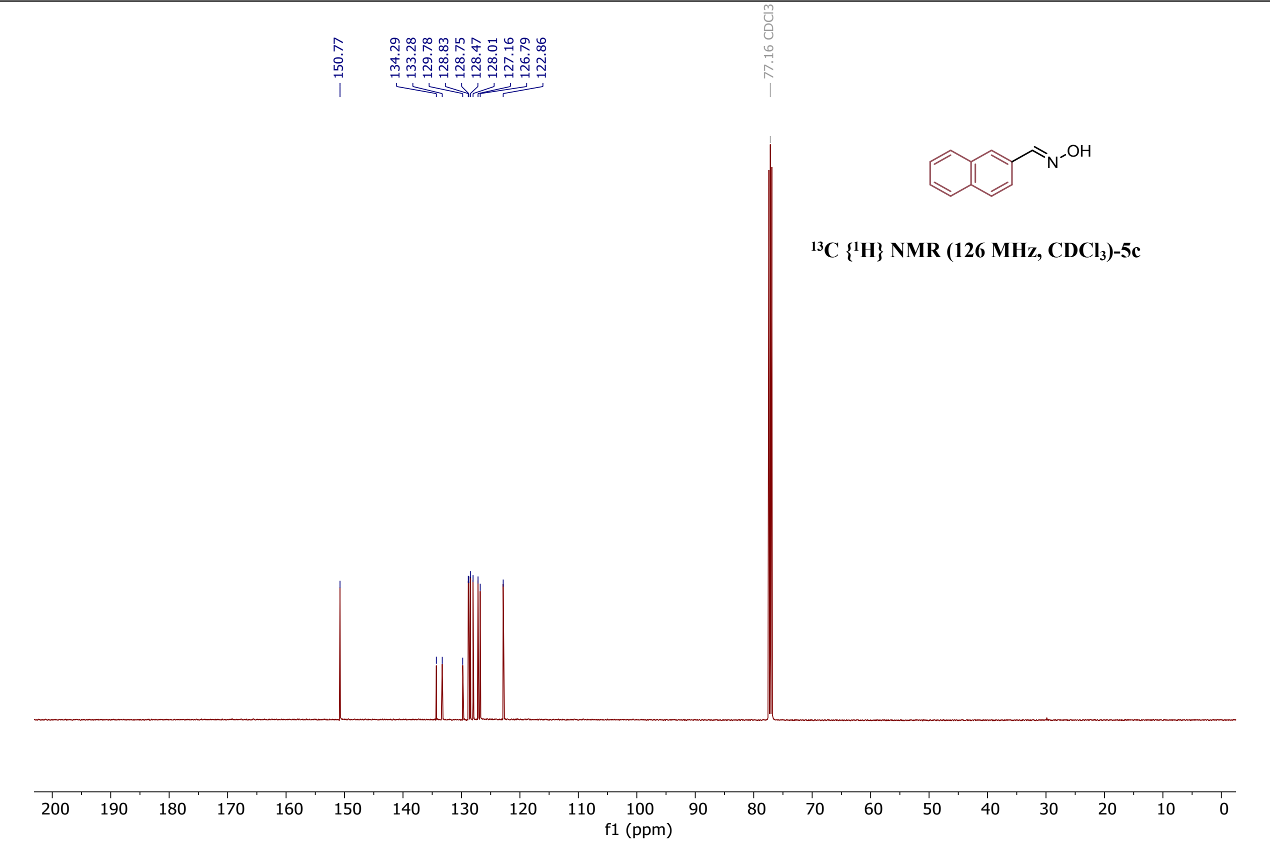

(*E*)-2-naphthaldehyde oxime ((5c)

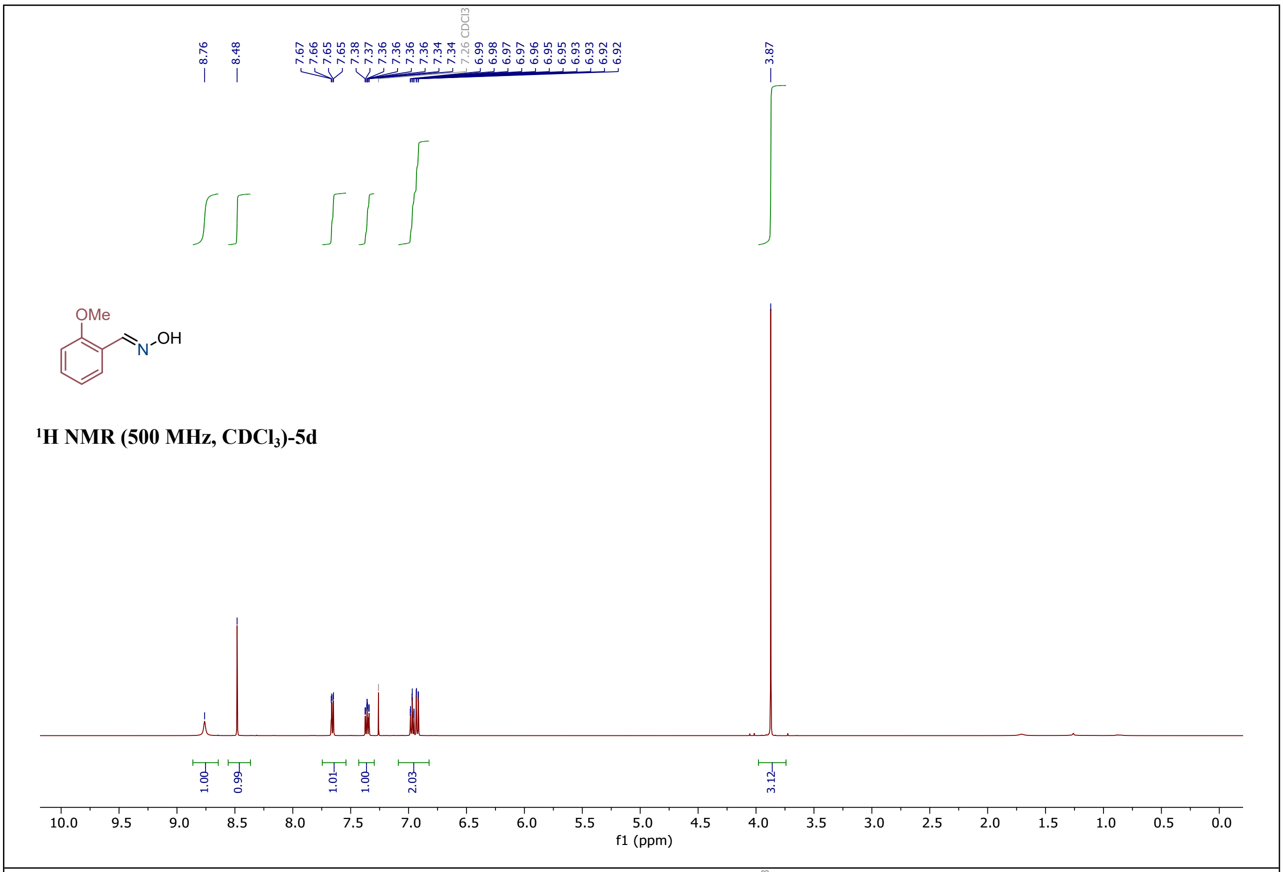

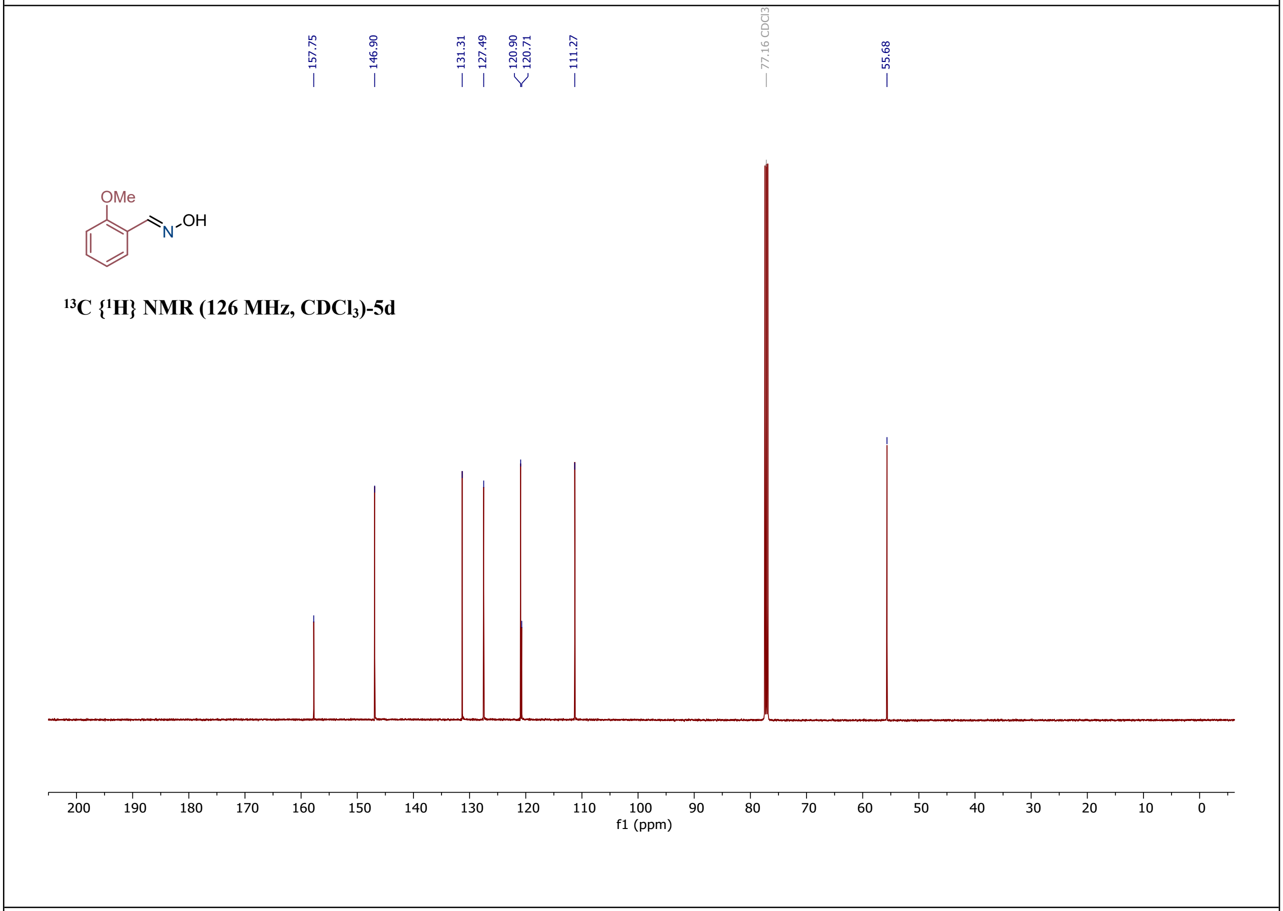

***(E)*-2-methoxybenzaldehyde oxime (5d)**

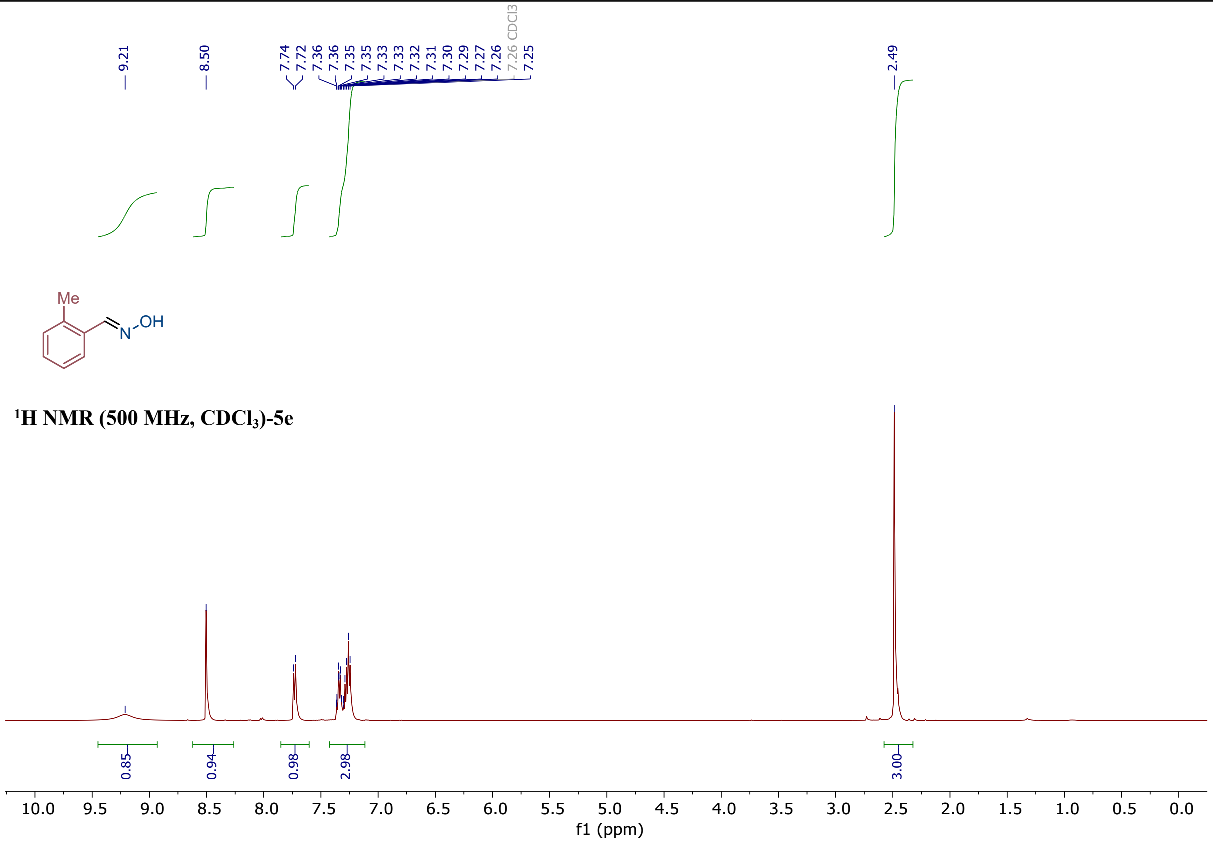

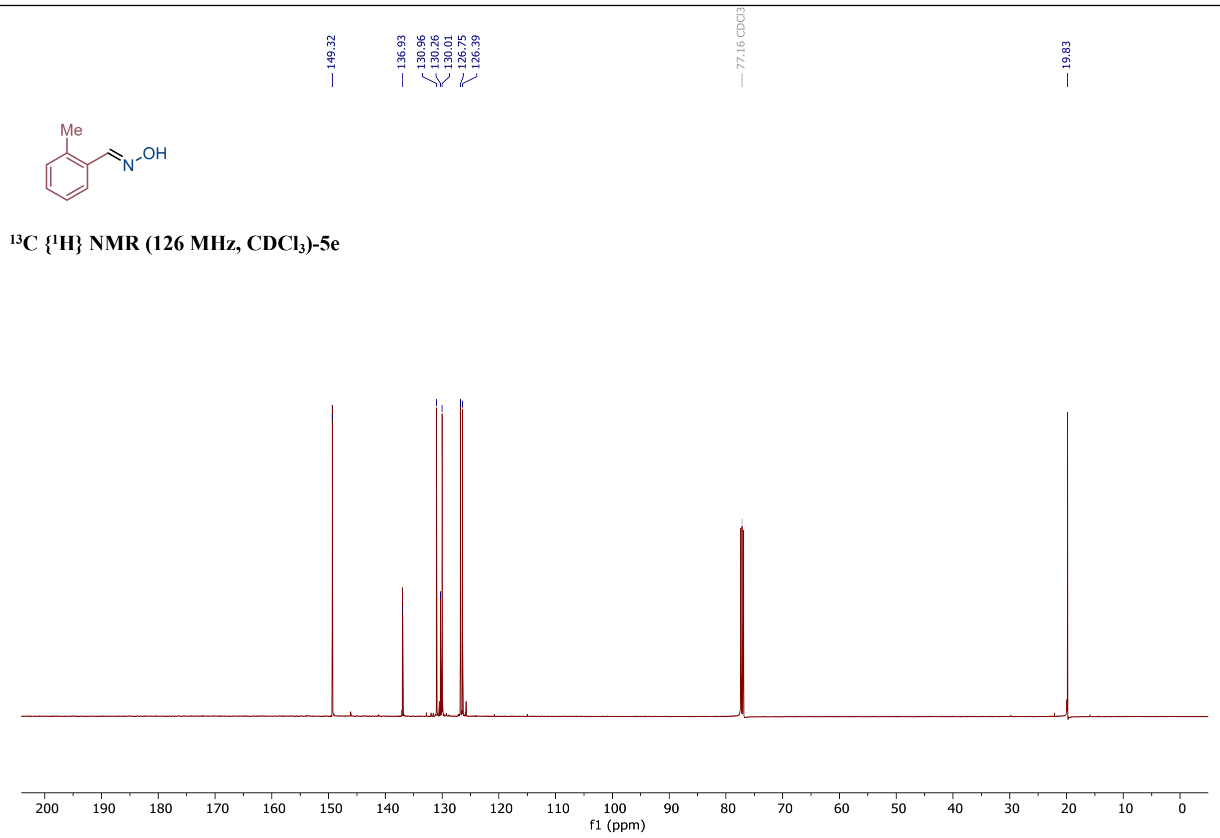

(*E*)-2-methylbenzaldehyde oxime (5e)

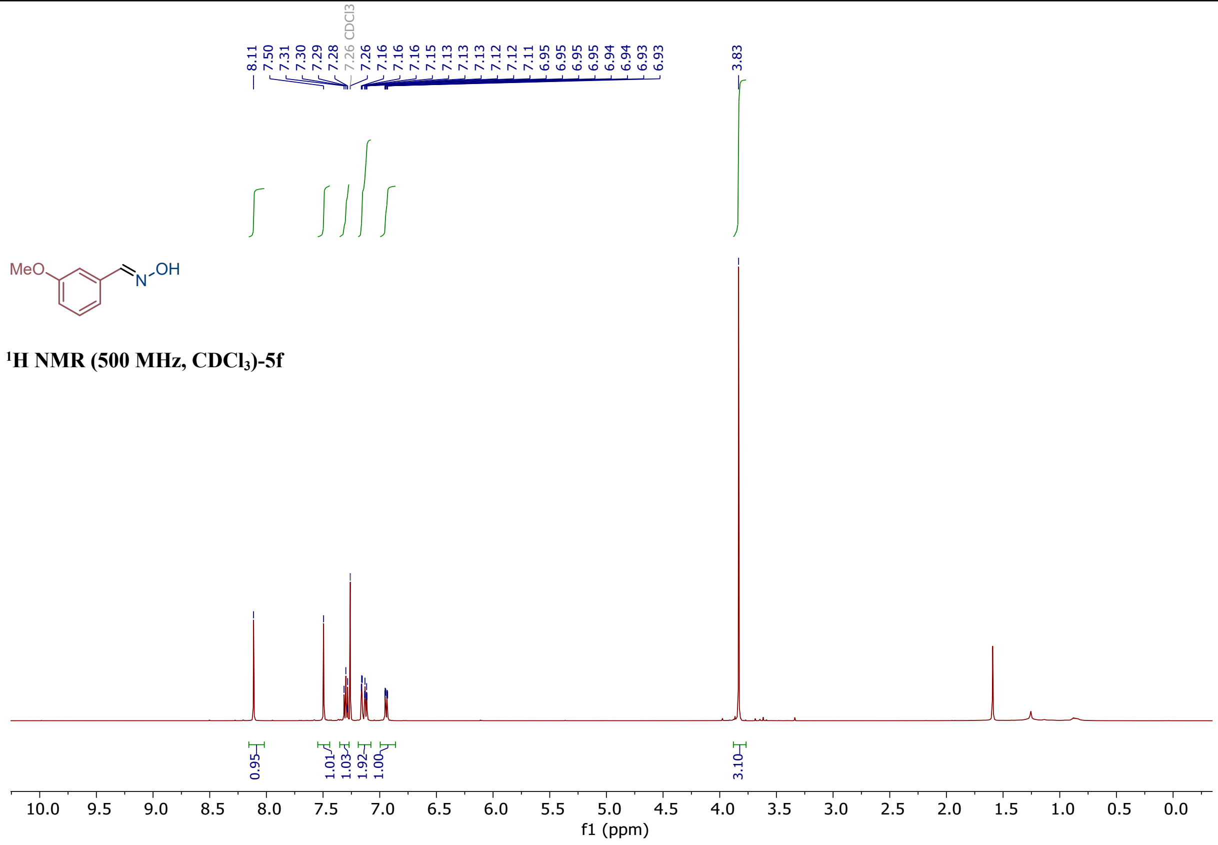

^{1}H NMR (500 MHz, $CDCl_3$)-5f

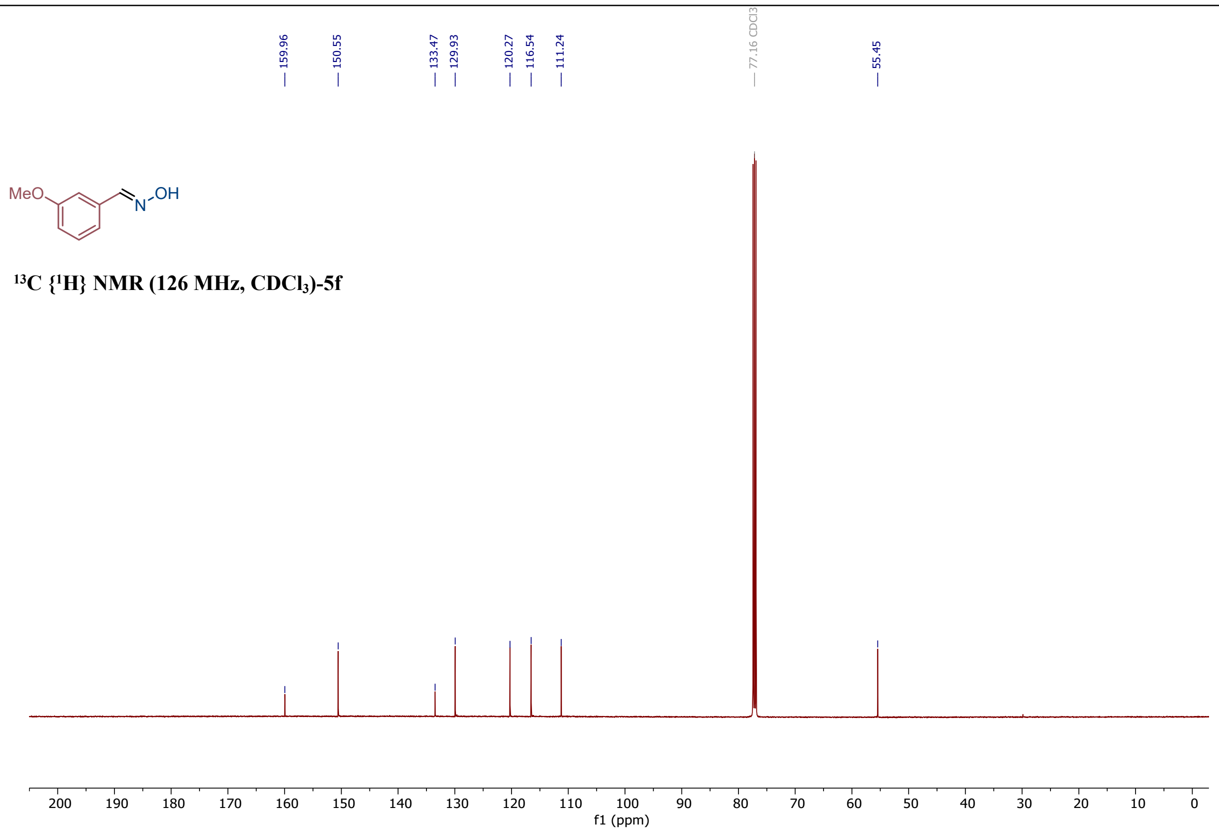

^{13}C {^{1}H} NMR (126 MHz, $CDCl_3$)-5f

(*E*)-3-methoxybenzaldehyde oxime (5f)

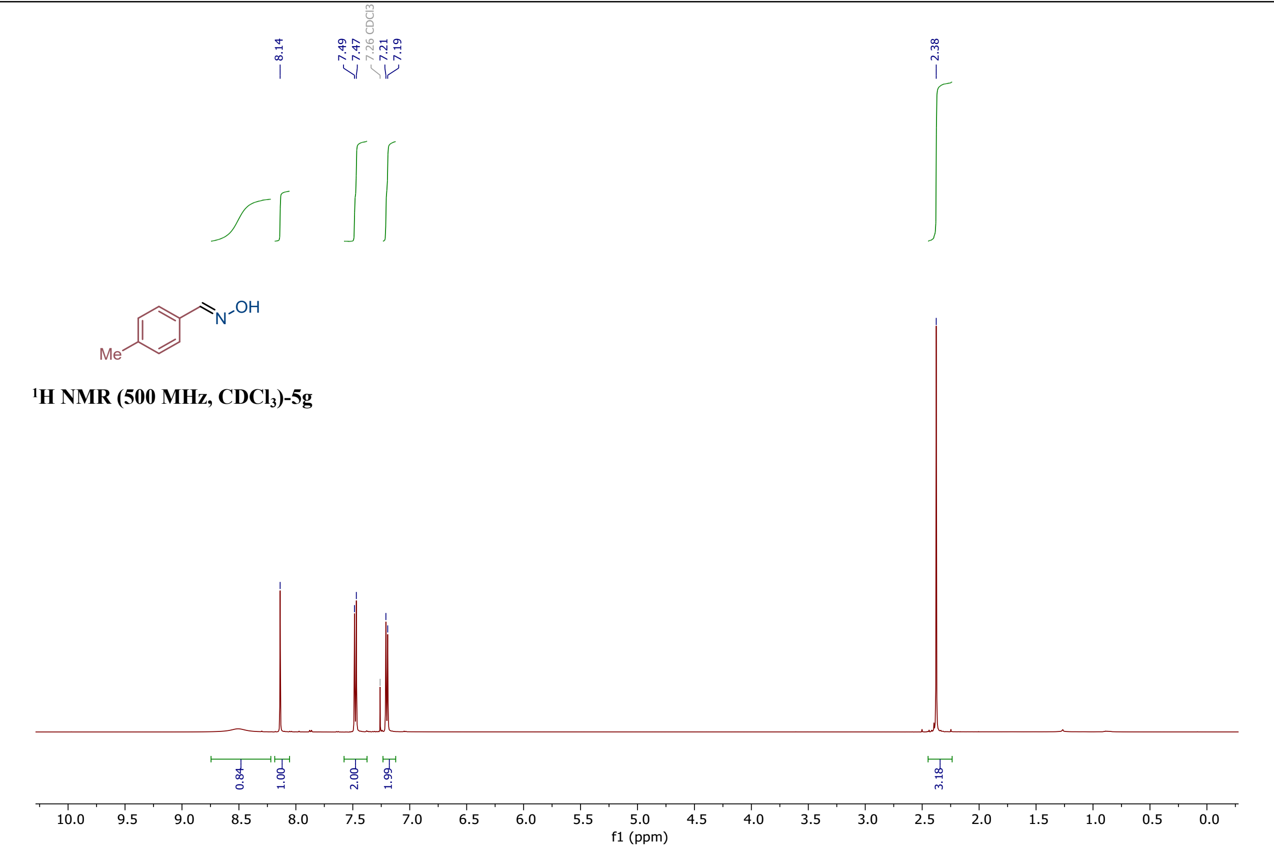

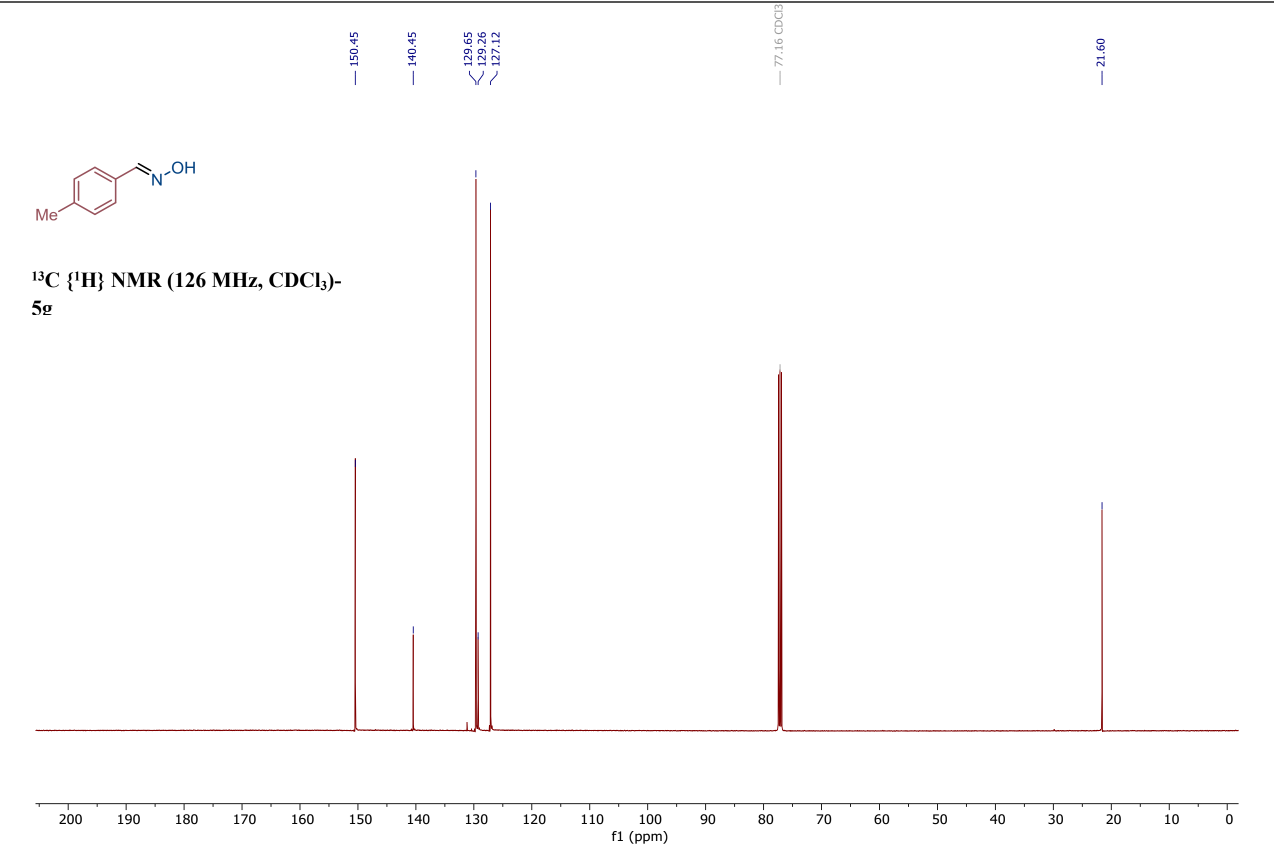

(*E*)-4-methylbenzaldehyde oxime(5g)

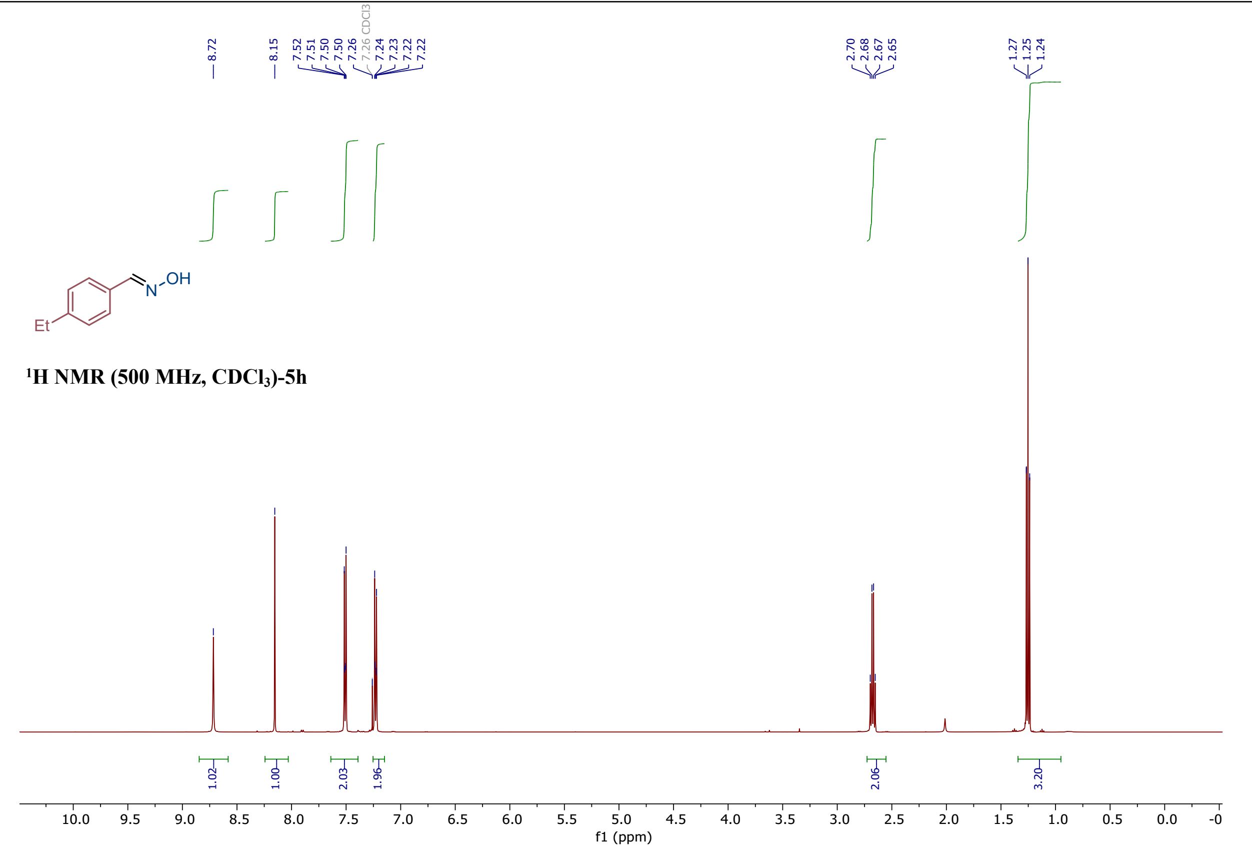

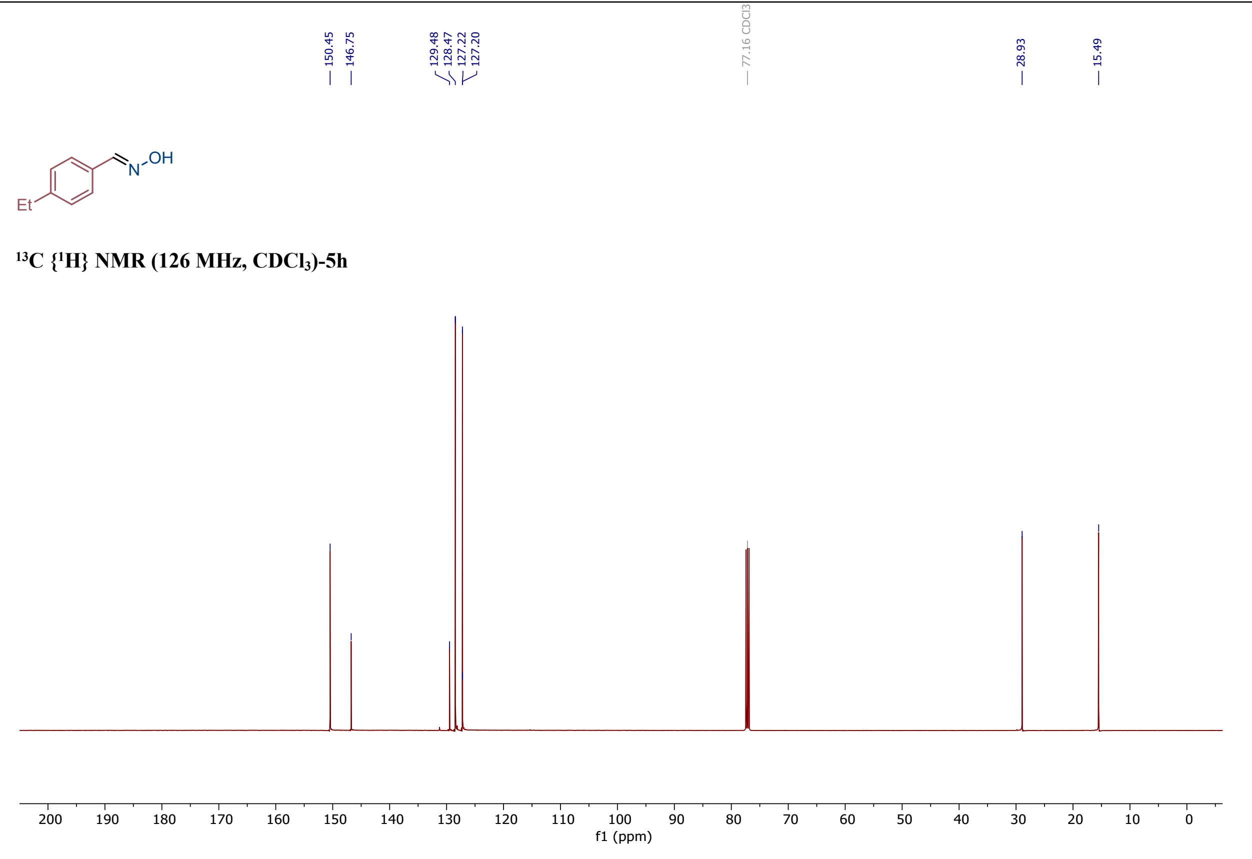

(*E*)-4-ethylbenzaldehyde oxime (5h)

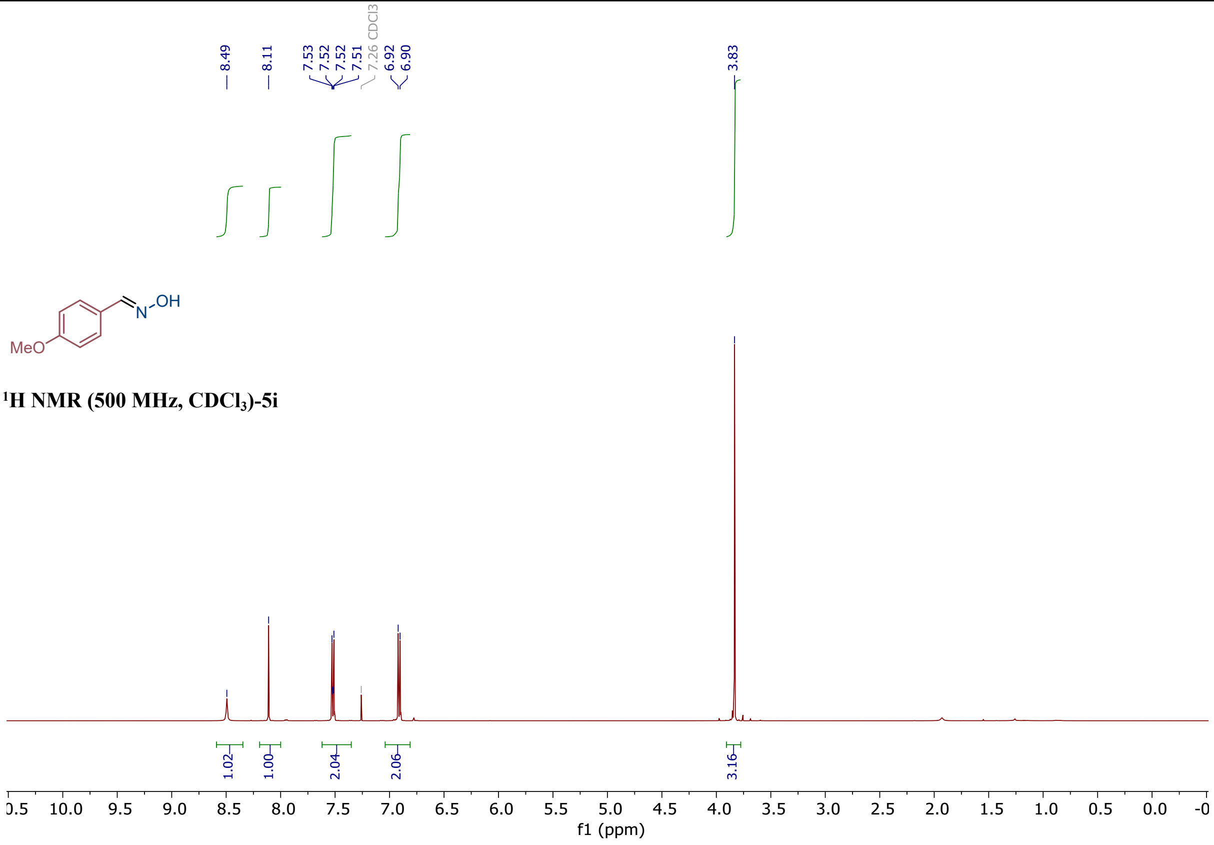

^{1}H NMR (500 MHz, $CDCl_3$)-5i

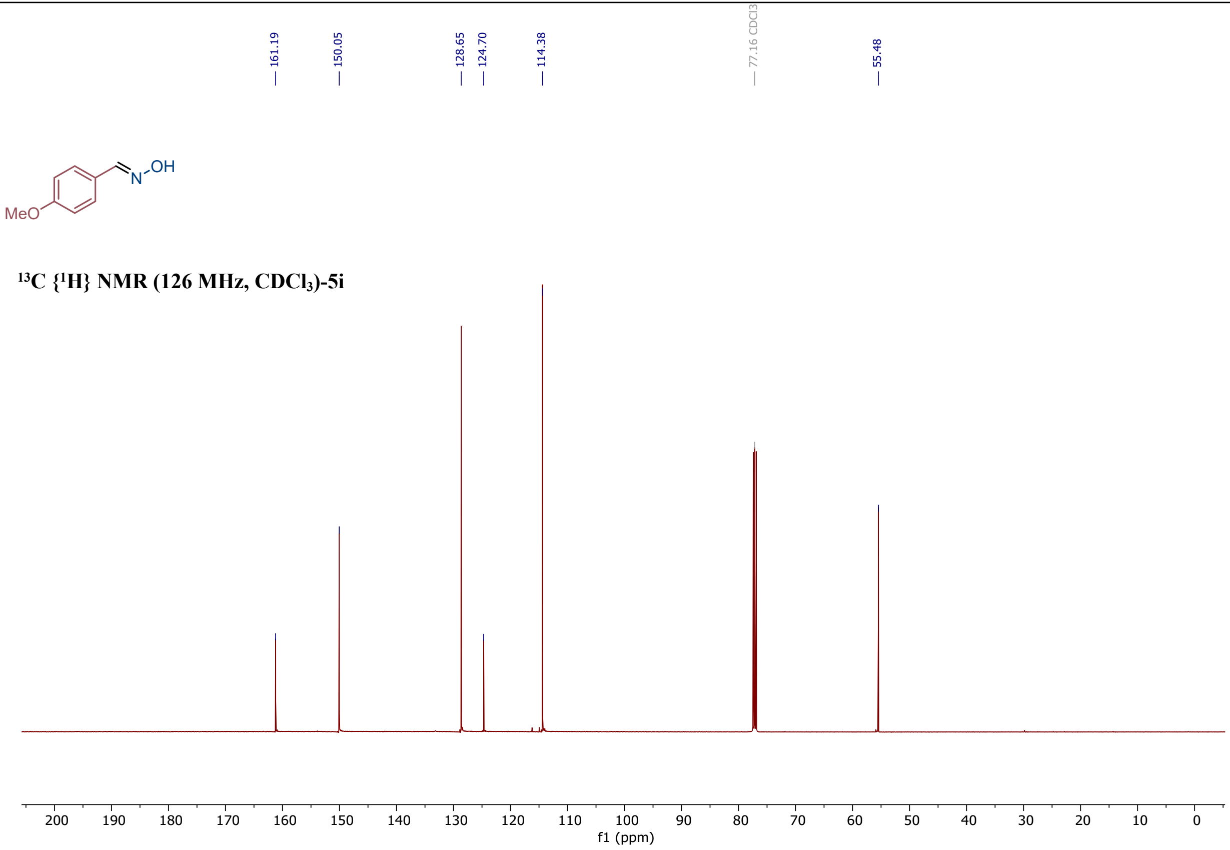

^{13}C {^{1}H} NMR (126 MHz, $CDCl_3$)-5i

(*E*)-4-methoxybenzaldehyde oxime(5i)

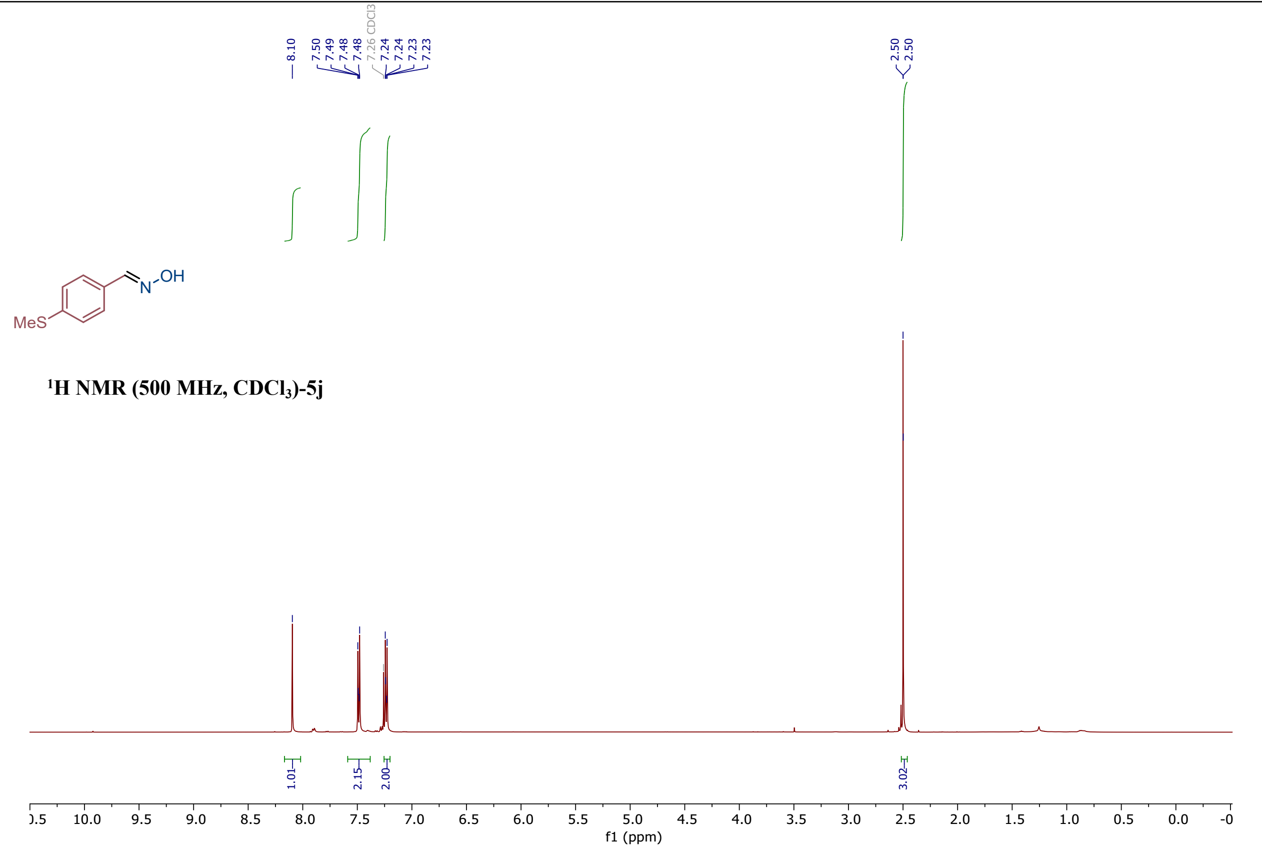

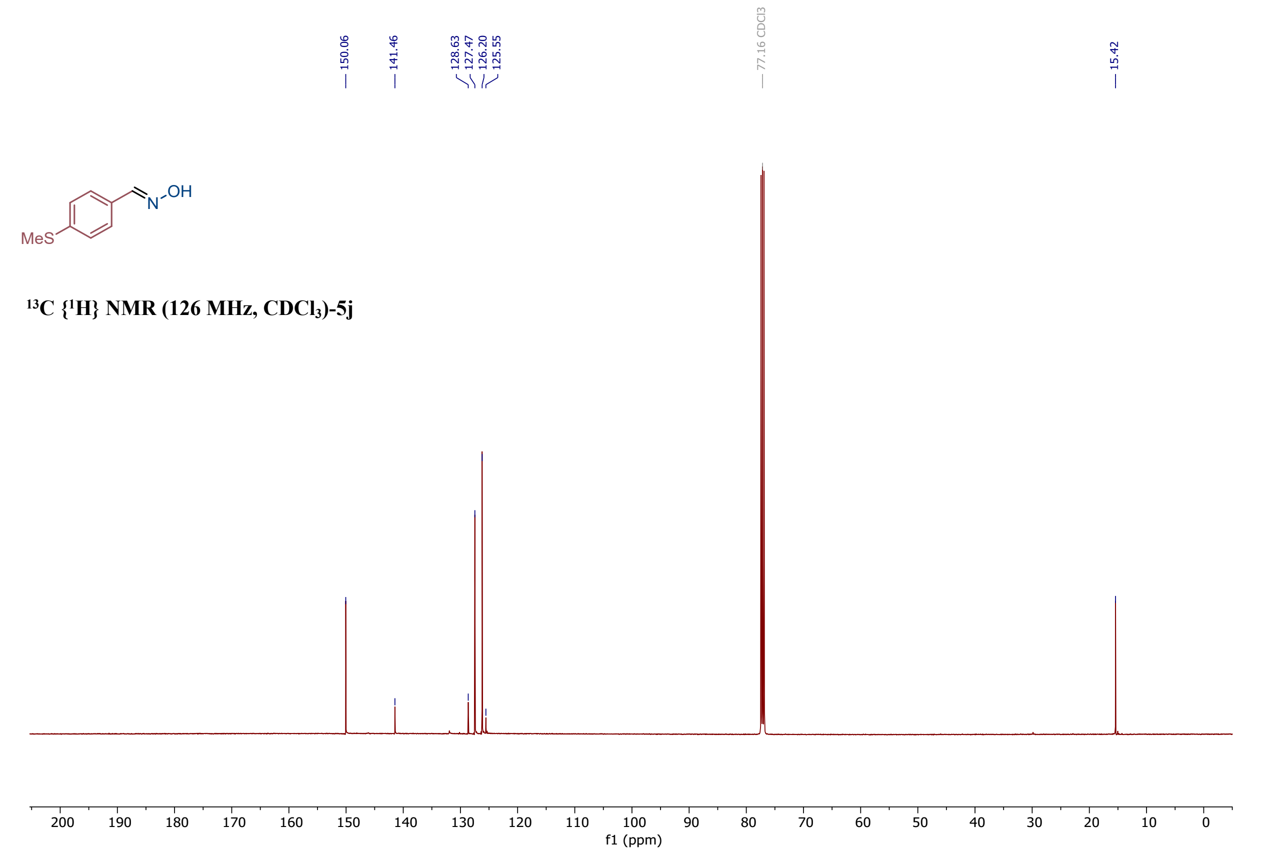

(*E*)-4-(methylthio)benzaldehyde oxime (5j)

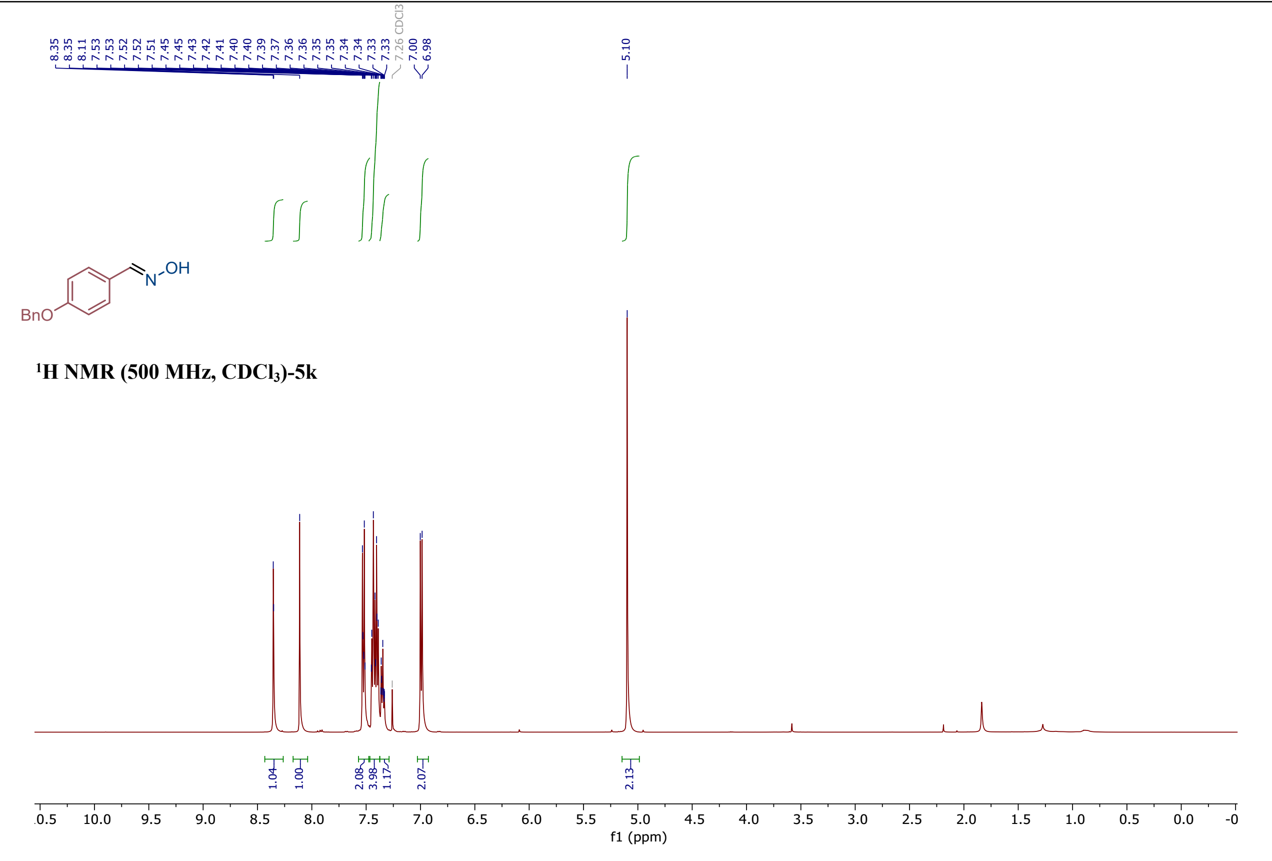

^{1}H NMR (500 MHz, $CDCl_3$)-5k

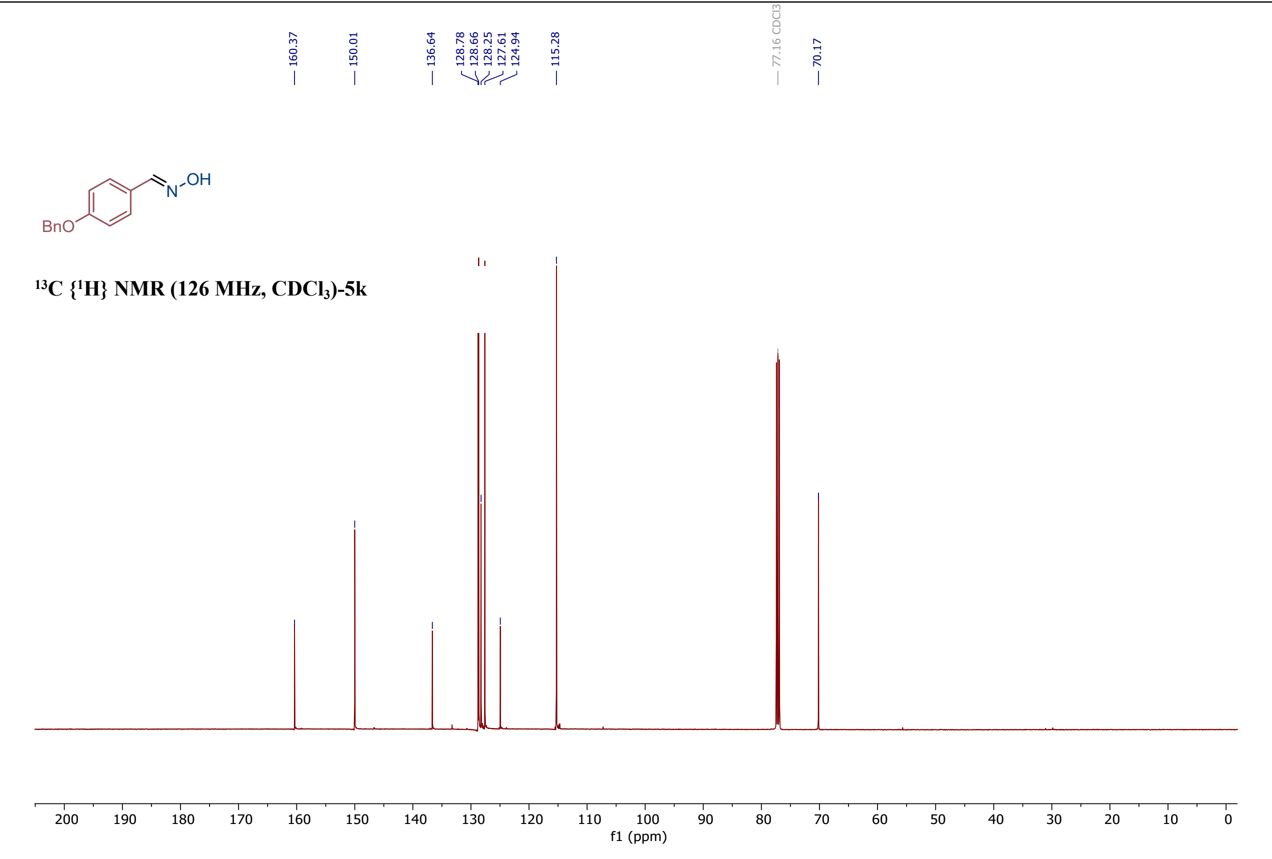

^{13}C {^{1}H} NMR (126 MHz, $CDCl_3$)-5k

(*E*)-4-(benzyloxy)benzaldehyde oxime(5k)

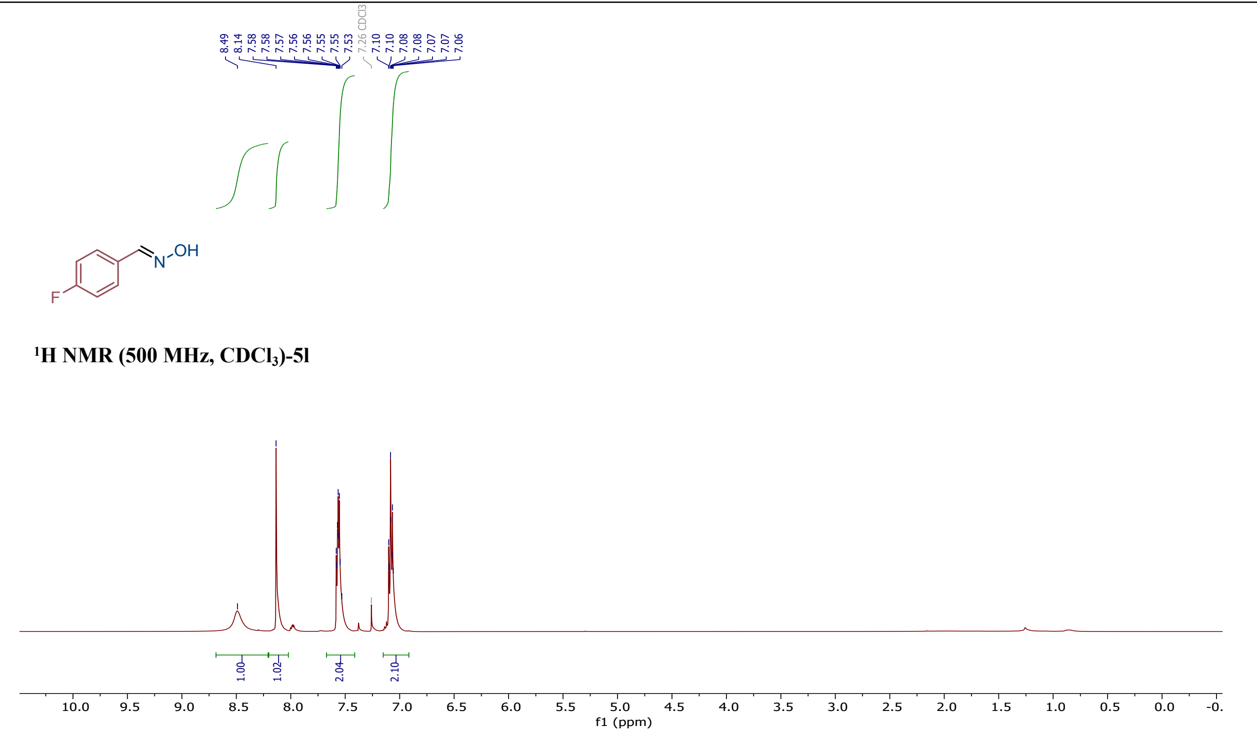

^{1}H NMR (500 MHz, CDCl$_3$)-5l

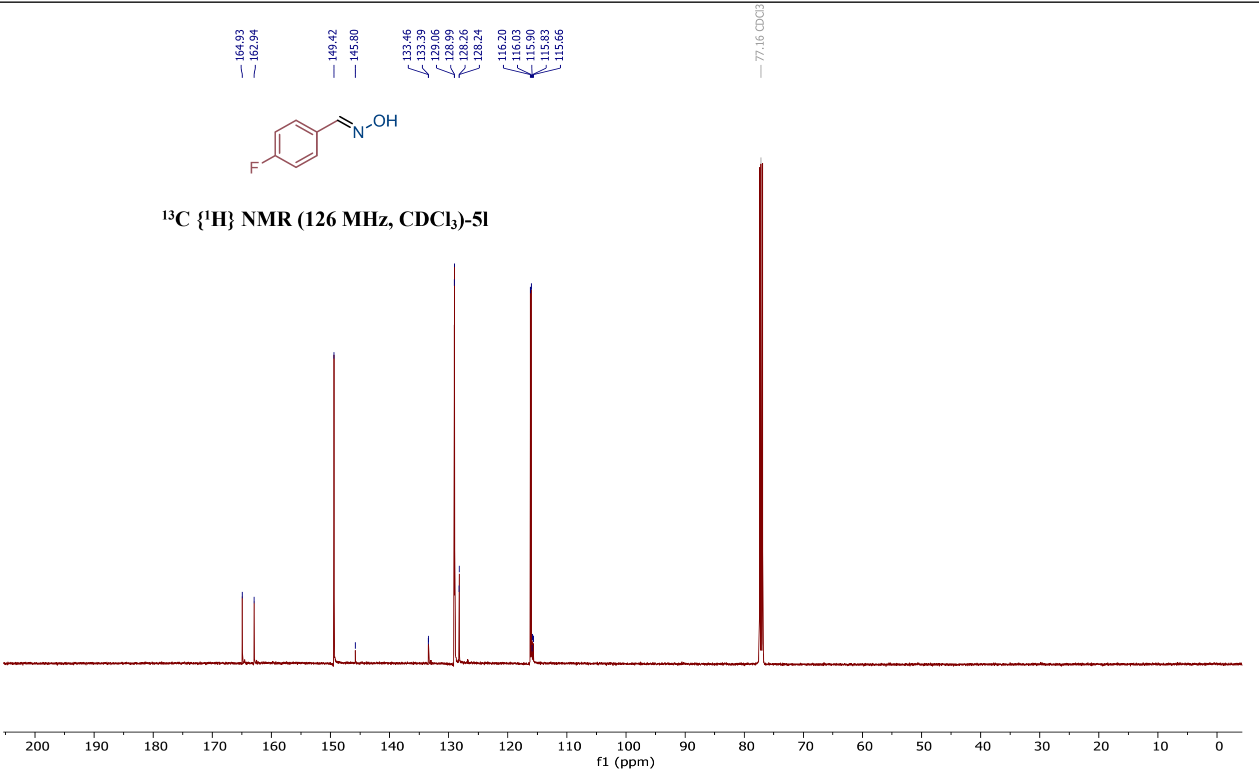

^{13}C {^{1}H} NMR (126 MHz, CDCl$_3$)-5l

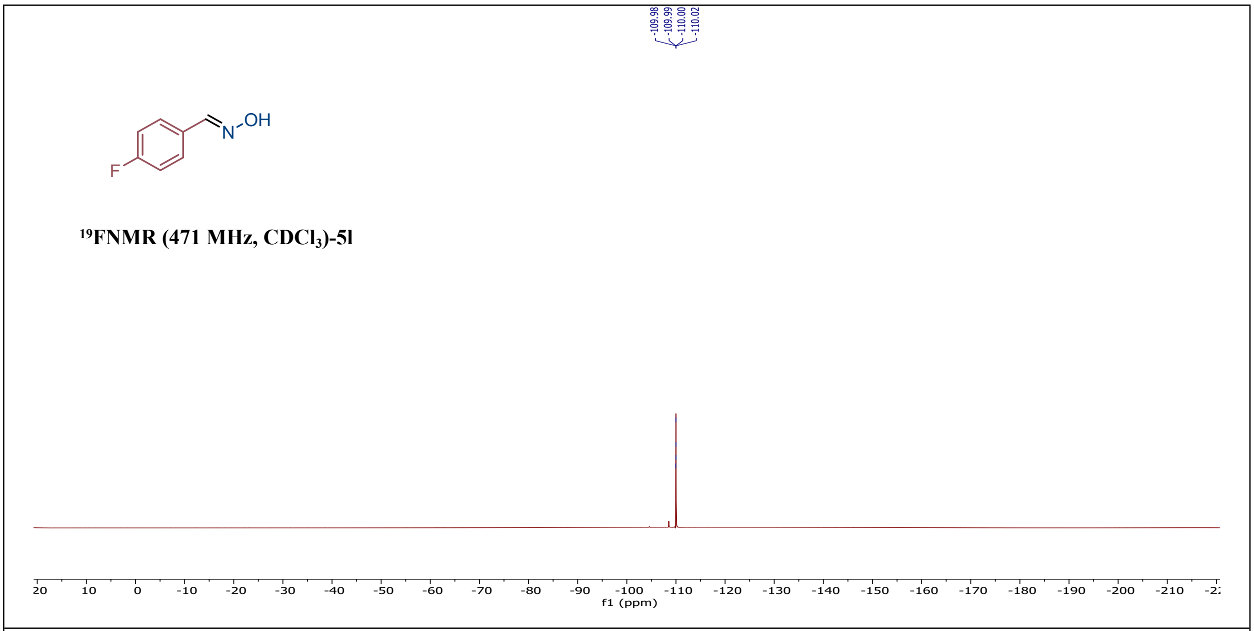

(*E*)-4-fluorobenzaldehyde oxime (5l)[4,6]

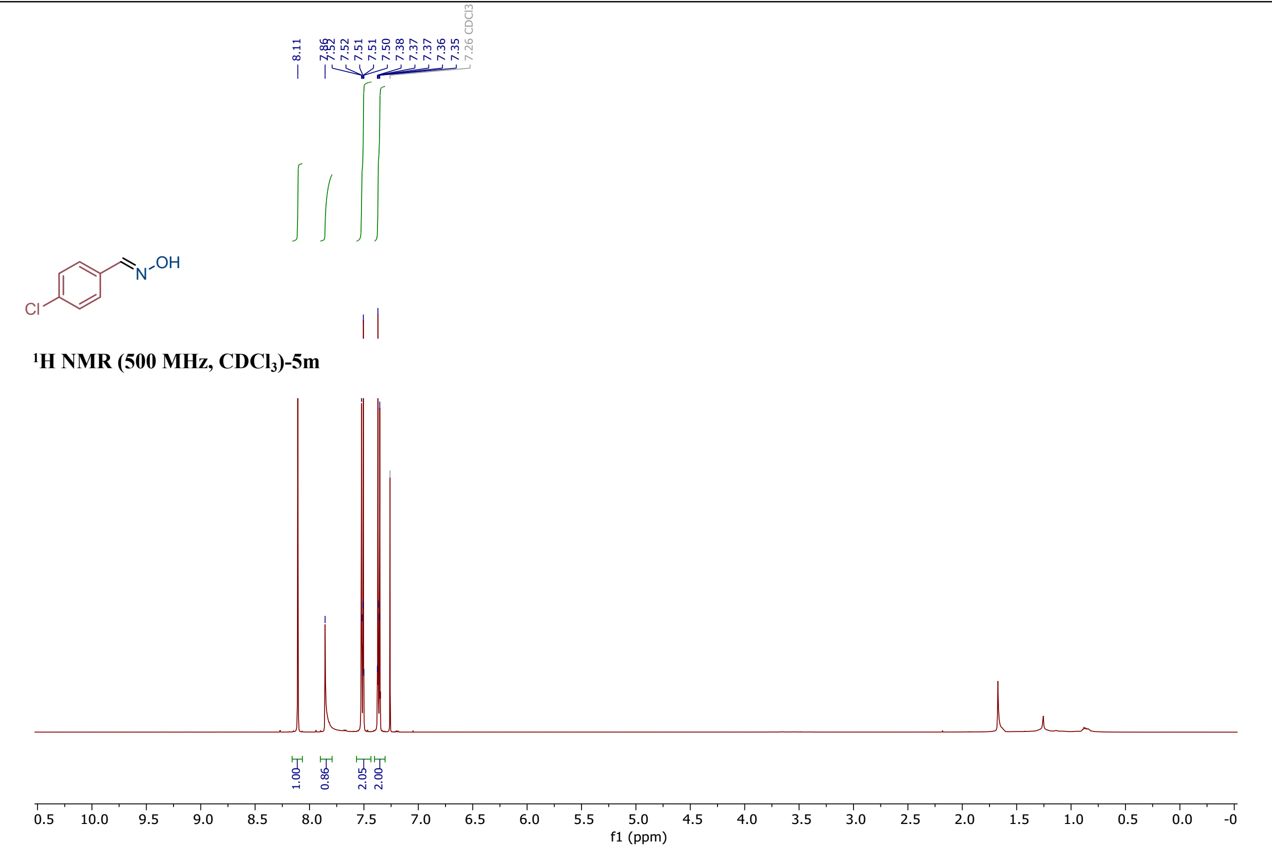

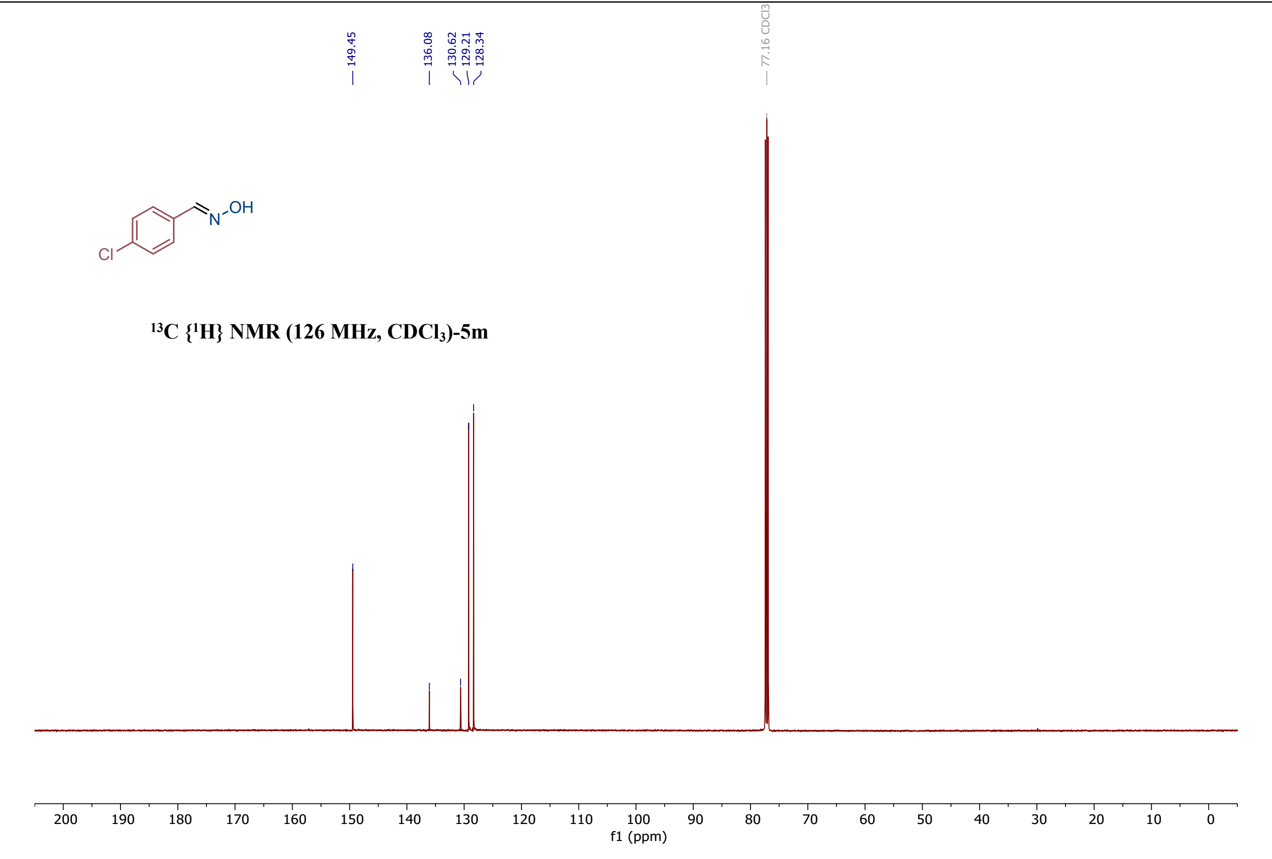

(*E*)-4-chlorobenzaldehyde oxime (5m)

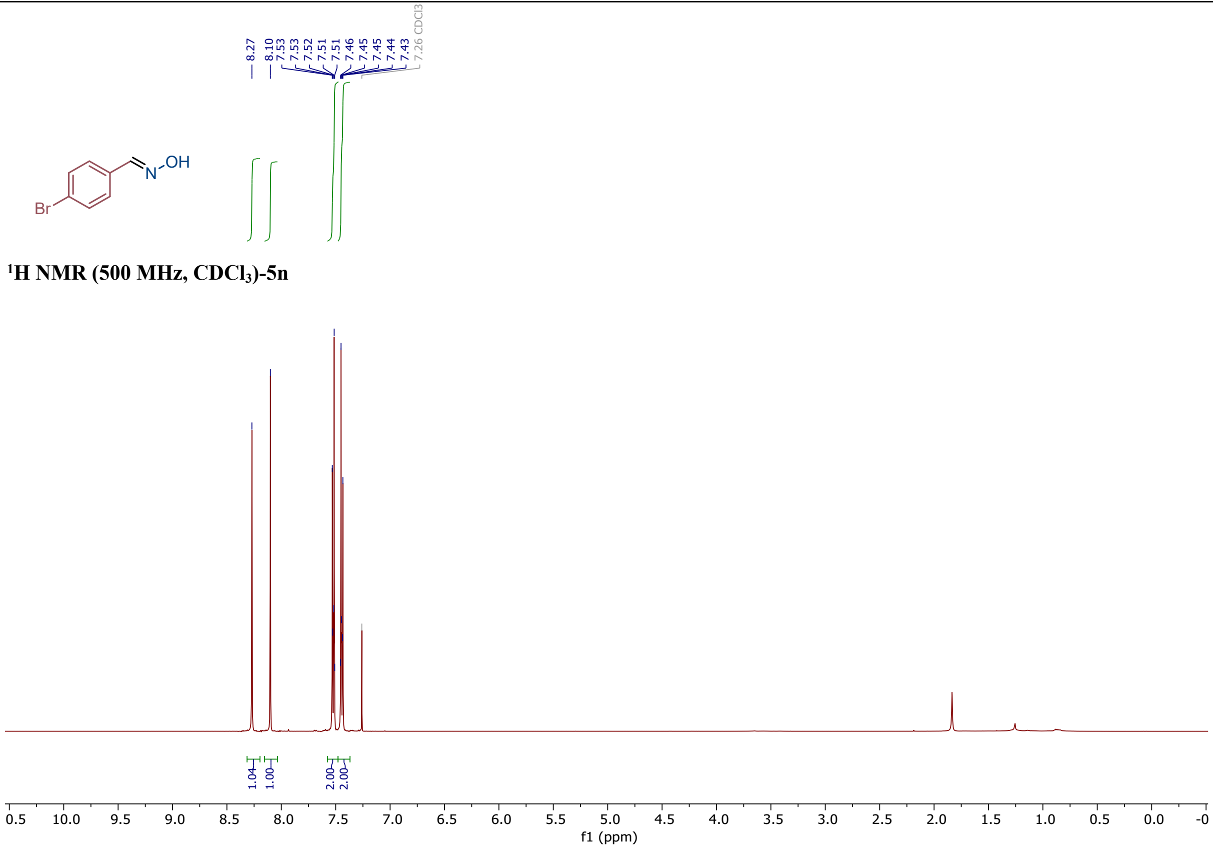

^{1}H NMR (500 MHz, $CDCl_3$)-5n

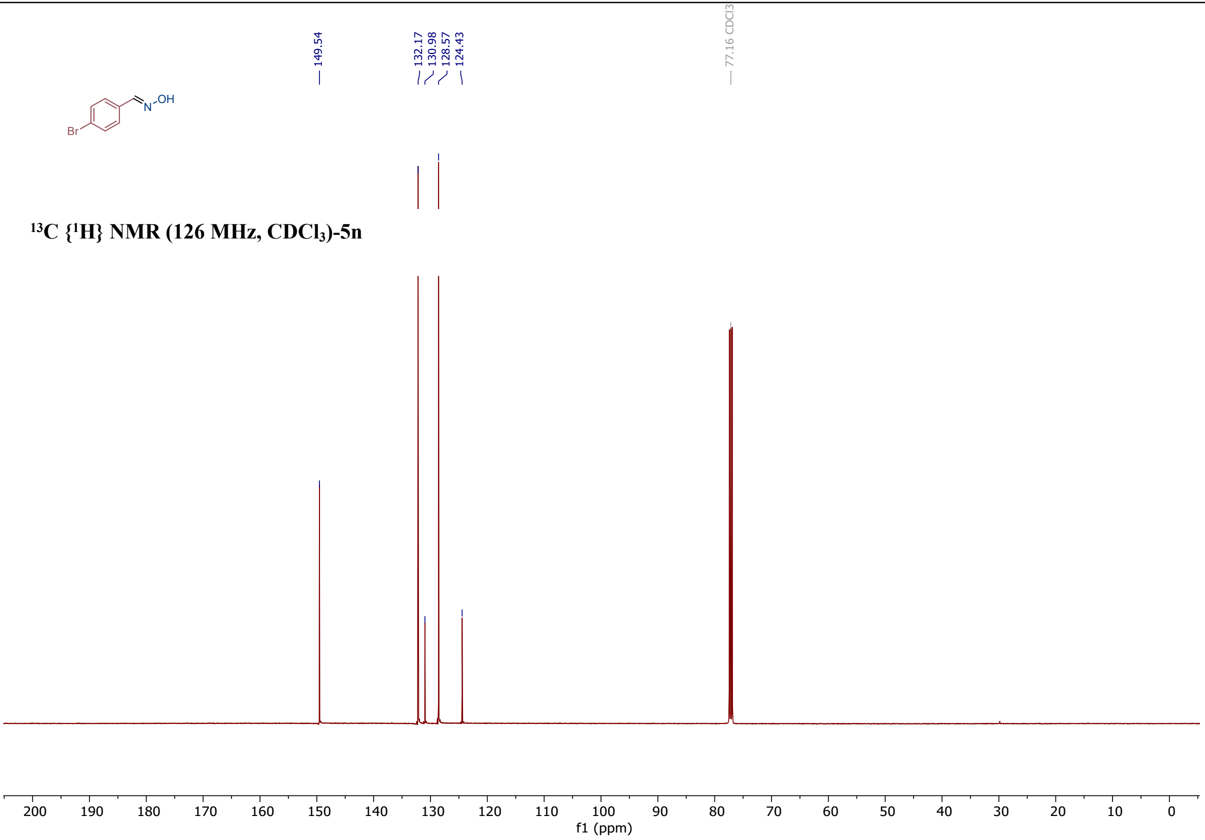

^{13}C {^{1}H} NMR (126 MHz, $CDCl_3$)-5n

(*E*)-4-bromobenzaldehyde oxime (5n)

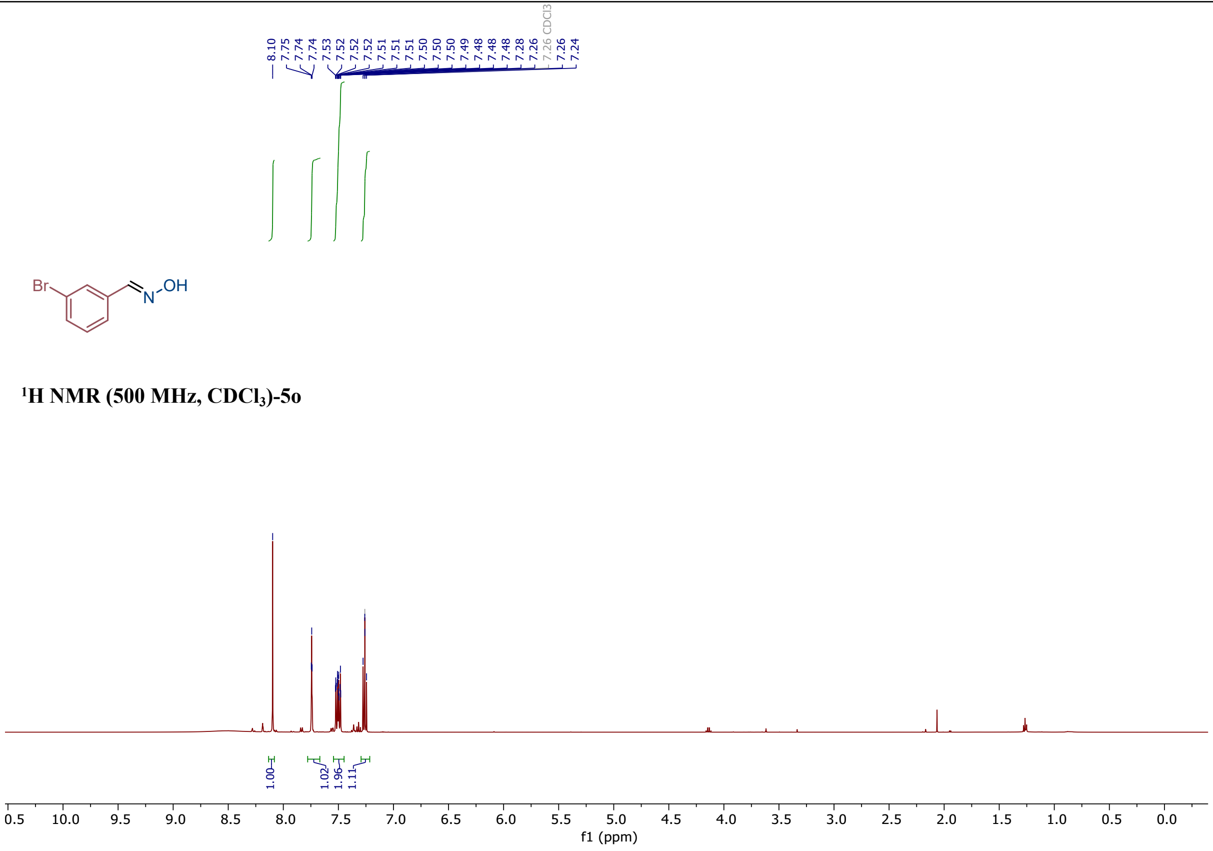

^{1}H NMR (500 MHz, $CDCl_3$)-5o

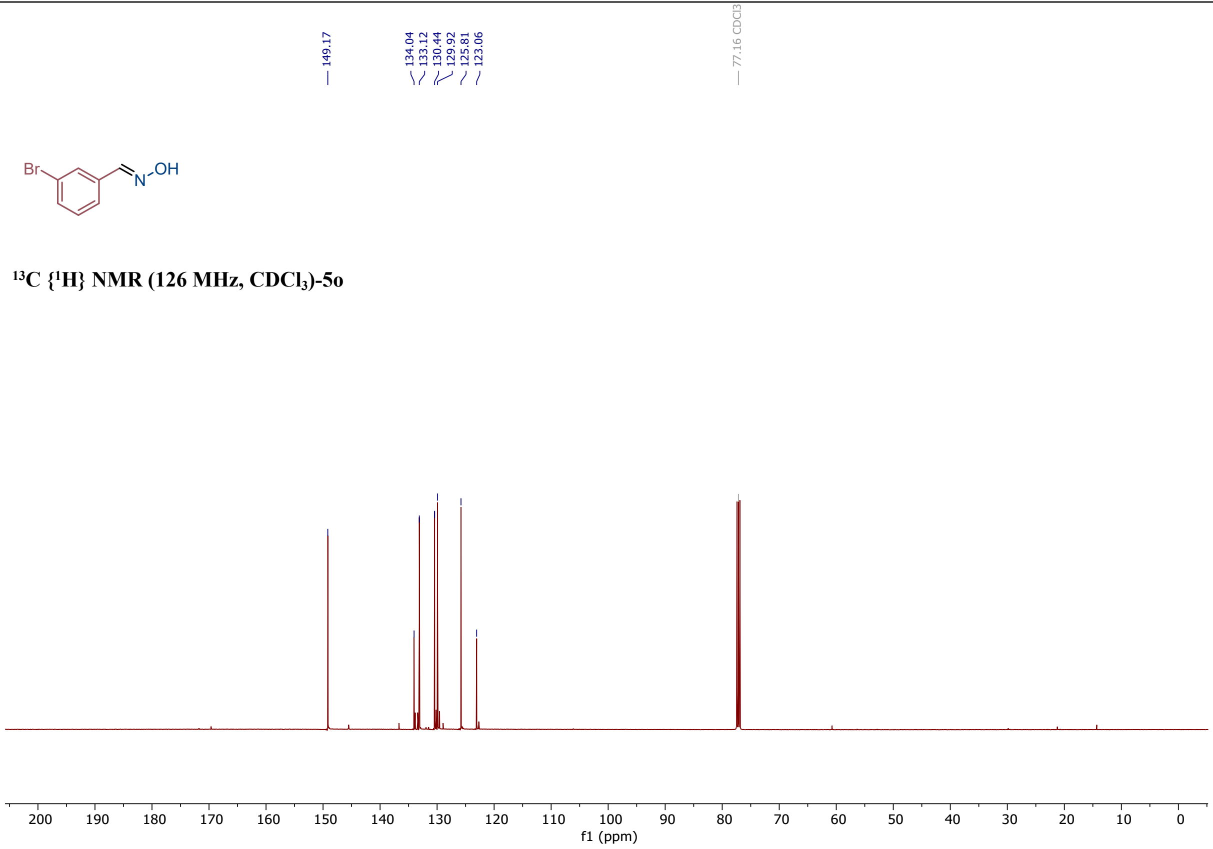

^{13}C {^{1}H} NMR (126 MHz, $CDCl_3$)-5o

(*E*)-3-bromobenzaldehyde oxime (5o)[5,7]

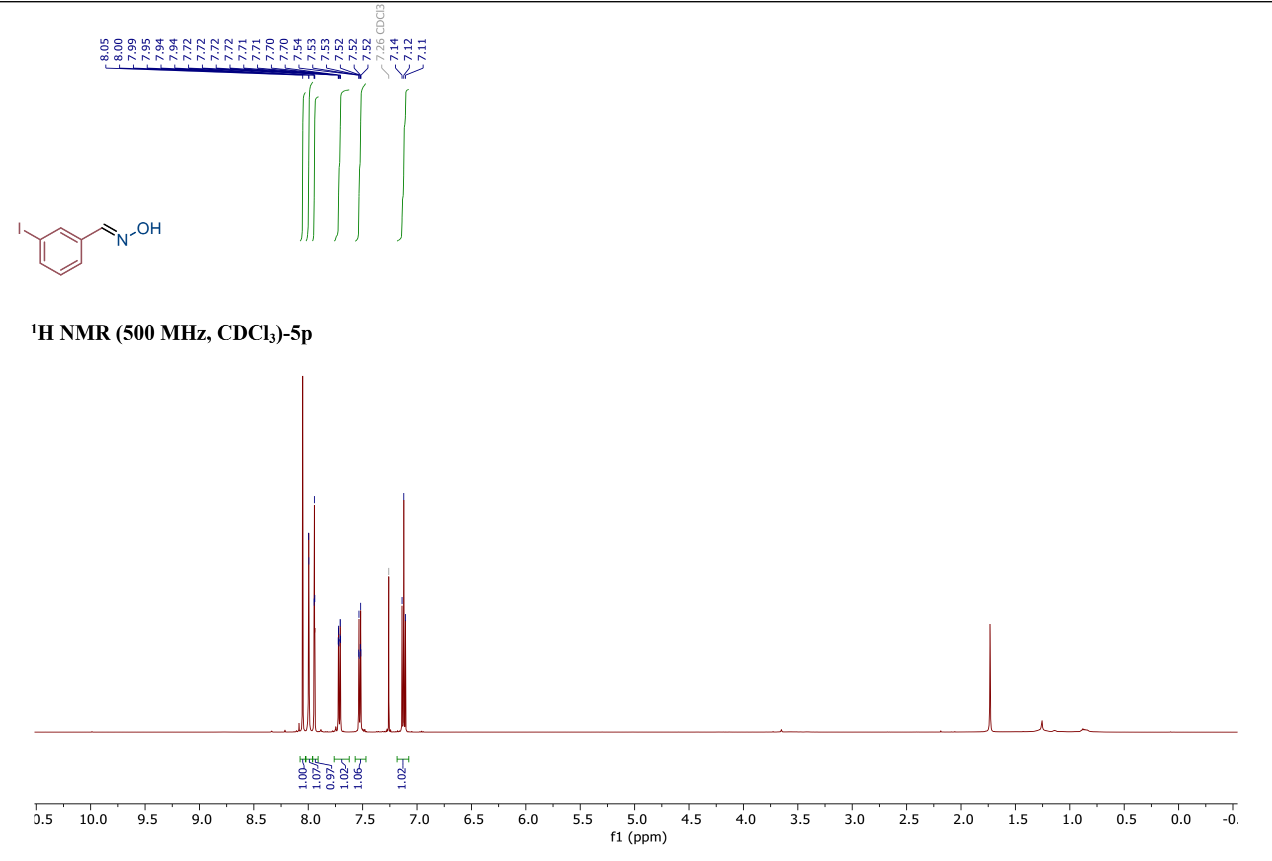

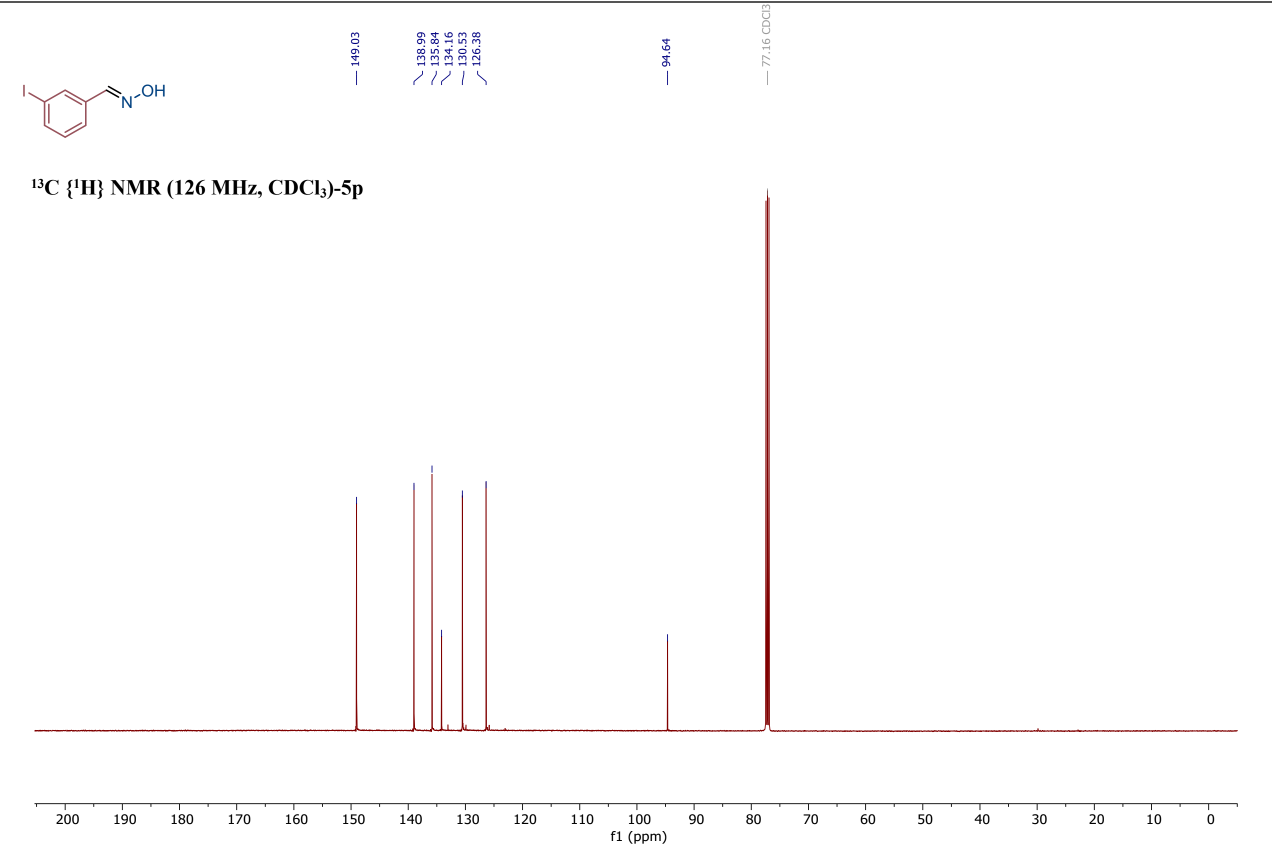

(*E*)-3-iodobenzaldehyde oxime (5p)[8]

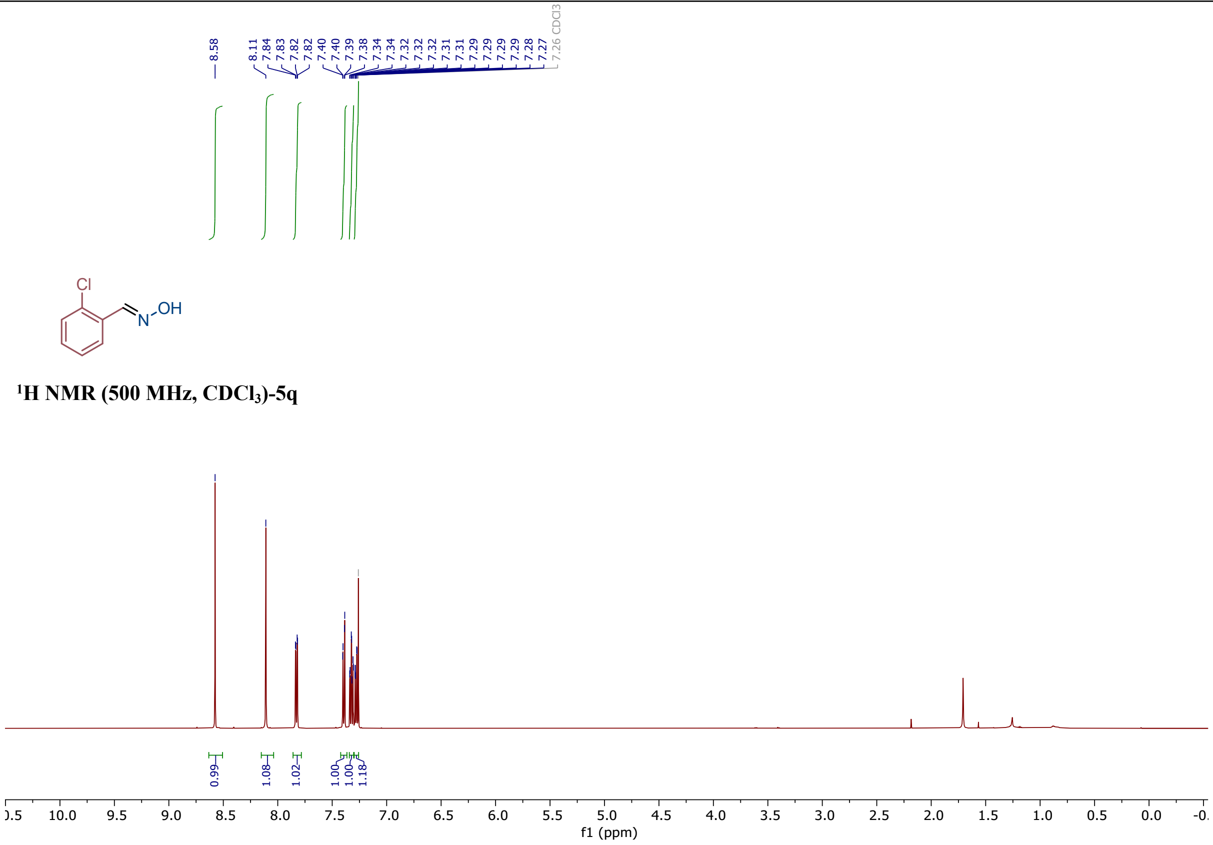

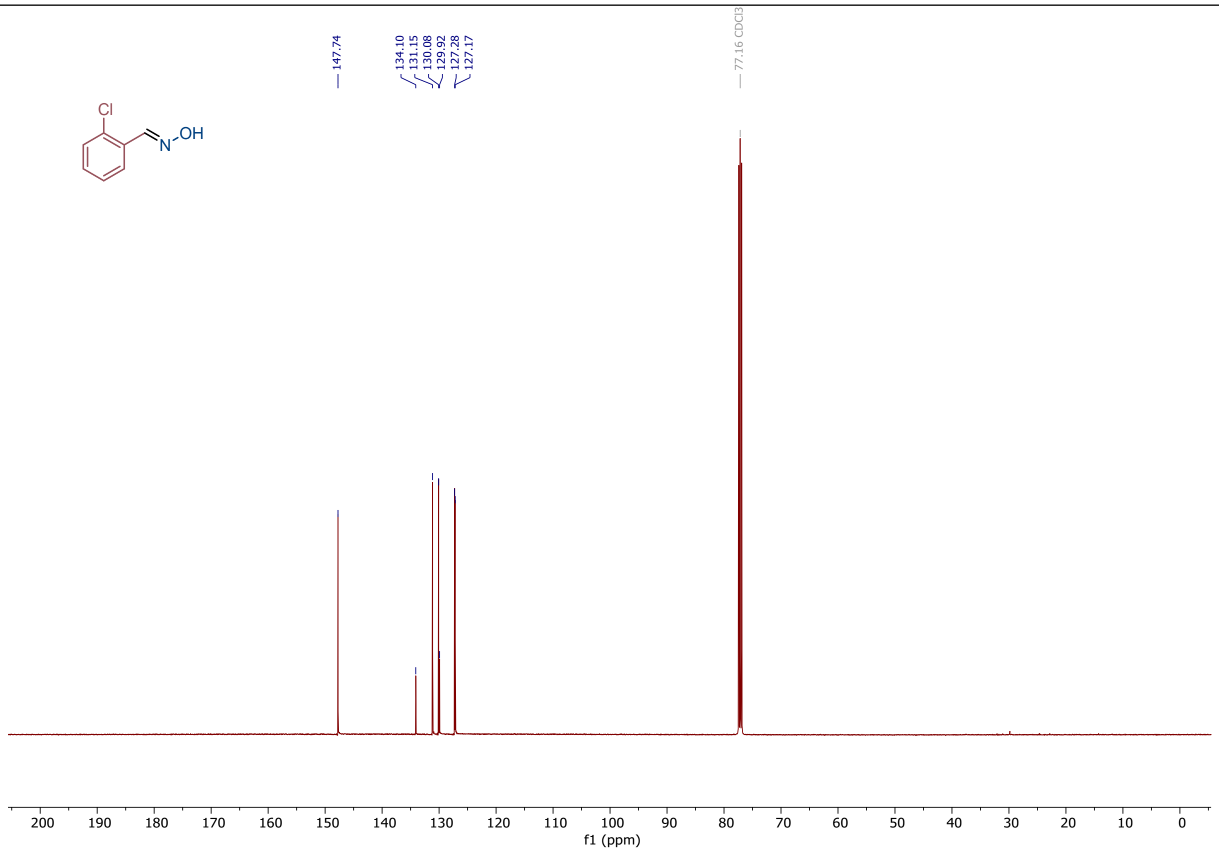

(*E*)-2-chlorobenzaldehyde oxime (5q)[4,5,11]

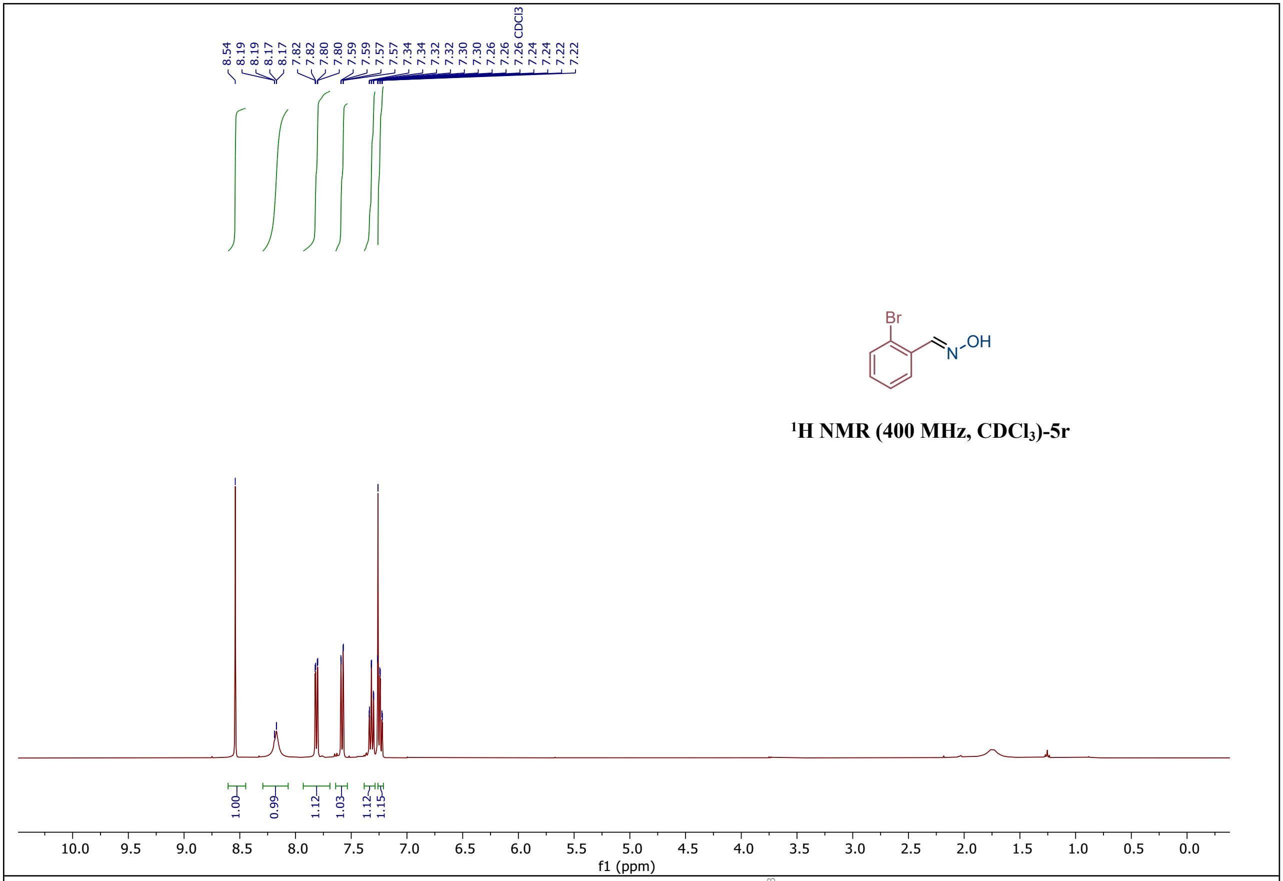

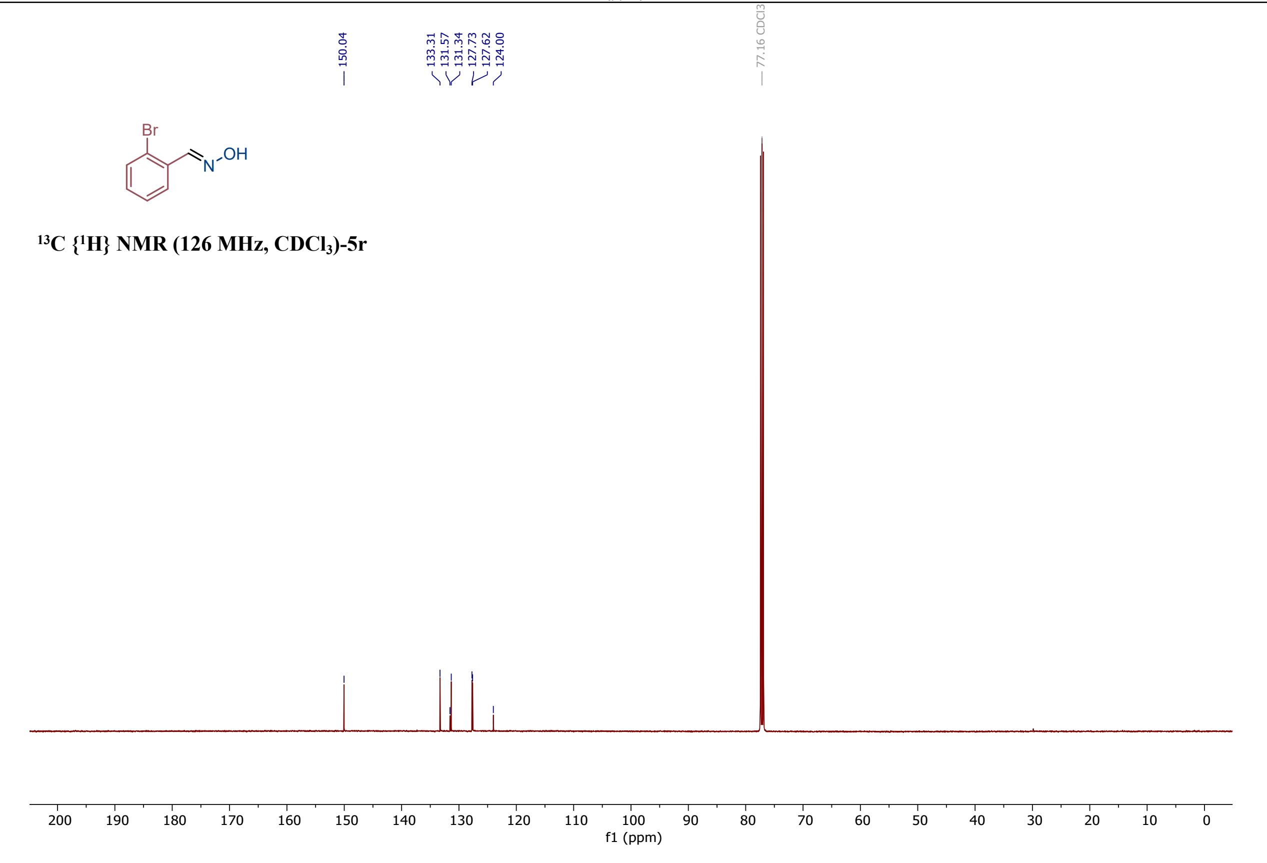

(*E*)-2-bromobenzaldehyde oxime (5r)[1,9]

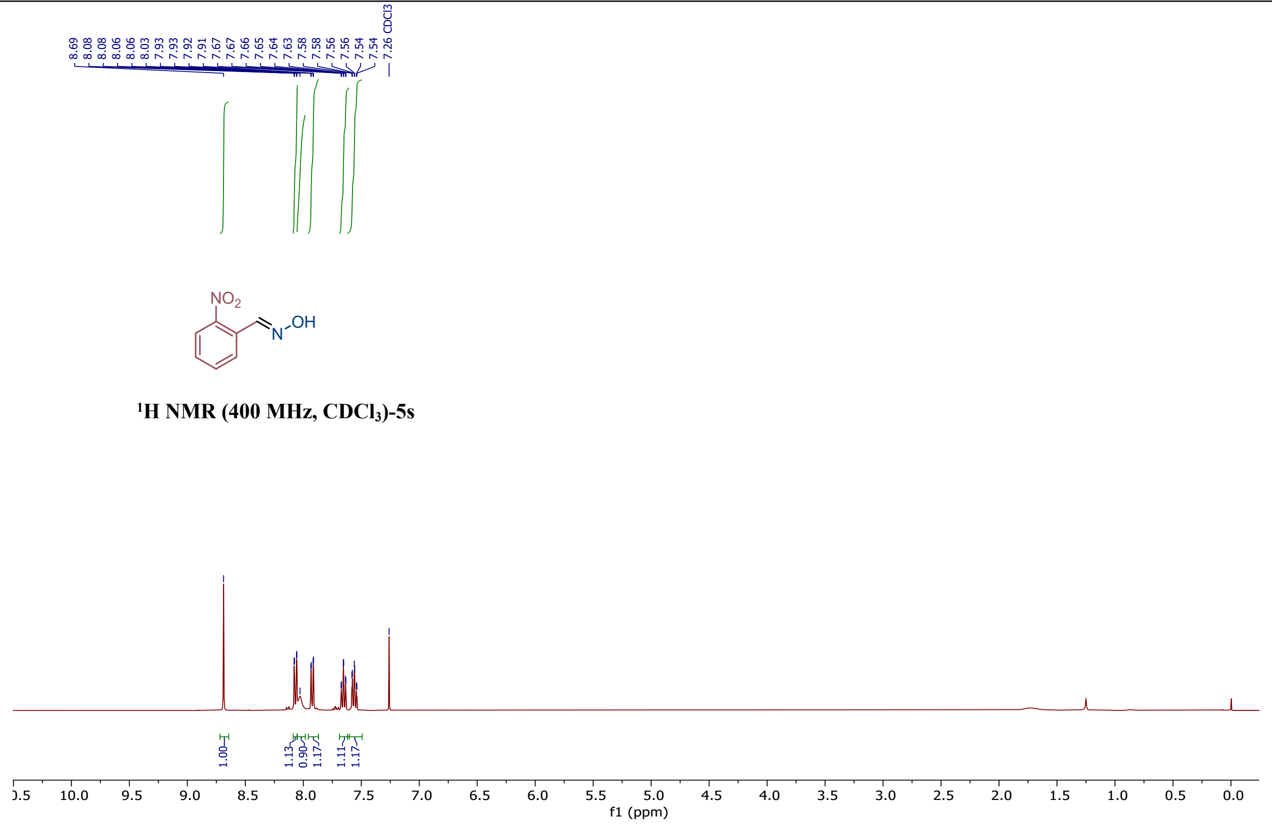

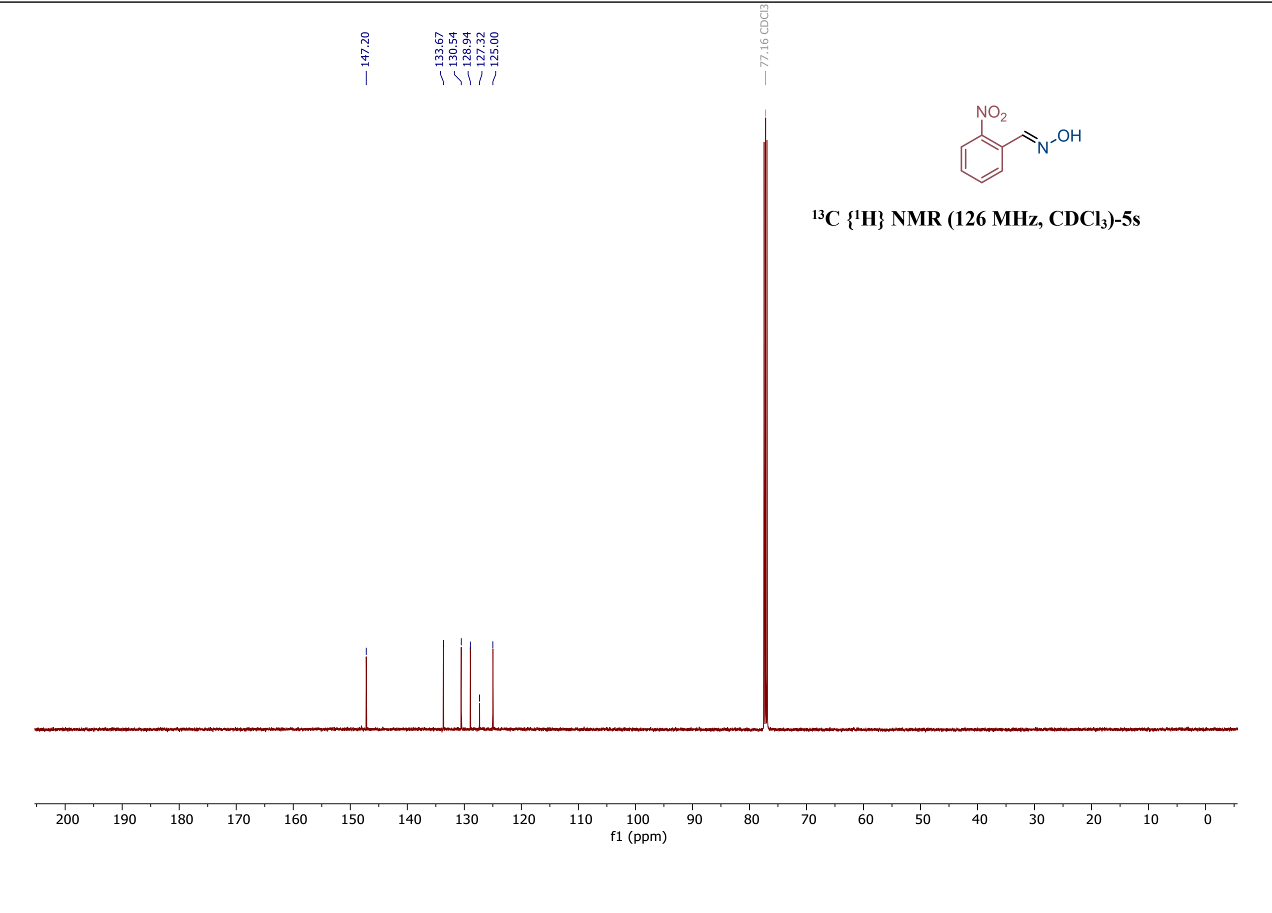

(*E*)-2-nitrobenzaldehyde oxime (5s)[3,9,10]

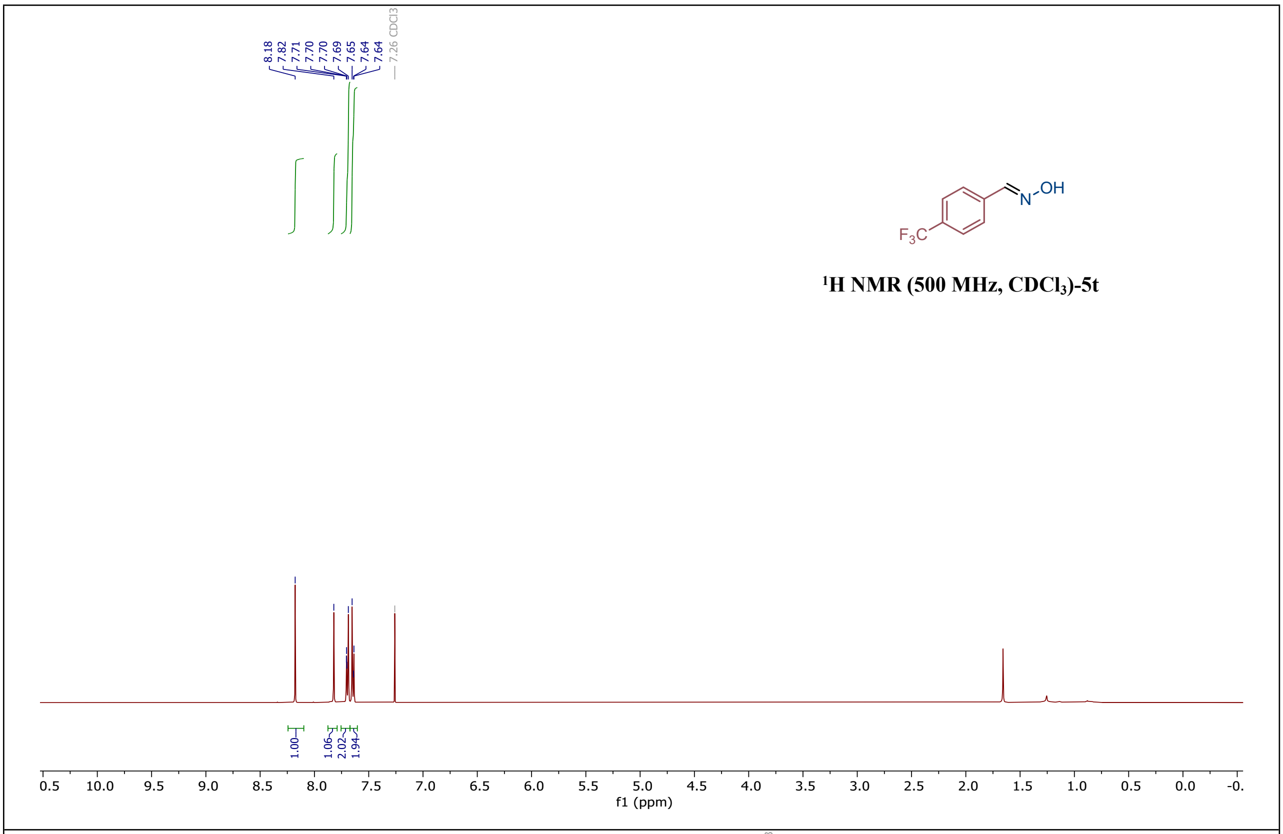

F3C
N
OH
1H NMR (500 MHz, CDCl3)-5t
f1 (ppm)

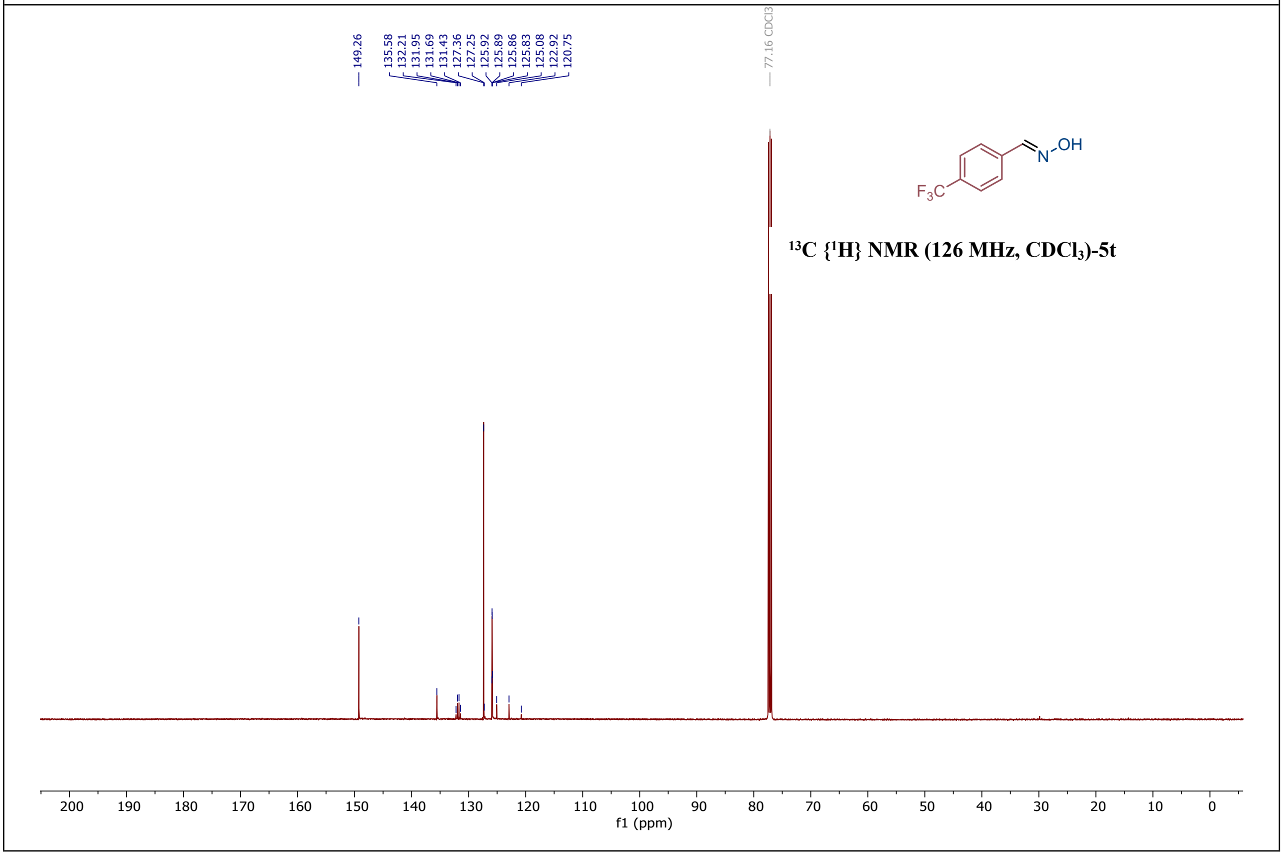

F3C
N
OH
13C {1H} NMR (126 MHz, CDCl3)-5t
f1 (ppm)

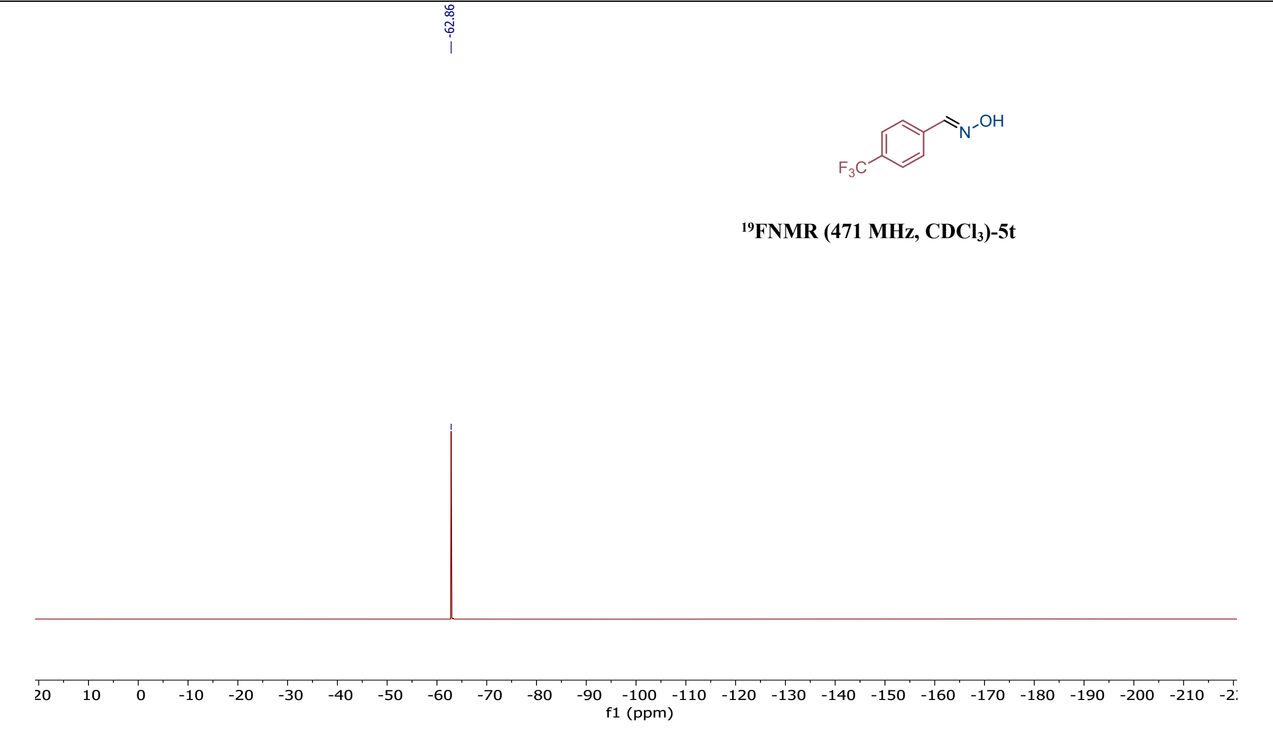

(*E*)-4-(trifluoromethyl)benzaldehyde oxime (5t)

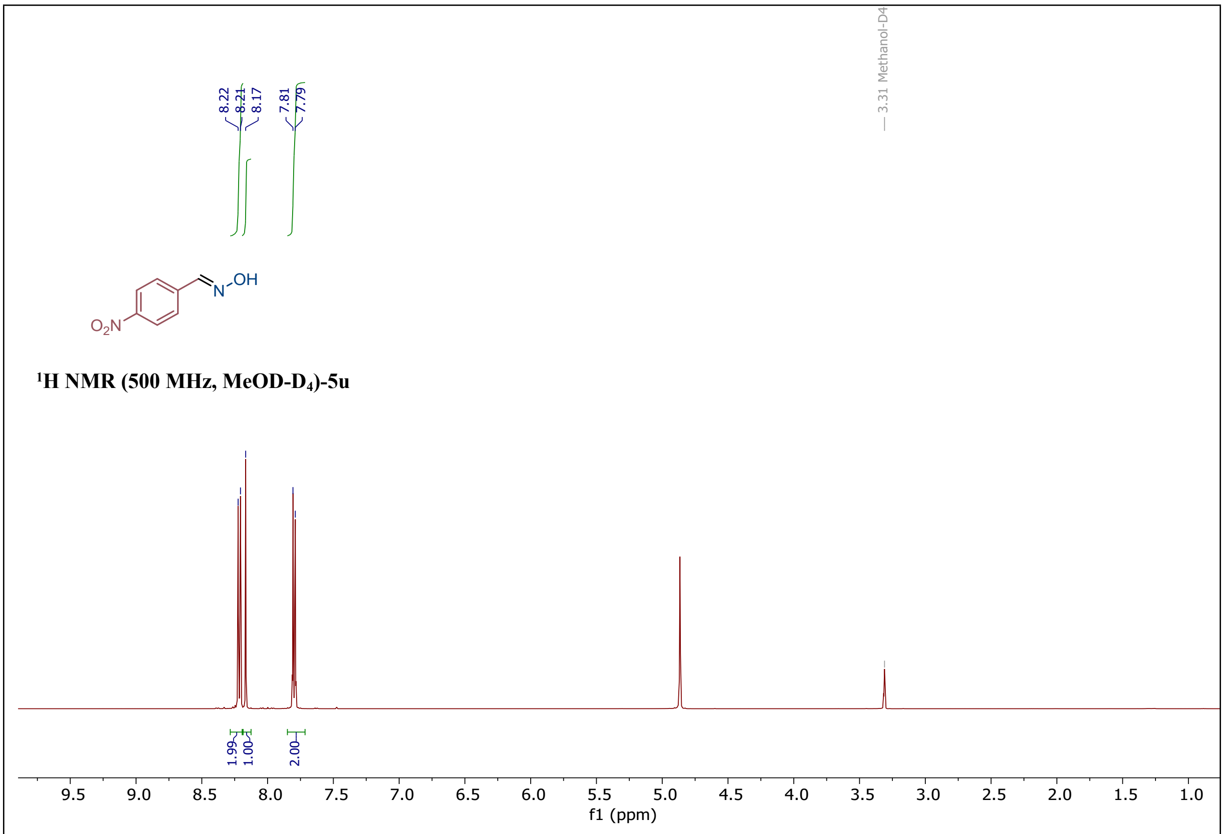

^{1}H NMR (500 MHz, MeOD-D$_4$)-5u

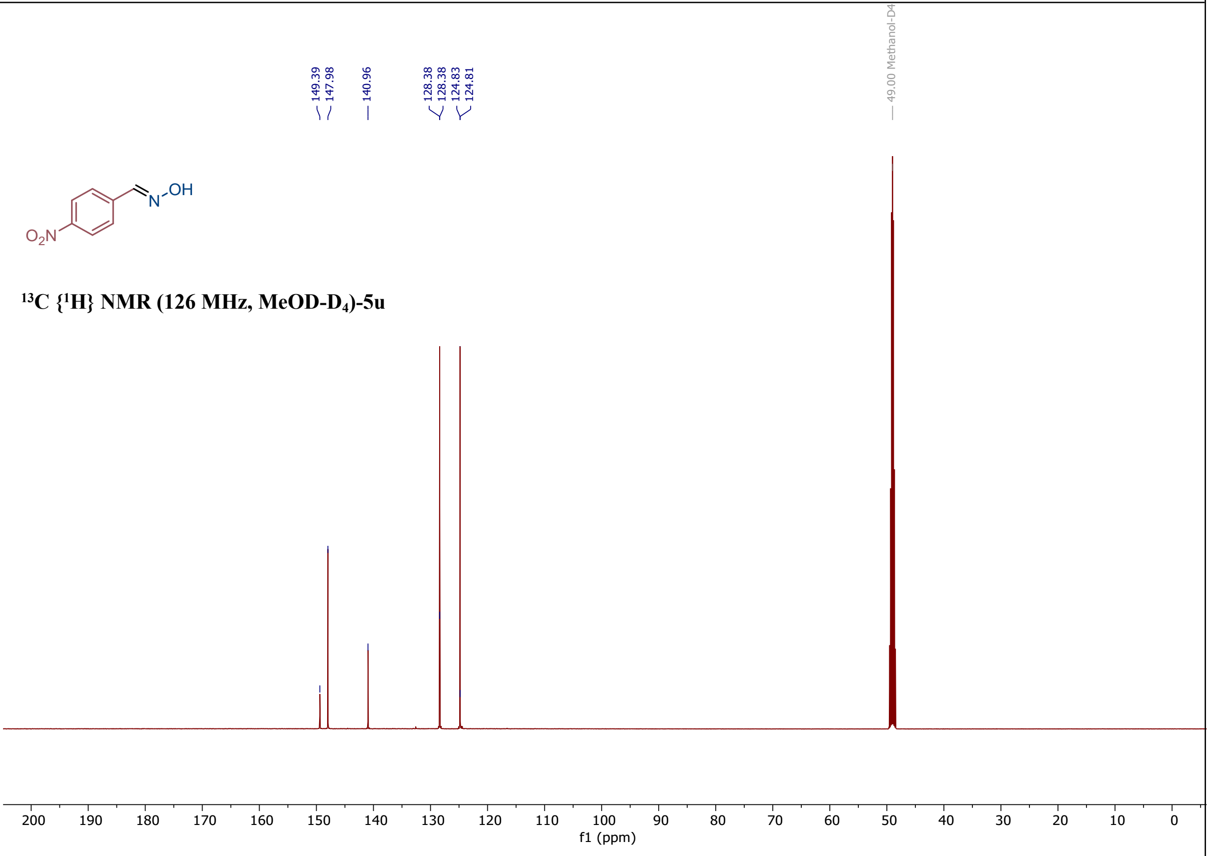

^{13}C {^{1}H} NMR (126 MHz, MeOD-D$_4$)-5u

***(E)-4*-nitrobenzaldehyde oxime (5u)**

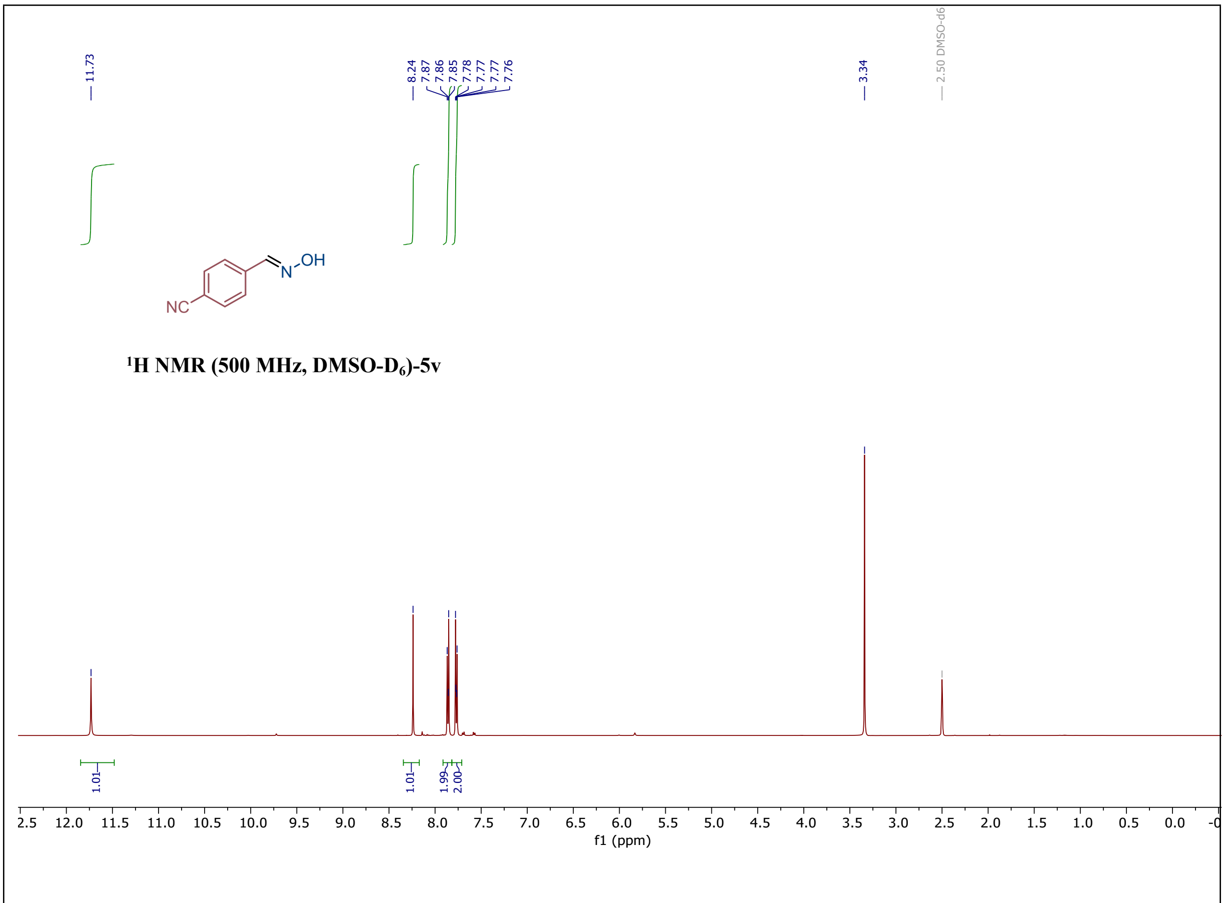

^{1}H NMR (500 MHz, DMSO-D$_6$)-5v

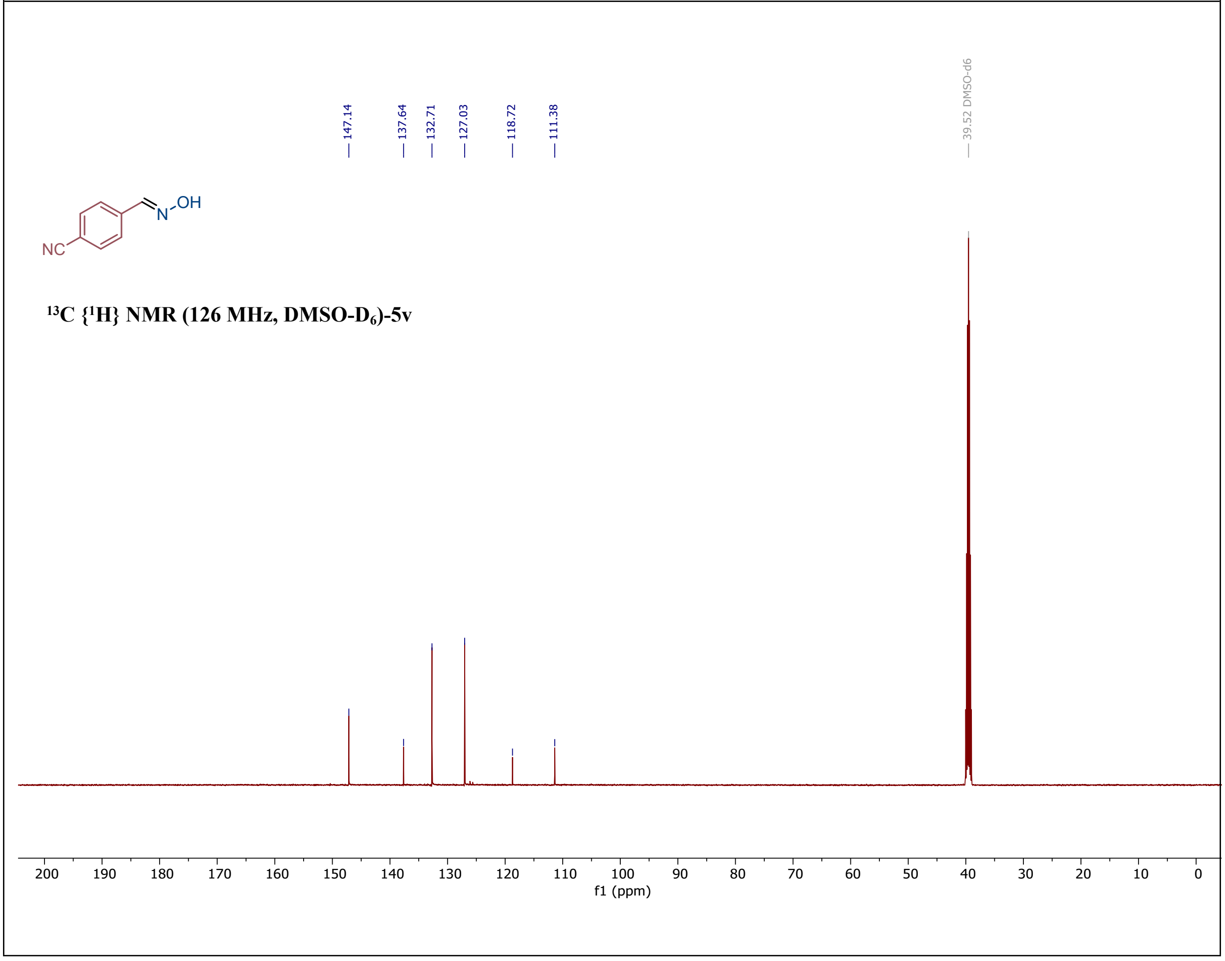

^{13}C {^{1}H} NMR (126 MHz, DMSO-D$_6$)-5v

(*E*)-4-((hydroxyimino)methyl)benzonitrile (5v)

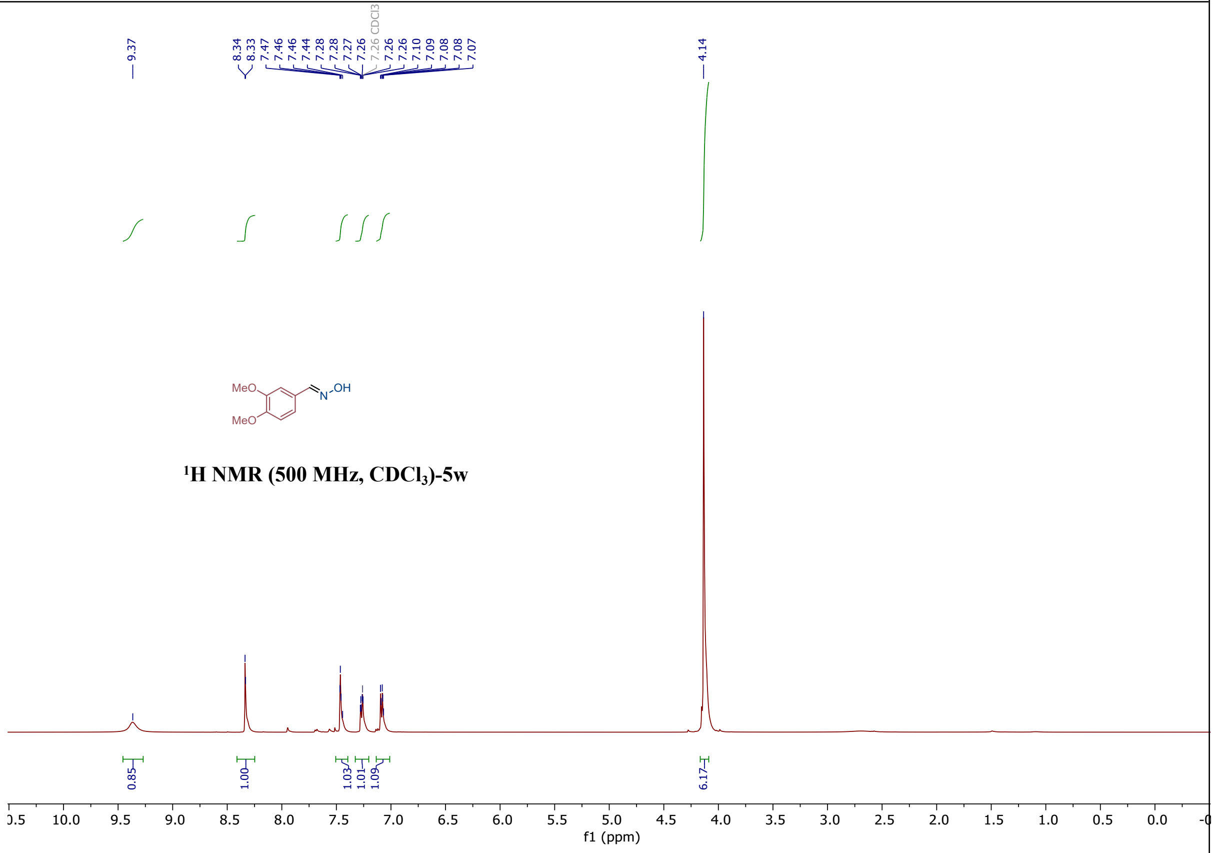

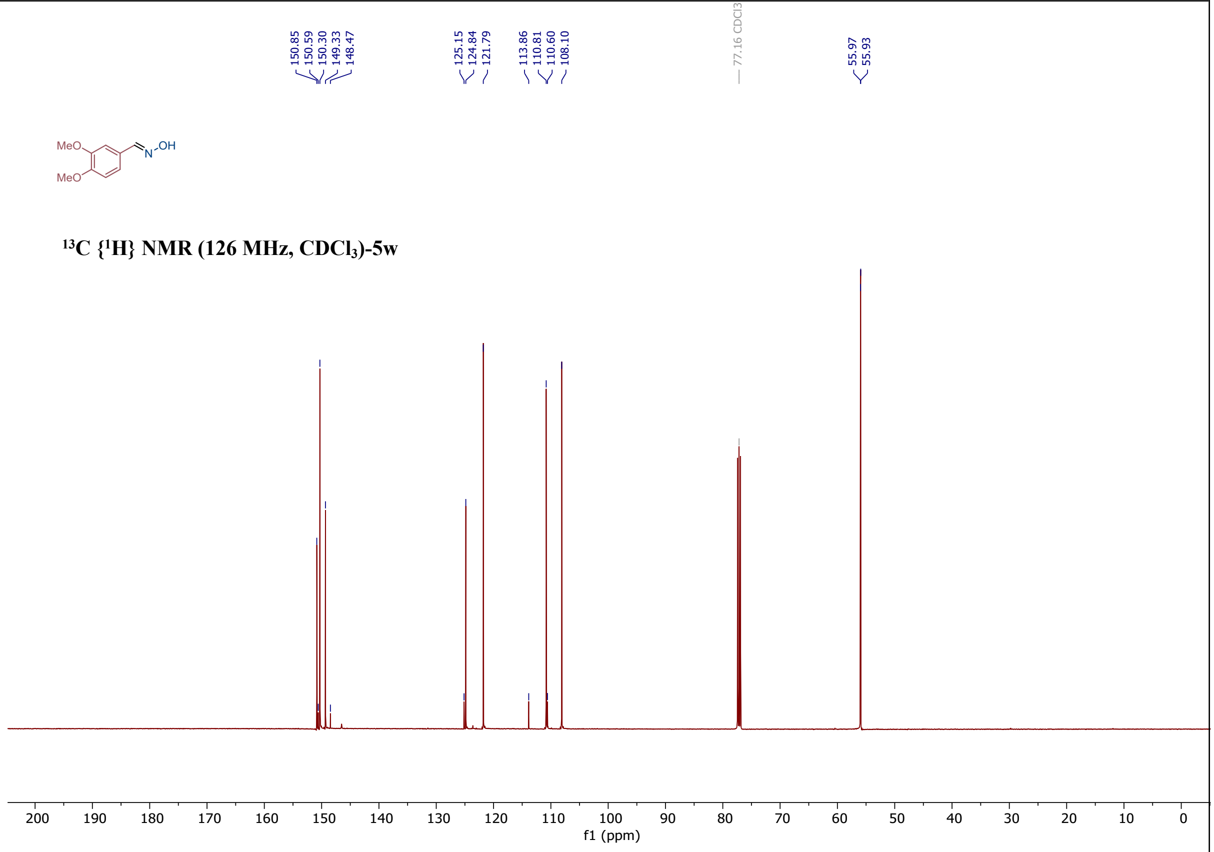

(*E*)-3,4-dimethoxybenzaldehyde oxime (5w)

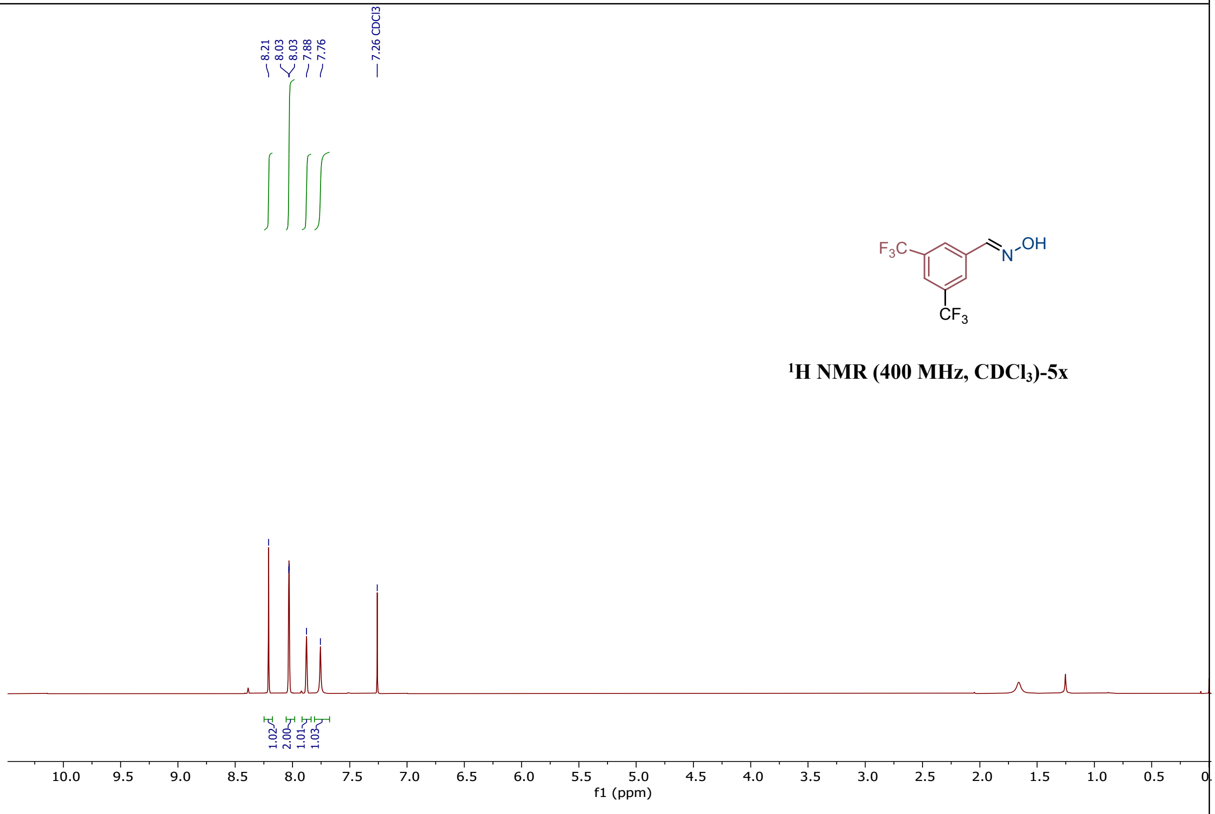

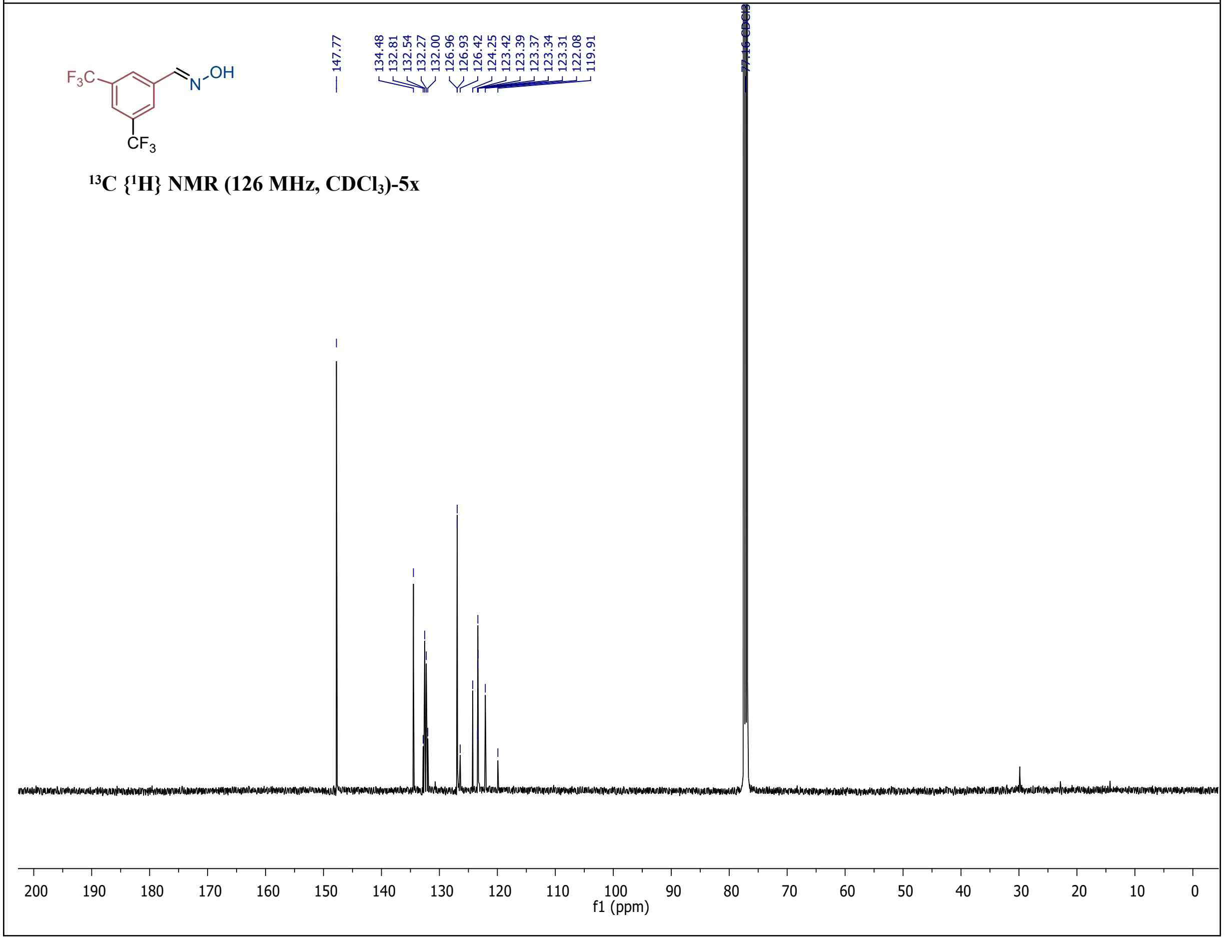

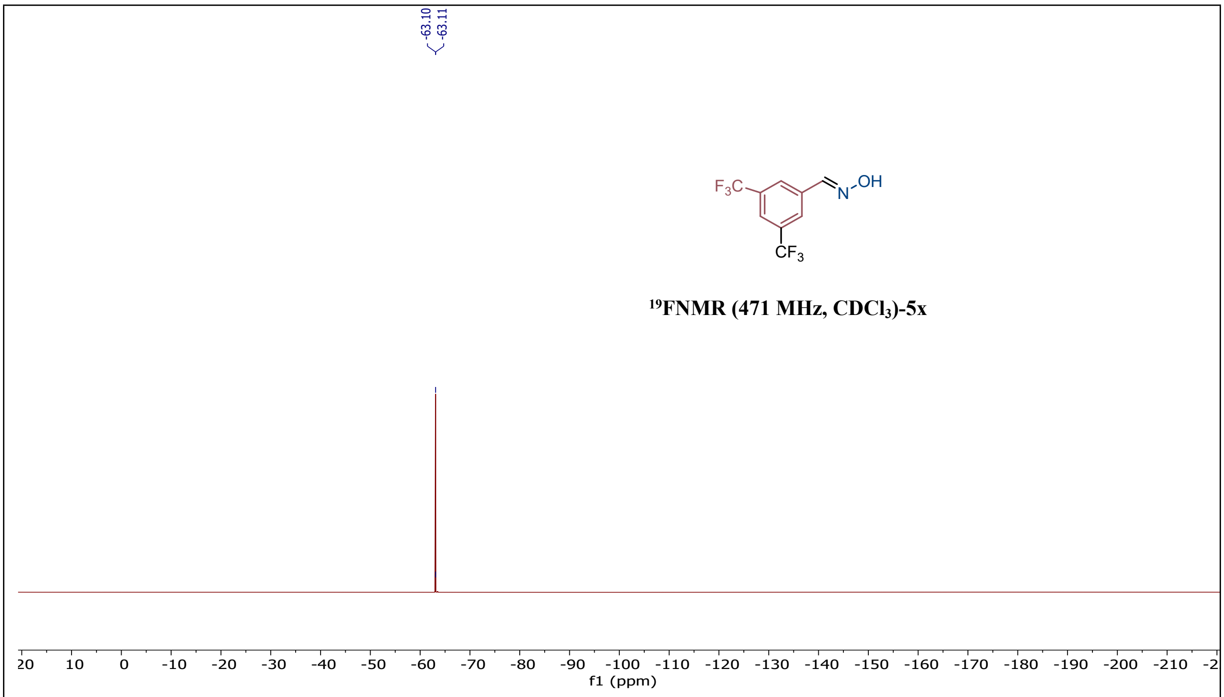

(*E*)-3,5-*bis*(trifluoromethyl)benzaldehyde oxime (5x)

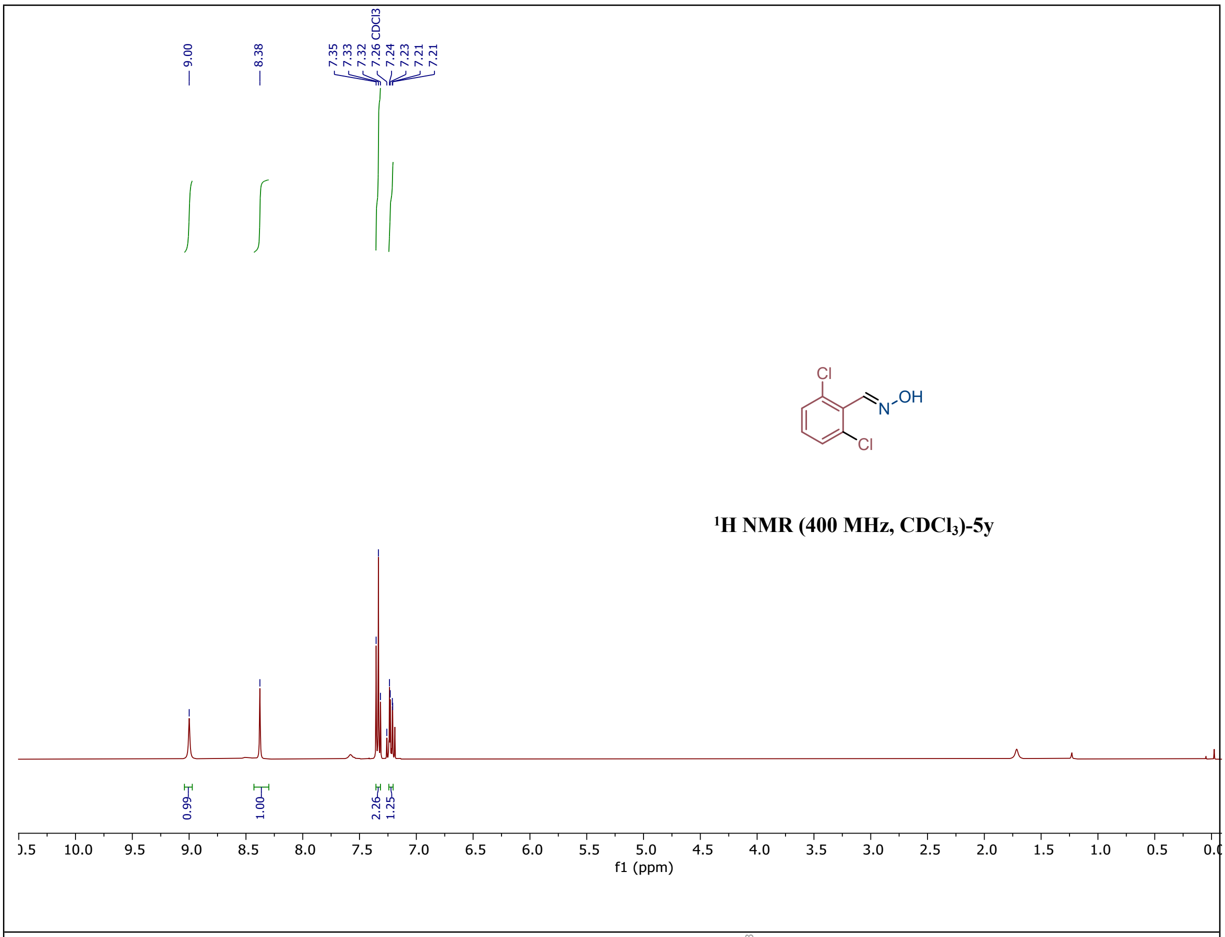

^{1}H NMR (400 MHz, $CDCl_3$)-5y

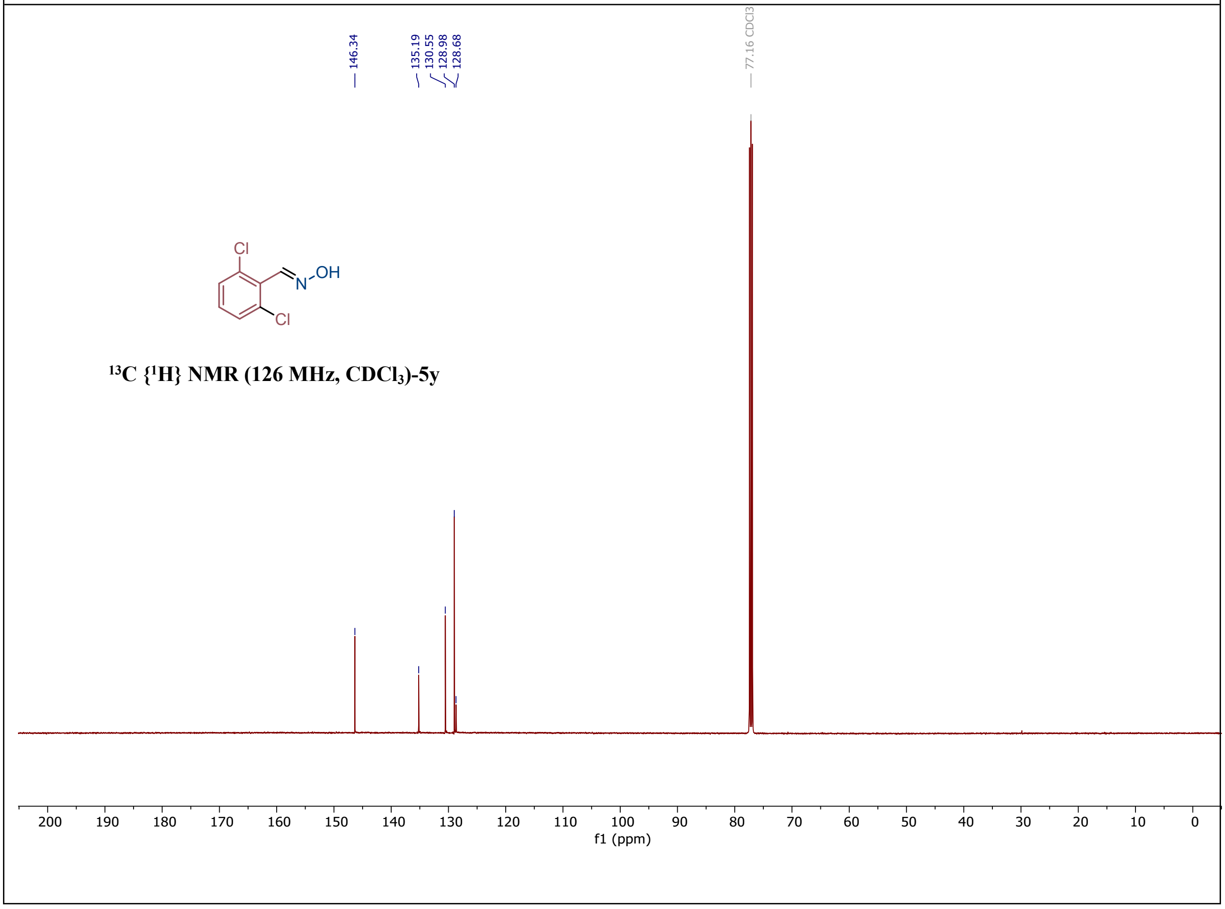

^{13}C {^{1}H} NMR (126 MHz, $CDCl_3$)-5y

(*E*)-2,6-dichlorobenzaldehyde oxime (5y)

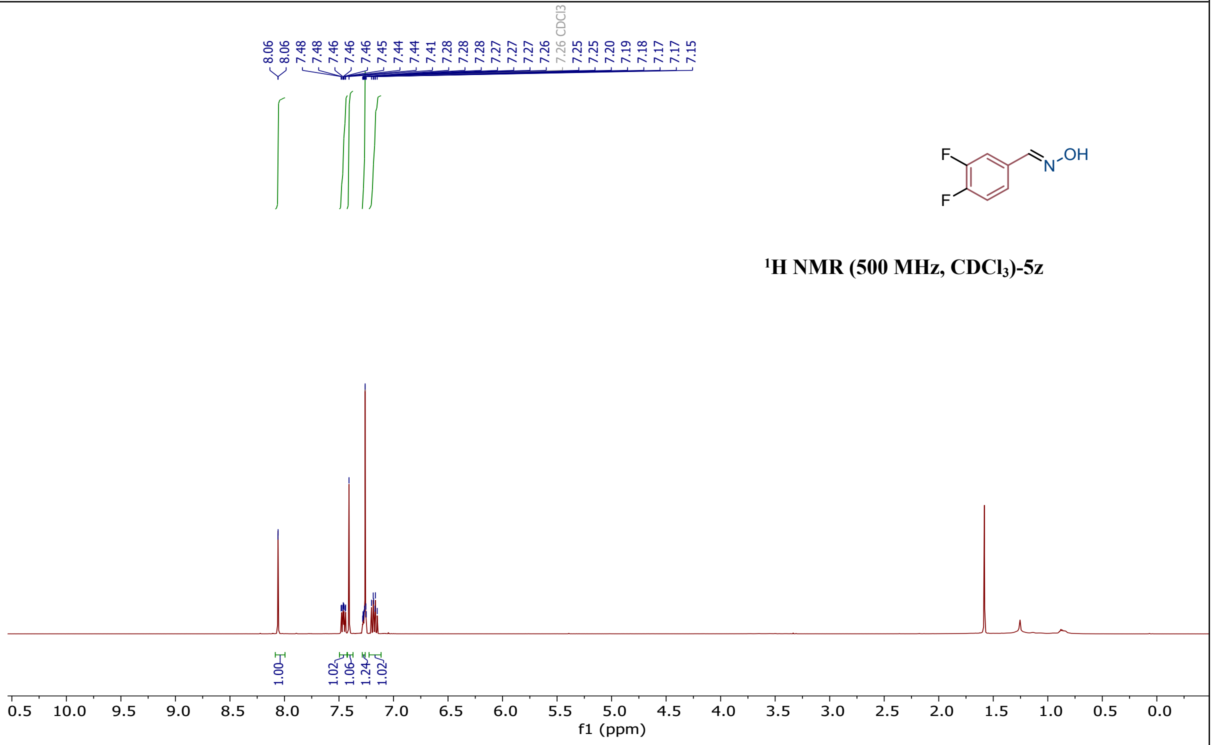

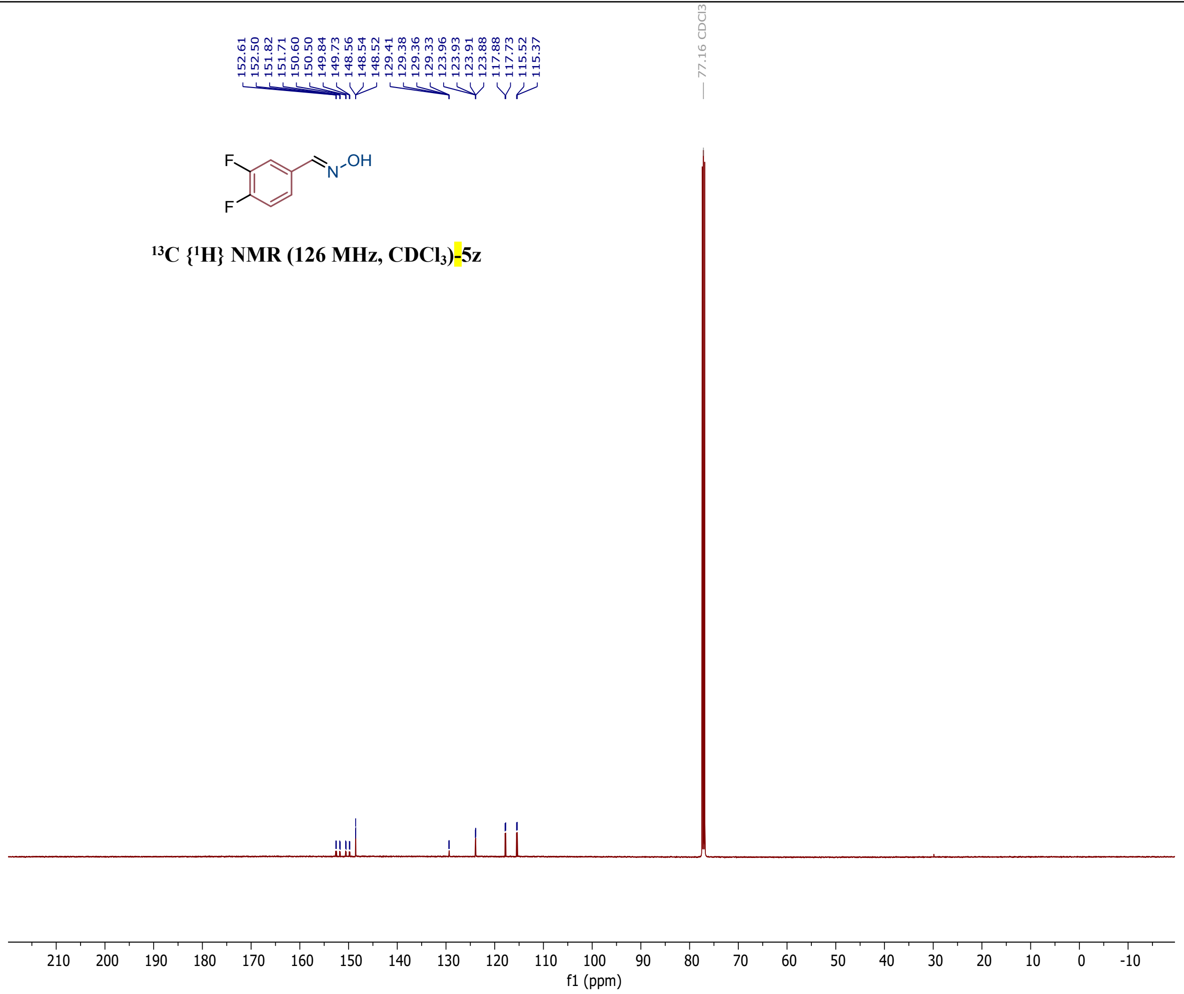

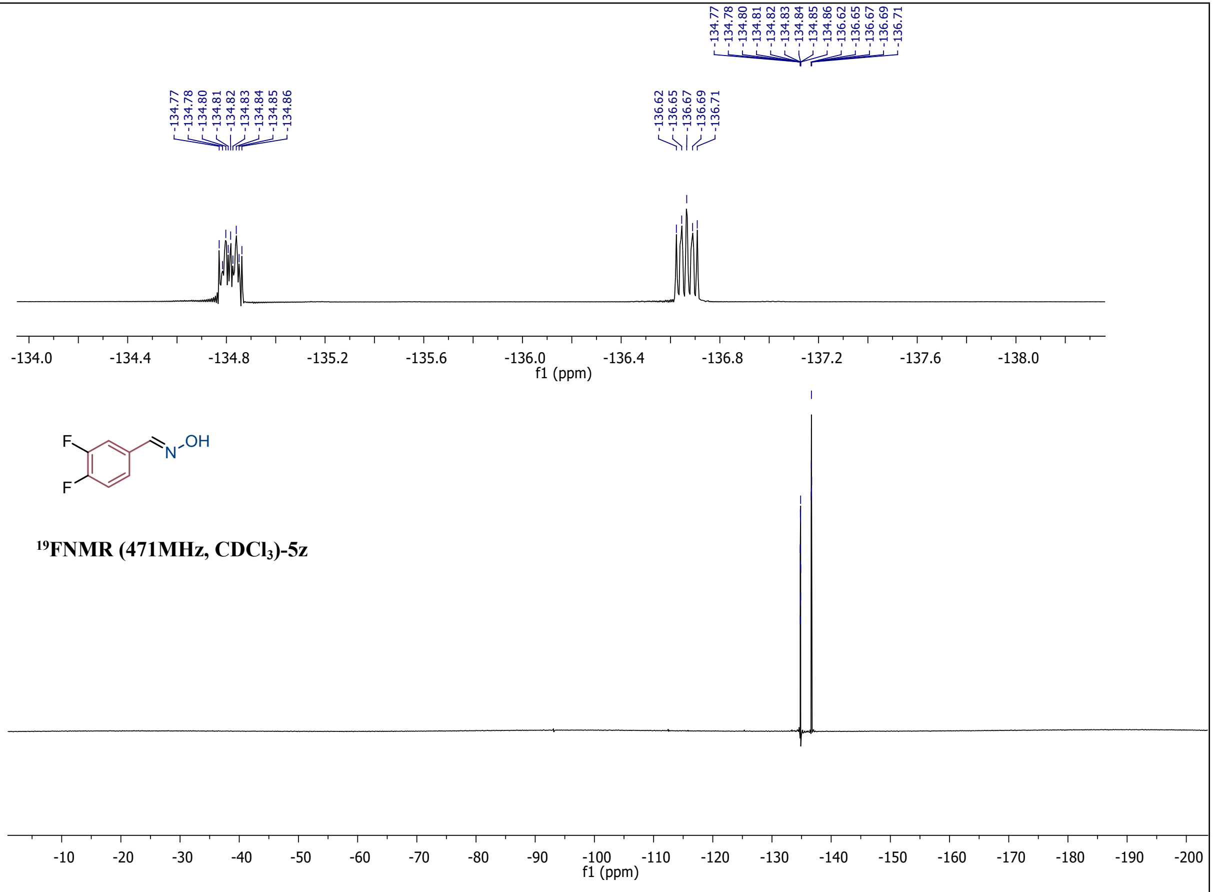

^{19}FNMR (471MHz, $CDCl_3$)-5z

(*E*)-3,4-difluorobenzaldehyde oxime (5z)

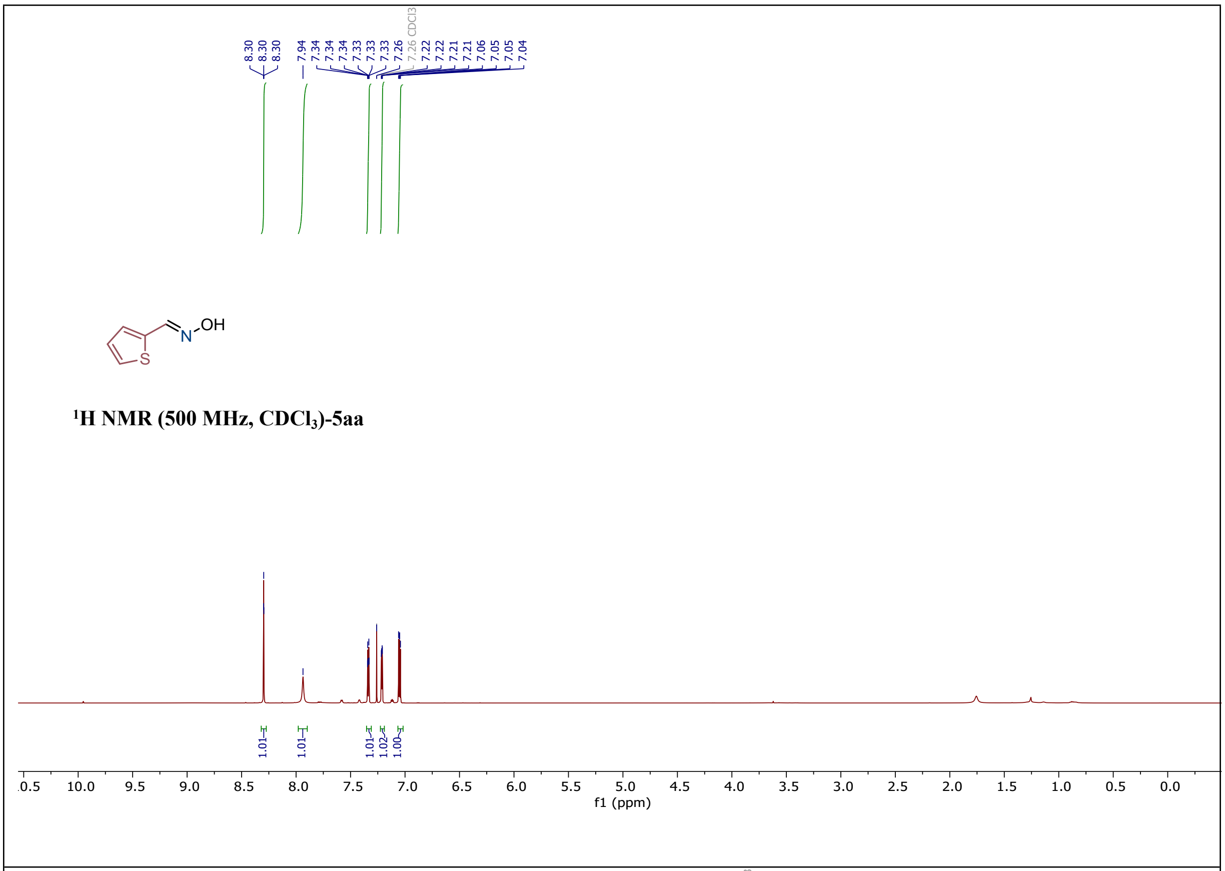

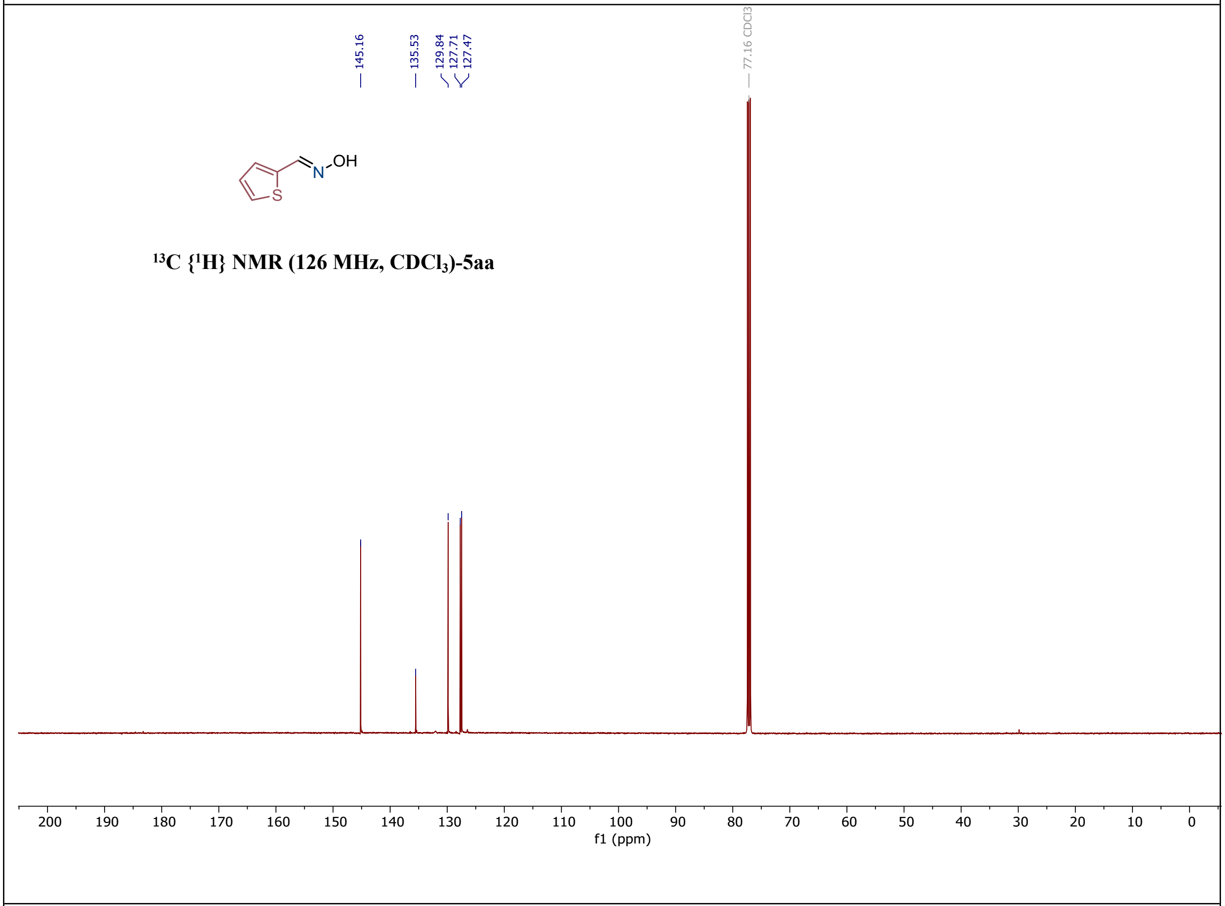

(*E*)-thiophene-2-carbaldehyde oxime (5aa)

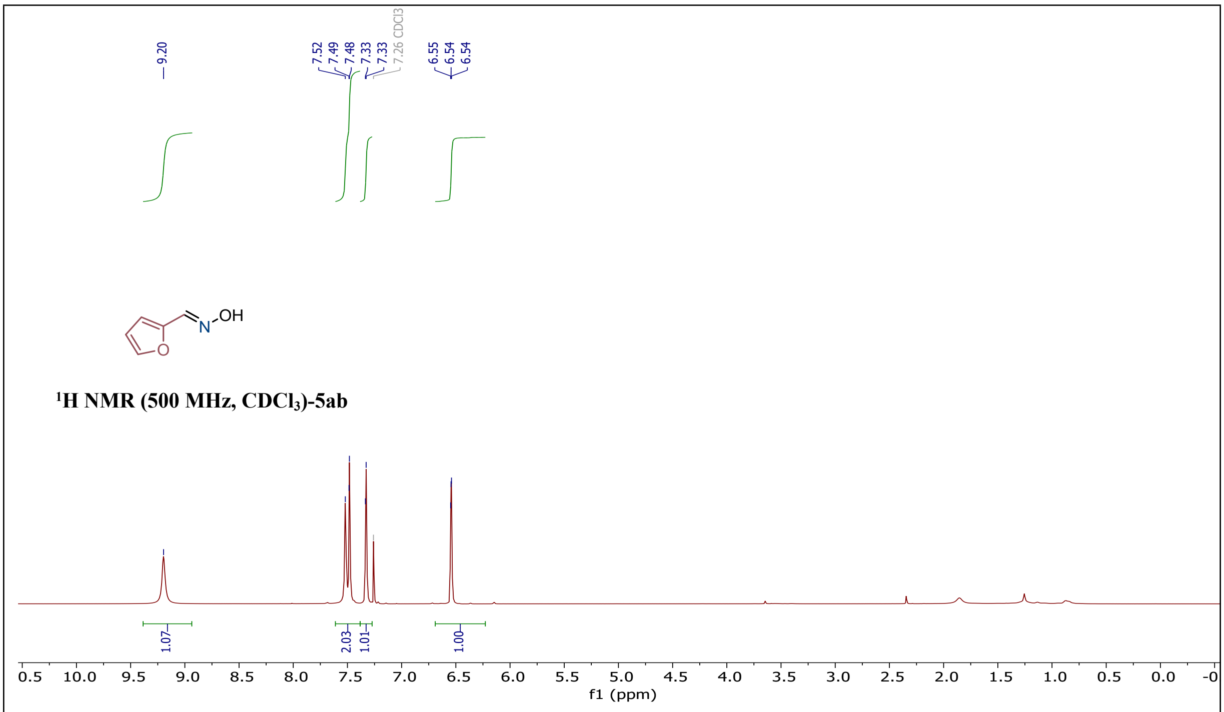

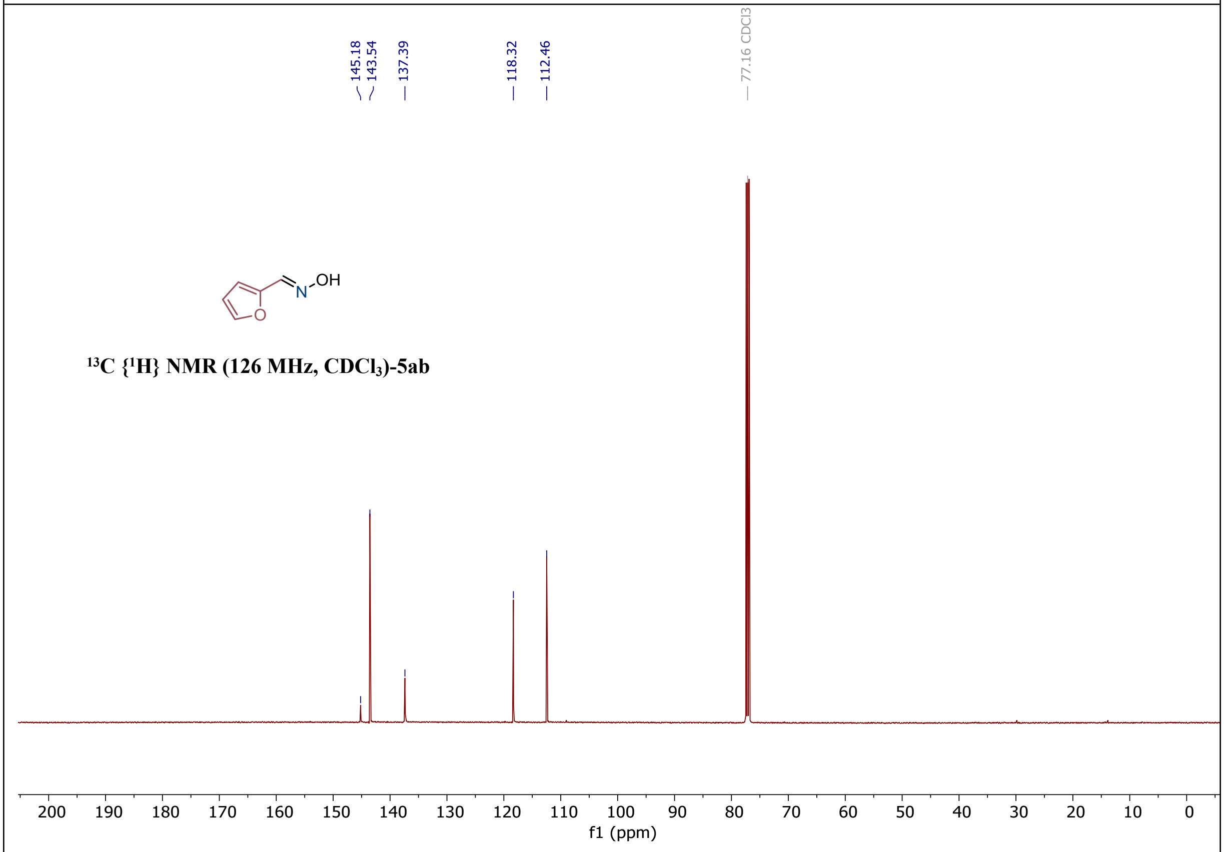

(*E*)-furan-2-carbaldehyde oxime (5ab)

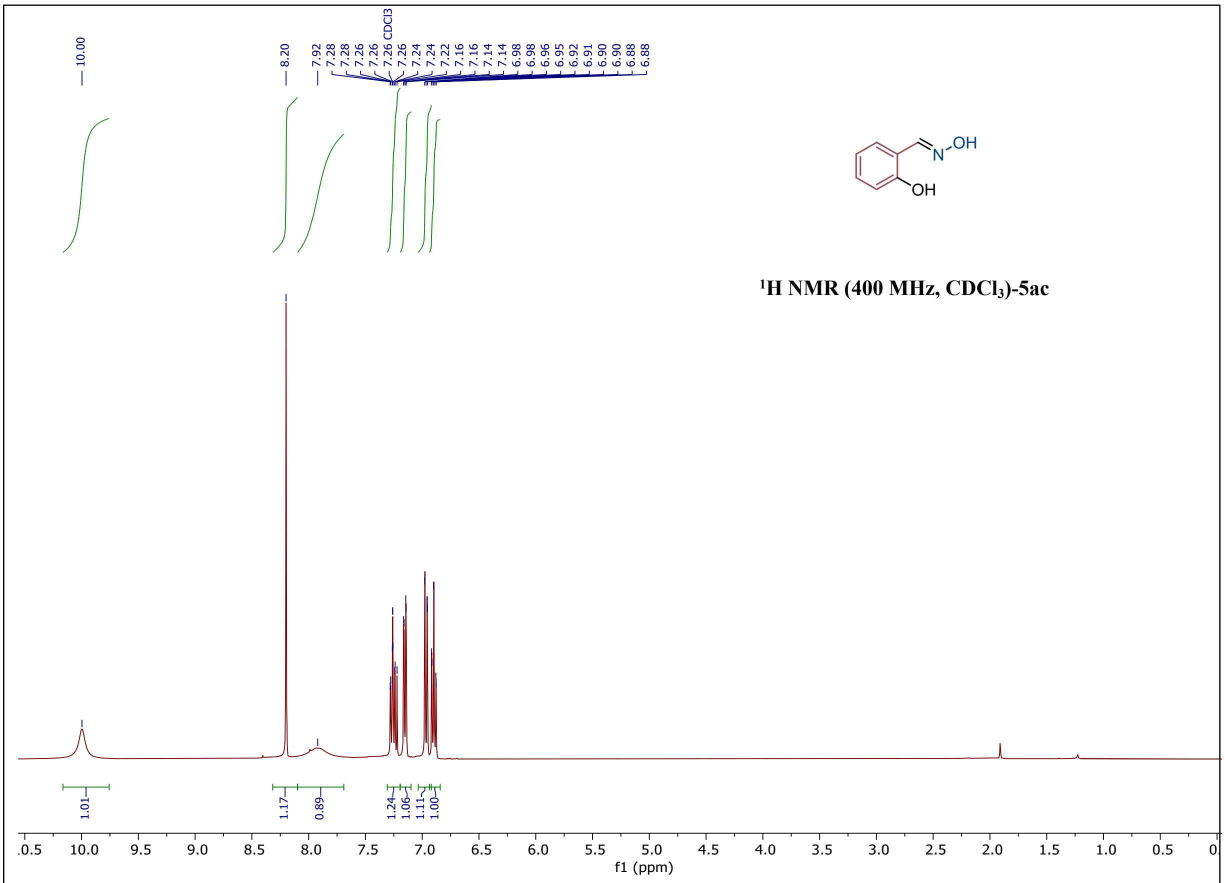

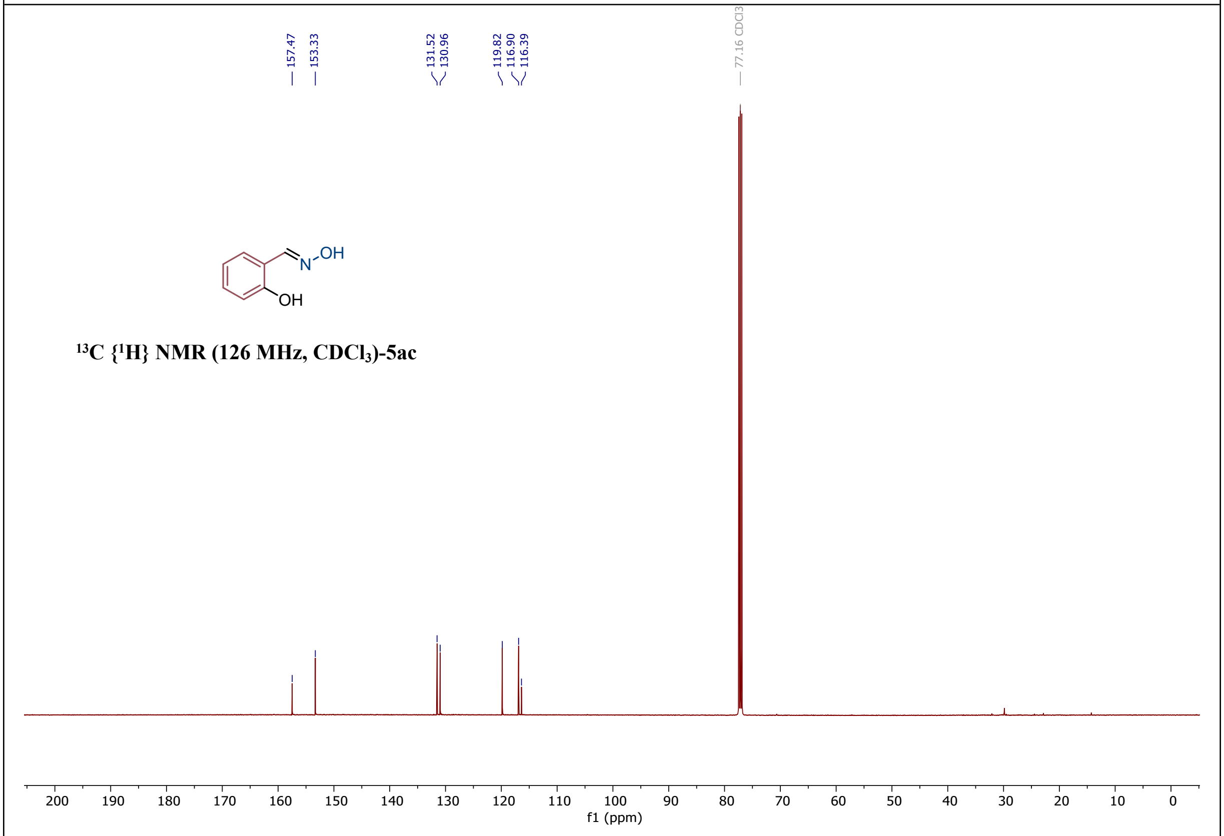

(*E*)-2-hydroxybenzaldehyde oxime (5ac)

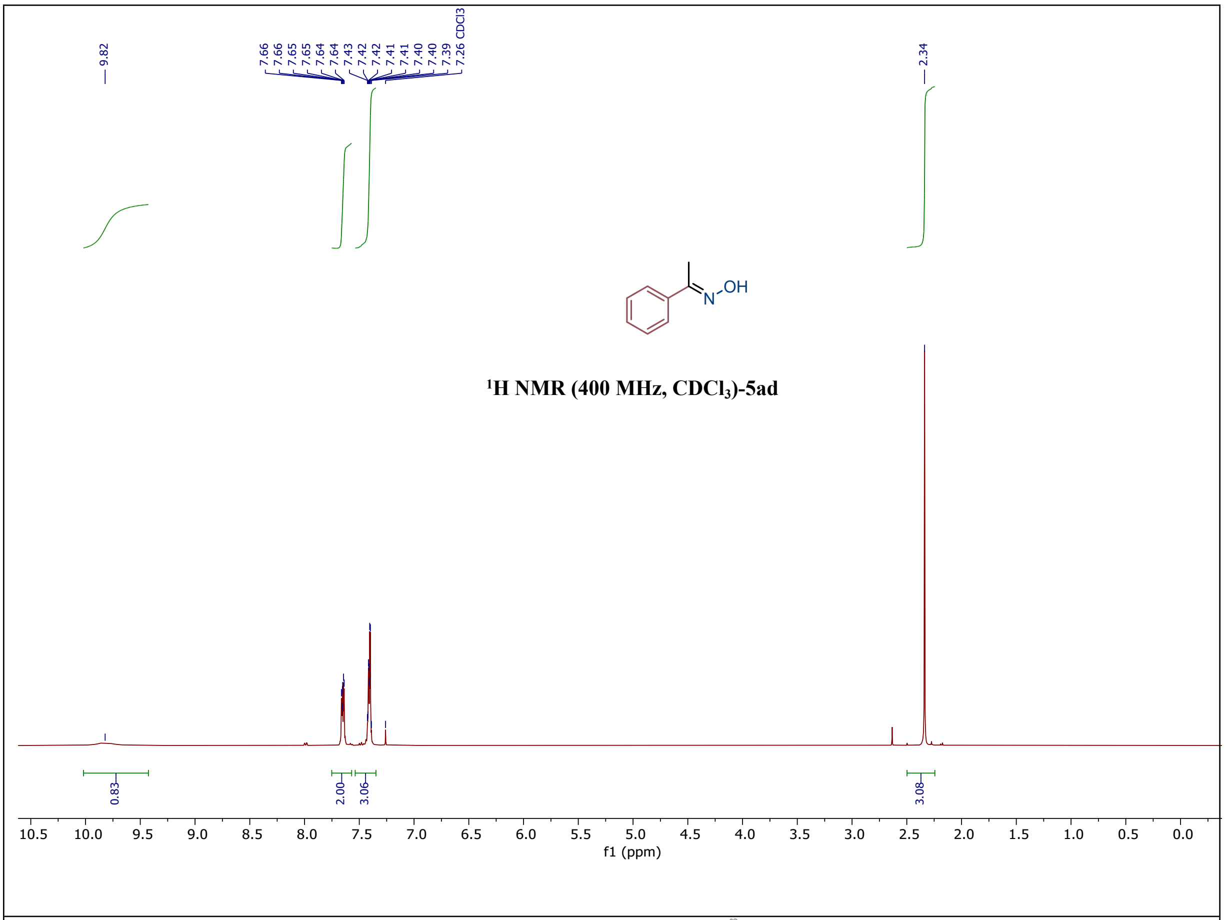

9.82
7.66
7.66
7.65
7.65
7.64
7.64
7.43
7.42
7.42
7.41
7.41
7.40
7.40
7.39
7.26 CDCl3
2.34
N
OH
¹H NMR (400 MHz, CDCl₃)-5ad
0.83
2.00
3.06
3.08
f1 (ppm)

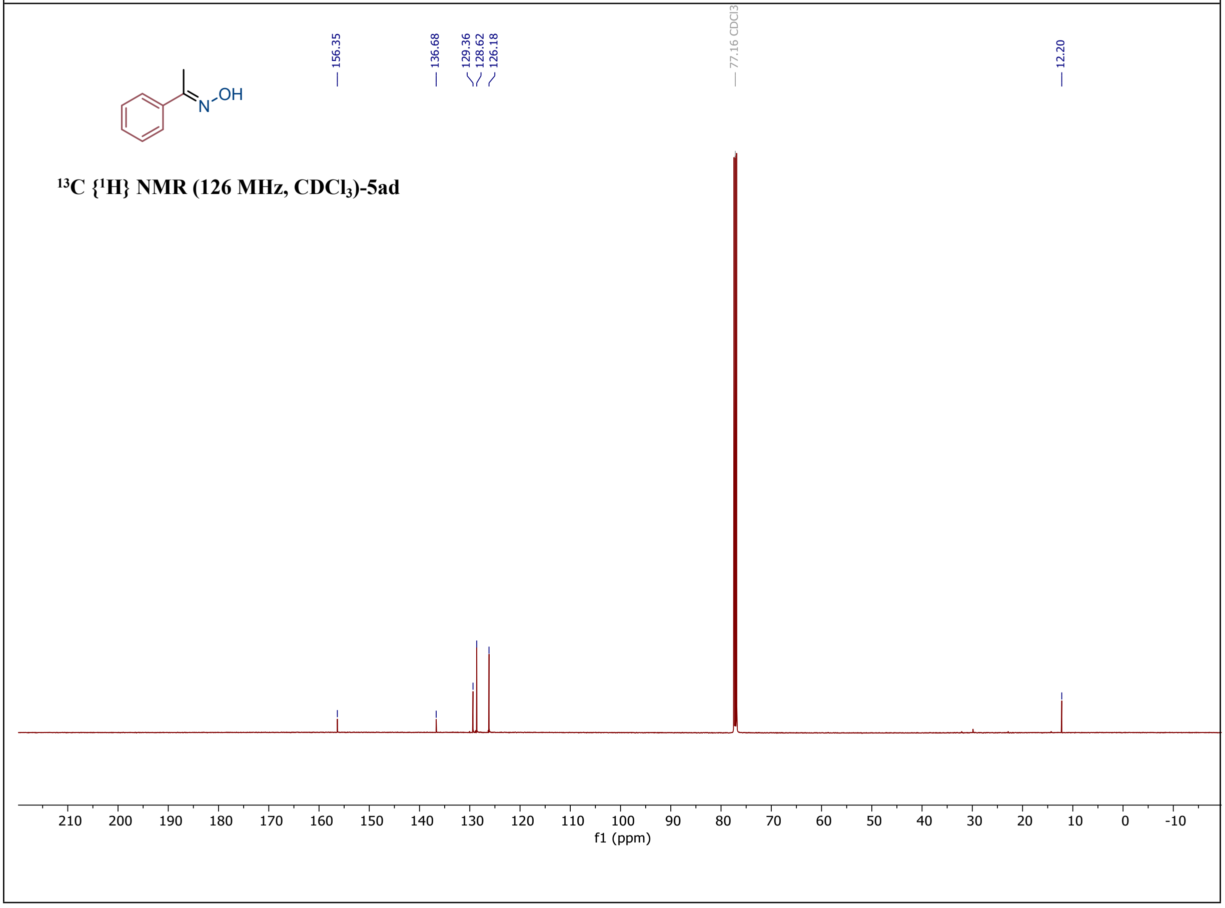

156.35
136.68
129.36
128.62
126.18
77.16 CDCl3
12.20
N
OH
¹³C {¹H} NMR (126 MHz, CDCl₃)-5ad
f1 (ppm)

(*E*)-1-phenylethan-1-one oxime (5ad)

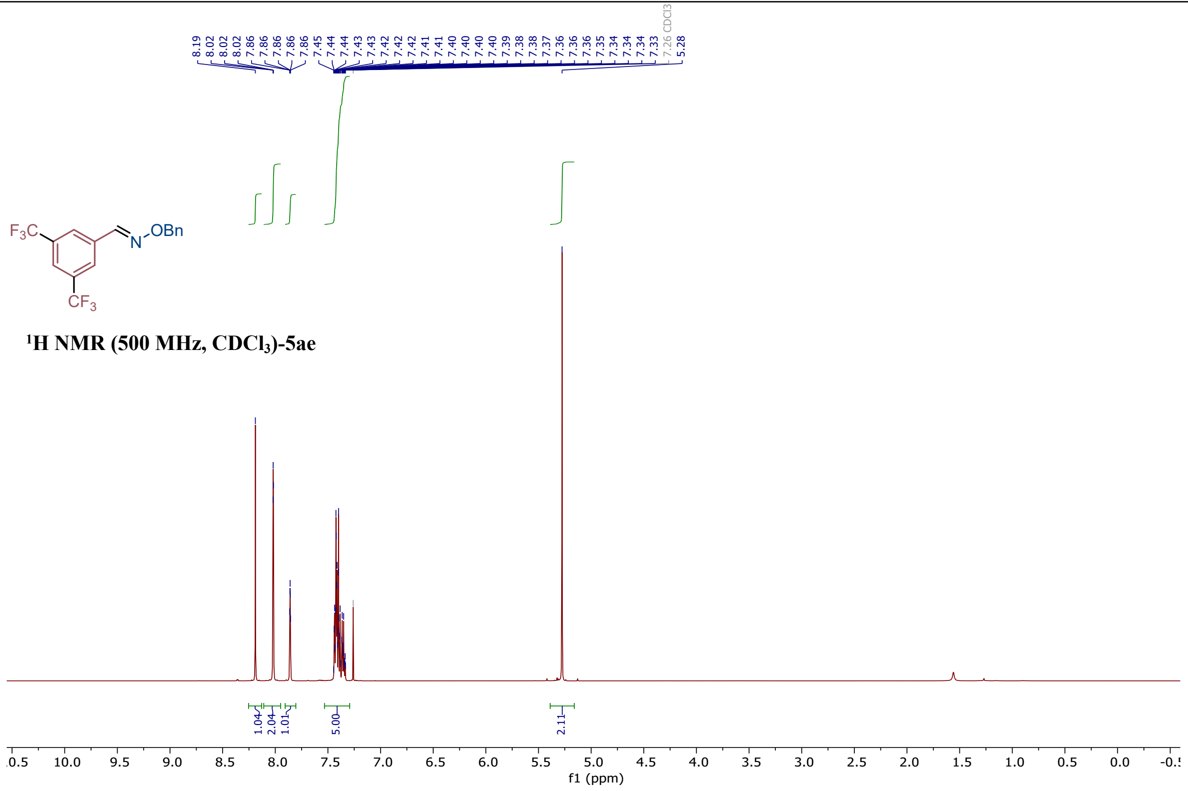

^{1}H NMR (500 MHz, CDCl$_3$)-5ae

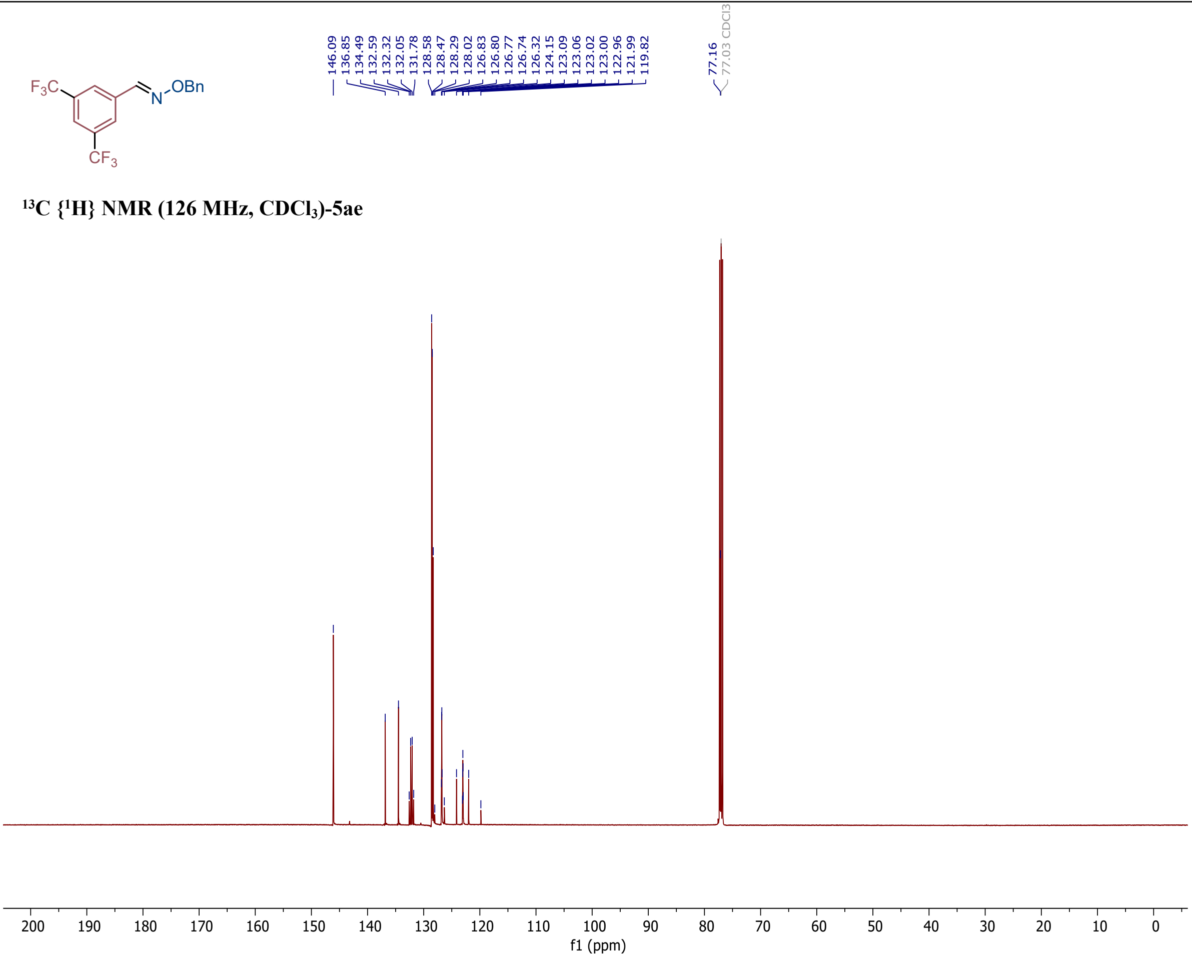

^{13}C {^{1}H} NMR (126 MHz, CDCl$_3$)-5ae

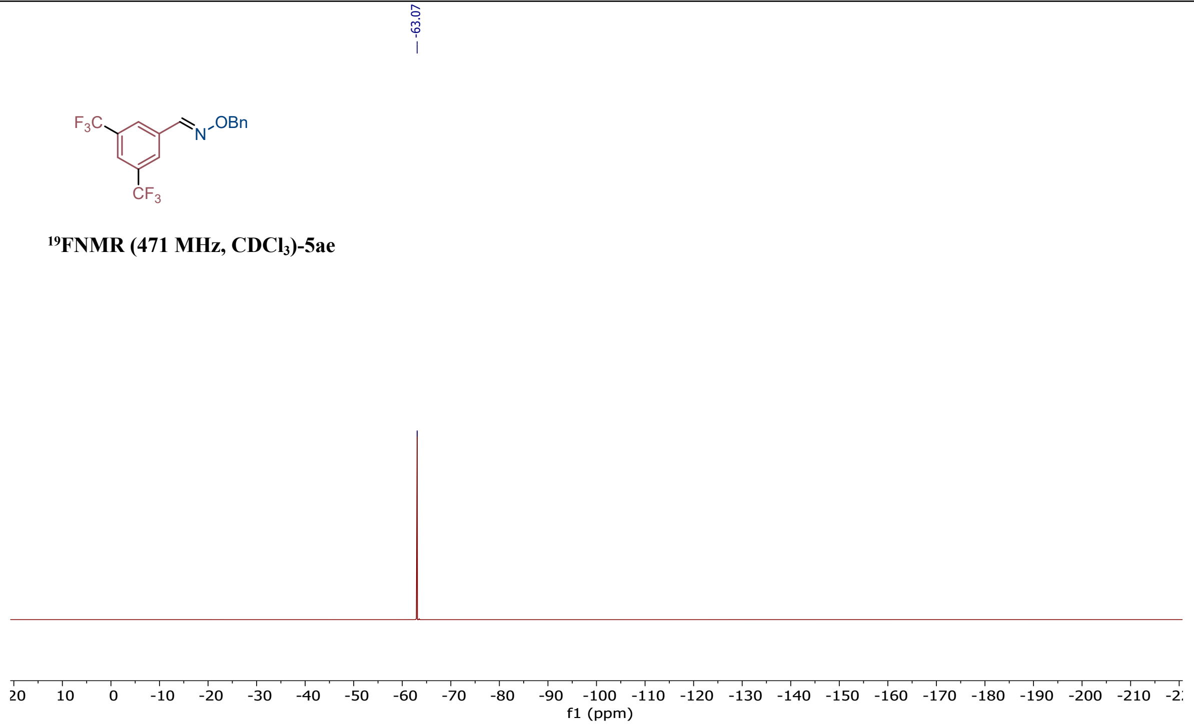

(*E*)-3,5-*bis*(trifluoromethyl)benzaldehyde *O*-benzyl oxime (5ae)

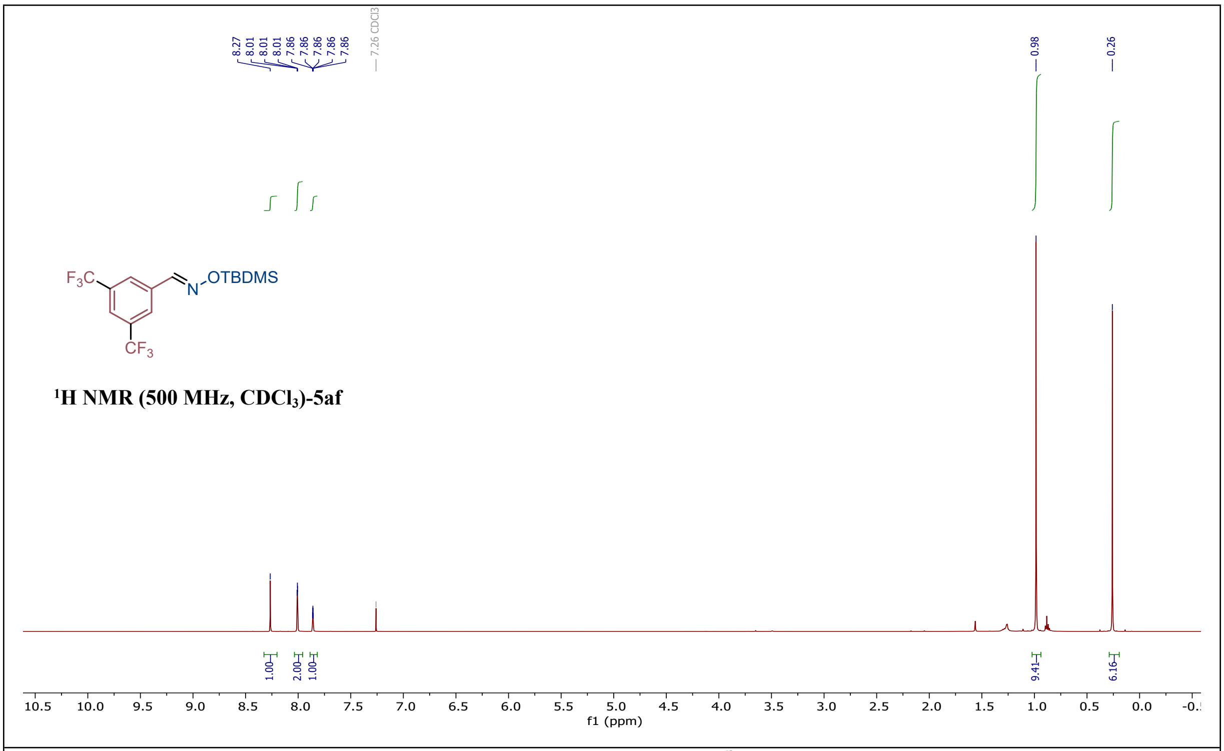
8.27
8.01
8.01
8.01
7.86
7.86
7.86
7.86
7.86
7.26 CDCl3
0.98
0.26
F3C
N
OTBDMS
CF3
1H NMR (500 MHz, CDCl3)-5af
1.00
2.00
1.00
9.41
6.16
f1 (ppm)

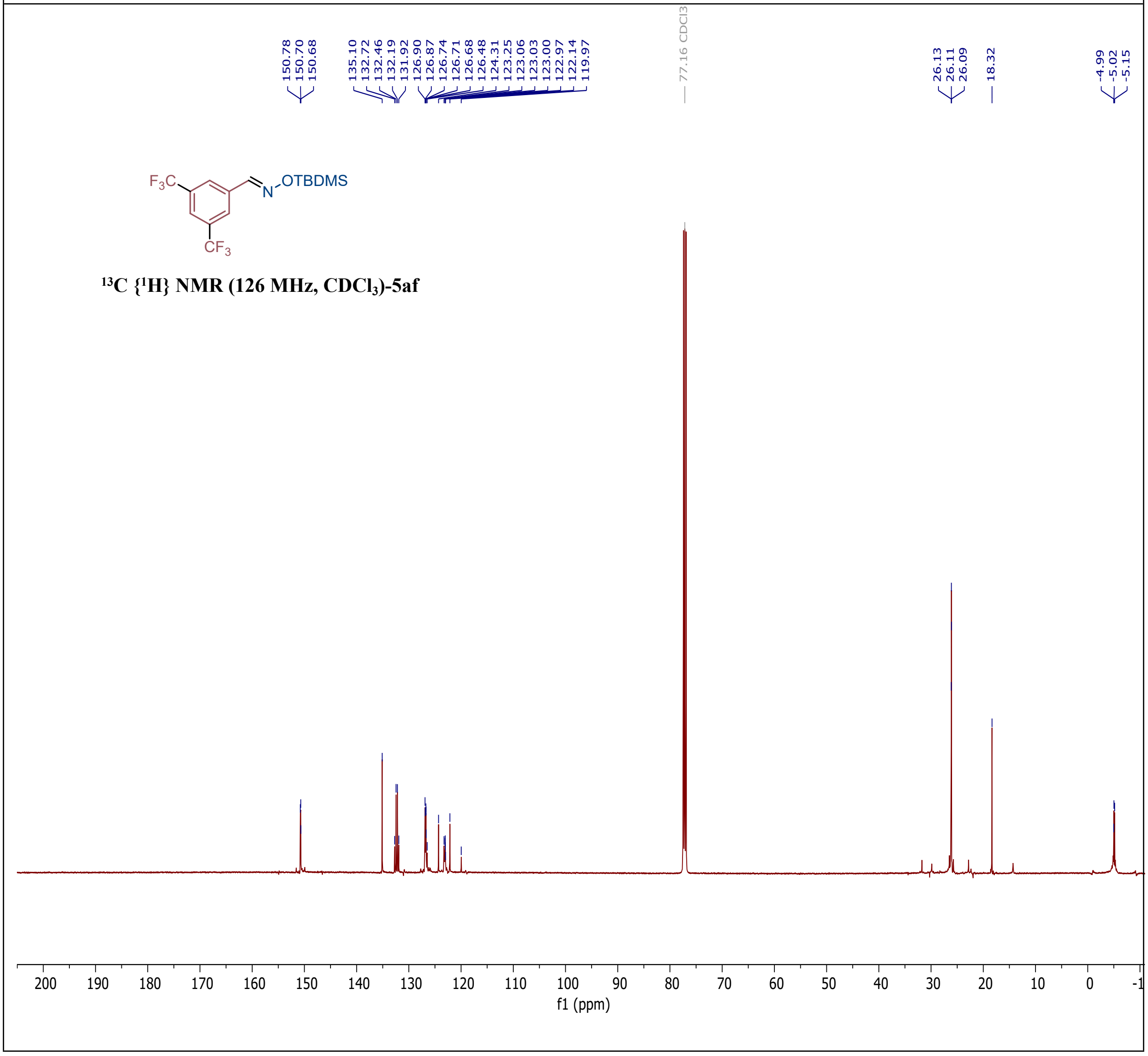
150.78
150.70
150.68
135.10
132.72
132.46
132.19
131.92
126.90
126.87
126.74
126.71
126.68
126.48
124.31
123.25
123.06
123.03
123.00
122.97
122.14
119.97
77.16 CDCl3
26.13
26.11
26.09
18.32
-4.99
-5.02
-5.15
F3C
N
OTBDMS
CF3
13C {1H} NMR (126 MHz, CDCl3)-5af
f1 (ppm)

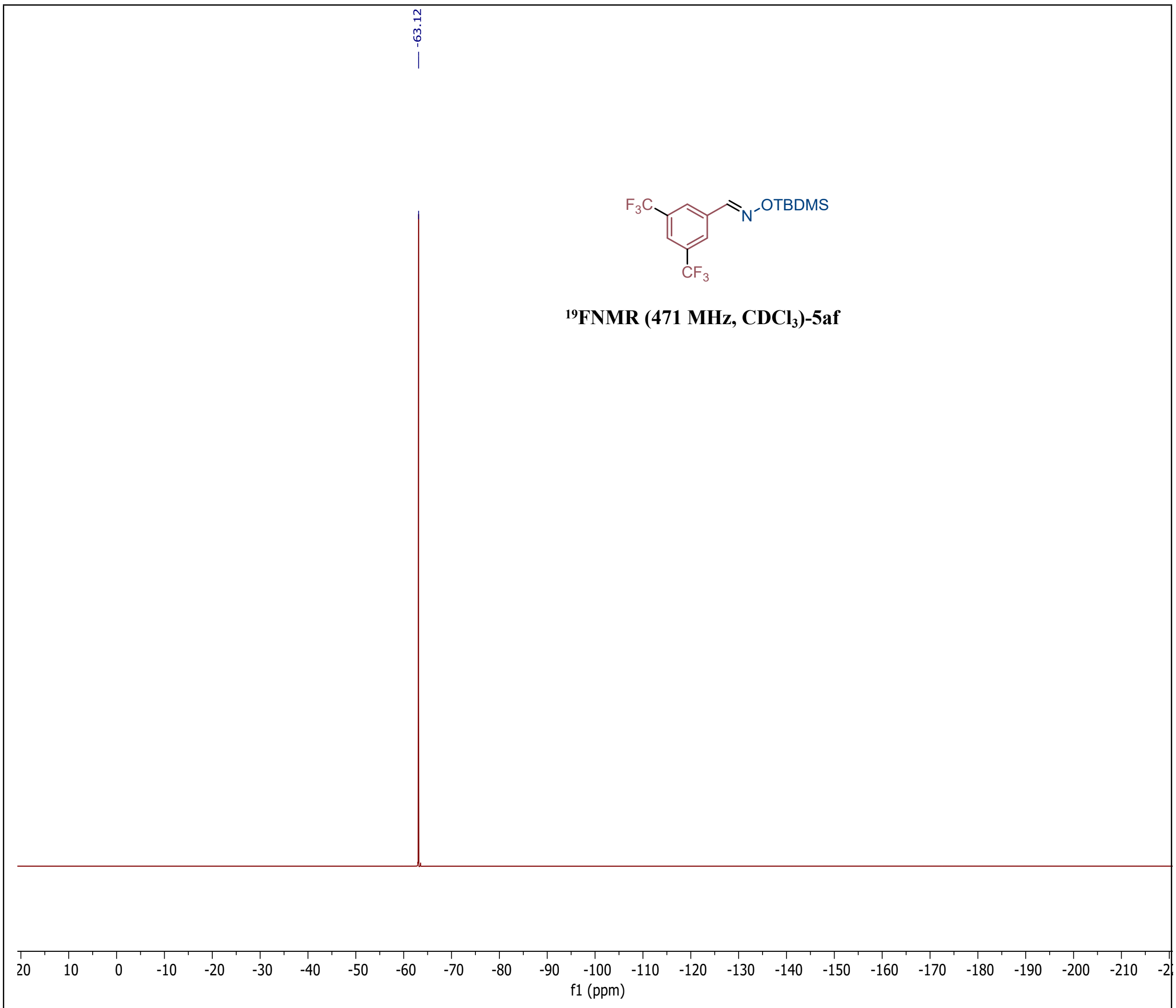

(*E*)-3,5-*bis*(trifluoromethyl)benzaldehyde *O*-(*tert*-butyldimethylsilyl) oxime (5af)

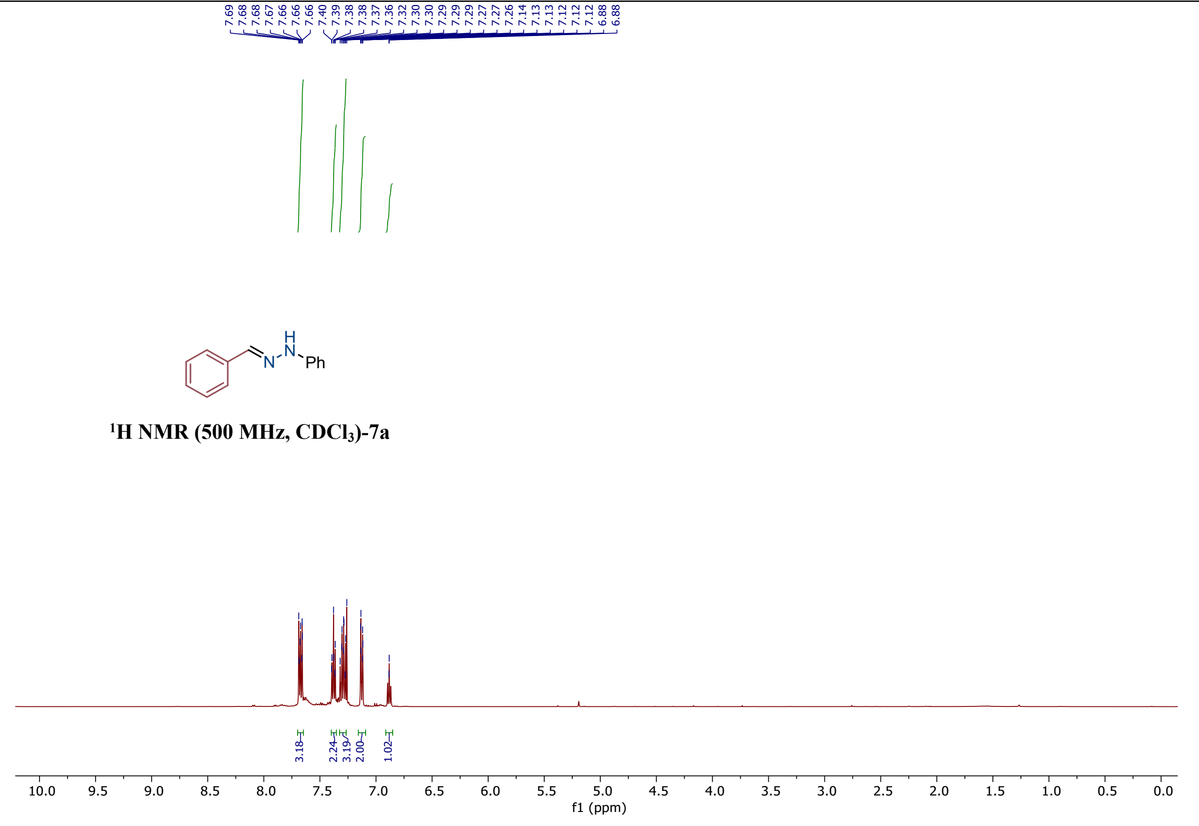

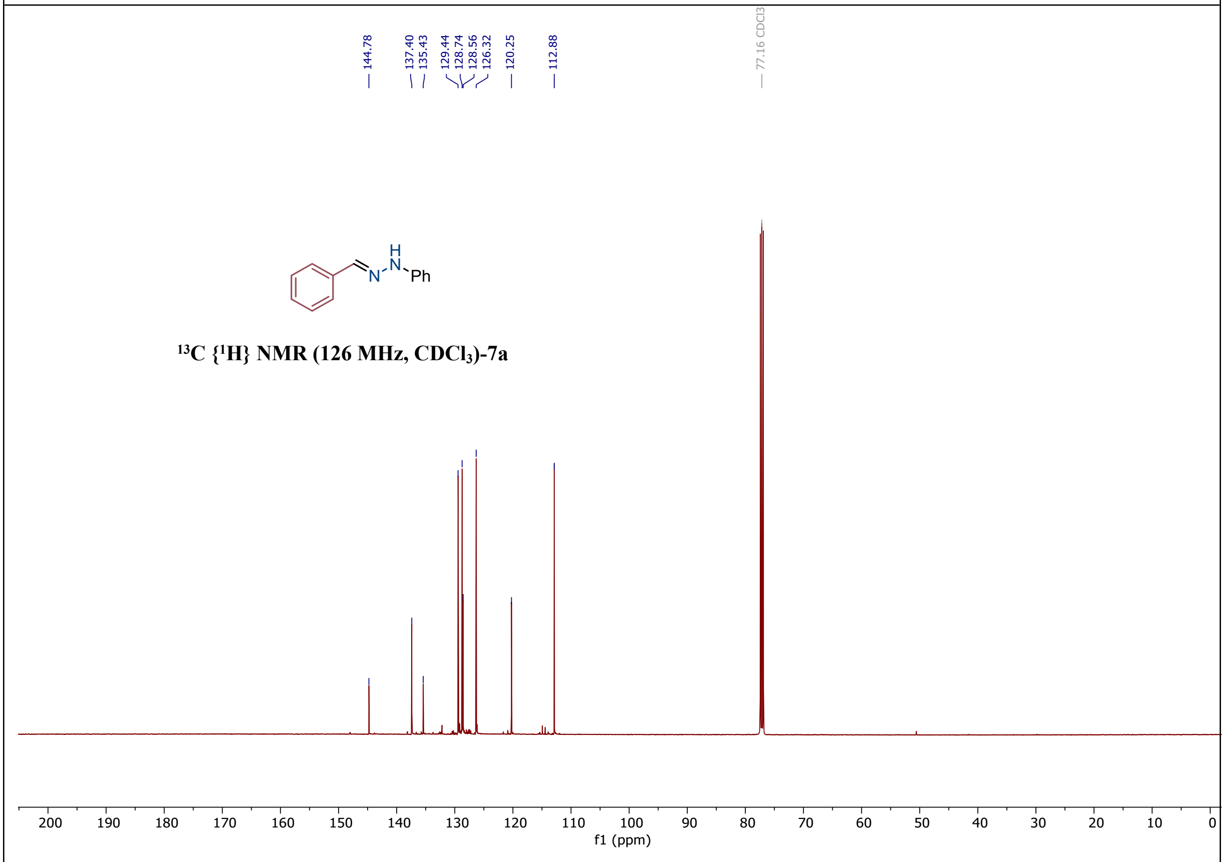

(*E*)-1-benzylidene-2-phenylhydrazine(7a)

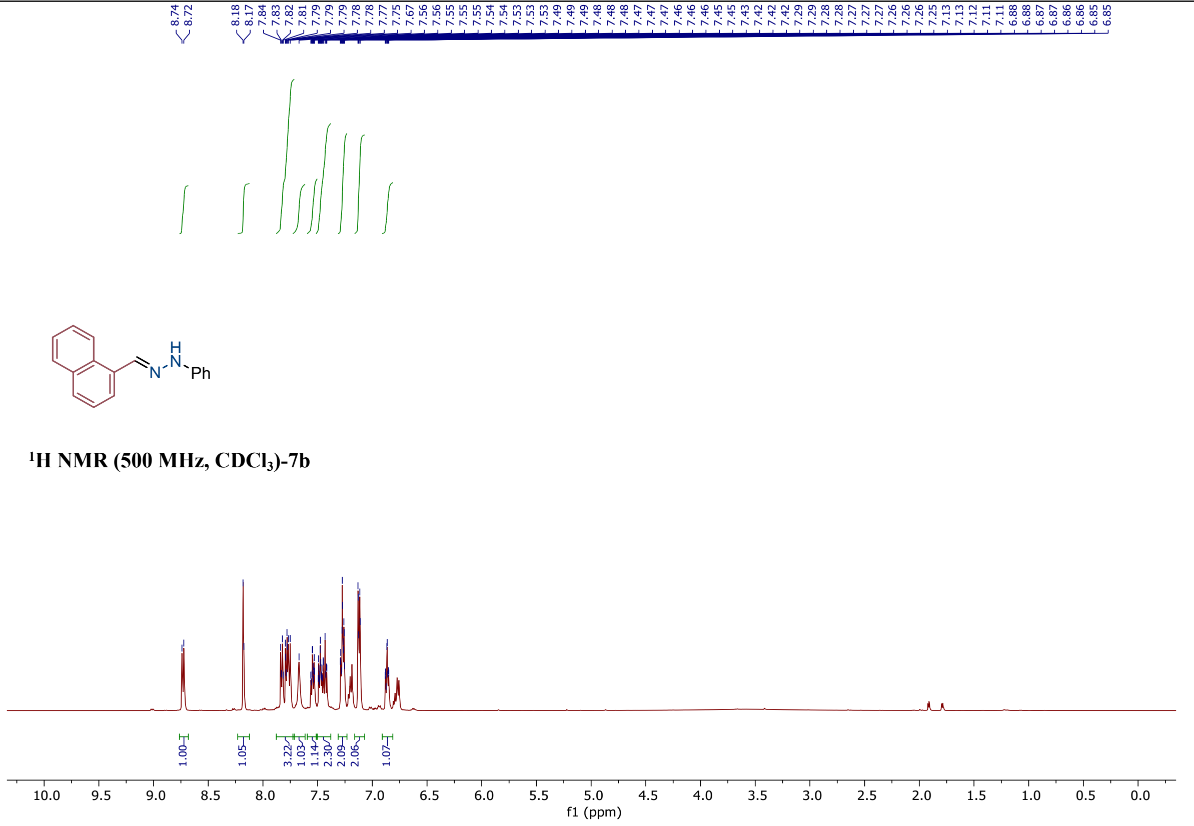

^{1}H NMR (500 MHz, $CDCl_3$)-7b

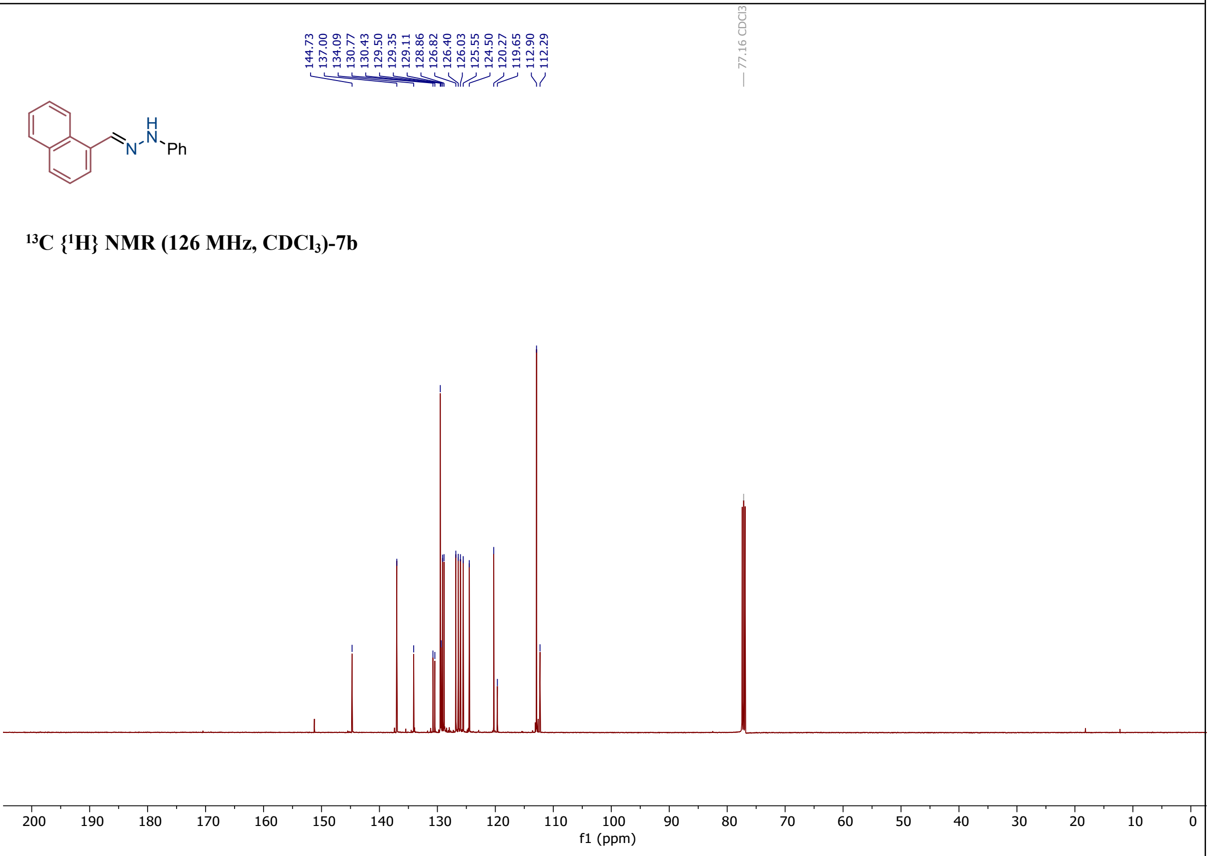

^{13}C {^{1}H} NMR (126 MHz, $CDCl_3$)-7b

(*E*)-1-(naphthalen-1-ylmethylene)-2-phenylhydrazine(7b)

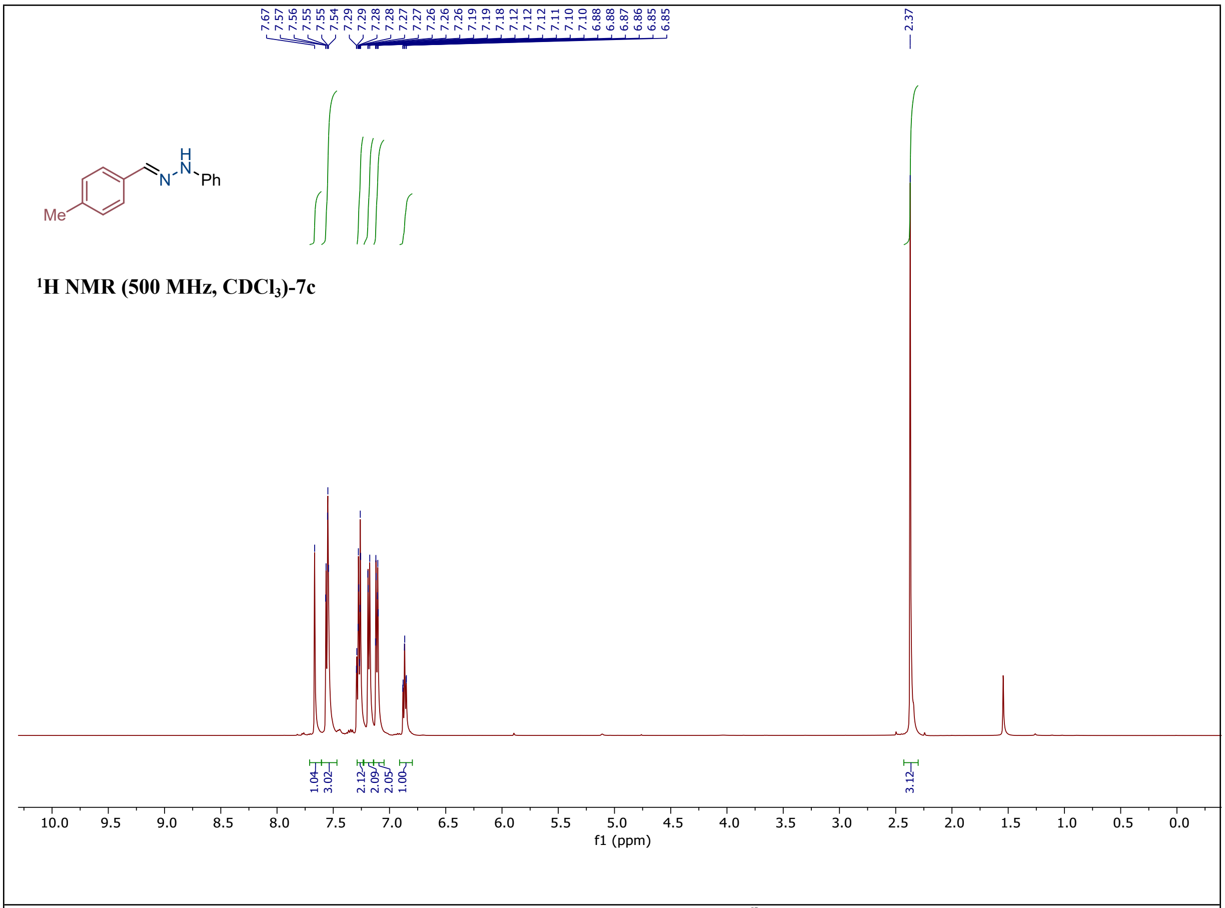

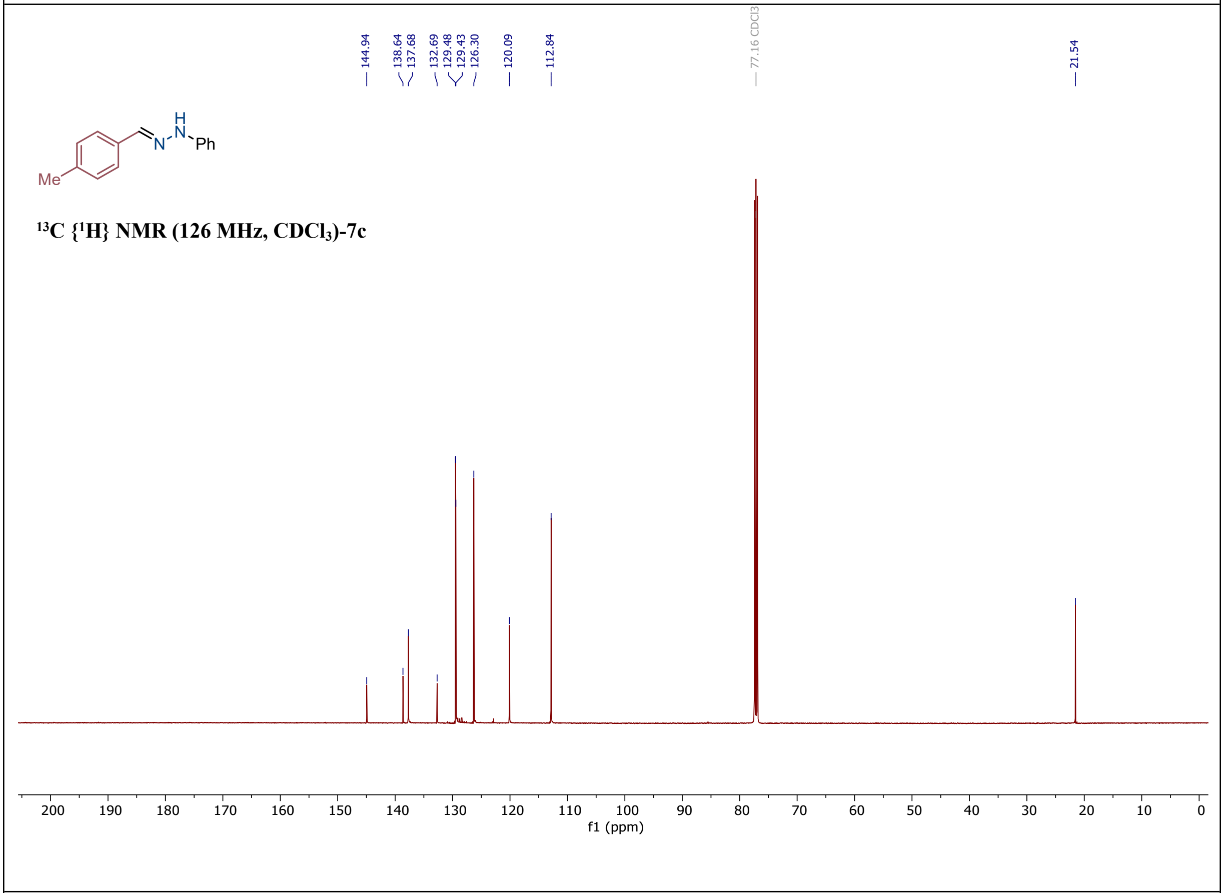

(E)-1-(4-methylbenzylidene)-2-phenylhydrazine(7c)

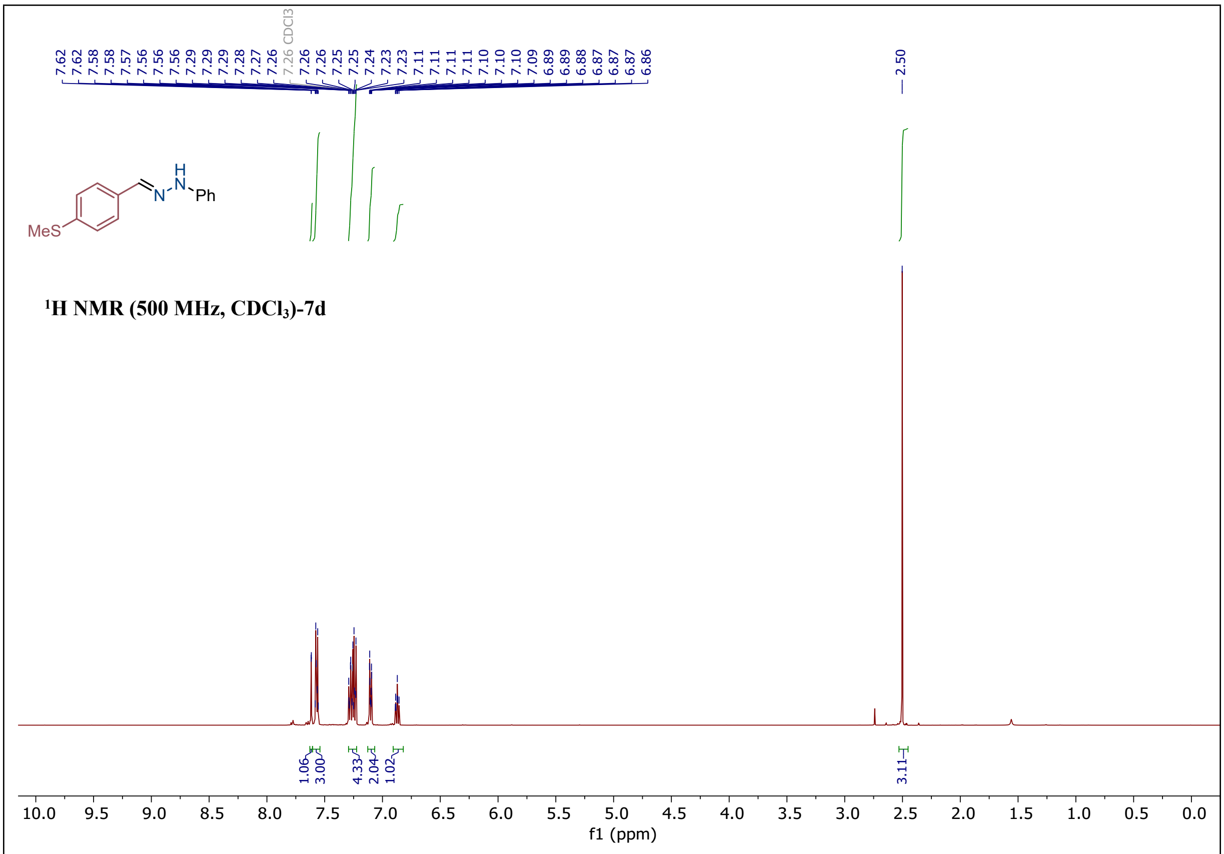

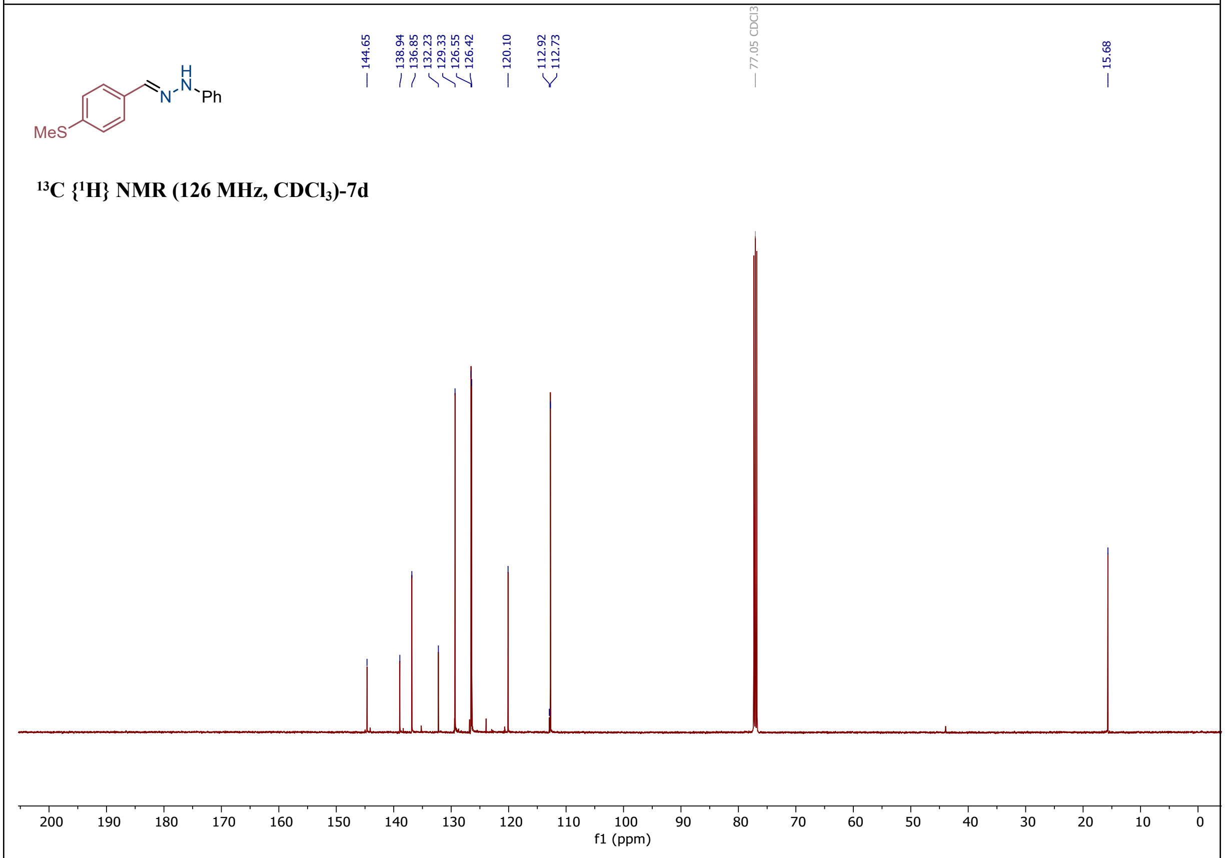

(*E*)-1-(4-(methylthio)benzylidene)-2-phenylhydrazine(7d)

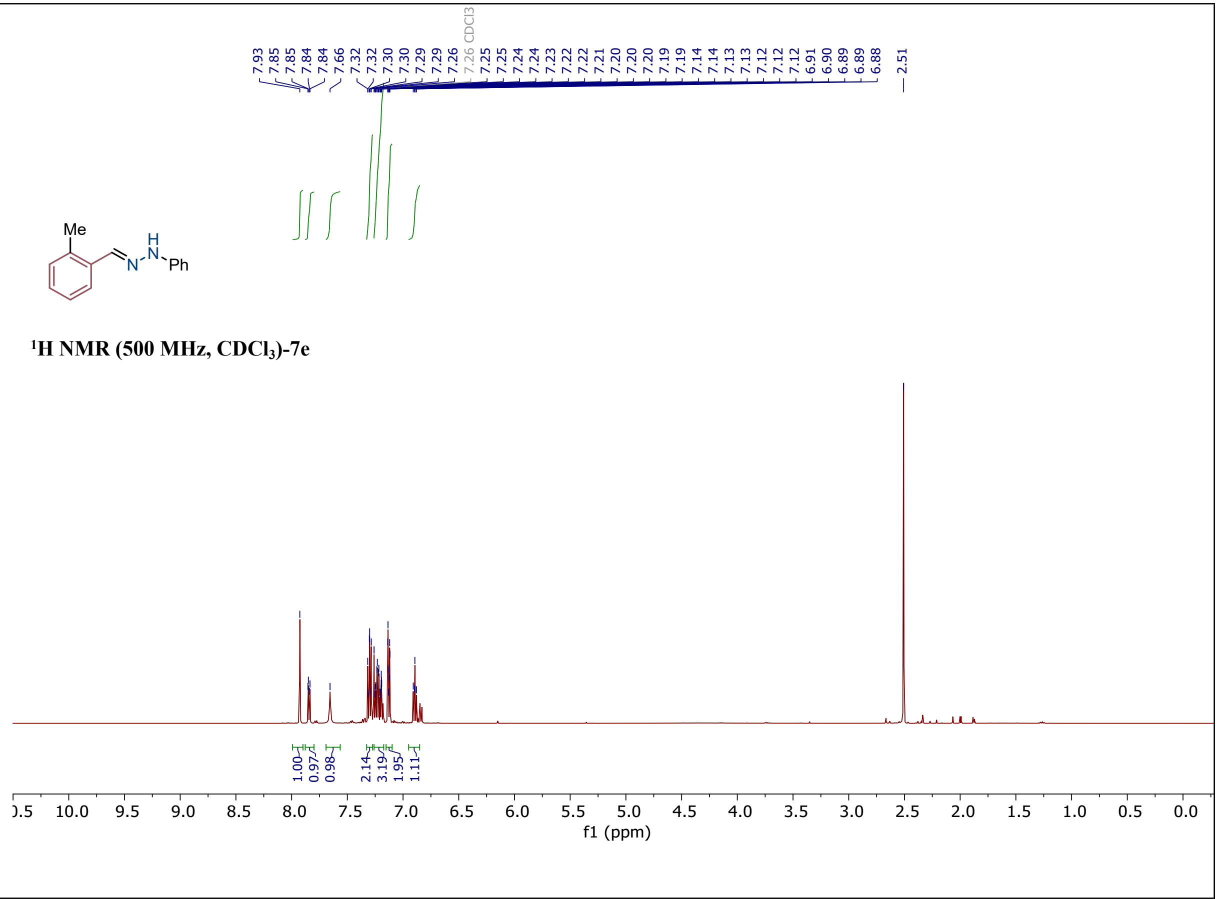

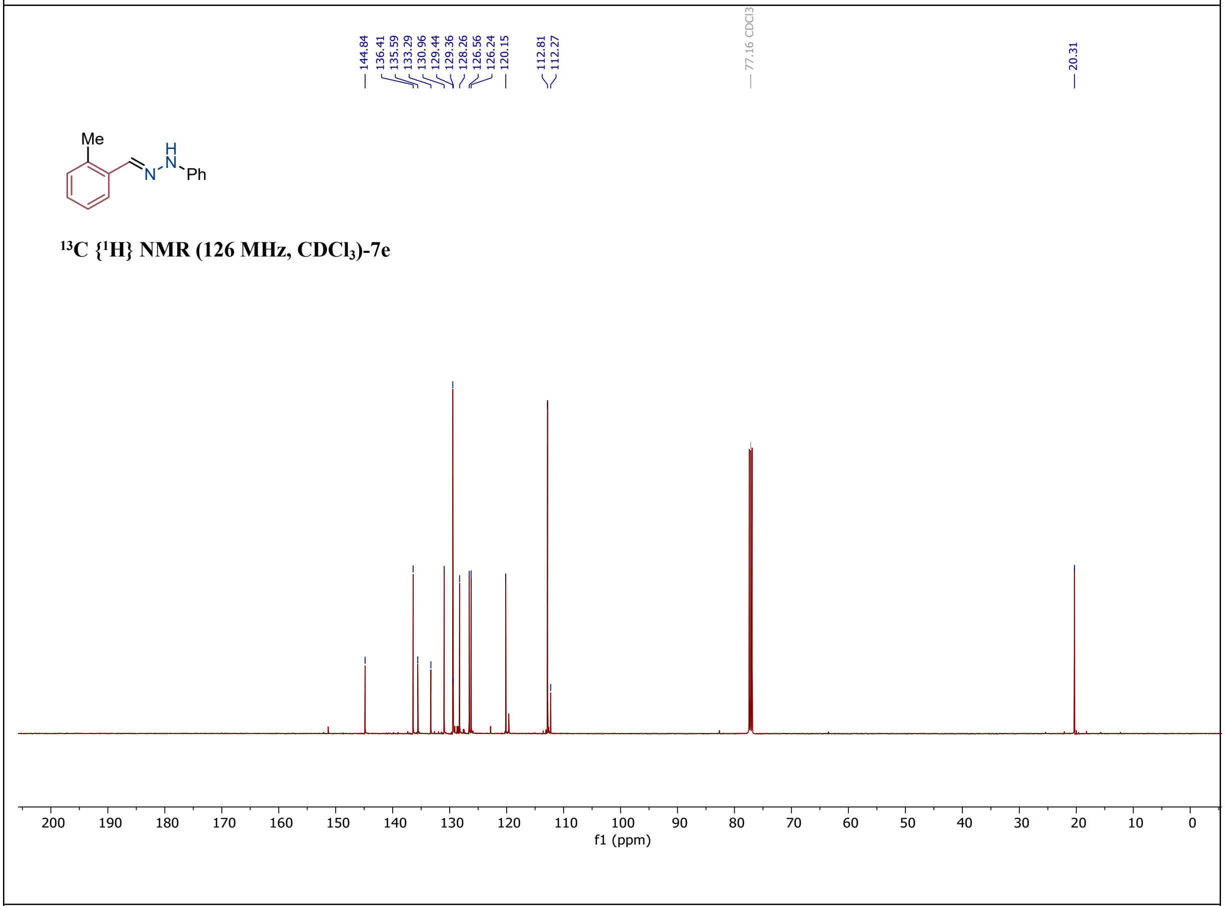

(*E*)-1-(2-methylbenzylidene)-2-phenylhydrazine(7e)

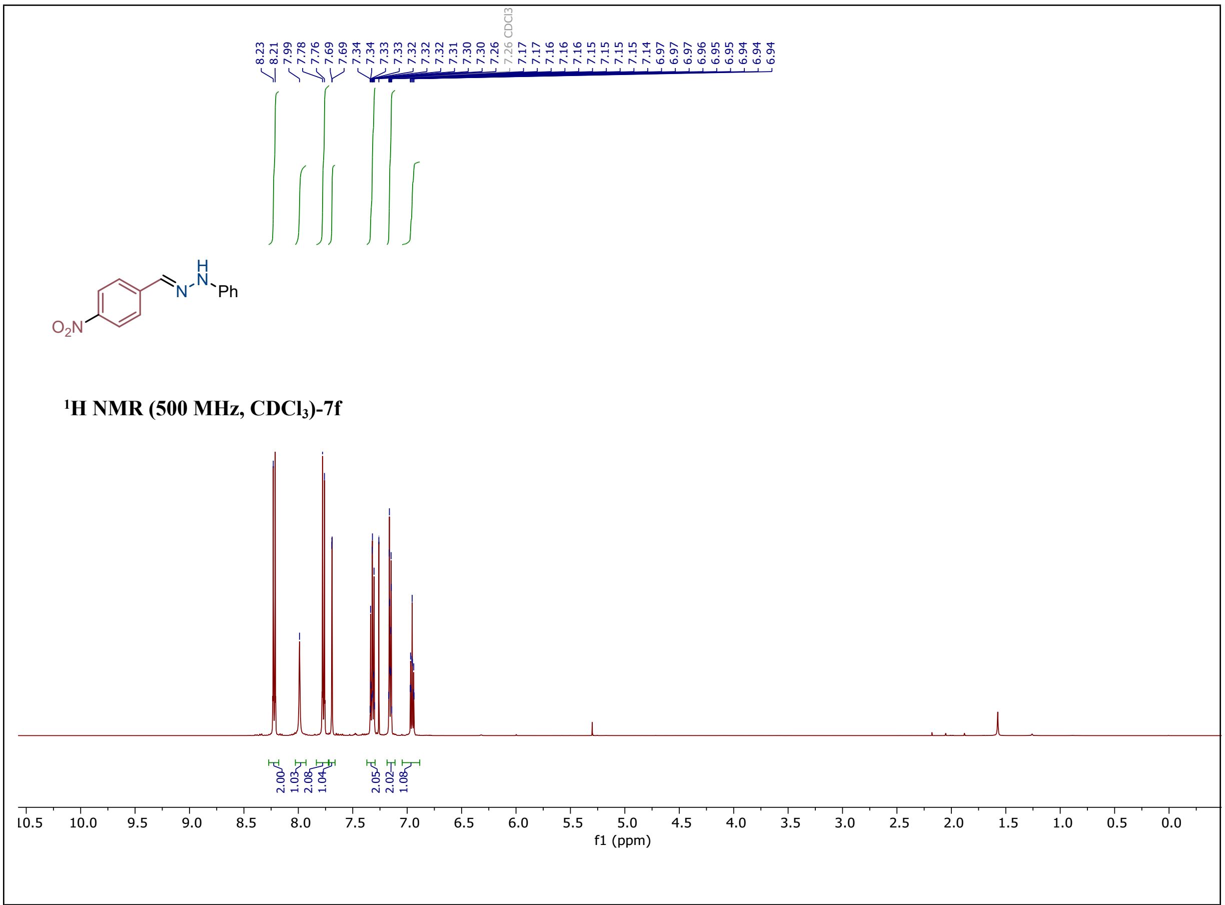

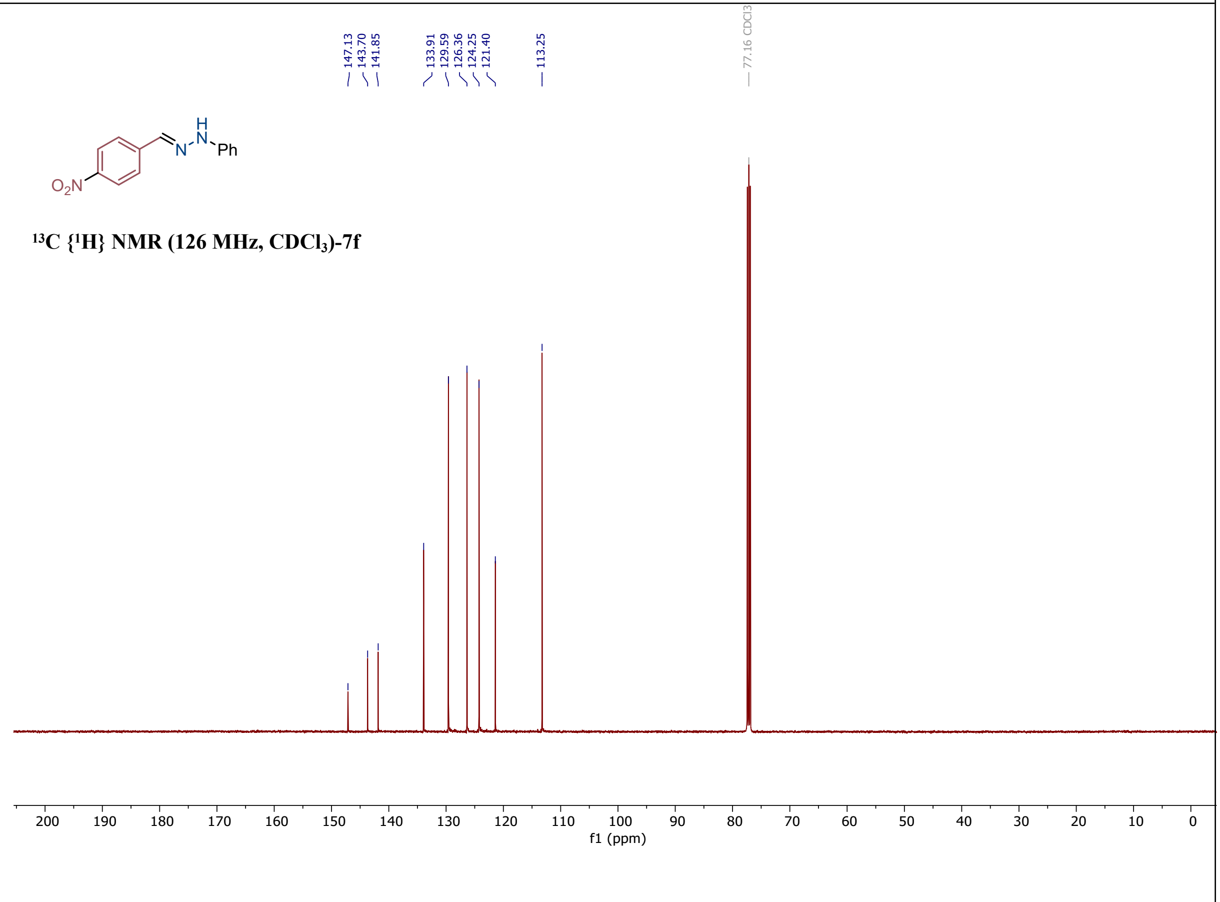

(*E*)-1-(4-nitrobenzylidene)-2-phenylhydrazine (7f)

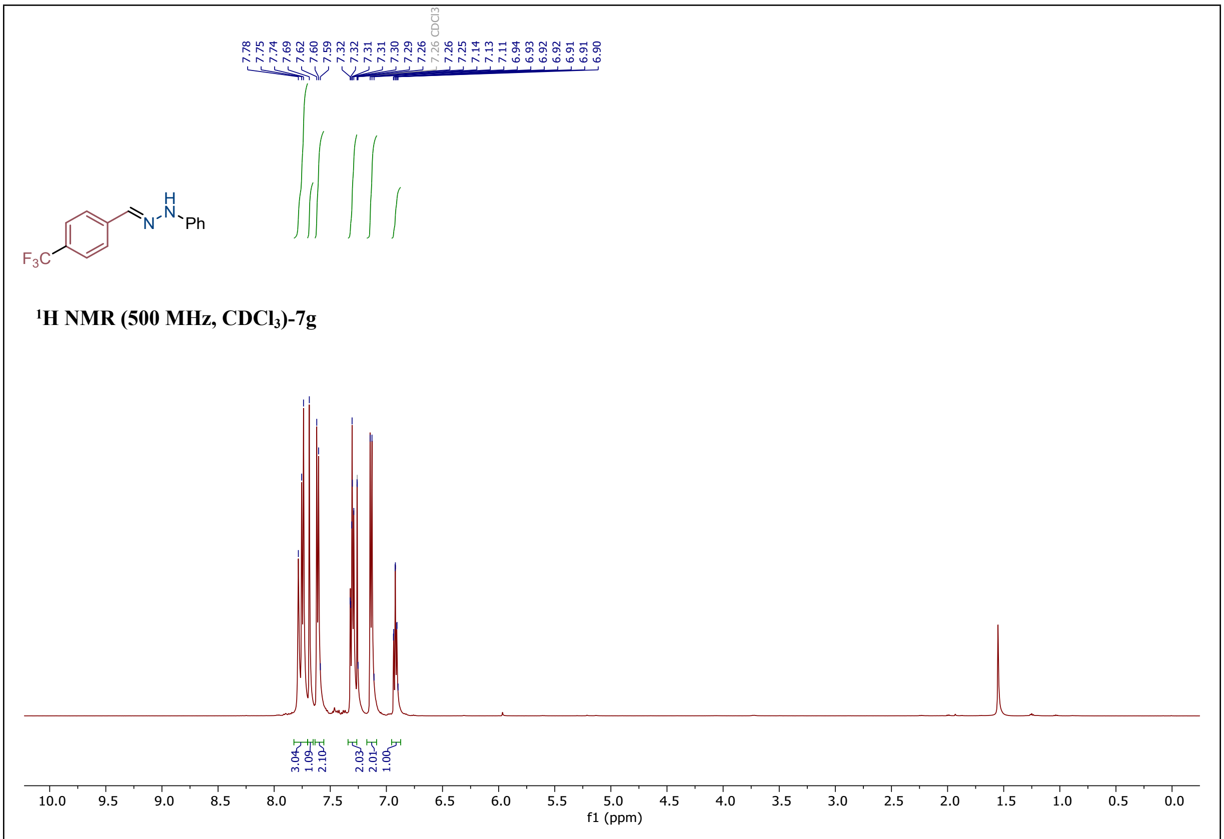

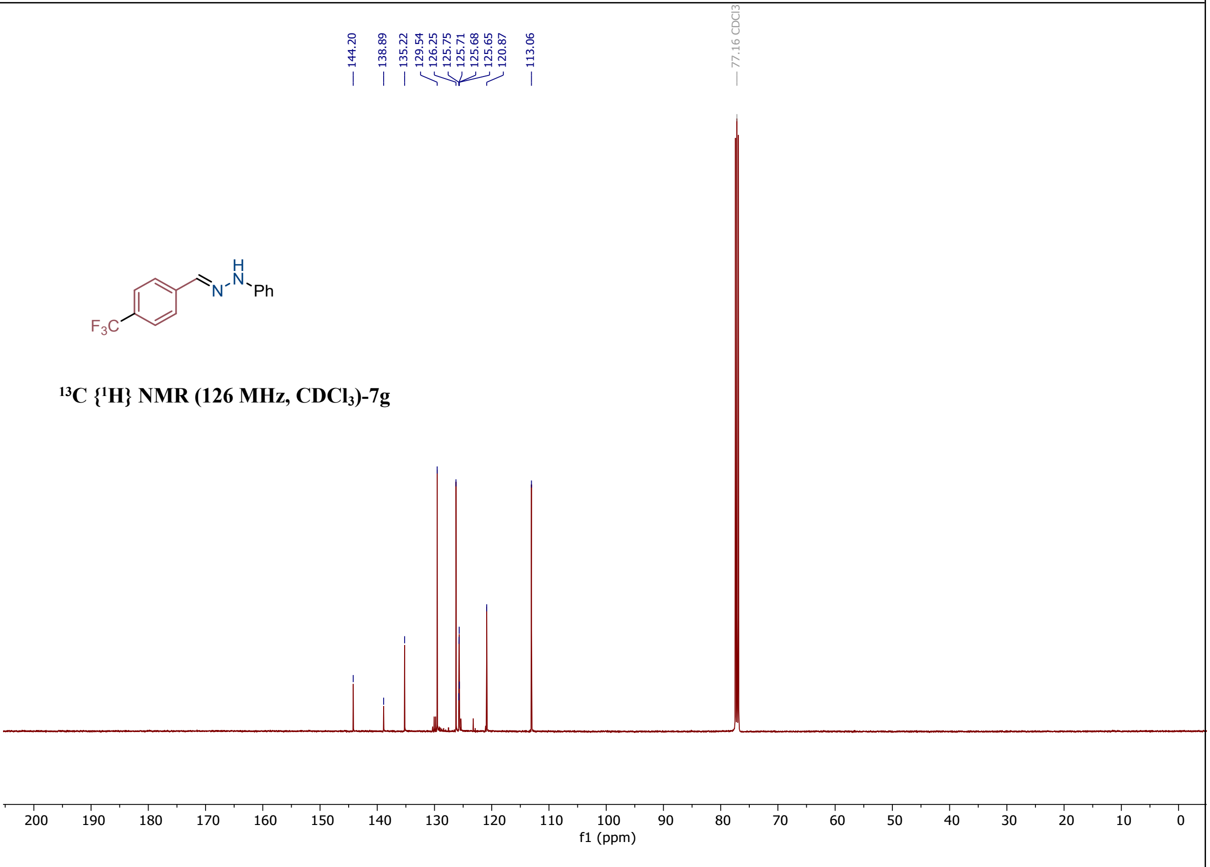

(*E*)-1-phenyl-2-(4-(trifluoromethyl)benzylidene)hydrazine (7g)

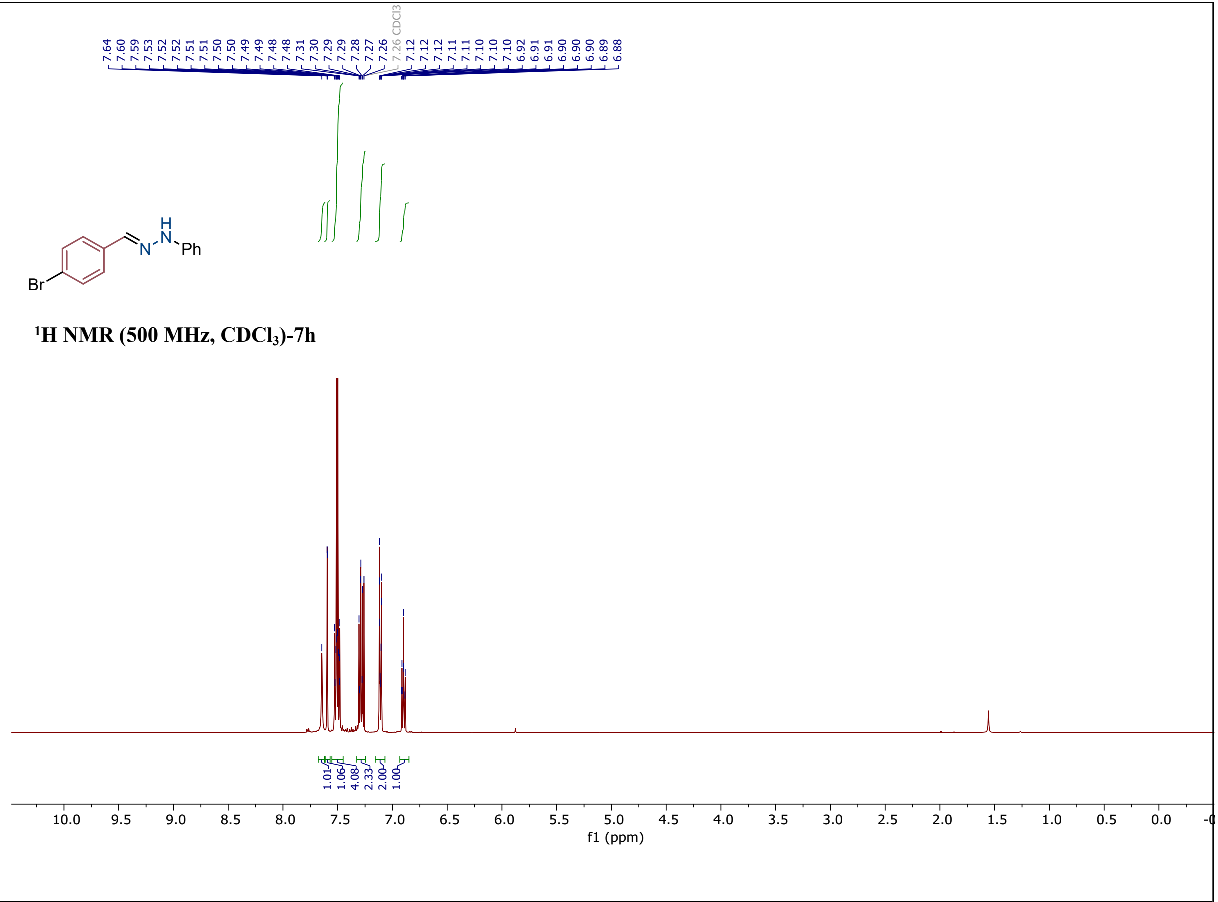

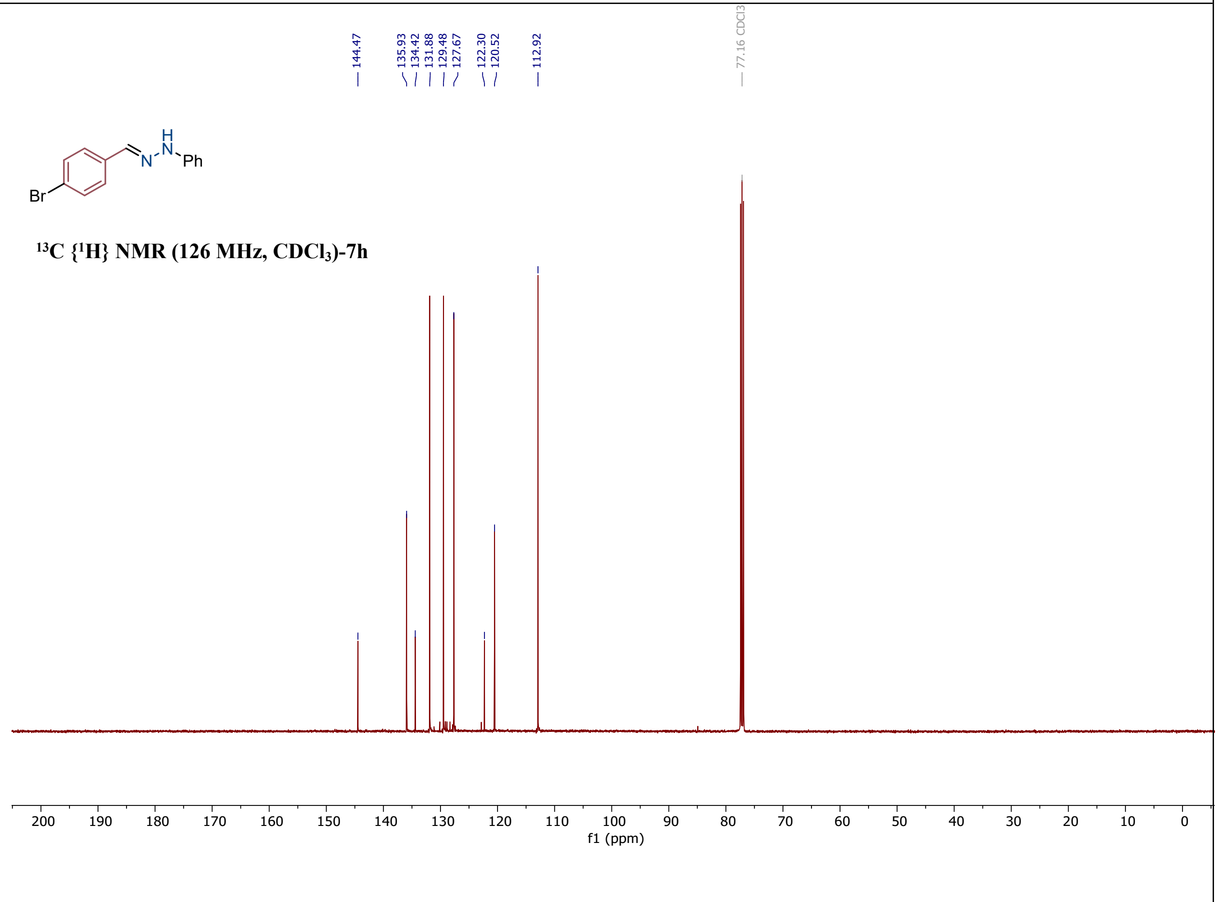

(*E*)-1-(4-bromobenzylidene)-2-phenylhydrazine(7h)

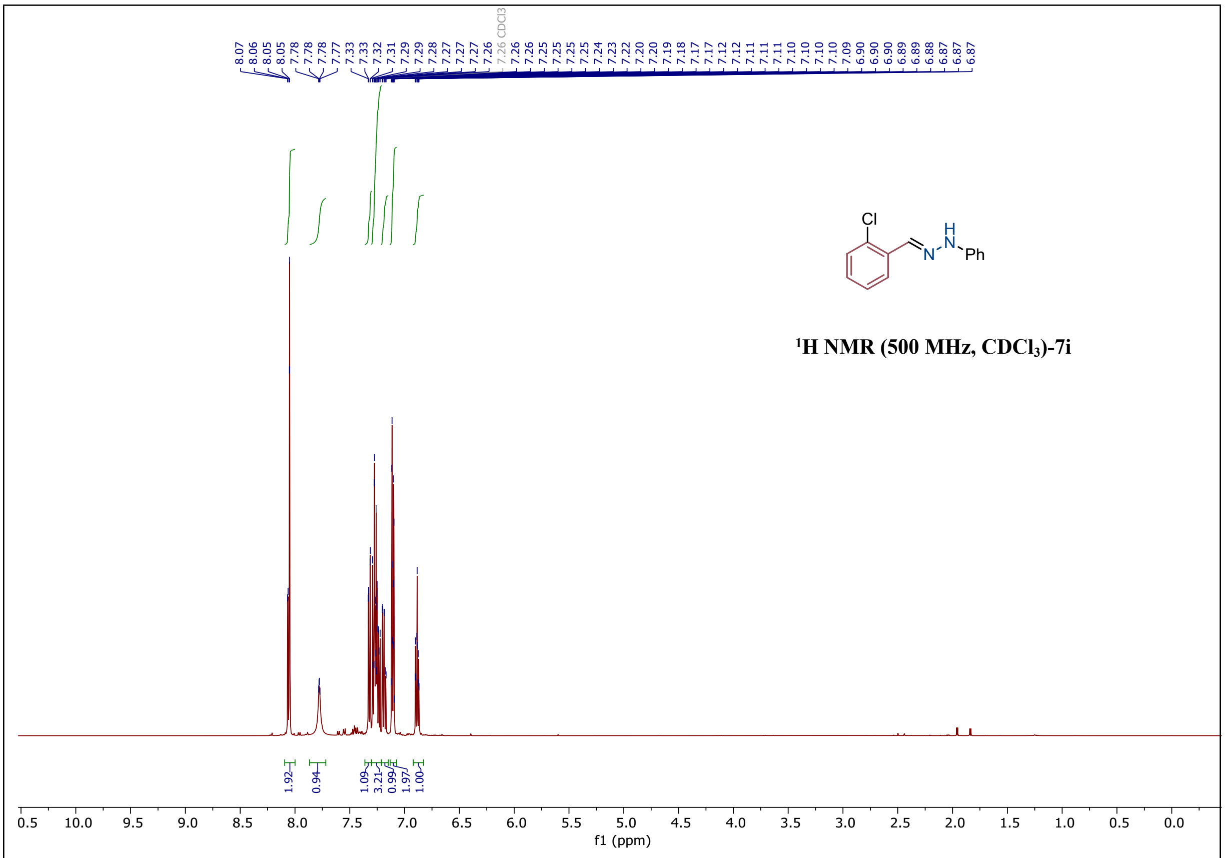

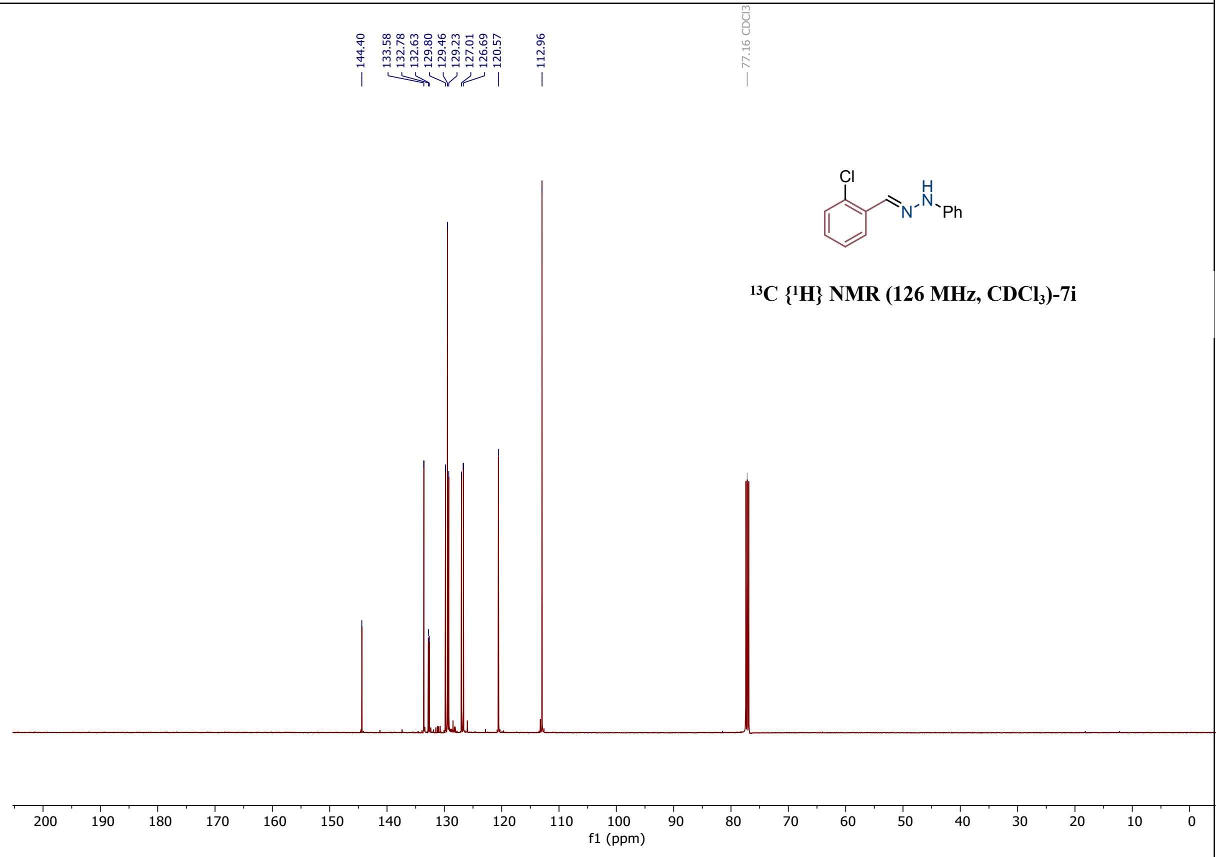

(*E*)-1-(2-chlorobenzylidene)-2-phenylhydrazine(7i)

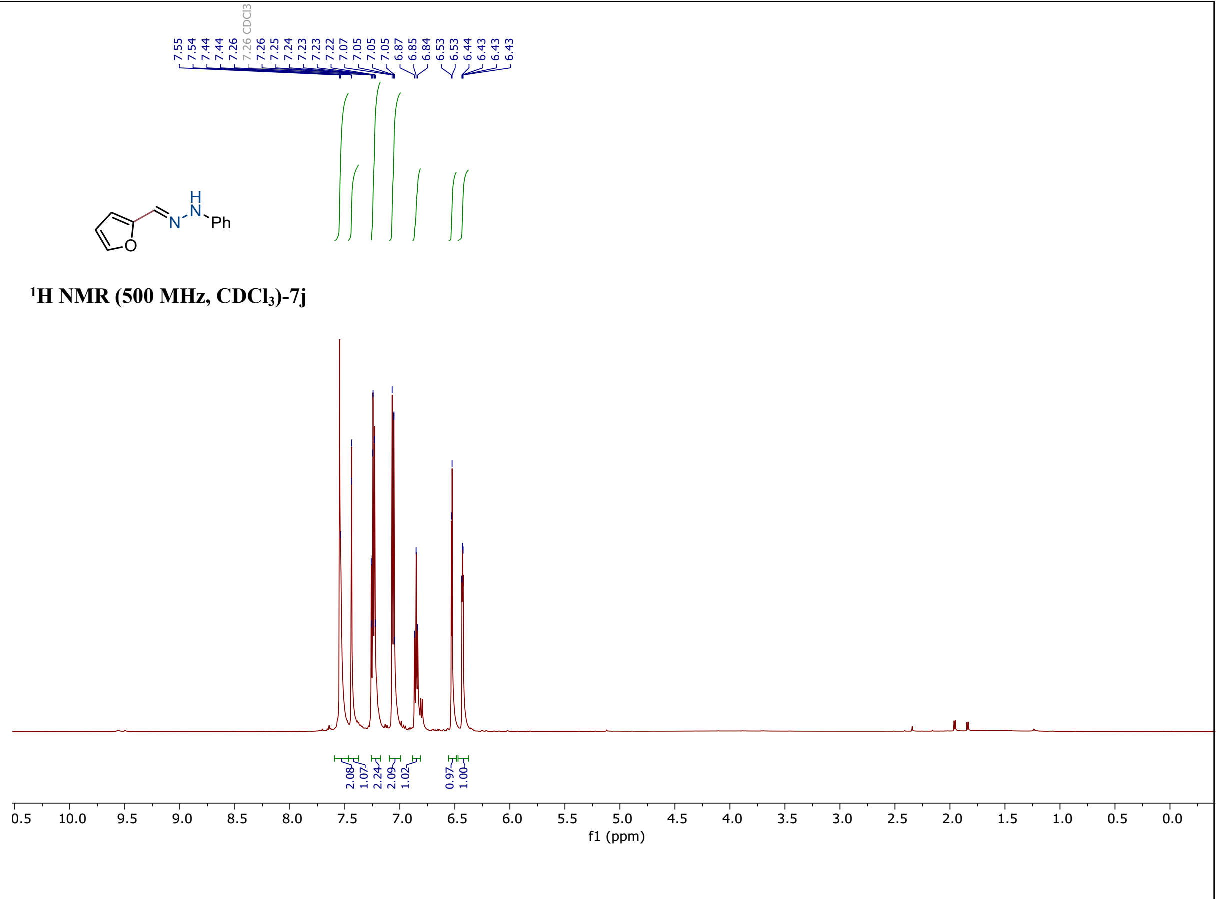

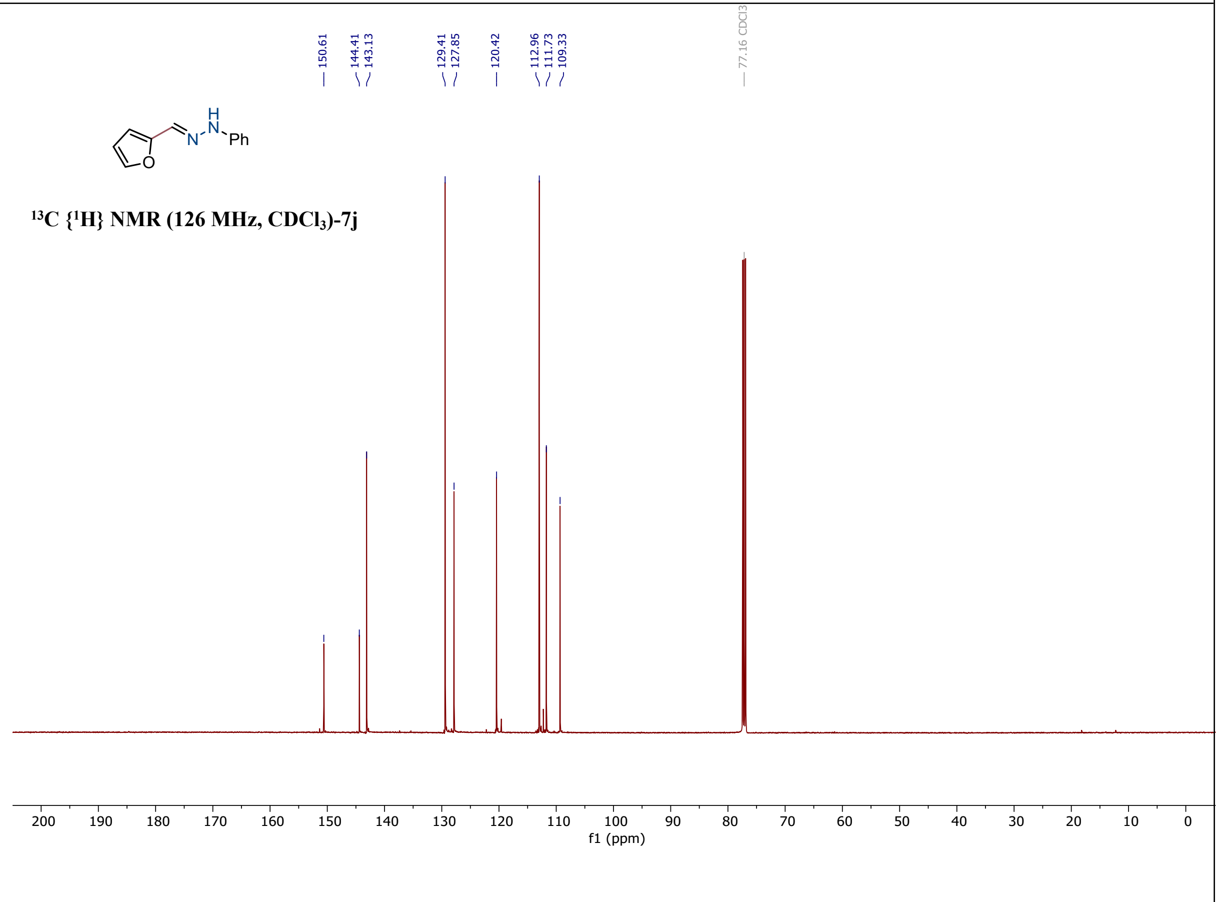

(*E*)-1-(furan-2-ylmethylene)-2-phenylhydrazine(7j)

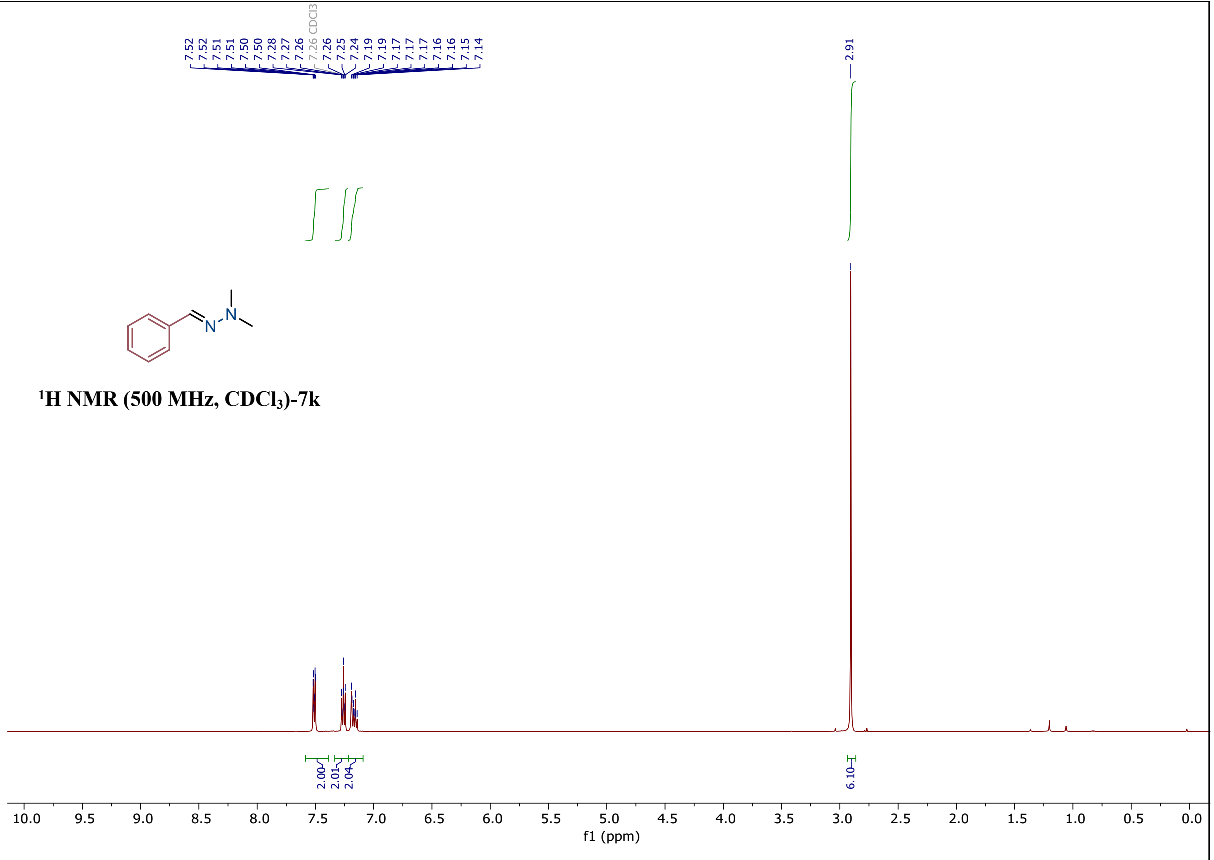

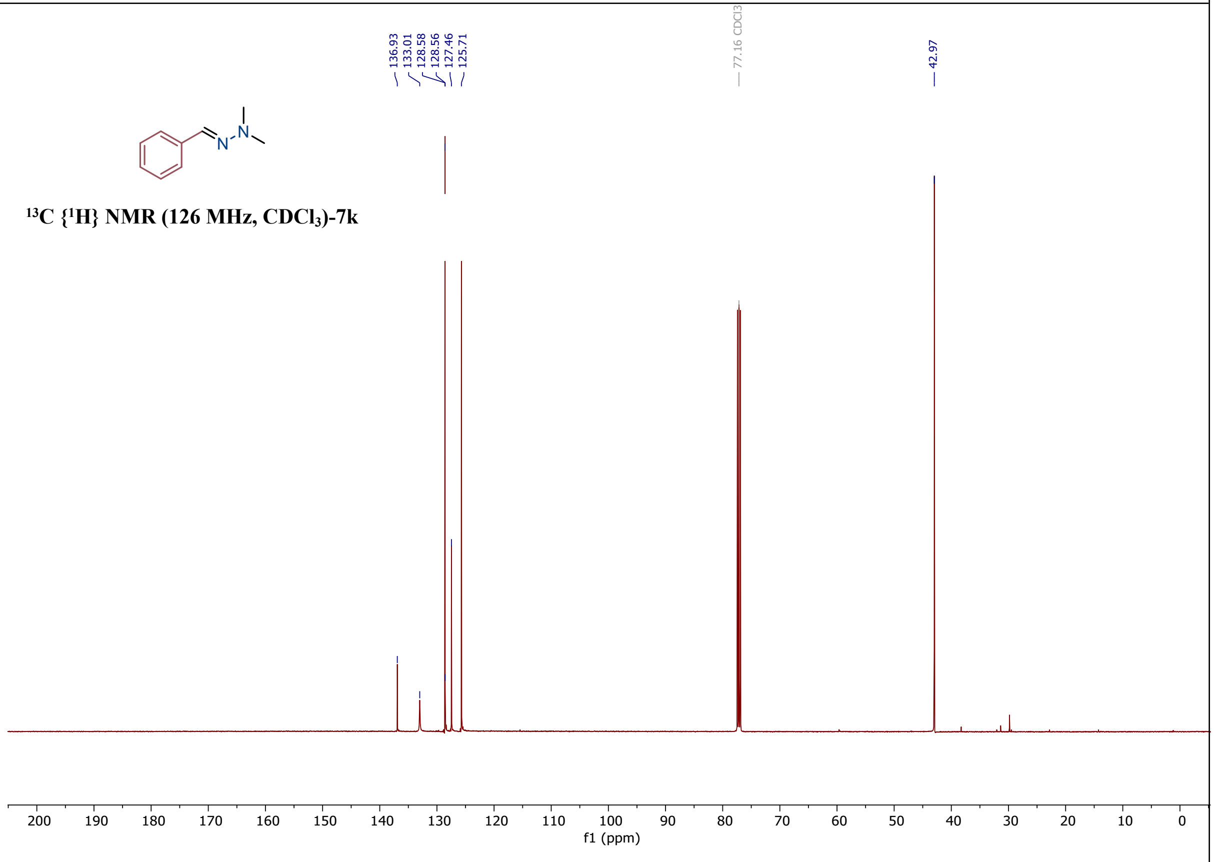

(*E*)-2-benzylidene-1,1-dimethylhydrazine(7k)

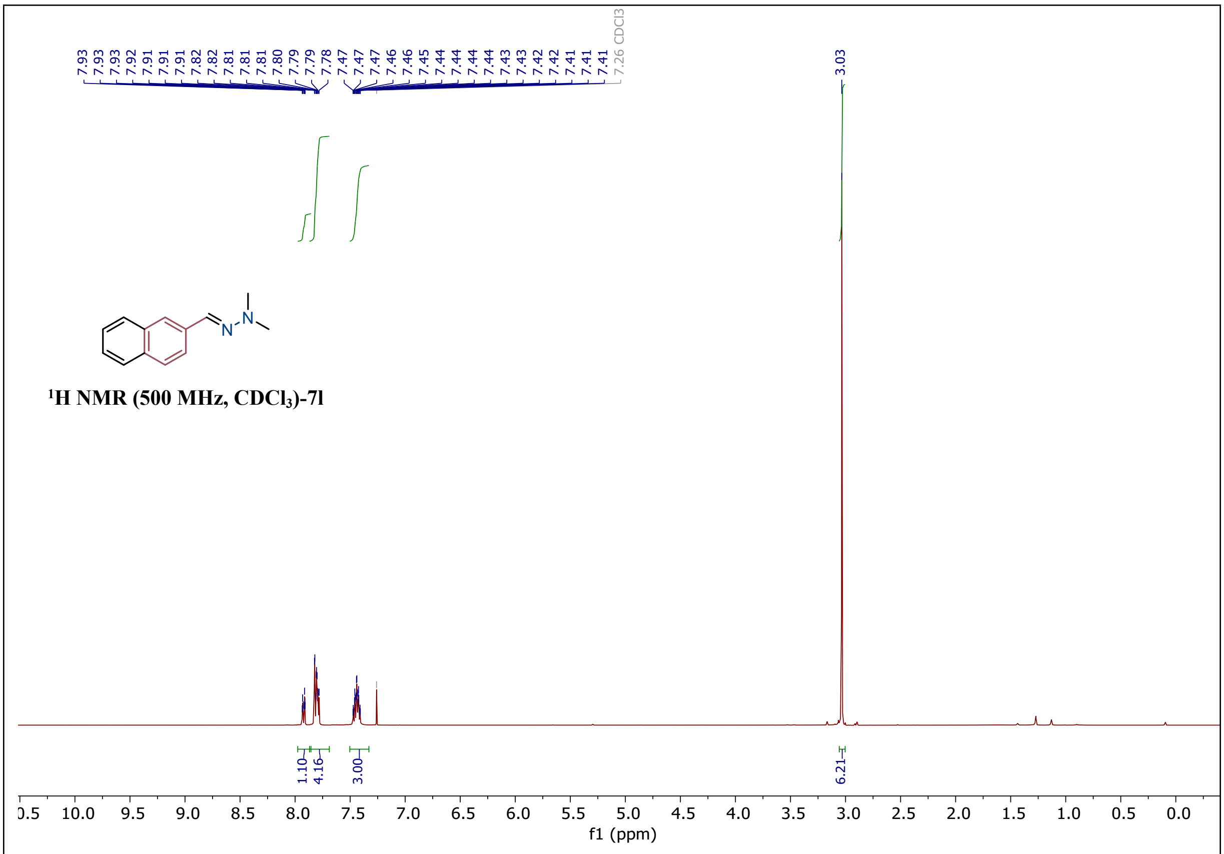

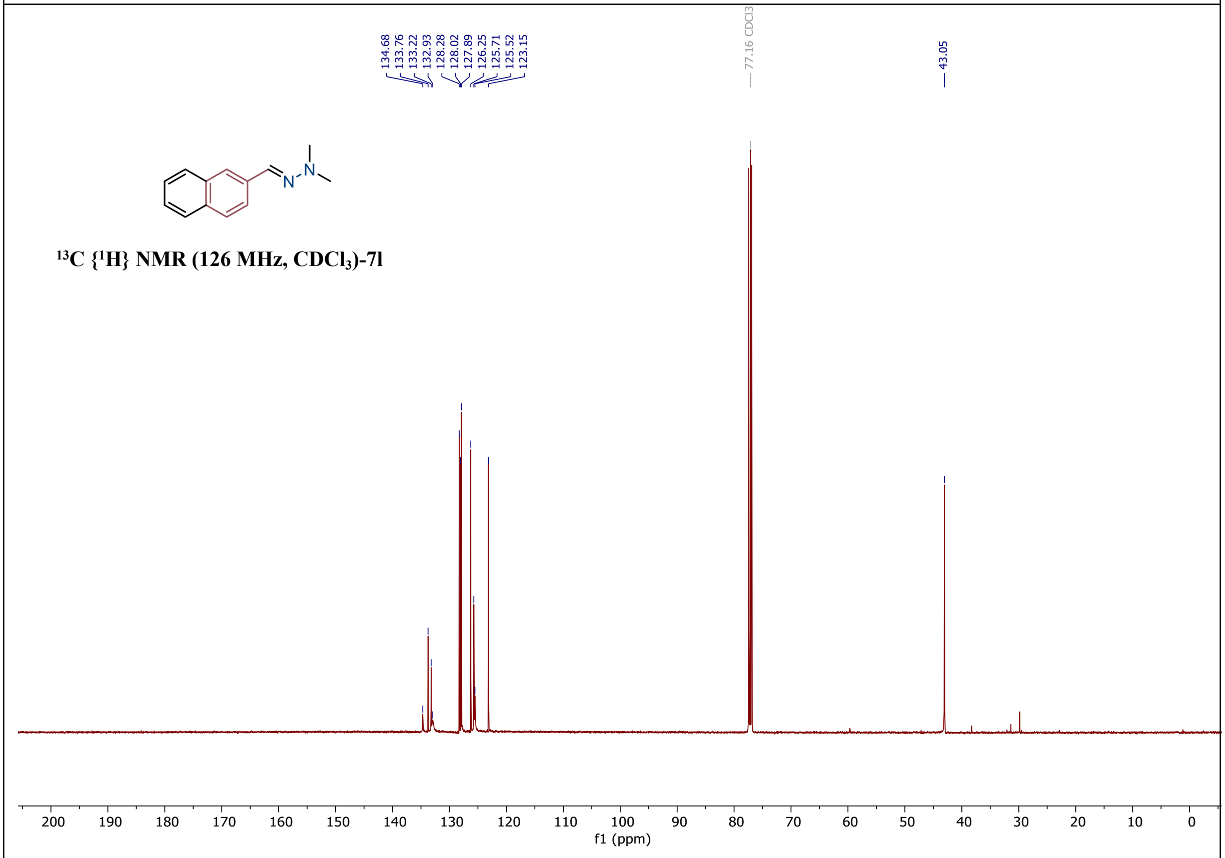

(*E*)-1,1-dimethyl-2-(naphthalen-2-ylmethylene)hydrazine(7l)

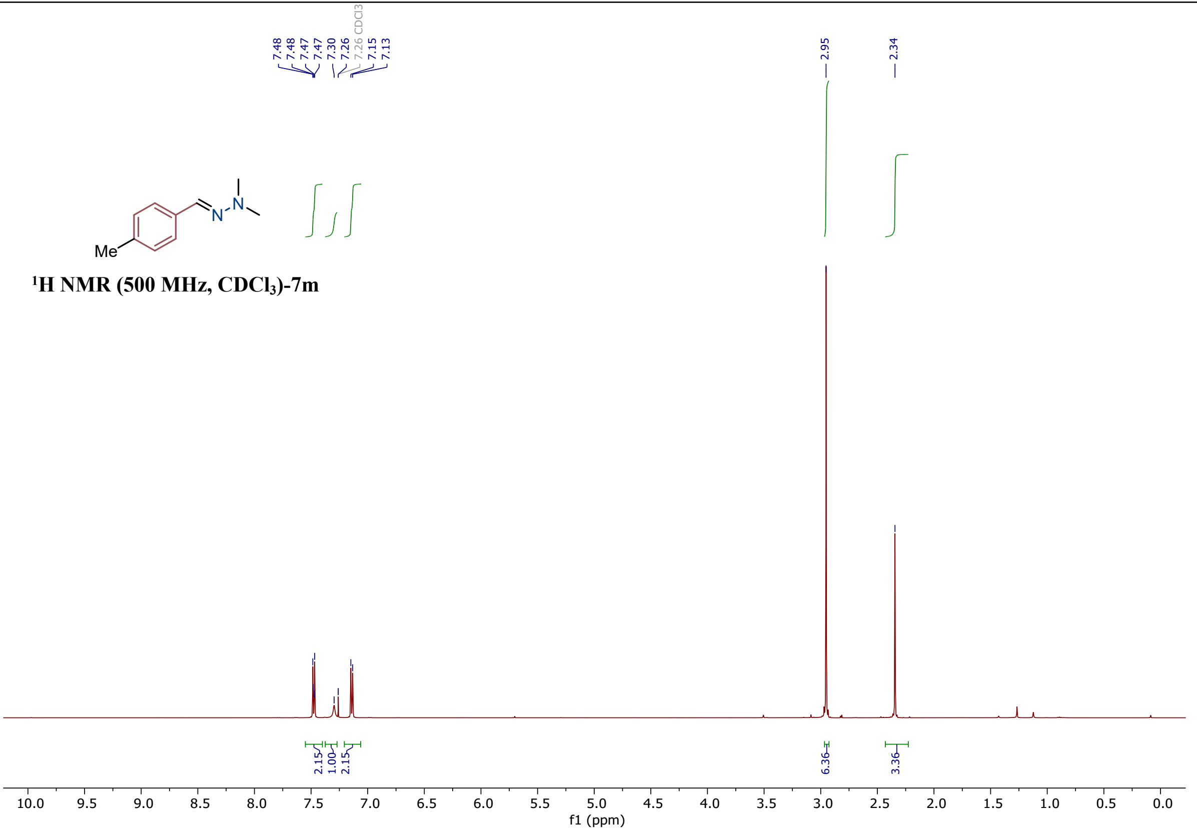

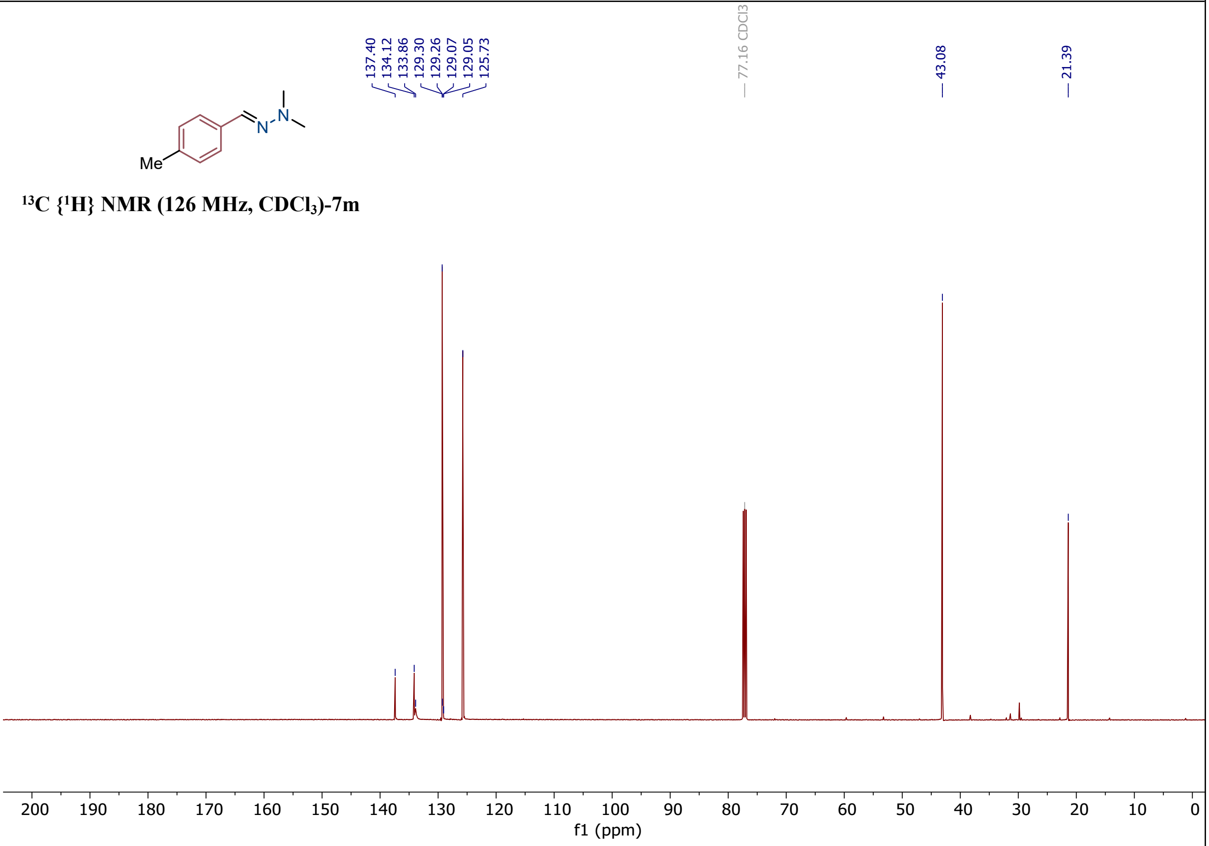

(*E*)-1,1-dimethyl-2-(4-methylbenzylidene)hydrazine(7m)

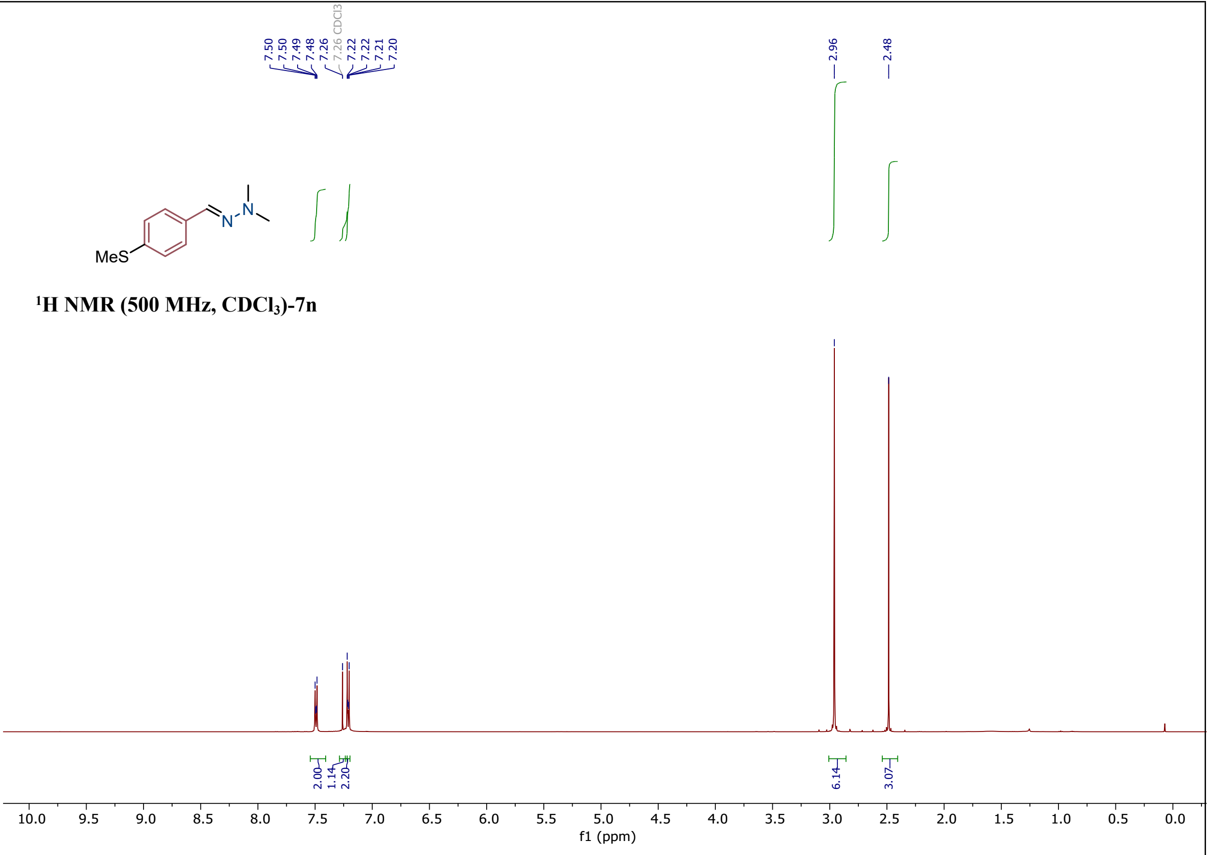

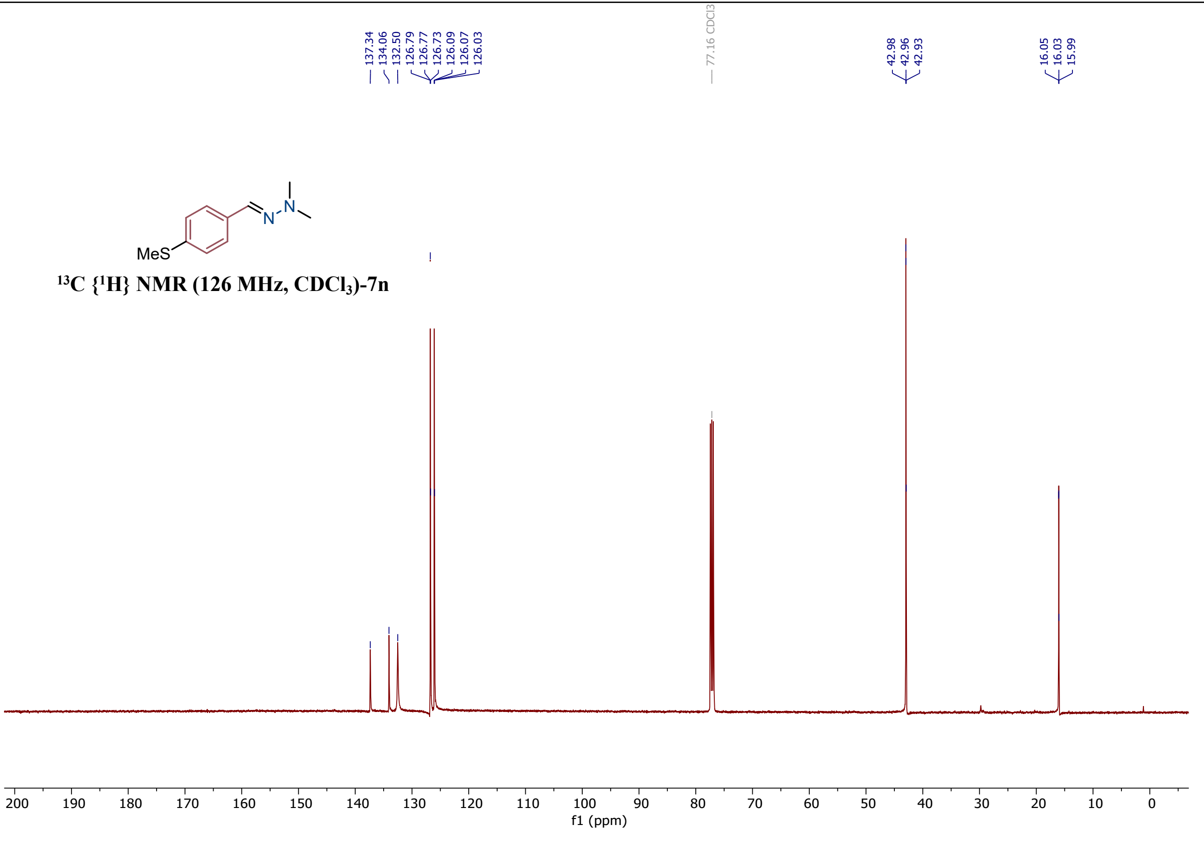

(*E*)-1,1-dimethyl-2-(4-(methylthio)benzylidene)hydrazine(7n)

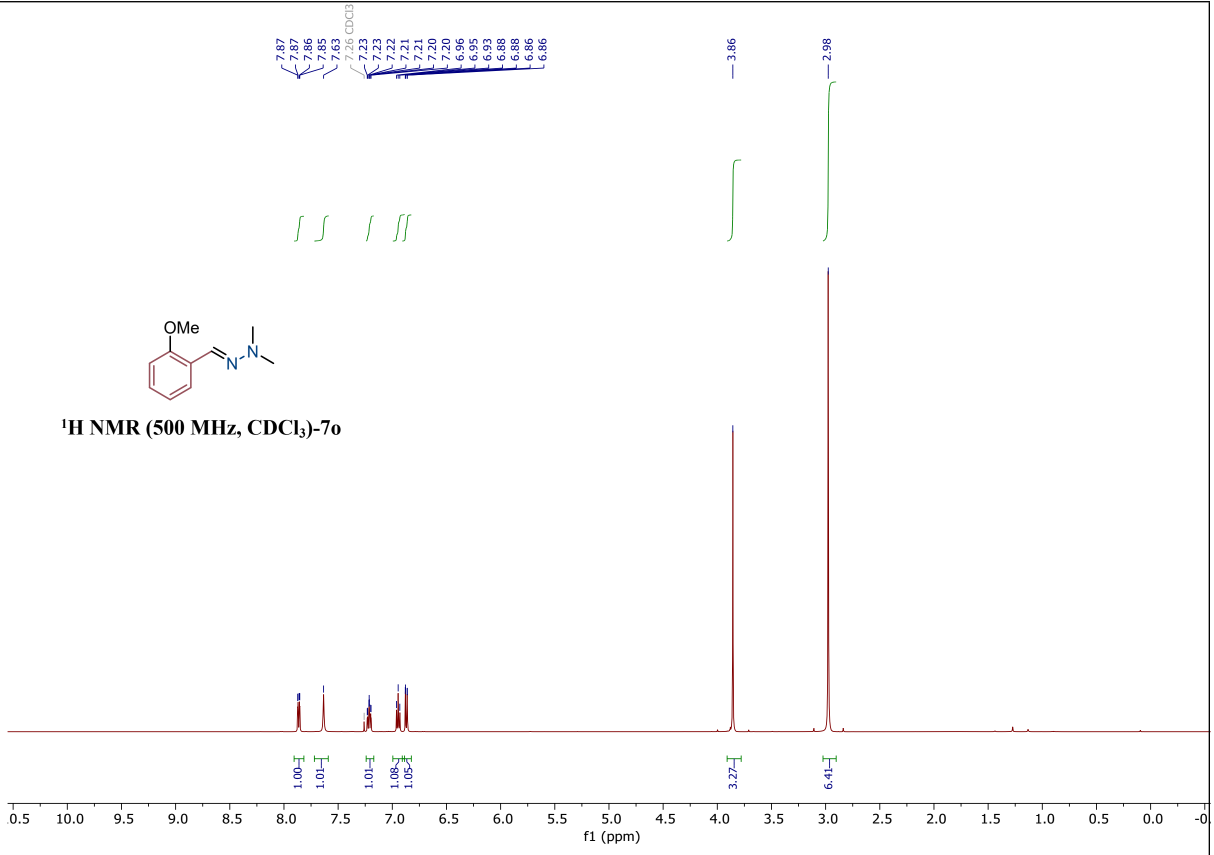

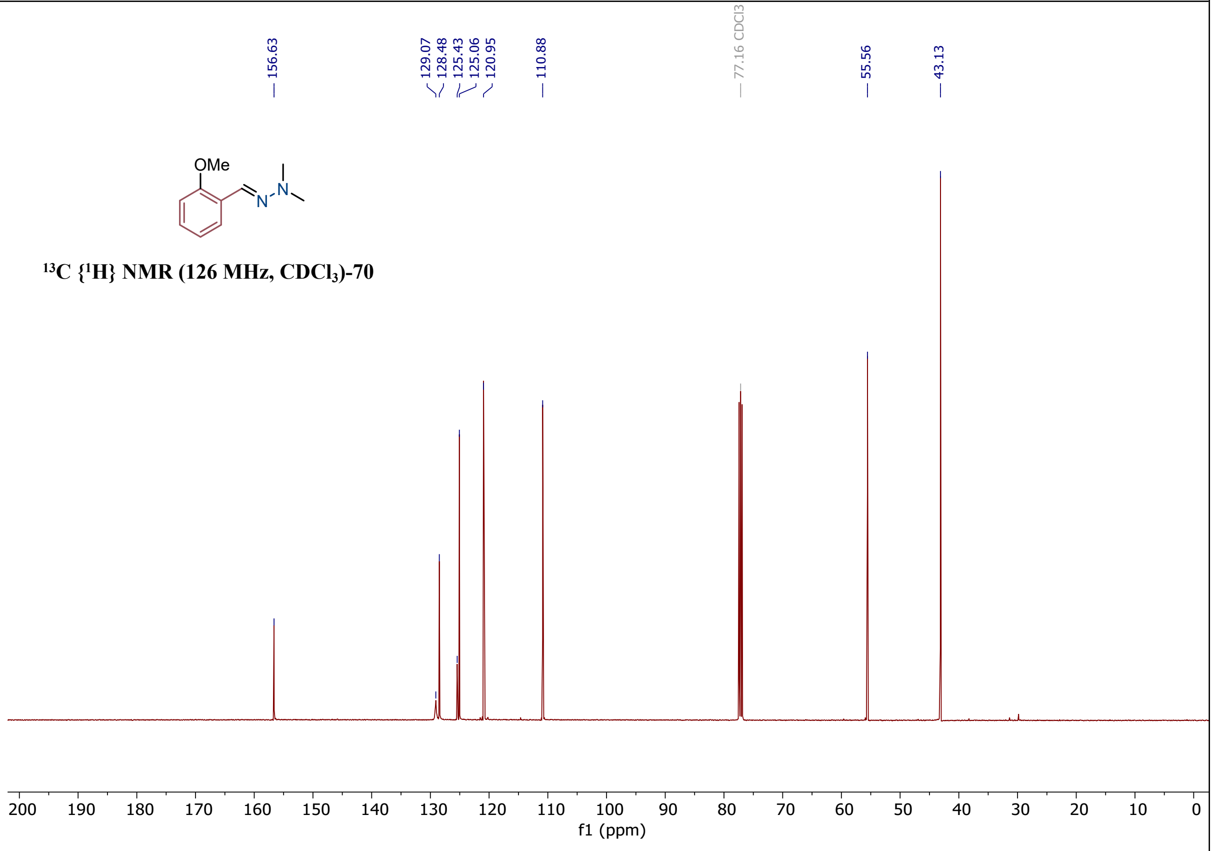

(E)-2-(2-methoxybenzylidene)-1,1-dimethylhydrazine(7o)

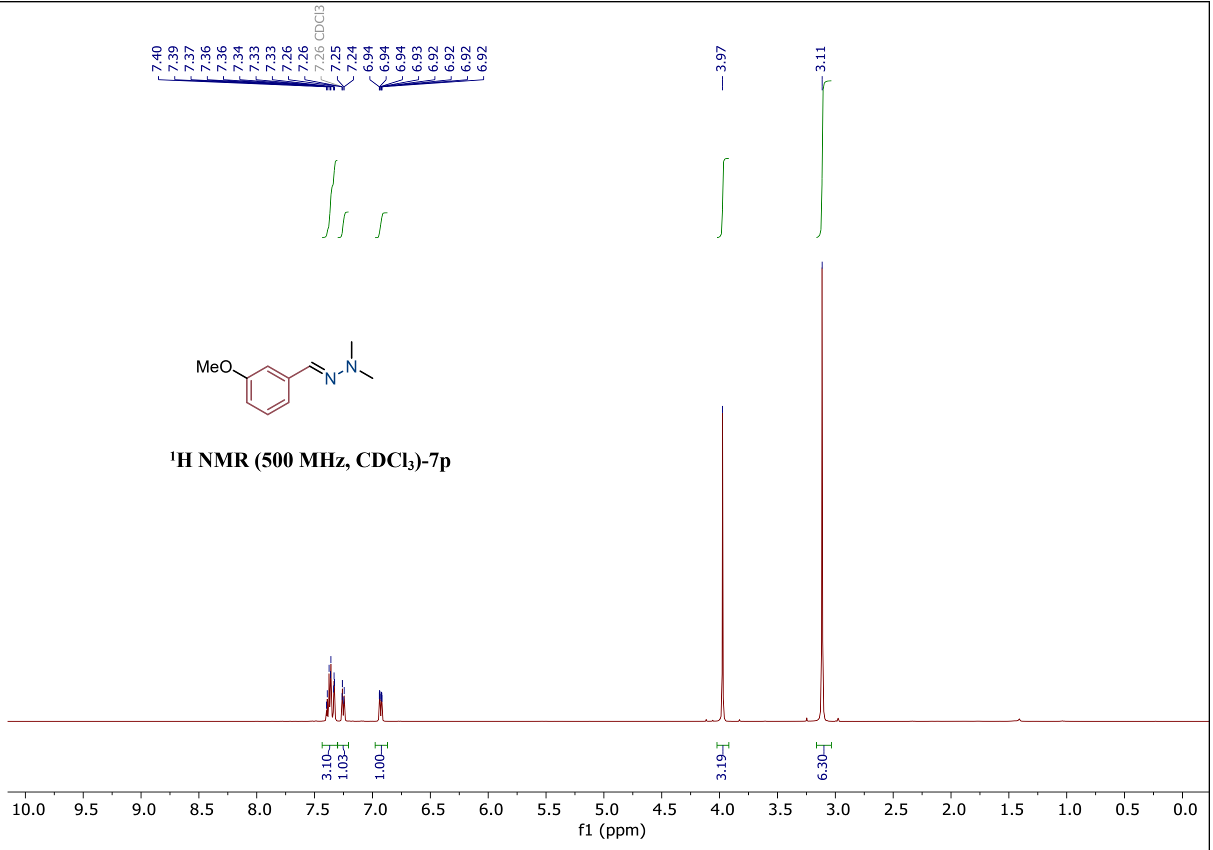

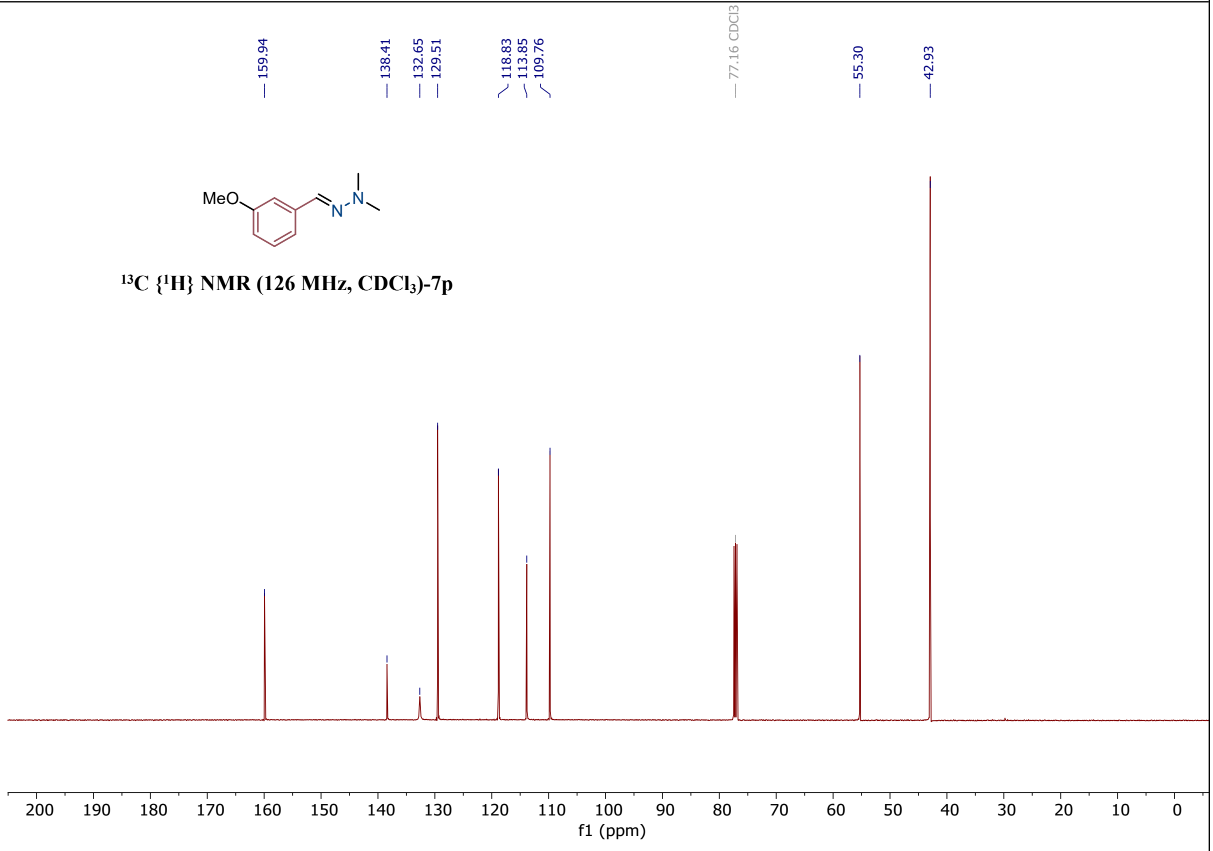

(*E*)-2-(3-methoxybenzylidene)-1,1-dimethylhydrazine(7p)

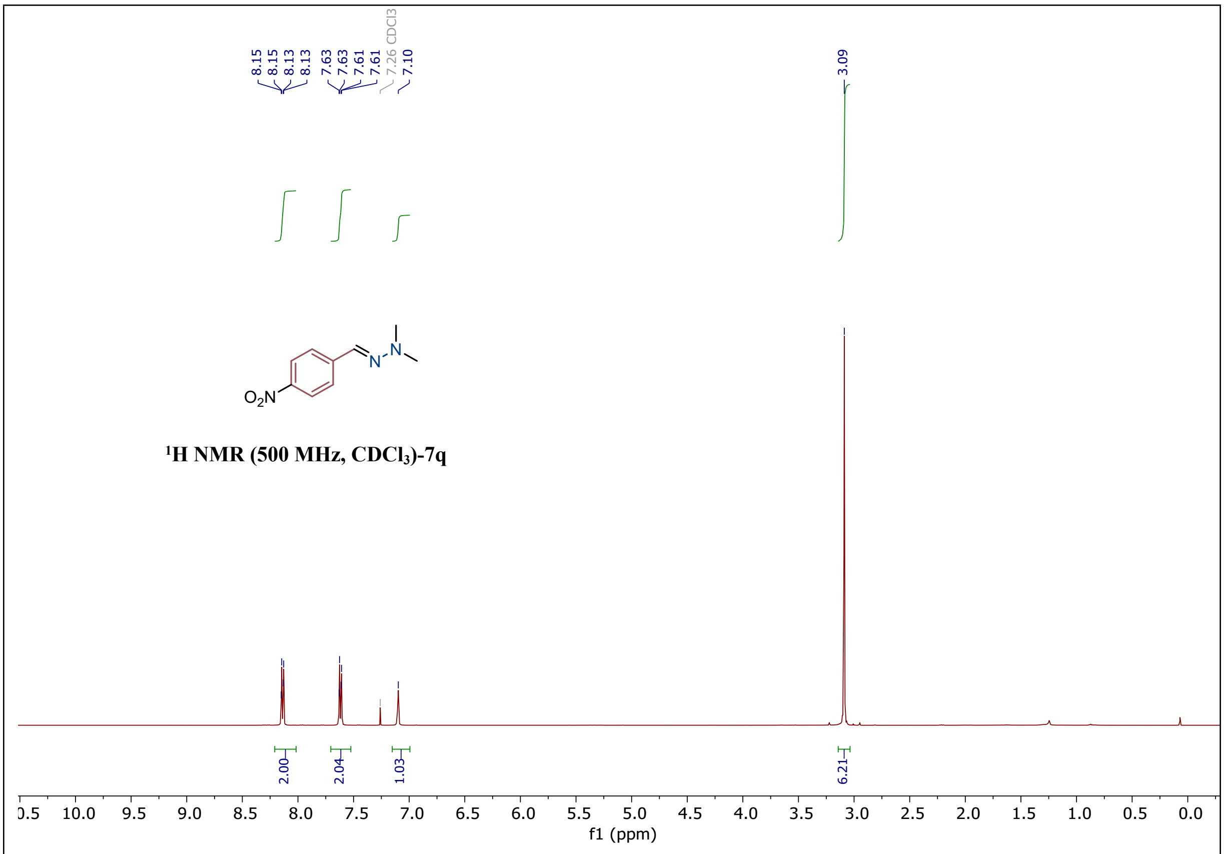

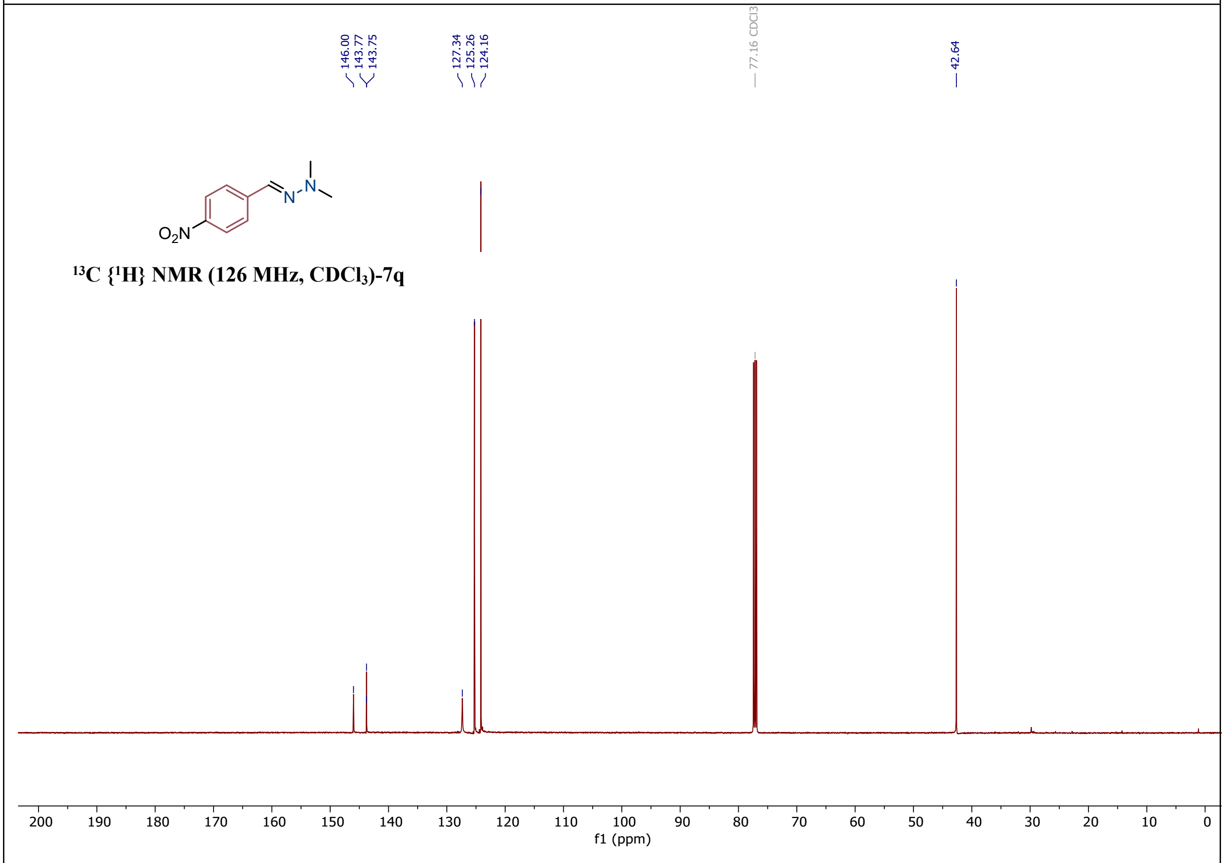

(*E*)-1,1-dimethyl-2-(4-nitrobenzylidene)hydrazine(7q)

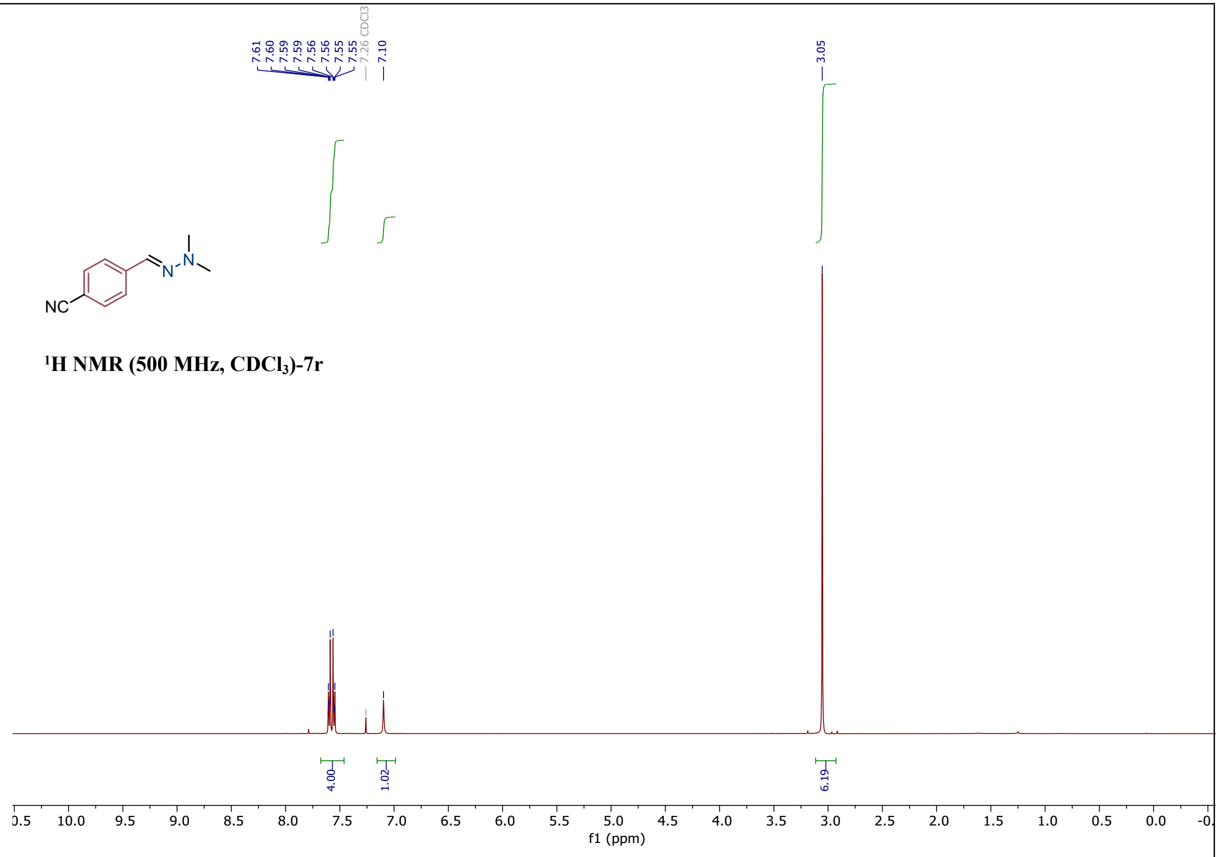

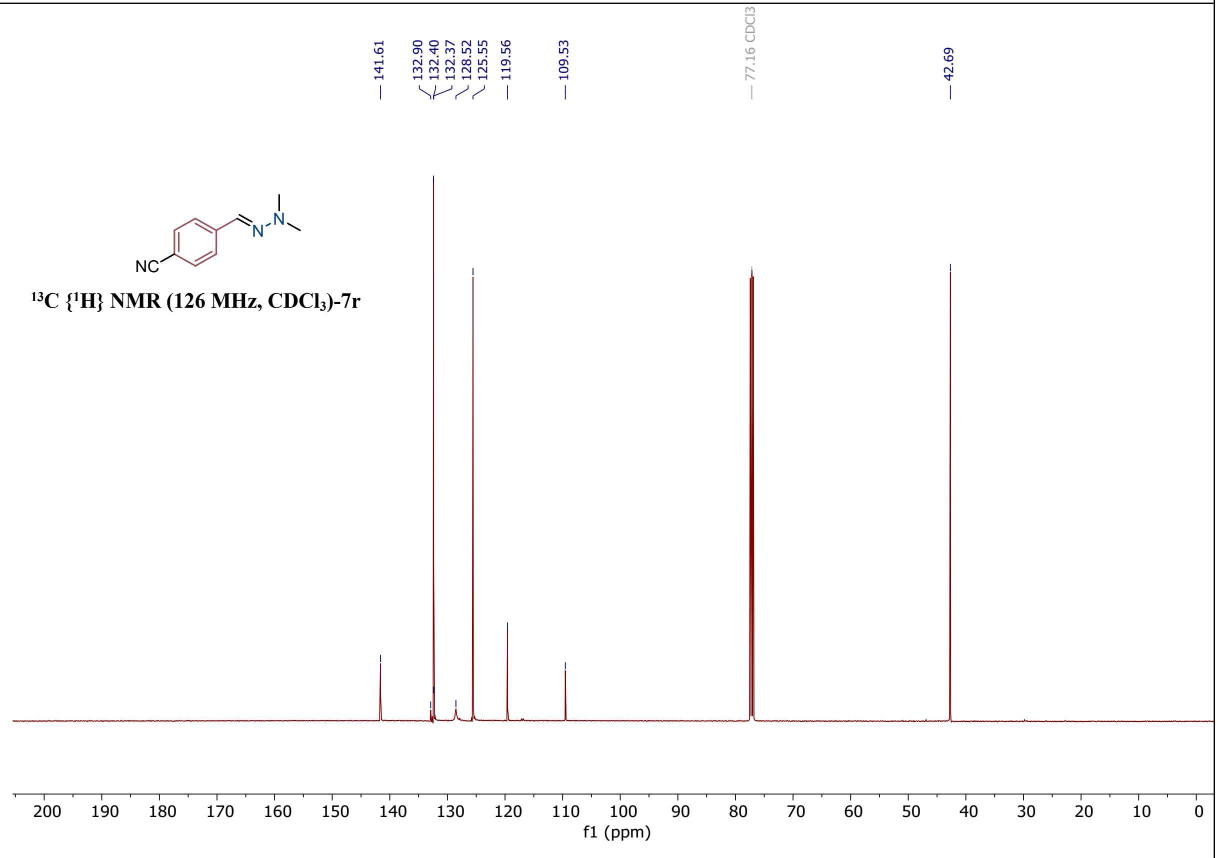

(*E*)-4-((2,2-dimethylhydrazineylidene)methyl)benzonitrile(7r)

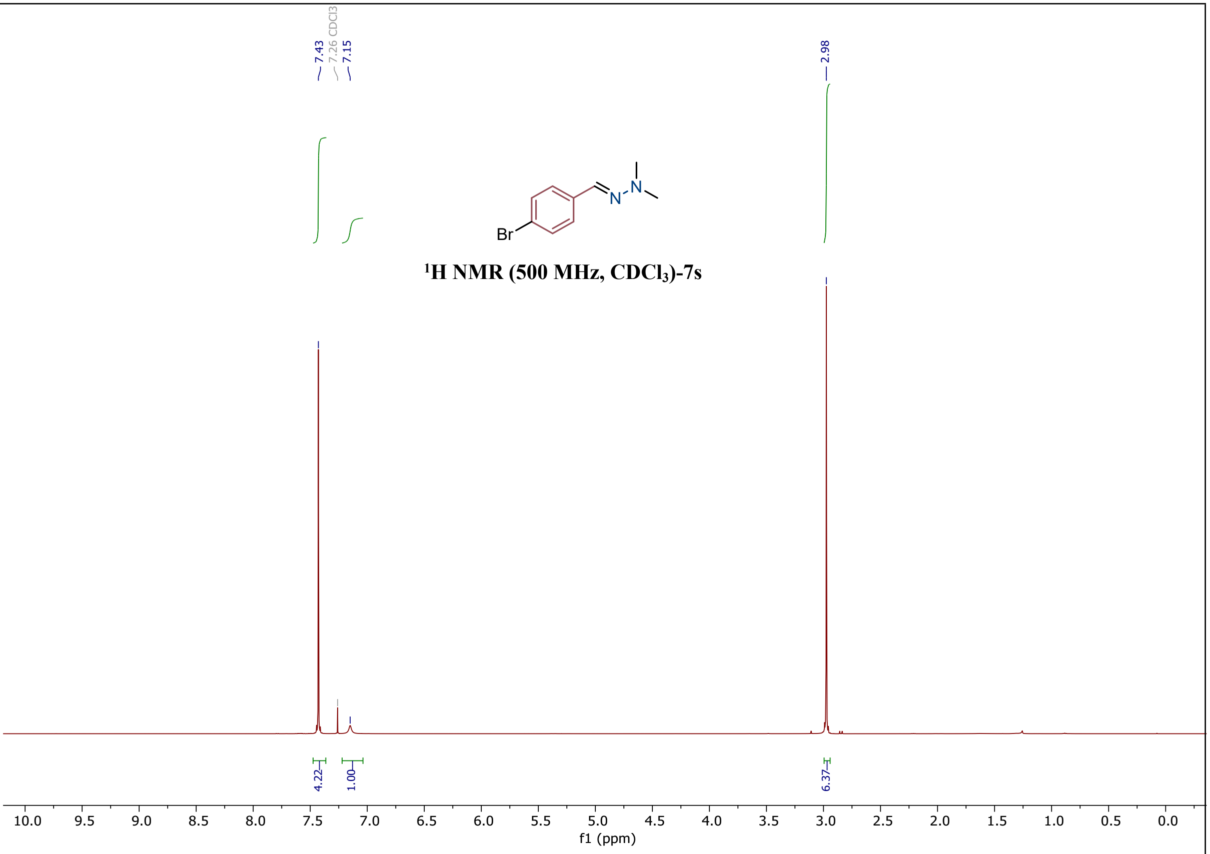

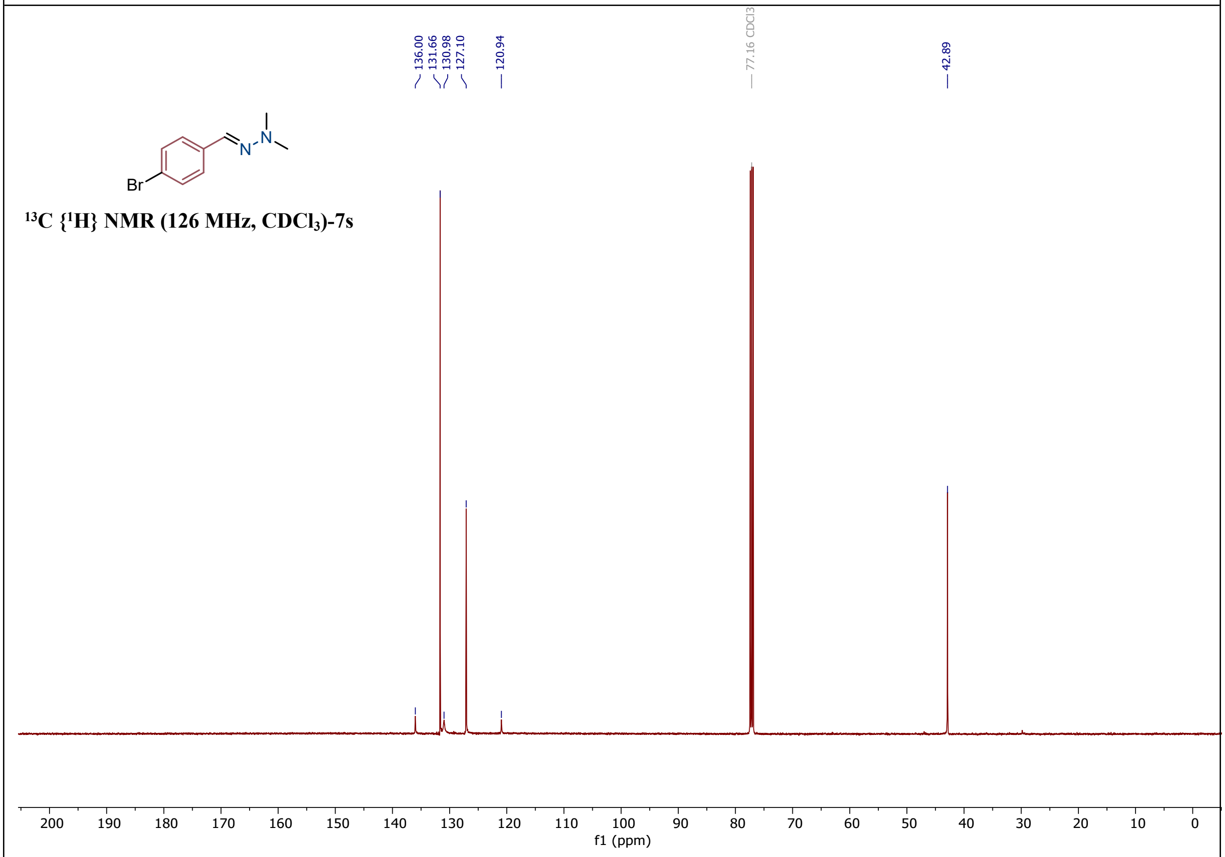

(*E*)-2-(4-bromobenzylidene)-1,1-dimethylhydrazine (7s)

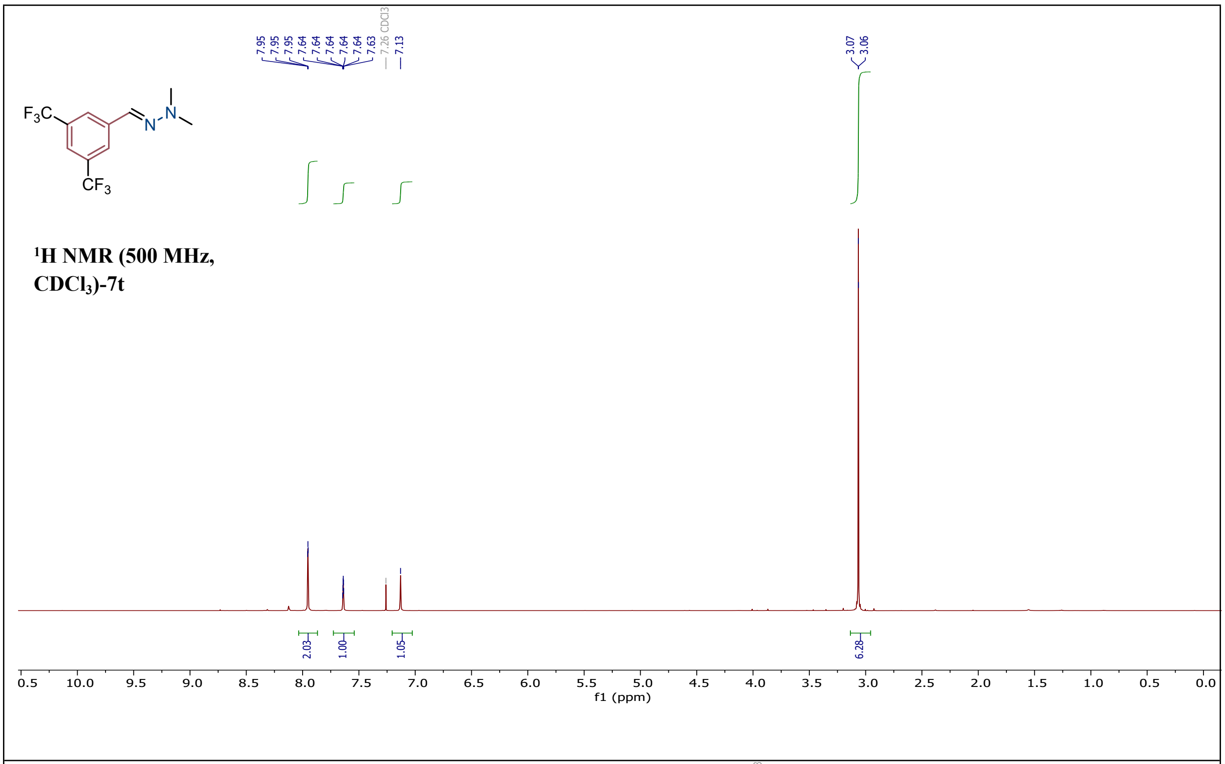

¹H NMR (500 MHz, CDCl₃)-7t
f1 (ppm)

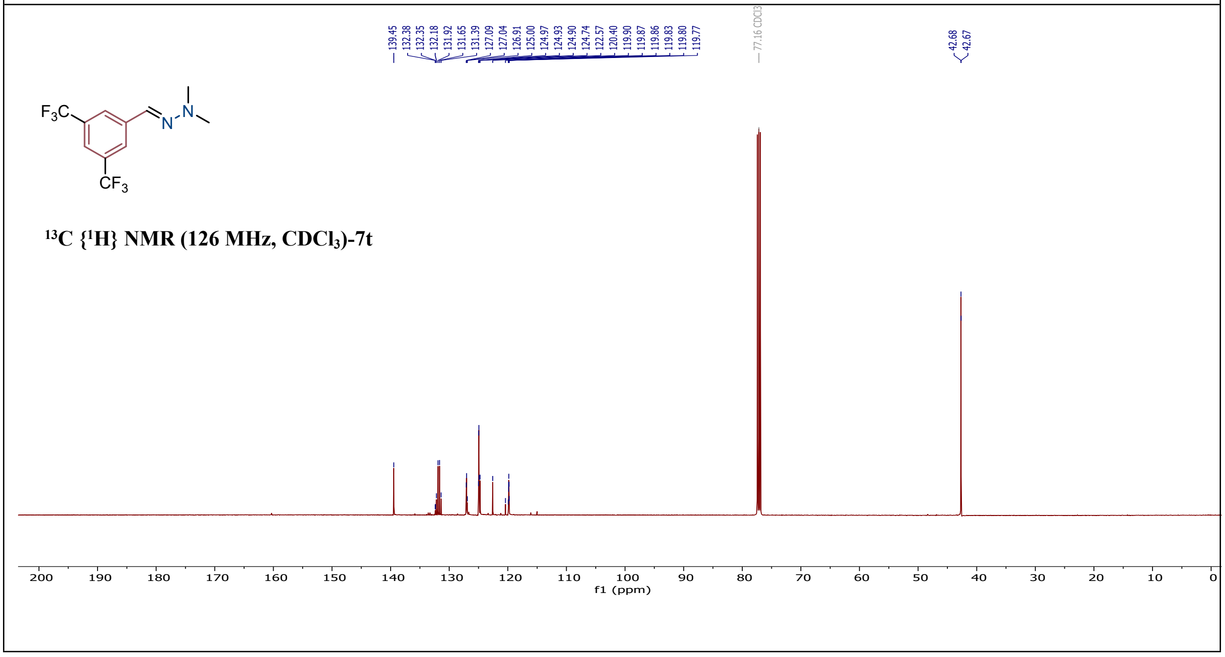

¹³C {¹H} NMR (126 MHz, CDCl₃)-7t
f1 (ppm)

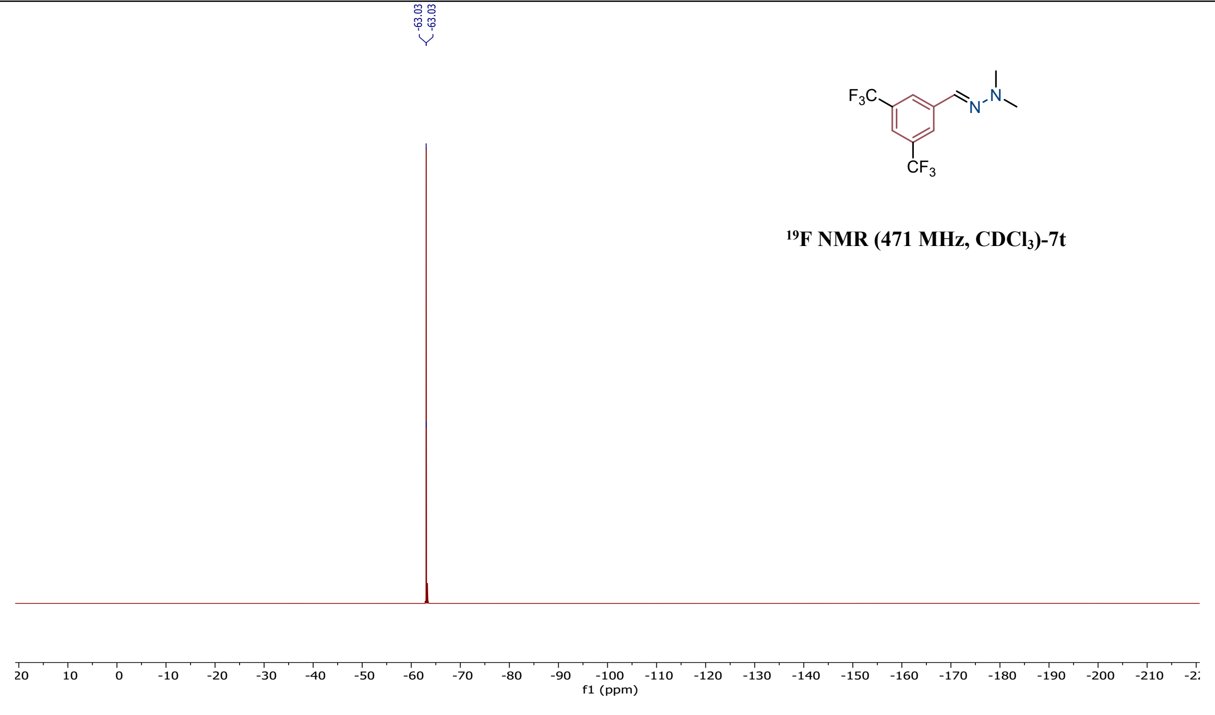

(*E*)-2-(3,5-*bis*(trifluoromethyl)benzylidene)-1,1-dimethylhydrazine(7t)

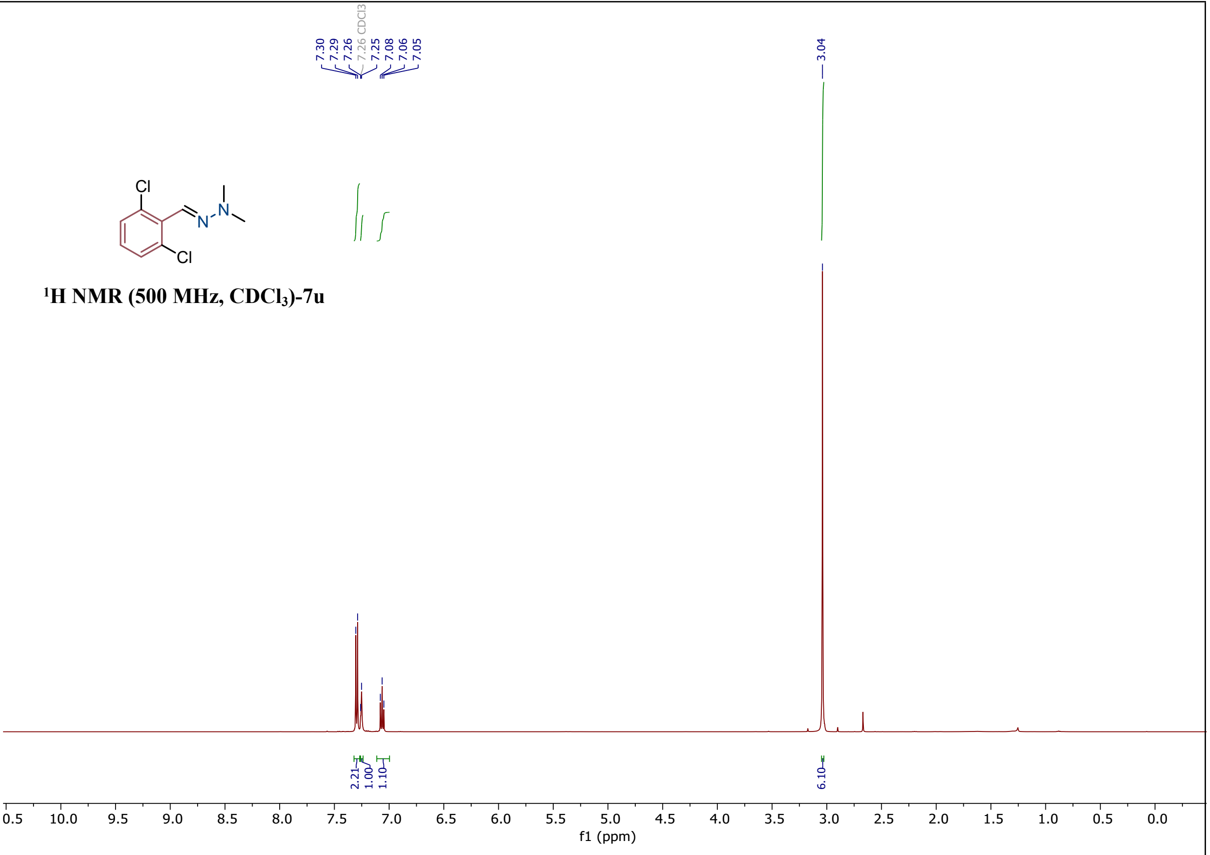

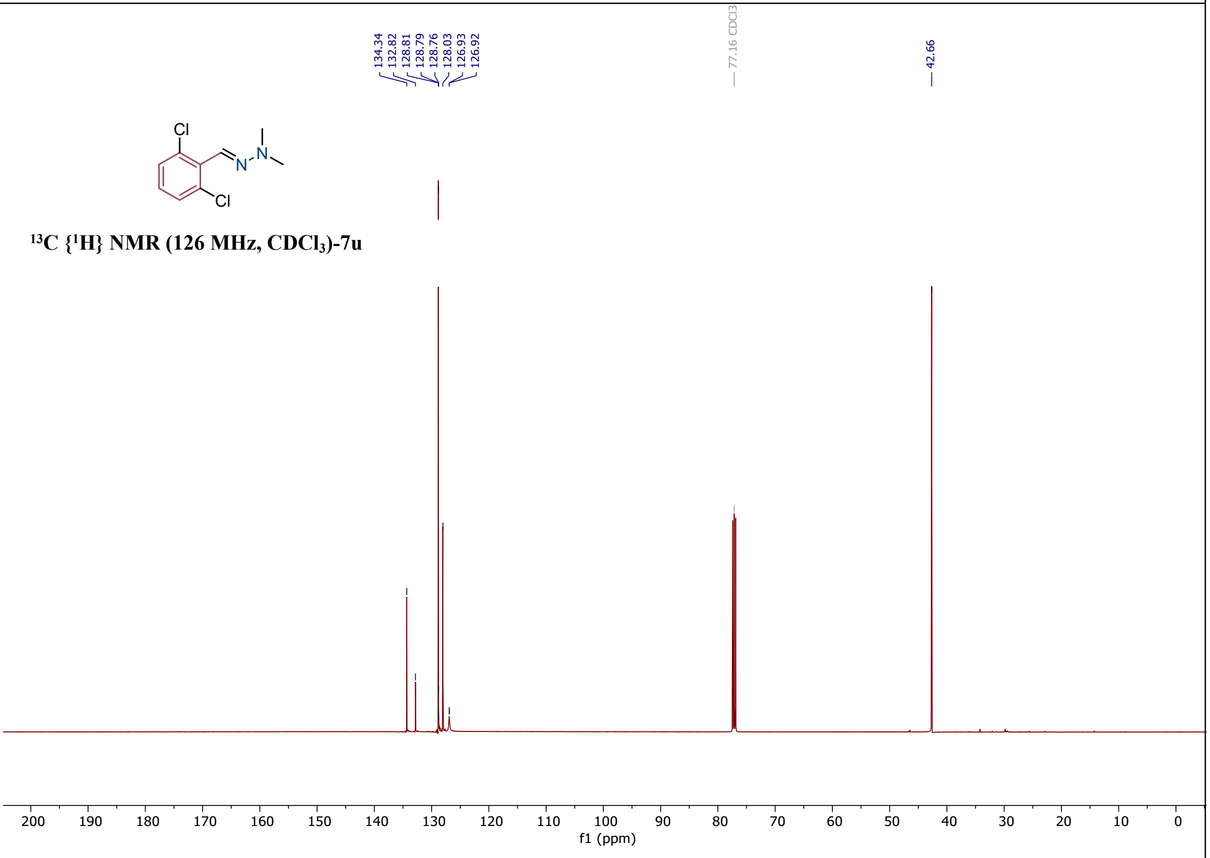

(*E*)-2-(2,6-dichlorobenzylidene)-1,1-dimethylhydrazine(7u)

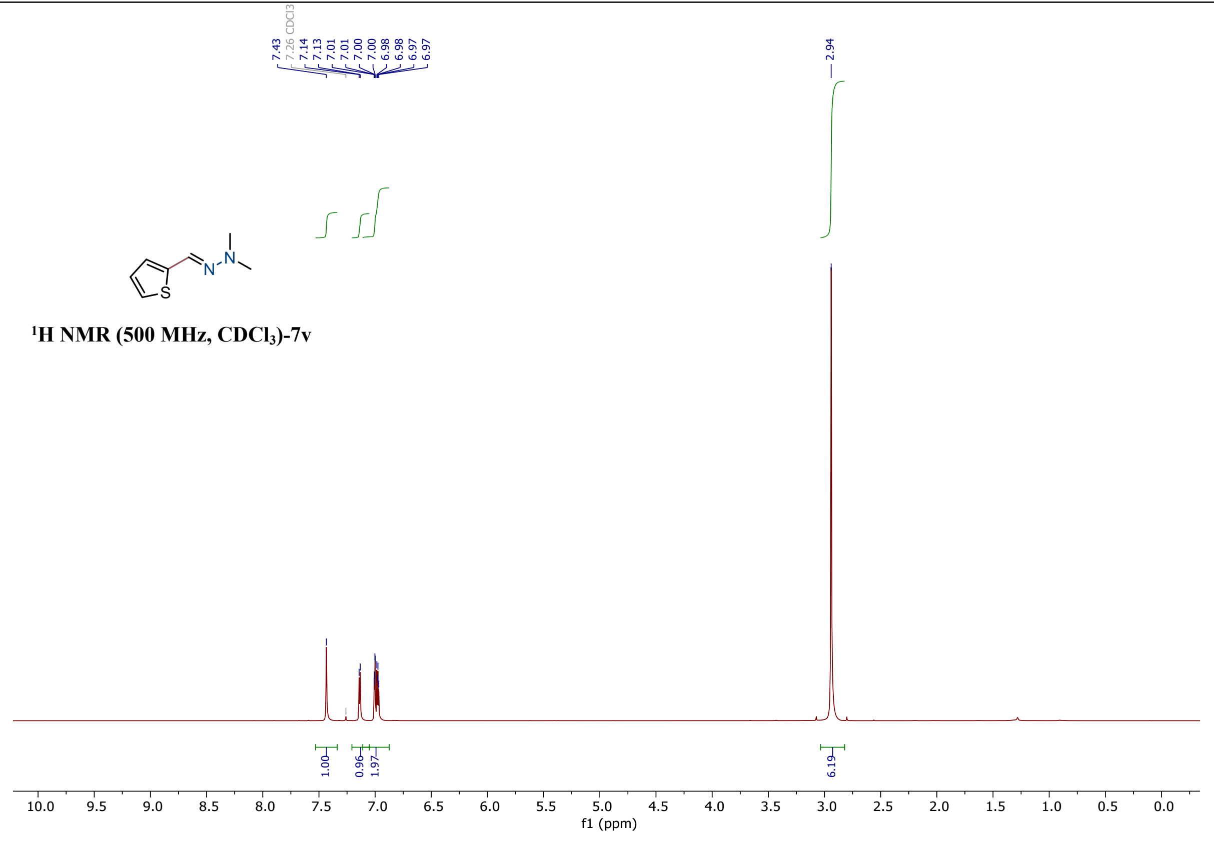

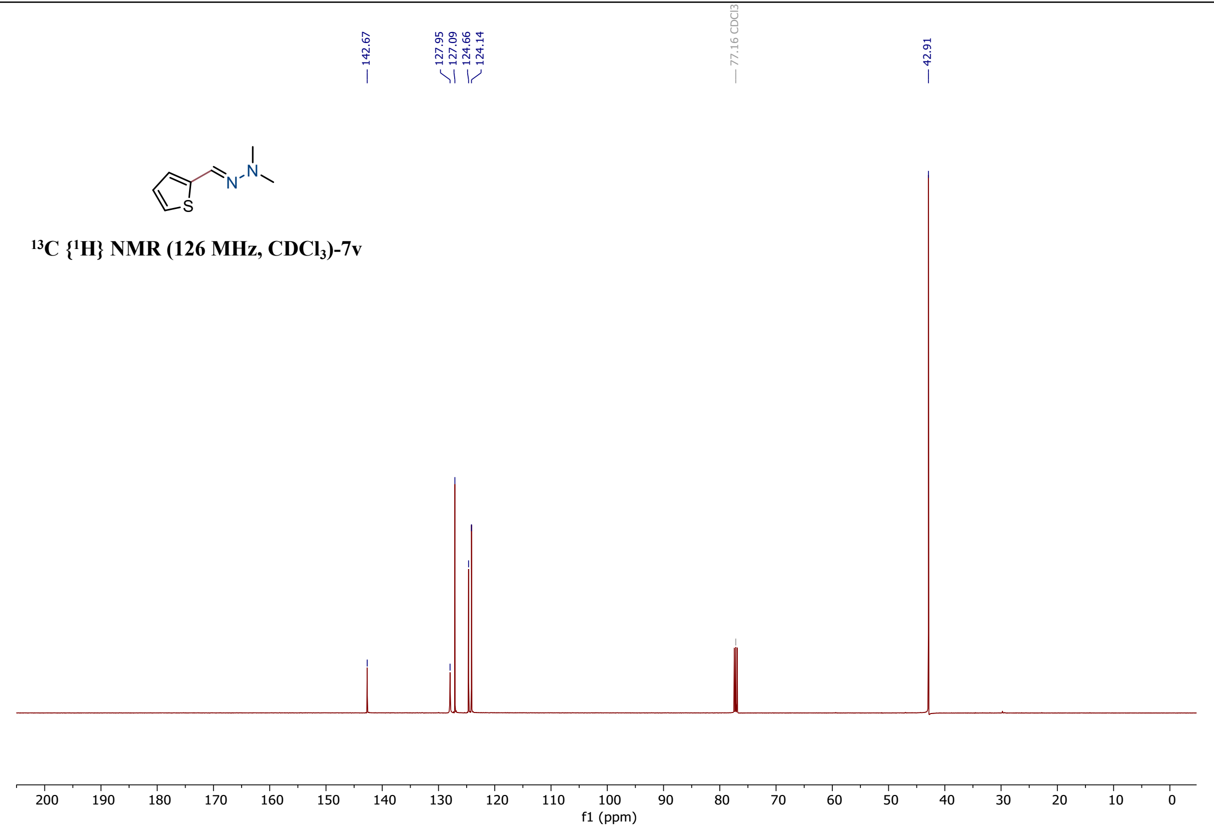

(*E*)-1,1-dimethyl-2-(thiophen-2-ylmethylene)hydrazine(7v)

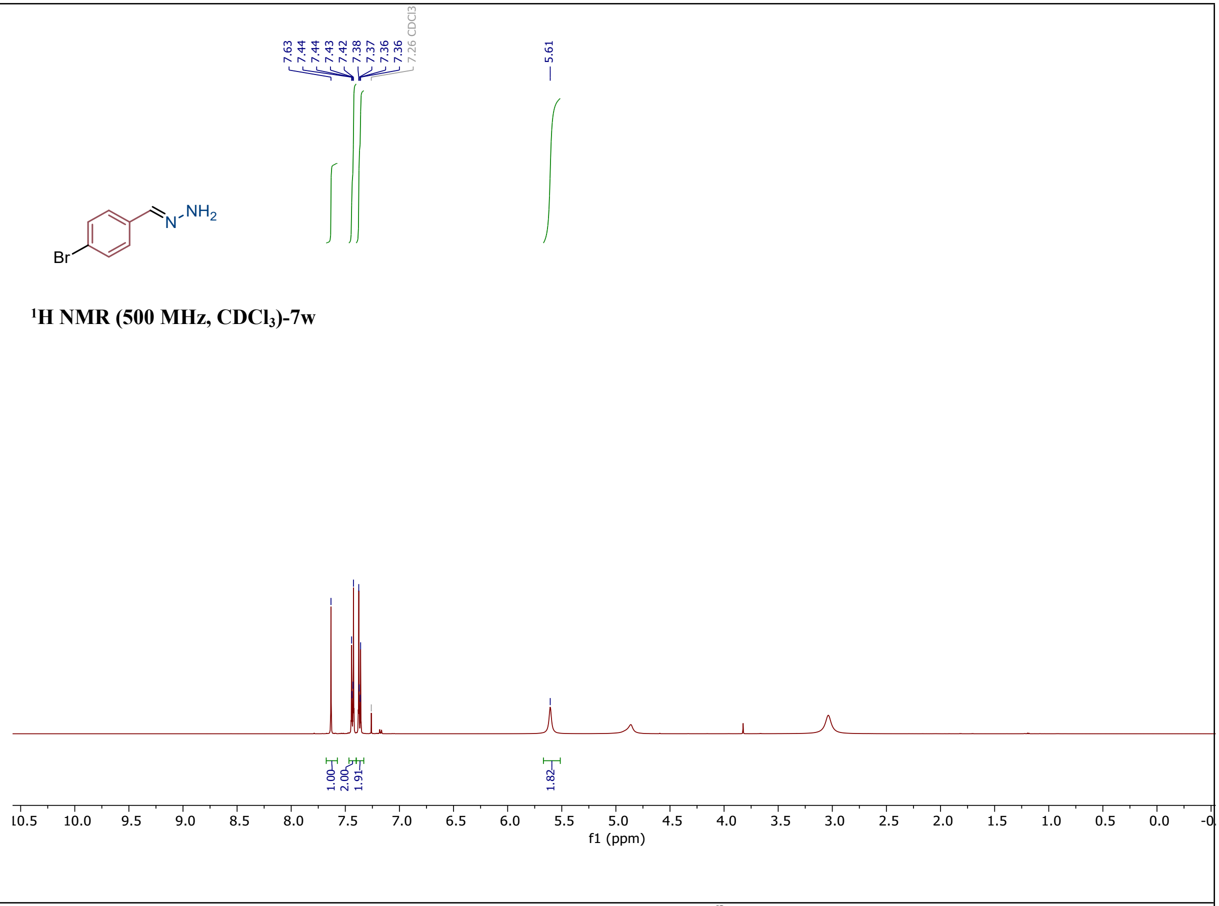

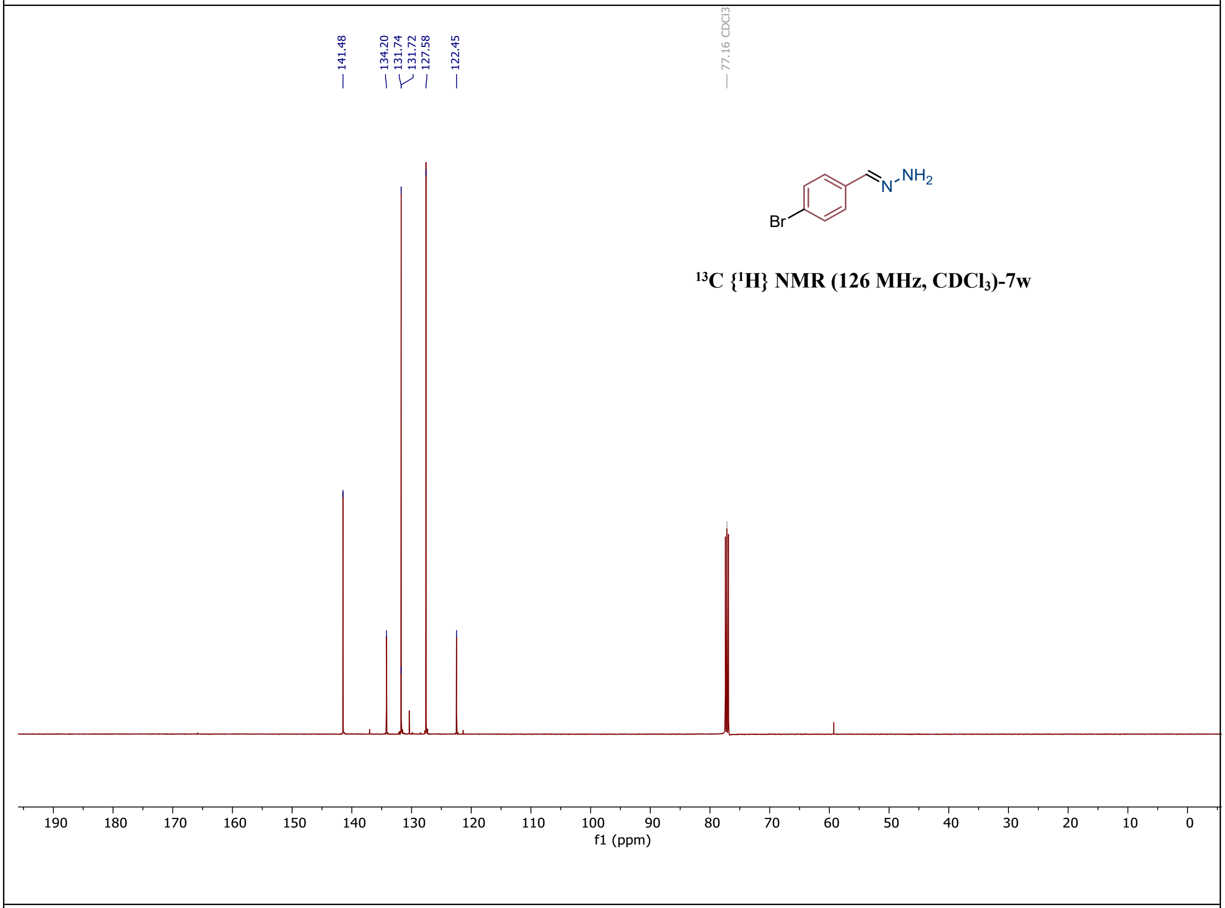

(*E*)-(4-bromobenzylidene)hydrazine(7w)

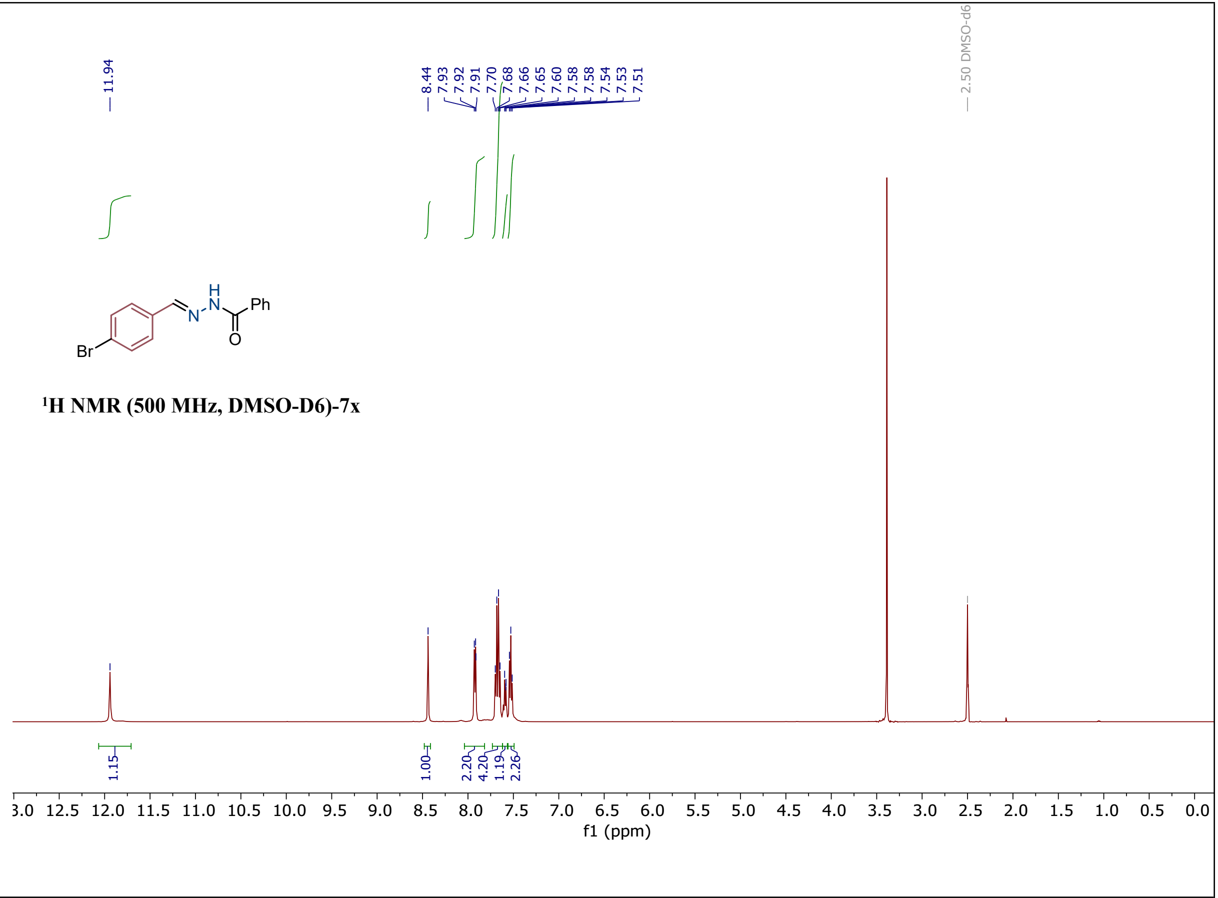

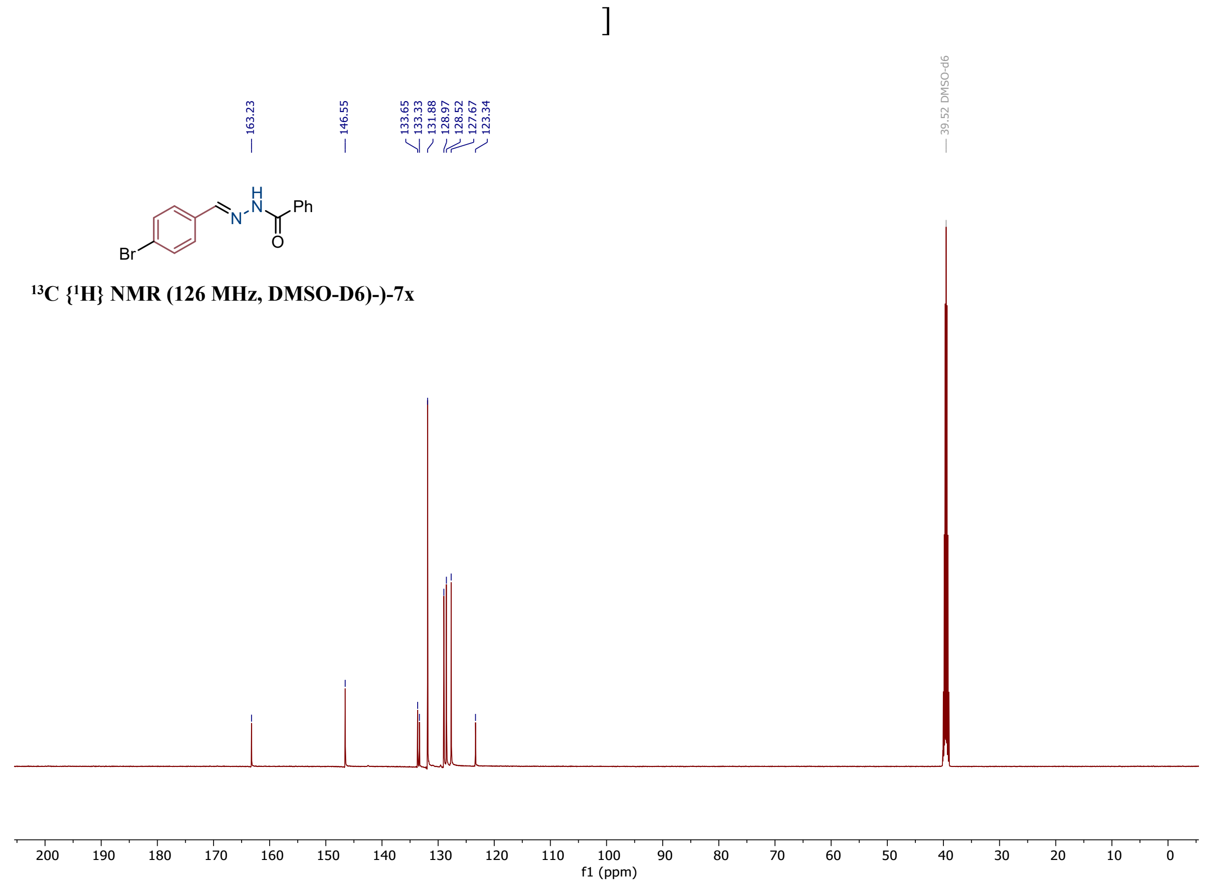

(*E*)-*N'*-(4-bromobenzylidene)benzohydrazide(7x)

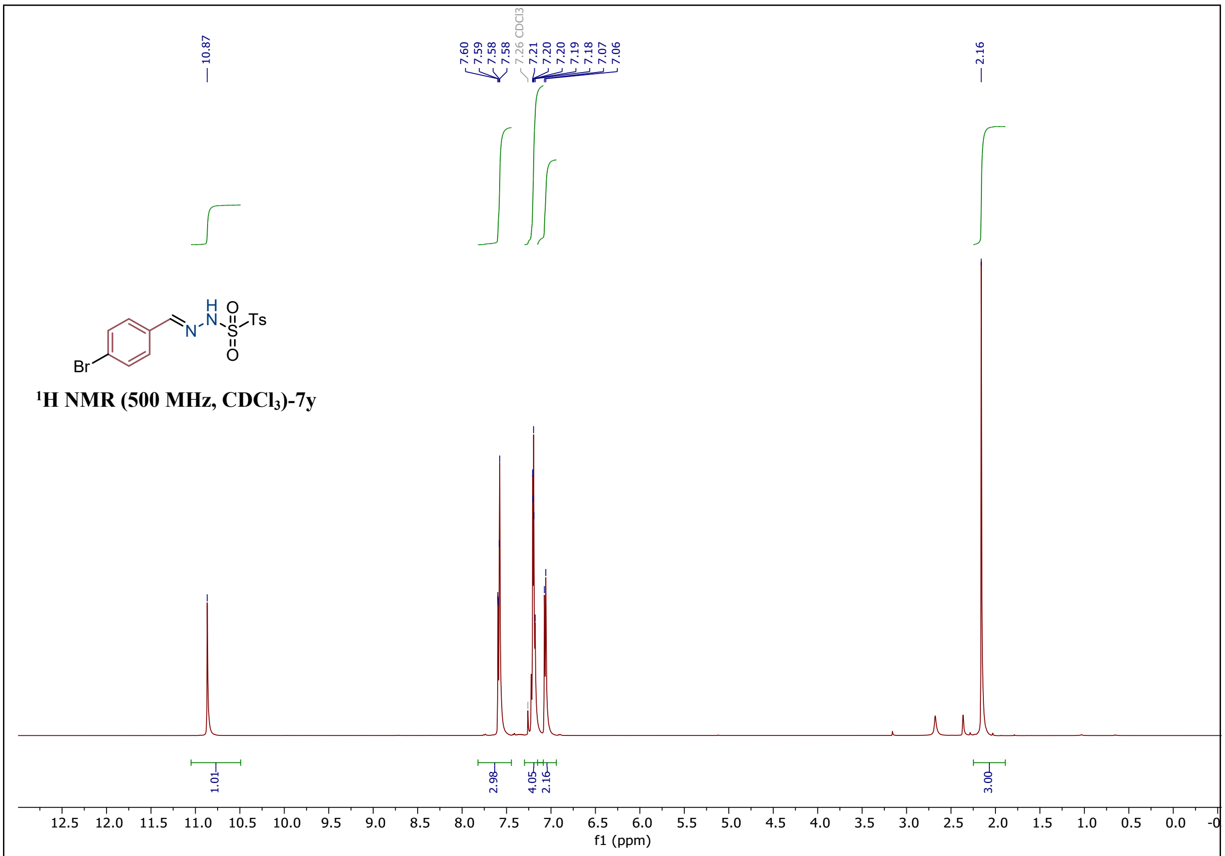

^{1}H NMR (500 MHz, CDCl$_3$)-7y

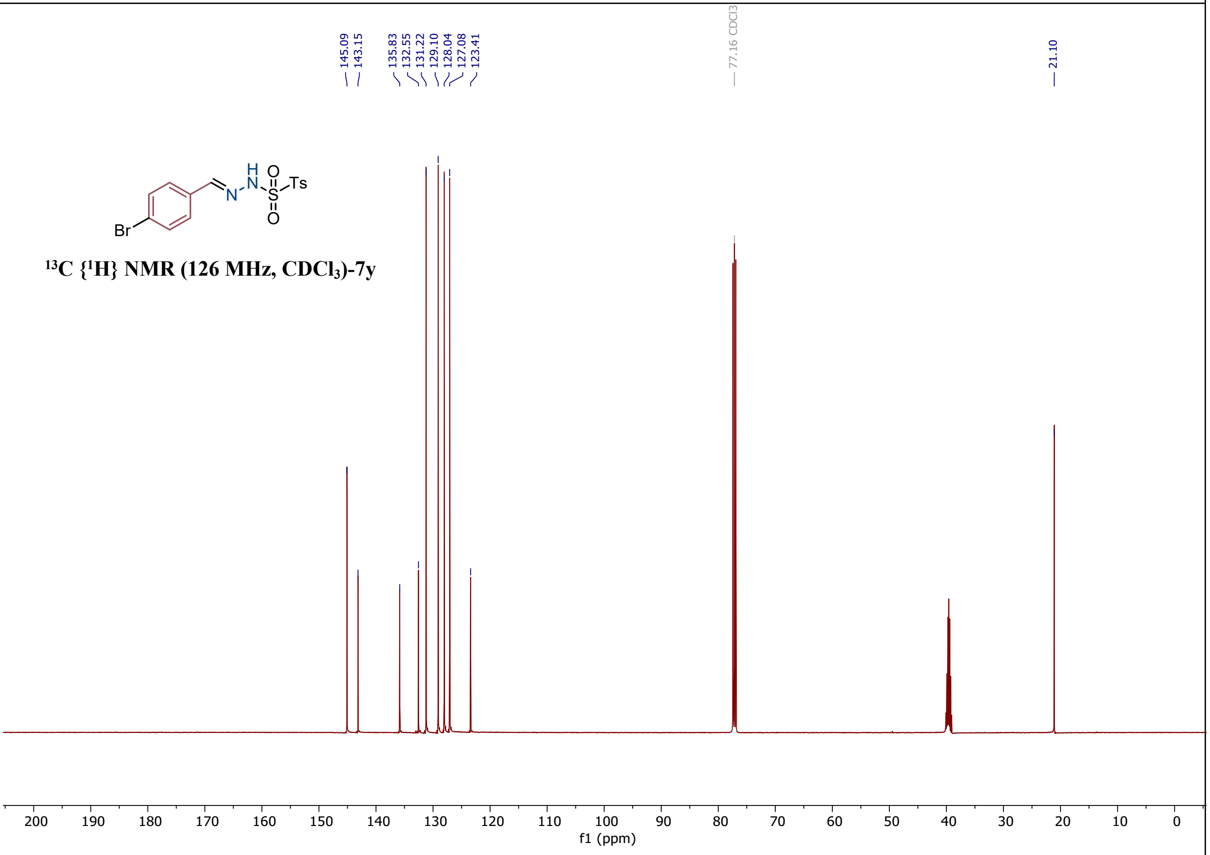

^{13}C {^{1}H} NMR (126 MHz, CDCl$_3$)-7y

(*E*)-*N'*-(4-bromobenzylidene)-4-methylbenzenesulfonohydrazide(7y)

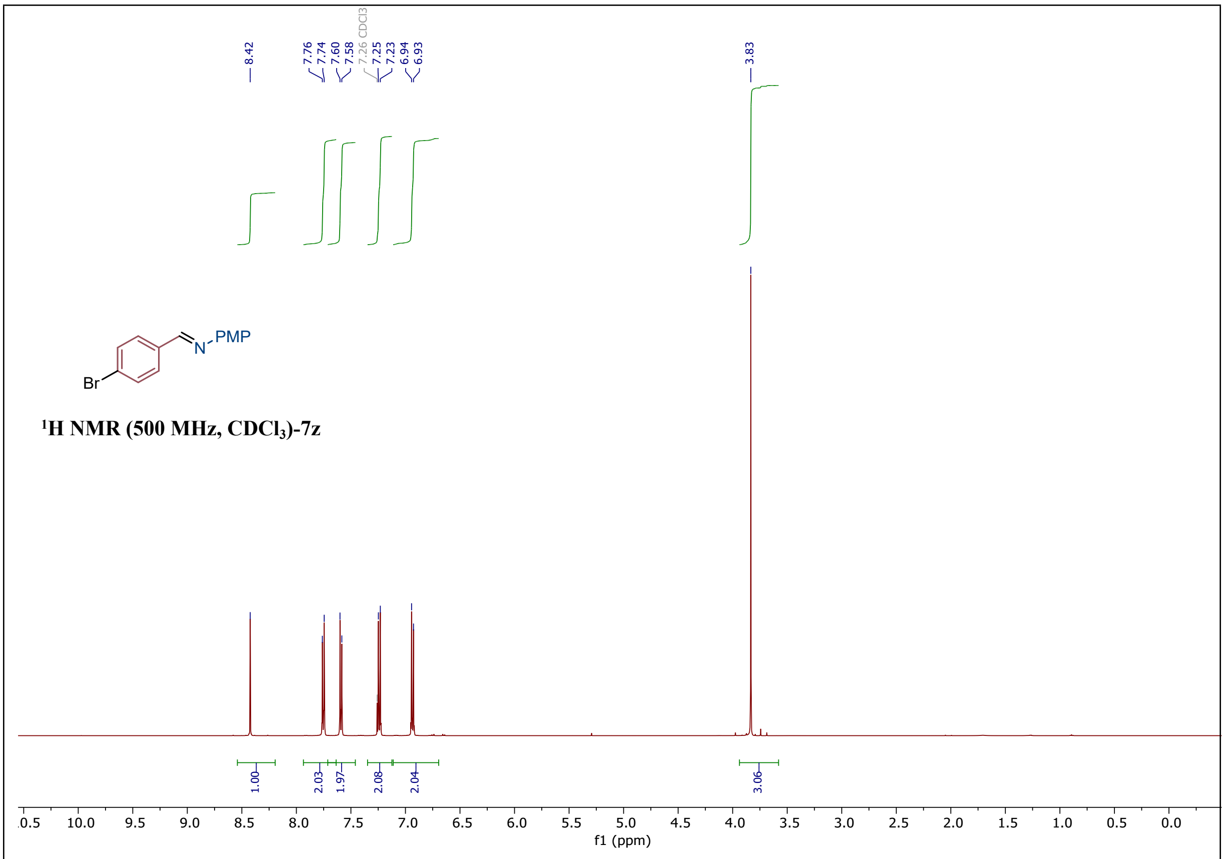

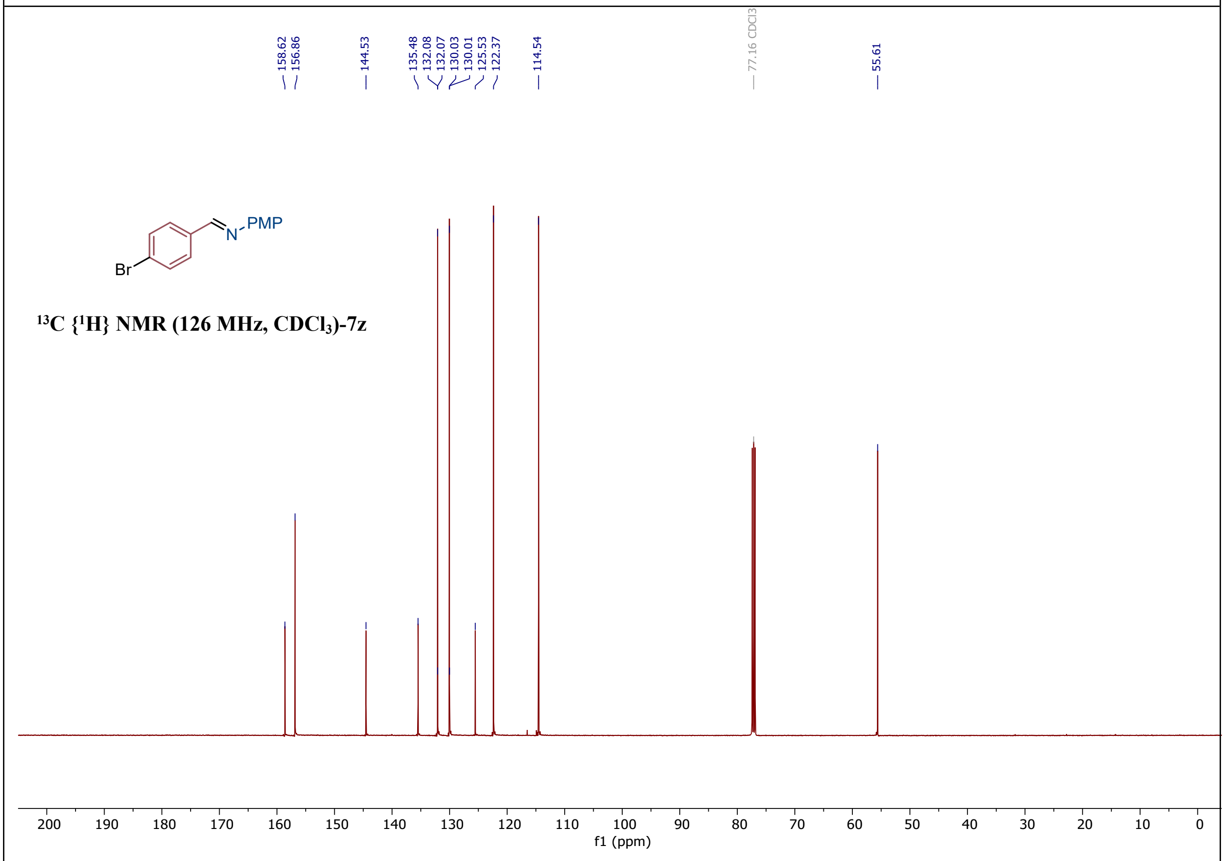

(*E*)-1-(4-bromophenyl)-*N*-(4-methoxyphenyl)methanimine(7z)

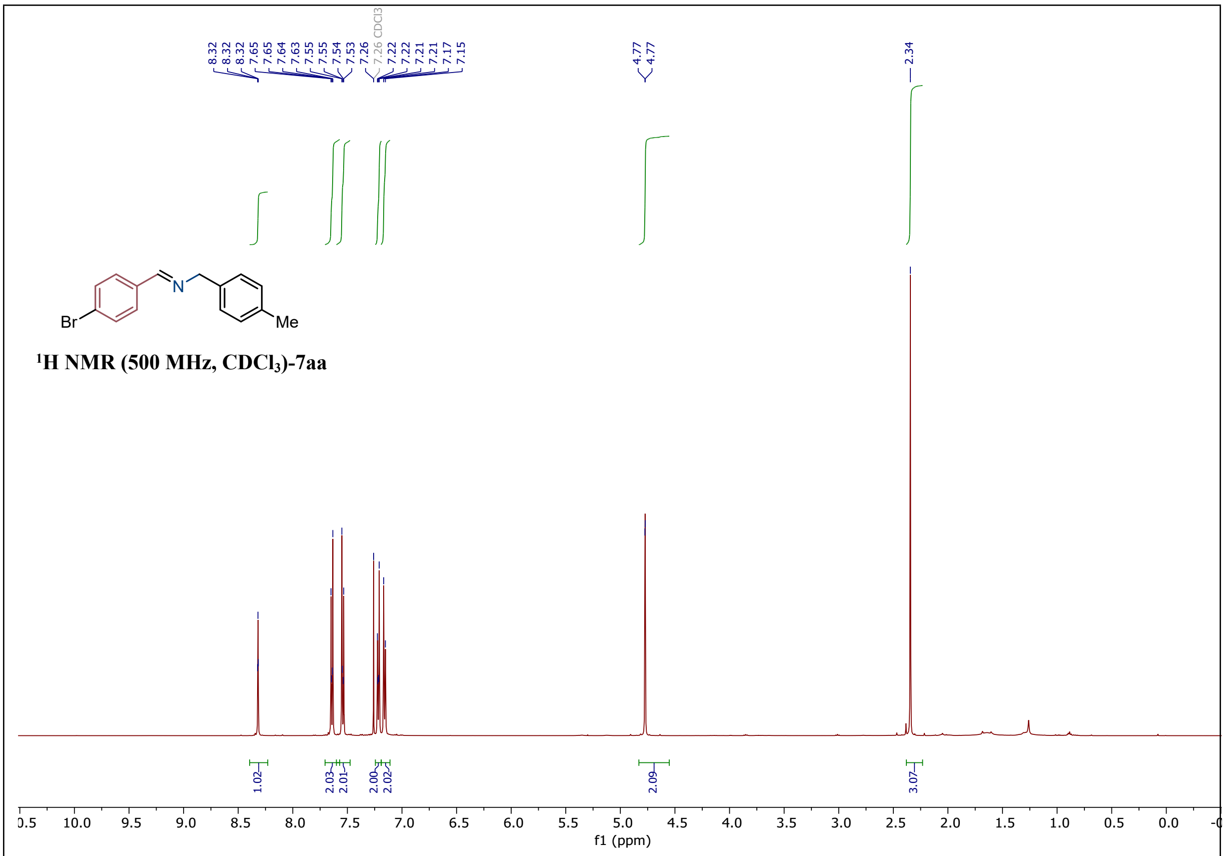

^{1}H NMR (500 MHz, $CDCl_3$)-7aa

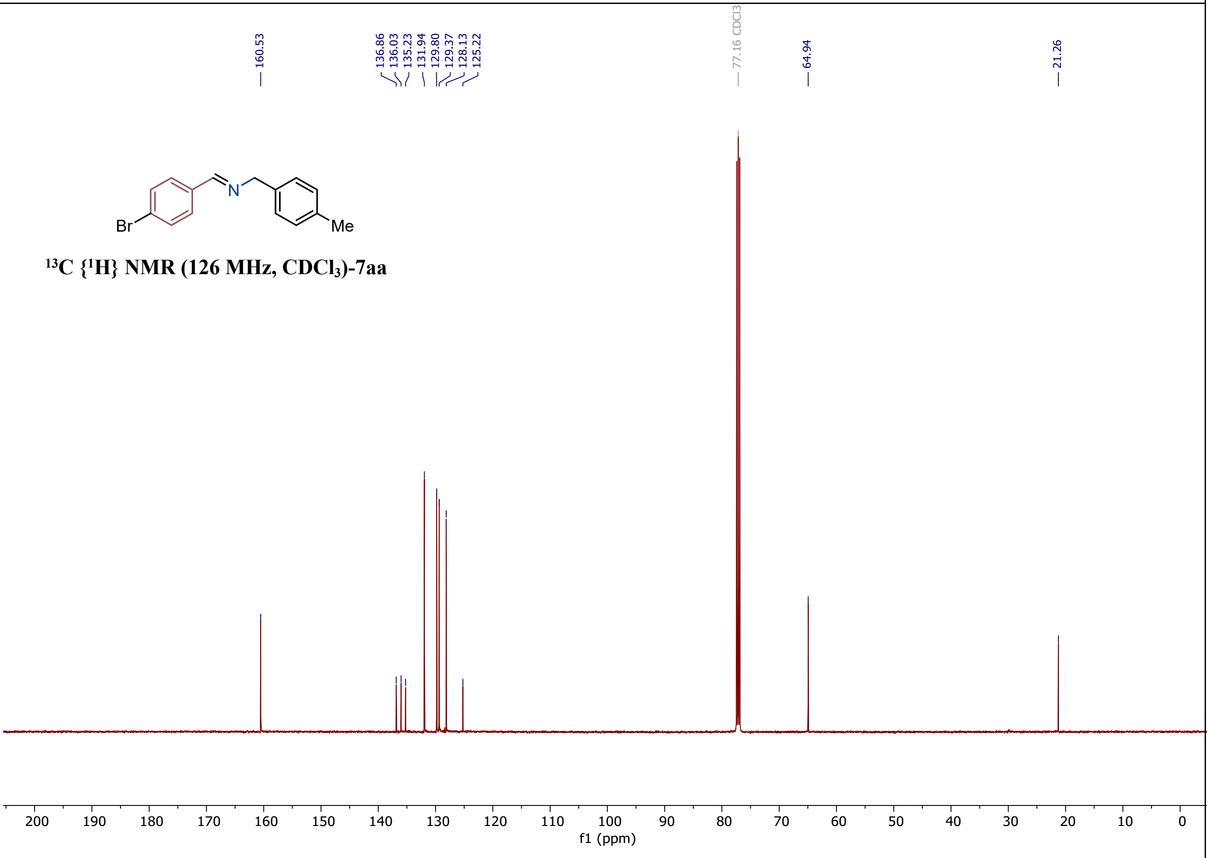

^{13}C {^{1}H} NMR (126 MHz, $CDCl_3$)-7aa

(*E*)-1-(4-bromophenyl)-*N*-(4-methylbenzyl)methanimine(7aa)

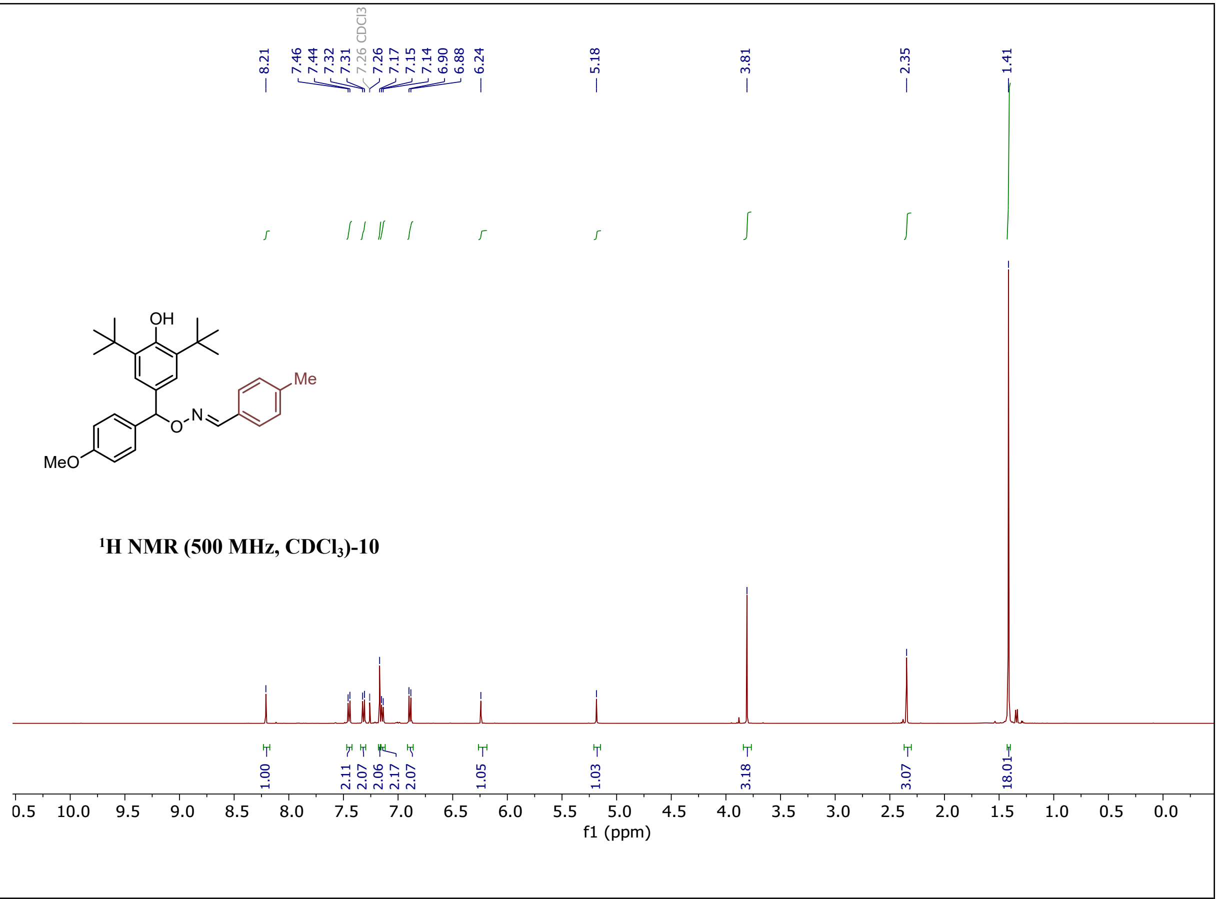

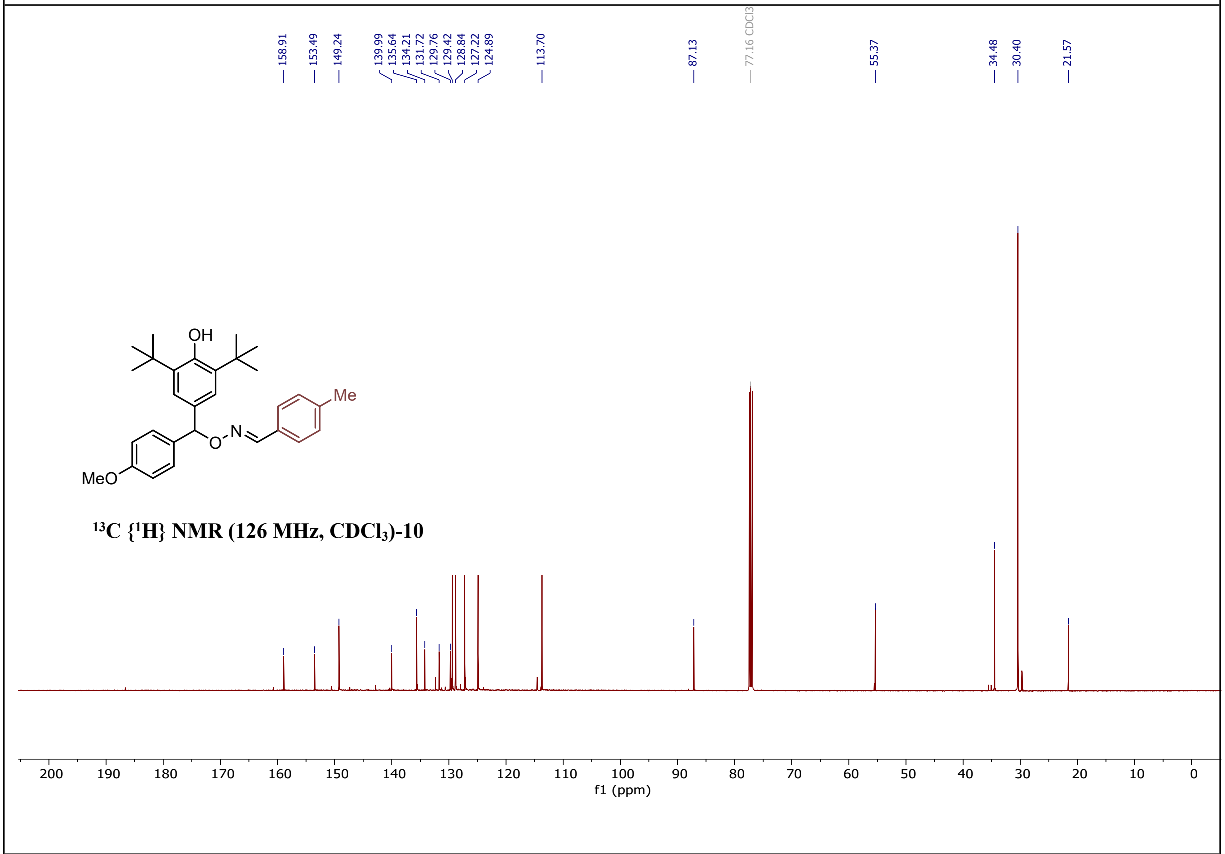

(*E*)-4-methylbenzaldehyde *O*-((3,5-*di*-*tert*-butyl-4-hydroxyphenyl)(4-methoxyphenyl) methyl) oxime (10)

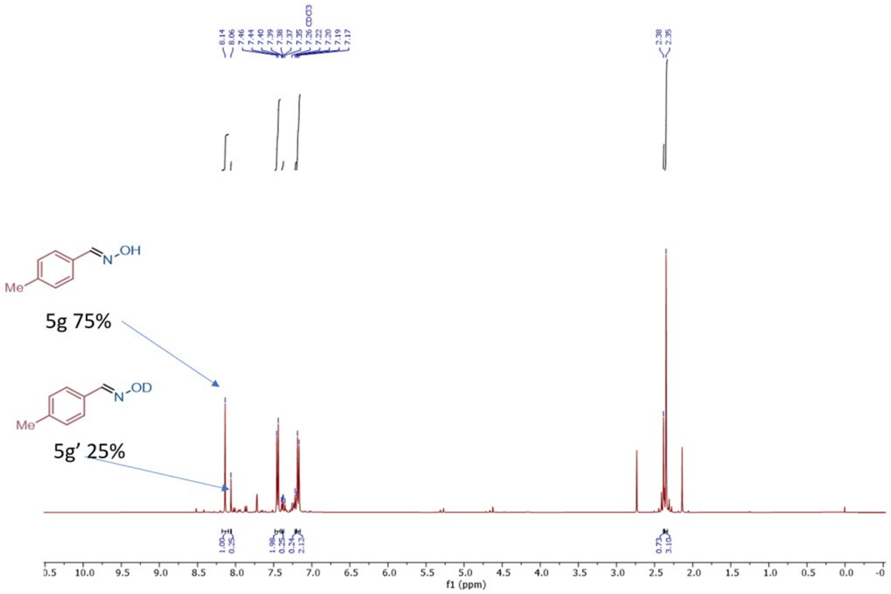

^{1}H NMR Spectra of the reaction carried out in deuterated water as solvent.
